# Supplementary material for: Multicomponent mapping of boron chemotypes furnishes selective enzyme inhibitors
Source: Nat Commun. 2017 Nov 24;8:1760. doi: 10.1038/s41467-017-01319-4 (PMC5701053; doi:10.1038/s41467-017-01319-4)
Supplement: Supplementary file 2 — Supplementary Information [file 41467_2017_1319_MOESM2_ESM.pdf]

Supplementary Methods

General Information: Methylene chloride (DCM), methanol (MeOH) and triethylamine were distilled from CaH_2 under nitrogen. Acetonitrile (MeCN) was distilled from activated 4Å MS under nitrogen. Toluene was purified via solvent purification system. Tetrahydrofuran (THF) was freshly distilled from sodium benzophenone ketyl. All other solvents were of reagent grade quality and dried over 4Å MS prior to use. All reagents were purchased from commercial sources and used as received.

Chromatography: Flash column chromatography was carried out using Silicycle 230-400 mesh silica gel, or ISCO Teledyne Combiflash R_f 200 Flash system. Thin-layer chromatography (TLC) was performed on Macherey Nagel pre-coated glass backed TLC plates (SIL G/UV254, 0.25 mm) and visualized using a UV lamp (254 nm), $KMnO_4$ or curcumin stain (preparation: 200 mg curcumin in 100 mL ethanol). Reverse-phase chromatography was carried out using Redi*Sep* Rf Gold C18 Columns.

Nuclear Magnetic Resonance Spectroscopy: ^{1}H NMR, ^{13}C, and 2D NMR spectra were recorded on Varian Mercury 300 MHz, 400 MHz, 500 MHz, 600 MHz or 700 MHz spectrometers. ^{11}B NMR were recorded using Bruker 400/500 MHz spectrometer at 128/160 MHz and referenced to an external standard of $BF_3{\cdot}Et_2O$ (δ = 0 ppm). ^{1}H NMR spectra chemical shifts (δ) are reported in parts per million (ppm) referenced to residual protonated solvent peak (CD_3CN δ = 1.94, DMSO-d_6, δ = 2.49, CD_3OD δ = 3.31 center line). Spectral data is reported as follows: chemical shift, multiplicity (s = singlet, d = doublet, t = triplet, q = quartet, dd = doublet of doublets, dt = doublet of triplets, ddt = doublet of doublet of triplets, dtd = doublet of triplet of doublets, m = multiplet, br = broad), coupling constant (J) in Hertz (Hz), and integration. ^{13}C NMR spectra chemical shifts (δ) are reported in parts per million (ppm) were referenced to carbon resonances in the NMR solvent (CD_3CN δ = 118.3, DMSO-d_6, δ = 39.5, CD_3OD δ = 49.0; center line). Carbons exhibiting significant line broadening brought about by boron substituents were not reported (quadrupolar relaxation). Rotameric peaks were resolved into single peaks using variable temperature NMR for compounds **4g-4k**.

Mass Spectroscopy: High resolution mass spectra were obtained on a VG 70- 250S (double focusing) mass spectrometer at 70 eV or on an ABI/Sciex Qstar mass spectrometer with ESI source, MS/MS and accurate mass capabilities. Low resolution mass spectra were obtained on an Agilent Technologies 1200 series HPLC paired to a 6130 Mass Spectrometer.

RP-HPLC/MS: Low-resolution mass spectra (ESI) were collected on an Agilent Technologies 1200 series HPLC paired to a 6130 Mass Spectrometer. Compounds were resolved on Phenomenex's Kinetex 2.6u C18 50x4.6mm column at room temperature with a flow of 1 mL/min. The gradient consisted of eluents A (0.1% formic acid in double distilled water) and B (0.1% formic acid in HPLC-grade acetonitrile).
Method A: A linear gradient starting from 5% of B to 95% over 15 min at a flow rate of 1.0 mL/min.

Synthesis of parent α-boryl aldehyde (1a)

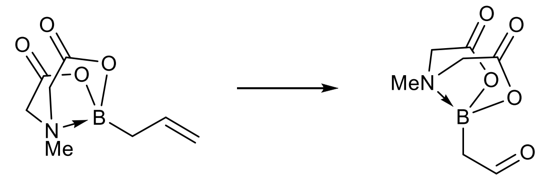

1a

Note: Although the synthesis of parent boryl aldehyde (**1a**) was previously reported by the Yudin lab[1], we have since improved the purification of **1a** using the procedure below.

Step 1: Allyl-MIDA boronate

Allyl-MIDA boronate was prepared according to literature[1].

Step 2: Ozonolysis

In a 3-L round-bottom flask equipped with a magnetic stir bar under nitrogen atmosphere was added allyl-MIDA boronate (37 g, 188 mmol) followed by reagent grade DCM (1.4 L) and MeOH (450 mL). The mixture was stirred for 10 minutes and resulted in a clear solution. The gas diffuser was placed into the solution and attached to an ozone generator. The generator was then set to 0.55 g/min and allowed to stir at -78 °C for 3.5 hours at which time the reaction mixture started to turn blue. The ozone was then stopped and N_2(g) was bubbled through the solution until colourless. A sample was then analyzed by ^{1}H NMR and showed complete consumption of starting material. The flask was then charged with Me_2S (58.34 g, 69 mL, 939 mmol, 5.0 equiv) and stirred at -78 °C for one hour then warmed to room temperature overnight. After stirring overnight, a white suspension had formed. The solvent was then removed *in vacuo* to yield a white solid. DCM* (150 mL) was introduced and the suspension was filtered to yield the product plus a minor impurity. The white solid was dissolved in MeCN and stirred overnight to solubilize the product. The impurity was filtered off, the organic layer was collected and the solvent was removed *in vacuo* to afford pure product as a white solid.

*The DCM layer can be collected and re-purified by to retrieve parent boryl aldehyde **1a** that was solubilized by any remaining DMSO.

General preparation of α-substituted boryl aldehydes (1b-g)

α-Substituted boryl aldehydes **1b-g** used for the synthesis of β-amino(MIDA)boronates were prepared according to literature method[2,3]. List of known α-substituted boryl aldehydes used:

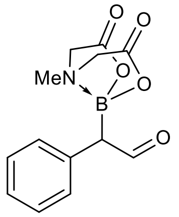

1b

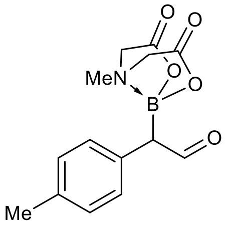

1c

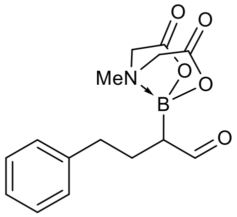

1d

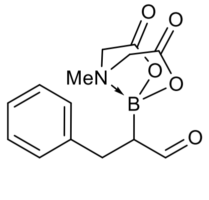

1e

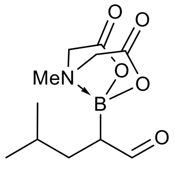

1f

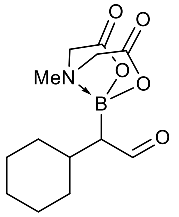

1g

General preparation of β-amino(MIDA)boronates (3)

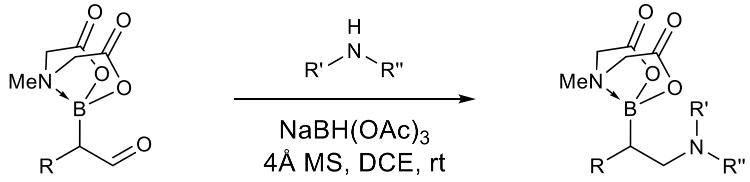

1 → 3

β-amino(MIDA)boronates **3a-3k** were prepared according to literature methods[4]. List of known β-amino(MIDA)boronates previously characterized:

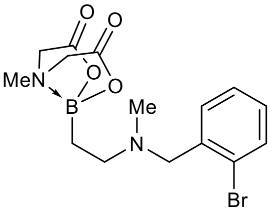

3a

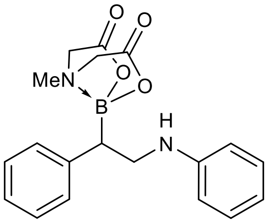

3b

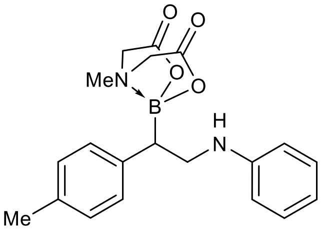

3c

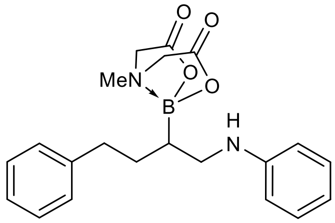

3d

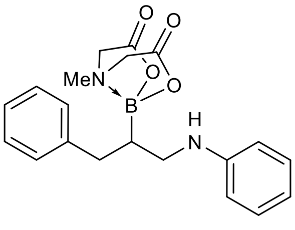

3e

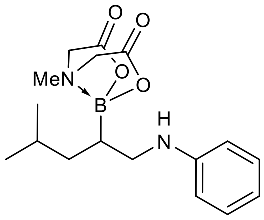

3f

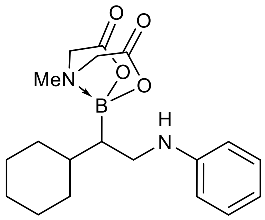

3g

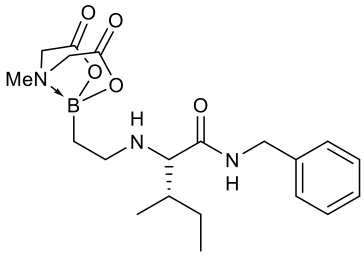

3h

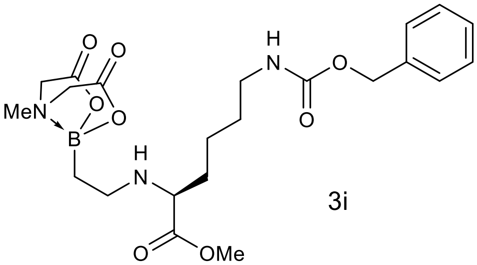

3i

MIDA (2-(3,4-dihydroisoquinolin-2(1H)-yl)ethyl)boronate (3j)

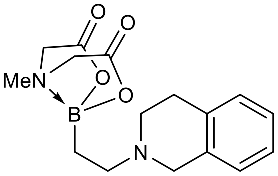

White solid; yield = 38.2 mg, 60 %; R_f = 0.36 (2:3, EtOAc:MeCN, with 1% TEA on pre-deactivated silica plate). ^{1}H NMR (400 MHz, CD_3CN) δ 0.88 - 0.99 (m, 2H), 2.48 - 2.58 (m, 2H), 2.68 (t, J = 5.9 Hz, 2H), 2.84 (t, J = 5.9 Hz, 2H), 2.87 (s, 3H), 3.51 - 3.61 (m, 2H), 3.81 (d, J = 16.8 Hz, 2H), 3.91 (d, J = 16.8 Hz, 2H), 6.98 - 7.06 (m, 1H), 7.06 - 7.15 (m, 3H); ^{13}C NMR (101 MHz, CD_3CN) δ 169.2, 136.3, 135.6, 129.5, 127.4, 126.9, 126.5, 62.8, 56.6, 55.0, 51.7, 46.7, 29.9; ^{11}B NMR (128 MHz, CD_3CN) δ 13.1; HRMS (DART-MS): *m/z* for $C_{16}H_{22}BN_2O_4$: calculated = 317.1673, found = 317.1675 [$M+H^+$].

MIDA (2-((4-bromophenyl)amino)ethyl)boronate (3k)

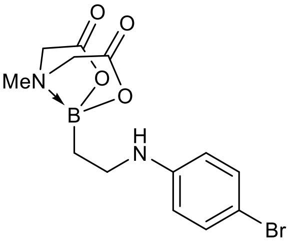

White solid; yield = 286 mg, 81%, R_f = 0.69 (1:1, EtOAc:MeCN, with 1% TEA on pre-deactivated silica plate). ^{1}H NMR (500 MHz, CD_3CN) δ 0.90 - 1.00 (m, 2H), 2.87 (s, 3H), 3.05 - 3.15 (m, 2H), 3.81 (d, J = 16.9 Hz, 2H), 3.95 (d, J = 16.9 Hz, 2H), 4.36 - 4.48 (m, 1H), 6.50 - 6.54 (m, 2H), 7.18 - 7.23 (m, 2H); ^{13}C NMR (126 MHz, CD_3CN) δ 169.1, 149.3, 132.5, 115.1, 107.9, 62.7, 46.8, 40.7; ^{11}B NMR (128

MHz, CD_3CN) δ 12.7; HRMS (DART-MS): *m/z* for $C_{13}H_{17}BBrN_2O_4$: calculated = 355.0446, found = 355.0449 [$M+H^+$].

General preparation of β,β-aminocyano(MIDA)boronates (4)

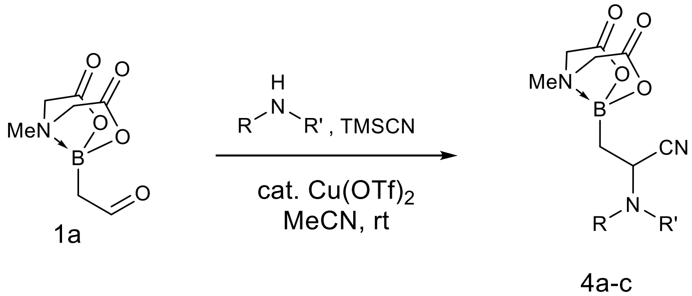

To a flame-dried, round bottom flask equipped with a magnetic stir bar under nitrogen atmosphere was added α-boryl aldehyde **1a** (1.0 equiv.), copper (II) trifluoromethanesulfonic acid (0.05 equiv.) and an amine (2 equiv.) in MeCN (0.1 M). After 30 min of stirring, trimethylsilyl cyanide (2.0 equiv.) was added and then the reaction was left to stir at room temperature until completion. Another equivalent of amine and trimethylsilyl cyanide was added if the reaction did not go to completion overnight. The reaction was monitored by TLC. The solvent was removed by *in vacuo* and then extracted with ethyl acetate (x3) and brine. The organic layer was dried over Na_2SO_4, filtered, concentrated *in vacuo* and purified by flash chromatography or CombiFlash using hexanes:acetone to afford pure product.

MIDA (2-((2-chloro-4-fluorobenzyl)amino)-2-cyanoethyl)boronate (4a)

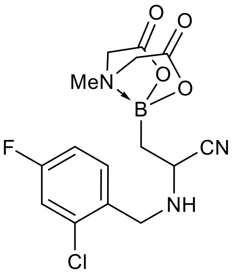

White solid; yield = 173 mg, 94 %; R_f = 0.49 (3:4, hex:ace). ^{1}H NMR (700 MHz, Acetonitrile-d_3) δ 7.52 – 7.47 (m, 1H), 7.23 (dd, J = 8.8, 2.6 Hz, 1H), 7.11 – 7.04 (m, 1H), 4.07 – 4.03 (m, 1H), 3.96 – 3.79 (m, 6H), 3.69 (dd, J = 8.4, 6.6 Hz, 1H), 2.90 (s, 3H), 1.30 (dd, J = 15.1, 6.6 Hz, 1H), 1.26 (dd, J = 15.1, 8.4 Hz, 1H). ^{13}C NMR (126 MHz, Acetonitrile-d_3) δ 169.1, 168.7, 162.3 (d, $^1J_{C\text{-}F}$ = 246.9 Hz), 134.8 (d, $^3J_{C\text{-}F}$

= 10.6 Hz), 133.6 (d, $^{4}J_{C\text{-}F}$ = 3.6 Hz), 132.5 (d, $^{3}J_{C\text{-}F}$ = 8.9 Hz), 122.4, 117.2 (d, $^{2}J_{C\text{-}F}$ = 25.0 Hz,), 114.8 (d, $^{2}J_{C\text{-}F}$ = 21.0 Hz), 62.9, 62.6, 49.0, 47.3, 46.9. ^{11}B NMR (128 MHz, Acetonitrile-d_3): δ 12.0. ^{19}F NMR (377 MHz, Acetonitrile-d_3) δ -114.5 (q, *J* = 8.5 Hz, 1F). HRMS (ESI, positive): *m/z* for $C_{15}H_{17}BClFN_3O_4$: calcd 367.1016, observed 367.1019 [M+H].

MIDA (2-((2-chlorophenyl)amino)-2-cyanoethyl)boronate (4b)

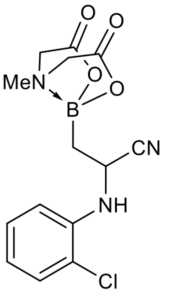

White solid; yield = 77 mg, 45 %; R_f = 0.53 (3:4, hex:ace). ^{1}H NMR (700 MHz, Acetonitrile-d_3) δ 7.35 (dd, *J* = 7.9, 1.5 Hz, 1H), 7.27 (tdd, *J* = 7.4, 1.5, 0.8 Hz, 1H), 6.93 (dd, *J* = 8.2, 1.4 Hz, 1H), 6.81 (ddd, *J* = 8.0, 7.4, 1.4 Hz, 1H), 5.00 (d, *J* = 8.4 Hz, 1H), 4.57 – 4.43 (m, 1H), 4.01 (d, *J* = 14.2 Hz, 1H), 3.98 (d, *J* = 14.1 Hz, 1H), 3.87 (d, *J* = 13.8 Hz, 1H), 3.84 (d, *J* = 13.7 Hz, 1H), 2.94 (s, 3H), 1.50 (d, *J* = 3.4 Hz, 1H), 1.49 (d, *J* = 4.5 Hz, 1H). ^{13}C NMR (126 MHz, Acetonitrile-d_3) δ 168.6, 168.5, 142.5, 130.3, 129.0, 121.6, 120.5, 120.4, 113.8, 62.9, 62.8, 46.9, 42.9. ^{11}B NMR (128 MHz, Acetonitrile-d_3): δ 11.7. HRMS (ESI, positive): *m/z* for $C_{14}H_{16}BClN_3O_4$: calcd 335.0953, observed 335.0953 [M+H].

MIDA (2-((2-chlorobenzyl)(methyl)amino)-2-cyanoethyl)boronate (4c)

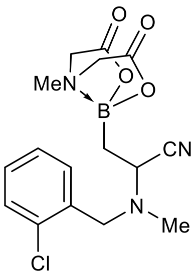

White solid; yield = 34 mg, 19 %; LC-MS retention time = 3.71 min (Method A). ^{1}H NMR (500 MHz, Acetonitrile-d_3) δ 7.59 – 7.50 (m, 1H), 7.40 (dd, *J* = 7.8, 1.5 Hz, 1H), 7.37 – 7.25 (m, 2H), 3.98 (d, *J* = 4.2 Hz, 1H), 3.95 (d, *J* = 4.2 Hz, 1H), 3.88 – 3.76 (m, 4H), 3.64 (d, *J* = 13.9 Hz, 1H), 2.87 (s, 3H), 2.25

(s, 3H), 1.32 (dd, J = 14.4, 9.4 Hz, 1H), 1.15 (dd, J = 14.4, 6.0 Hz, 1H). ^{13}C NMR (126 MHz, Acetonitrile-d_3) δ 168.8, 168.7, 136.4, 134.8, 132.0, 130.3, 129.7, 127.9, 118.9, 62.7, 62.7, 56.7, 54.4, 46.7, 37.8. ^{11}B NMR (128 MHz, Acetonitrile-d_3) δ 11.7. HRMS (ESI, positive): *m/z* for $C_{16}H_{20}BClN_3O_4$: calcd 363.1266, observed 363.1263 [M+H].

General preparation of acylated β,β-aminocyano(MIDA)boronates (4d-l)

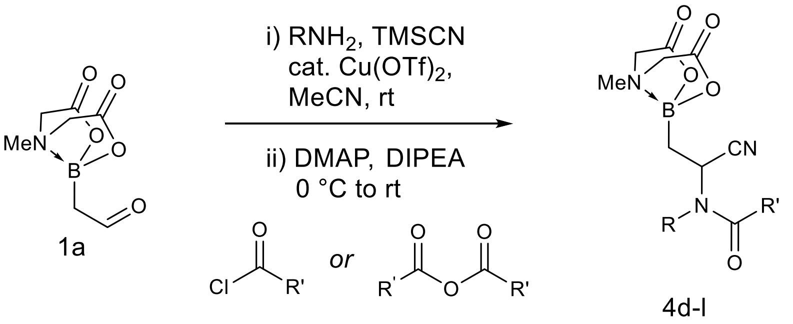

To a flame-dried, round bottom flask equipped with a magnetic stir bar under nitrogen atmosphere was added α-boryl aldehyde **1a** (1.0 equiv.), copper (II) trifluoromethanesulfonic acid (0.05 equiv.) and an amine (2 equiv.) in MeCN (0.1 M). After 30 min of stirring, trimethylsilyl cyanide (2.0 equiv.) was added and then the reaction was left to stir at room temperature until completion. Another equivalent of amine and trimethylsilyl cyanide was added if the reaction did not go to completion overnight. The reaction was monitored by TLC. Next, catalytic 4-dimethylaminopyridine and N,N-diisopropylethylamine (5.0 equiv.) was added and the reaction was cooled to 0 ºC. The corresponding acid chloride or anhydride (4.0 equiv.) was added to the solution dropwise or in 2 separate increments every 2 hours. The reaction was stirred at room temperature for 4 hours until completion. The solvent was removed by *in vacuo* and then extracted with ethyl acetate (x3) and brine. The organic layer was collected and then acidified to pH 1 using 0.1 M HCl. The aqueous layer was removed and then saturated $NaHCO_3$ was added to the organic layer until it reached pH 8-9. The organic layer was dried over Na_2SO_4, filtered, concentrated *in vacuo* and purified by flash chromatography or CombiFlash using

hexanes:acetone to afford pure product. Compounds **4g-4k** exhibited rotamers at room temperature but this was resolved by heating the molecules to 60 ºC.

MIDA (2-cyano-2-(*N*-(4-methylbenzyl)acetamido)ethyl)boronate (4d)

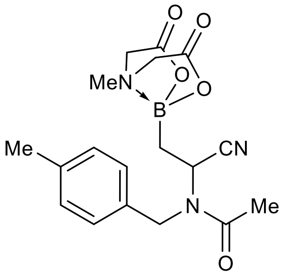

White solid; yield = 151 mg, 81 %; R_f = 0.18 (3:4, hex:ace). ^{1}H NMR (400 MHz, Acetonitrile-d_3) δ 7.21 (s, 4H), 5.01 (t, *J* = 8.0 Hz, 1H), 4.70 (d, *J* = 17.2 Hz, 1H), 4.52 (d, *J* = 17.3 Hz, 1H), 3.97 (d, *J* = 17.1 Hz, 2H), 3.83 (d, *J* = 4.9 Hz, 1H), 3.79 (d, *J* = 5.0 Hz, 1H), 2.85 (s, 3H), 2.33 (s, 3H), 1.34 (d, *J* = 8.0 Hz, 2H). ^{13}C NMR (126 MHz, Acetone-d_6): δ 171.7, 168.7, 168.6, 138.2, 134.9, 130.3, 127.7, 120.3, 62.8 (s, 2C, methylene carbons of MIDA ligand), 51.4, 46.8, 44.7, 22.4, 21.0. ^{11}B NMR (128 MHz, Acetone-d_6): δ 11.4. HRMS (ESI, positive): *m/z* for $C_{18}H_{23}BN_3O_5$: calcd 371.1762, observed 371.1753 [M+H].

MIDA (2-(*N*-(2-bromobenzyl)acetamido)-2-cyanoethyl)boronate (4e)

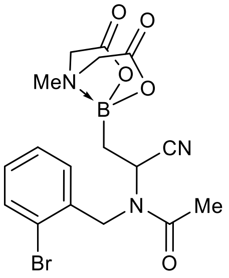

White solid; yield = 374 mg, 83 %; R_f = 0.23 (4:3, hex:ace). ^{1}H NMR (700 MHz, Acetonitrile-d_3) δ 7.65 (d, *J* = 7.9 Hz, 1H), 7.42 (t, *J* = 7.6 Hz, 1H), 7.35 (d, *J* = 7.7 Hz, 1H), 7.26 (t, *J* = 7.7 Hz, 1H), 5.10 (t, *J* = 8.0 Hz, 1H), 4.83 – 4.43 (m, 2H), 3.98 (dd, *J* = 17.1, 2.9 Hz, 2H), 3.82 (d, *J* = 17.1 Hz, 2H), 2.86 (s, 3H), 2.00 (s, 3H), 1.38 (dd, *J* = 14.2, 7.7 Hz, 1H), 1.34 (dd, *J* = 14.4, 8.4 Hz, 1H). ^{13}C NMR (126 MHz, Acetonitrile-d_3) δ 172.0, 168.6, 168.6, 136.6, 133.9, 130.3, 129.1, 128.9, 123.1, 120.1, 62.8, 62.8, 52.2,

46.9, 44.7, 22.2. ^{11}B NMR (128 MHz, Acetonitrile-d_3): δ 11.6. HRMS (ESI, positive): *m/z* for $C_{17}H_{20}BBrN_3O_5$: calcd 435.0710, observed 435.0701 [M+H].

MIDA (2-(*N*-(2-chlorobenzyl)acetamido)-2-cyanoethyl)boronate (4f)

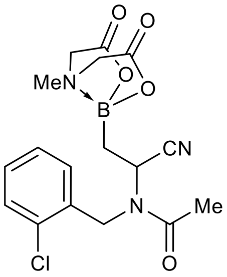

White solid; yield = 156 mg, 79 %; R_f = 0.49 (3:4, hex:ace). ^{1}H NMR (500 MHz, Acetonitrile-d_3) δ 7.50 – 7.21 (m, 4H), 5.09 (t, *J* = 8.0 Hz, 1H), 4.77 (d, *J* = 18.0 Hz, 1H), 4.69 (d, *J* = 18.0 Hz, 1H), 3.98 (dd, *J* = 17.1, 1.6 Hz, 2H), 3.82 (d, *J* = 17.1 Hz, 2H), 2.86 (s, 3H), 2.01 (s, 3H), 1.37 (td, *J* = 12.3, 10.7, 8.0 Hz, 2H). ^{13}C NMR (126 MHz, Acetonitrile-d_3) δ 171.9, 168.6, 168.6, 135.1, 133.3, 130.6, 130.1, 129.0, 128.3, 120.1, 62.8, 62.8, 49.7, 46.9, 44.7, 22.2. ^{11}B NMR (128 MHz, Acetonitrile-d_3): δ 11.4. HRMS (DART-TOF+): *m/z* for $C_{17}H_{20}BClN_3O_5$: calcd 392.1184, observed 392.1189 [M+H].

MIDA (2-cyano-2-(*N*-(4-methylbenzyl)benzamido)ethyl)boronate (4g)

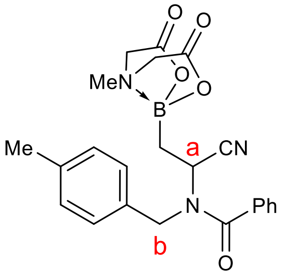

White solid; yield = 198 mg, 91 %; R_f = 0.20 (4:3, hex:ace). ^{1}H NMR (700 MHz, Acetonitrile-d_3) δ 7.57 – 7.52 (m, 2H), 7.50 – 7.40 (m, 3H), 7.18 (s, 4H), 4.80 (dd, *J* = 8.5, 7.3 Hz, 1H), 4.66 (d, *J* = 16.1 Hz, 1H), 4.57 (d, *J* = 16.2 Hz, 1H), 3.99 (d, *J* = 2.1 Hz, 1H), 3.97 (d, *J* = 2.1 Hz, 1H), 3.84 (d, *J* = 6.9 Hz, 1H), 3.82 (d, *J* = 6.9 Hz, 1H), 2.88 (s, 3H), 2.32 (s, 3H), 1.60 (dd, *J* = 14.4, 8.4 Hz, 1H), 1.42 (dd, *J* = 14.3, 7.2 Hz, 1H). ^{13}C NMR (126 MHz, Acetonitrile-d_3) δ 172.2, 168.8, 168.5, 138.4, 136.8, 134.4,

130.8, 130.1, 129.4, 128.4, 127.7, 120.1, 62.8, 62.7, 46.8, 21.0. ^{11}B NMR (128 MHz, Acetonitrile-d_3): δ 11.5. HRMS (ESI, positive): *m/z* for $C_{23}H_{25}BN_3O_5$: calcd 433.1918, observed 433.1907 [M+H].

*Both sp^3 carbons next to the nitrogen were not observed in the ^{13}C NMR. This case is similar to compounds **4h-4k**.

MIDA (2-(*N*-(2-chlorobenzyl)benzamido)-2-cyanoethyl)boronate (4h)

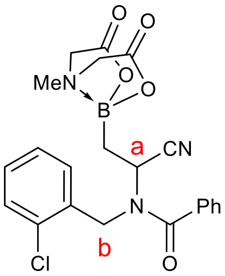

White solid; yield = 212 mg, 92 %; R_f = 0.47 (3:4, hex:ace) ^{1}H NMR (400 MHz, Acetonitrile-d_3) δ 7.60 – 7.53 (m, 2H), 7.53 – 7.26 (m, 7H), 4.76 (m, 3H), 4.00 (dd, J = 17.1, 1.4 Hz, 2H), 3.84 (dd, J = 17.1, 4.1 Hz, 2H), 2.87 (s, 3H), 1.67 (s, 1H), 1.37 (dd, J = 14.2, 6.7 Hz, 1H). ^{13}C NMR (126 MHz, Acetonitrile-d_3) δ 172.4, 168.8, 4j168.5, 136.5, 134.7, 134.0, 131.0, 130.6, 130.3, 129.3, 128.2, 127.9 (s, 2C), 119.9, 62.8, 62.7, 46.9. ^{11}B NMR (128 MHz, Acetonitrile-d_3): δ 11.5. HRMS (ESI, positive): *m/z* for $C_{22}H_{22}BClN_3O_5$: calcd 453.1372, observed 453.1358 [M+H].

*Both sp^3 carbons next to the nitrogen were not observed in the ^{13}C NMR. A cosy at 60 ºC showed the protons of **a** coupling to the CH_2. An HMBC at 60 ºC showed the protons of **b** coupling to the aromatic carbons.

MIDA (2-(*N*-(2-chloro-5-(trifluoromethyl)benzyl)benzamido)-2-cyanoethyl)boronate (4i)

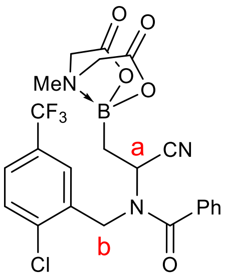

White solid; yield = 828 mg, 80 %; R_f = 0.55 (3:4, hex:ace). ^{1}H NMR (700 MHz, Acetonitrile-d_3) δ 7.76 (dt, *J* = 2.2, 0.7 Hz, 1H), 7.61 (ddt, *J* = 8.3, 2.3, 0.7 Hz, 1H), 7.57 (d, *J* = 8.4 Hz, 1H), 7.55 – 7.51 (m, 2H), 7.50 – 7.45 (m, 1H), 7.45 – 7.41 (m, 2H), 4.96 (dd, *J* = 8.7, 7.1 Hz, 1H), 4.91 (d, *J* = 17.0 Hz, 1H), 4.80 (d, *J* = 16.9 Hz, 1H), 3.99 (d, *J* = 17.0 Hz, 2H), 3.85 (d, *J* = 10.9 Hz, 1H), 3.83 (d, *J* = 10.9 Hz, 1H), 2.90 (s, 3H), 1.63 (t, *J* = 10.9 Hz, 1H), 1.42 (dd, *J* = 14.3, 7.1 Hz, 1H). ^{13}C NMR (126 MHz, Acetonitrile-d_3) δ 172.9, 168.7, 168.4, 131.8, 131.4, 130.3 (q, $^{2}J_{C\text{-}F}$ = 32.9 Hz), 129.8, 128.0, 127.4 (q, $^{3}J_{C\text{-}F}$ = 3.9 Hz), 127.2 (q, $^{3}J_{C\text{-}F}$ = 3.7 Hz), 125.2 (q, $^{1}J_{C\text{-}F}$ = 271.6 Hz), 120.0, 63.3, 63.2, 47.2. ^{11}B NMR (128 MHz, Acetonitrile-d_3): δ 11.3. HRMS (ESI, positive): *m/z* for $C_{23}H_{21}BClF_3N_3O_5$: calcd 521.1246, observed 521.1237 [M+H].

*Both sp^{3} carbons next to the nitrogen were not observed in the ^{13}C NMR. A cosy at 60 ºC showed the protons of **a** coupling to the CH_2. An HMBC at 60 ºC showed the protons of **b** coupling to the aromatic carbons.

MIDA (2-(*N*-(2-chloro-4-fluorobenzyl)benzamido)-2-cyanoethyl)boronate (4j)

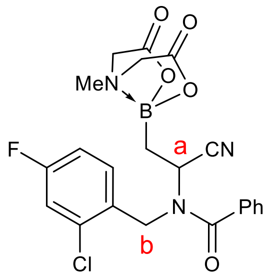

White solid; yield = 1073 mg, 91 %; R_f = 0.23 (4:3, hex:ace). ^{1}H NMR (700 MHz, Acetonitrile-d_3) δ 7.57 – 7.54 (m, 2H), 7.51 (dd, *J* = 8.7, 6.0 Hz, 1H), 7.50 – 7.46 (m, 1H), 7.46 – 7.41 (m, 2H), 7.21 (dd, *J* = 8.7, 2.6 Hz, 1H), 7.13 (td, *J* = 8.5, 2.6 Hz, 1H), 4.88 (dd, *J* = 8.5, 7.2 Hz, 1H), 4.81 (d, *J* = 16.6 Hz, 1H), 4.72 (d, *J* = 16.5 Hz, 1H), 3.99 (dd, *J* = 17.1, 0.9 Hz, 2H), 3.85 (d, *J* = 4.3 Hz, 1H), 3.82 (d, *J* = 4.3 Hz, 1H), 2.89 (s, 3H), 1.61 (dd, *J* = 14.2, 8.6 Hz, 1H), 1.41 (dd, *J* = 14.3, 7.2 Hz, 1H). ^{13}C NMR (126 MHz, DMSO-d_6) δ 170.6, 168.1, 167.8, 161.1 (d, $^{1}J_{C\text{-}F}$ = 247.6 Hz), 135.0, 133.0 (d, $^{3}J_{C\text{-}F}$ = 10.6 Hz), 130.6 (d, $^{3}J_{C\text{-}F}$ = 9.4 Hz), 129.8 (d, $^{4}J_{C\text{-}F}$ = 3.3 Hz), 129.7, 128.1, 126.4, 118.5, 116.5 (d, $^{2}J_{C\text{-}F}$ = 25.1 Hz),

114.1 (d, $^2J_{C\text{-}F}$ = 21.2 Hz), 61.6, 61.5, 45.6. ^{11}B NMR (160 MHz, Acetone-d_6) δ 11.5. ^{19}F NMR (377 MHz, Acetone-d_6) δ -114.2. HRMS (DART-TOF+): *m/z* for $C_{22}H_{21}BClFN_3O_5$: calcd 472.1246, observed 472.1257 [M+H].

*Both sp^3 carbons next to the nitrogen were not observed in the ^{13}C NMR. A cosy at 60 ºC showed the protons of **a** coupling to the CH_2. An HMBC at 60 ºC showed the protons of **b** coupling to the aromatic carbons.

MIDA (2-(*N*-(2-chlorophenethyl)benzamido)-2-cyanoethyl)boronate (4k)

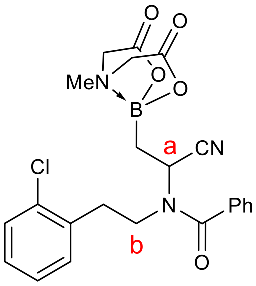

White solid; yield = 224 mg, 96 %; R_f = 0.20 (4:3, hex:ace). ^{1}H NMR (700 MHz, Acetonitrile-d_3) δ 7.49 – 7.45 (m, 1H), 7.44 – 7.40 (m, 2H), 7.38 (dt, *J* = 7.0, 1.4 Hz, 2H), 7.31 – 7.28 (m, 1H), 7.25 – 7.17 (m, 3H), 5.06 (d, *J* = 8.2 Hz, 1H), 3.99 (d, *J* = 8.0 Hz, 1H), 3.97 (d, *J* = 8.1 Hz, 1H), 3.86 (d, *J* = 17.5 Hz, 1H), 3.83 (d, *J* = 17.3Hz, 1H), 3.68 (ddd, *J* = 14.9, 9.3, 6.4 Hz, 1H), 3.66 – 3.61 (m, 1H), 3.13 – 3.01 (m, 2H), 2.92 (s, 3H), 1.56 (s, 1H), 1.49 (dd, *J* = 14.2, 7.4 Hz, 1H). ^{13}C NMR (126 MHz, Acetonitrile-d_3) δ 172.2, 168.8, 168.6, 136.8, 136.6, 134.6, 132.3, 130.5, 130.4, 129.4, 129.4, 128.2, 127.5, 120.8, 62.8, 62.7, 46.9, 33.7. ^{11}B NMR (128 MHz, Acetonitrile-d_3) δ 11.6. HRMS (ESI, positive): *m/z* for $C_{23}H_{24}BClN_3O_5$: calcd 467.1528, observed 467.1526 [M+H].

*Both sp^3 carbons next to the nitrogen were not observed in the ^{13}C NMR. A cosy at 60 ºC showed the protons of **a** coupling to the CH_2. An HMBC at 60 ºC showed the protons of **b** coupling to the aromatic carbons.

MIDA (2-(*N*-(2-bromobenzyl)-2,2,2-trifluoroacetamido)-2-cyanoethyl)boronate (4l)

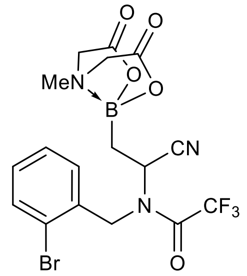

White solid; yield = 207 mg, 81 %; R_f = 0.34 (4:3, hex:ace). ^{1}H NMR (400 MHz, Acetonitrile-d_3) δ 7.67 (dt, J = 8.1, 2.4 Hz, 1H), 7.43 (dt, J = 16.7, 7.3 Hz, 2H), 7.29 (tdd, J = 13.5, 8.9, 4.9 Hz, 1H), 4.92 (d, J = 17.5 Hz, 1H), 4.81 (d, J = 17.6 Hz, 1H), 4.58 (t, J = 8.0 Hz, 1H), 4.02 (d, J = 5.3 Hz, 1H), 3.97 (d, J = 5.3 Hz, 1H), 3.85 (d, J = 4.2 Hz, 1H), 3.81 (d, J = 4.2 Hz, 1H), 2.86 (s, 3H), 1.62 (dd, J = 14.1, 8.7 Hz, 1H), 1.39 (ddd, J = 15.6, 10.0, 7.3 Hz, 1H). ^{13}C NMR (126 MHz, Acetone-d_6) δ 168.3, 168.1, 157.6 (q, $^{2}J_{C\text{-}F}$ = 37.1 Hz), 135.0, 134.1, 130.8, 129.3, 129.0, 123.0, 118.3, 116.8 (q, $^{1}J_{C\text{-}F}$ = 287.6 Hz), 62.8, 62.7, 53.1, 48.3, 46.7. ^{11}B NMR (128 MHz, Acetonitrile-d_3): δ 11.3. ^{19}F NMR (377 MHz, Acetonitrile-d_3) δ -70.2 (s, 3F). HRMS (DART-TOF+): *m/z* for $C_{17}H_{17}BBrF_3N_3O_5$: calcd 490.0396, observed 490.0390 [M+H].

Synthesis of parent acylboronate (5)

Parent acylboronate **5** was prepared according to literature methods.[4]

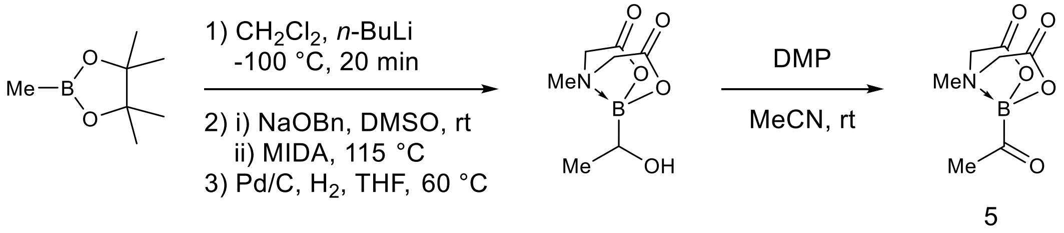

Synthesis of α,α-aminocyano(MIDA)boronate (7)

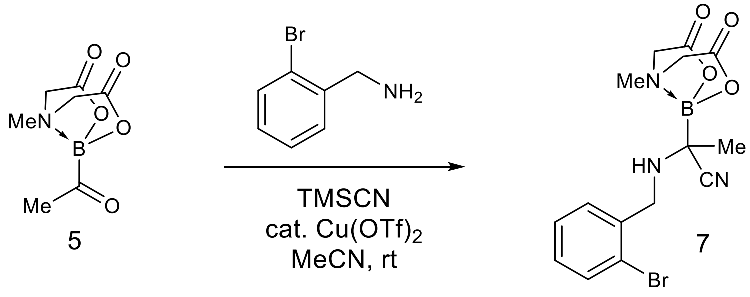

To a flame-dried, round bottom flask equipped with a magnetic stir bar under nitrogen atmosphere was added acylboronate **5** (1.0 equiv.), copper (II) trifluoromethanesulfonic acid (0.05 equiv.) and an amine (2 equiv.) in MeCN (0.1 M). After 30 min of stirring, trimethylsilyl cyanide (2.0 equiv.) was added and then the reaction was left to stir at room temperature until completion. Another equivalent of amine and trimethylsilyl cyanide was added if the reaction did not go to completion overnight. The reaction was monitored by TLC. The solvent was removed by *in vacuo* and then extracted with ethyl acetate (x3) and brine. The organic layer was dried over Na_2SO_4, filtered, concentrated *in vacuo* and purified by flash chromatography or CombiFlash using hexanes:acetone to afford pure product.

MIDA (1-((2-bromobenzyl)amino)-1-cyanoethyl)boronate (7a)

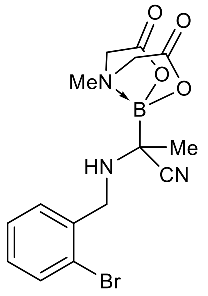

White solid; yield = 18 mg, 91 %; R_f = 0.48 (4:3, hex:ace). ^{1}H NMR (500 MHz, Acetonitrile-d_3) δ 7.57 (dd, *J* = 8.0, 1.2 Hz, 1H), 7.47 (ddq, *J* = 7.6, 1.6, 0.5 Hz, 1H), 7.33 (td, *J* = 7.5, 1.3 Hz, 1H), 7.21 – 7.15 (m, 1H), 4.13 – 3.85 (m, 7H), 3.28 (s, 3H), 1.58 (s, 3H). ^{13}C NMR (126 MHz, Acetonitrile-d_3) δ 168.3, 168.0, 139.9, 133.5, 131.9, 129.9, 128.6, 124.7, 123.9, 64.3, 64.1, 49.3, 48.3, 20.6. ^{11}B NMR (128 MHz, Acetonitrile-d_3): δ 9.8. HRMS (ESI, positive): *m/z* for $C_{15}H_{18}BBrN_3O_4$: calcd 393.0605, observed 393.0606 [M+H].

General preparation of α-functionalized alkyl(MIDA)boronates (8-10)

α-Functionalized alkyl(MIDA)boronates **8-10** were prepared according to literature methods.[5] List of known α-functionalized alkyl(MIDA)boronates previously characterized:

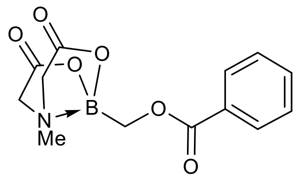

8a

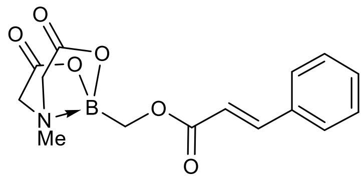

8b

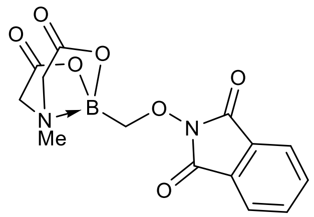

8c

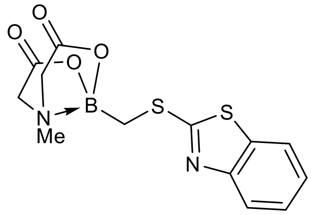

9a

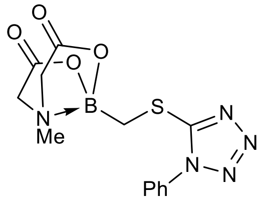

9b

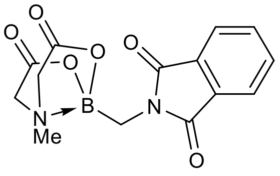

9c

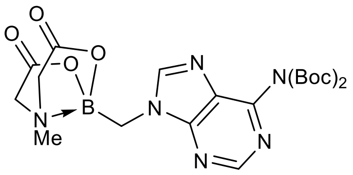

10a

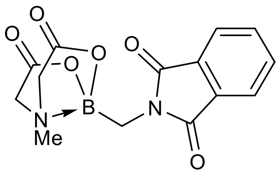

10b

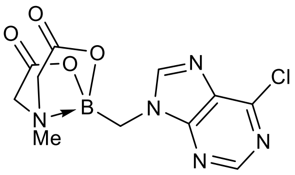

10c

Preparation of acylated β-amino(MIDA)boronate (11a)

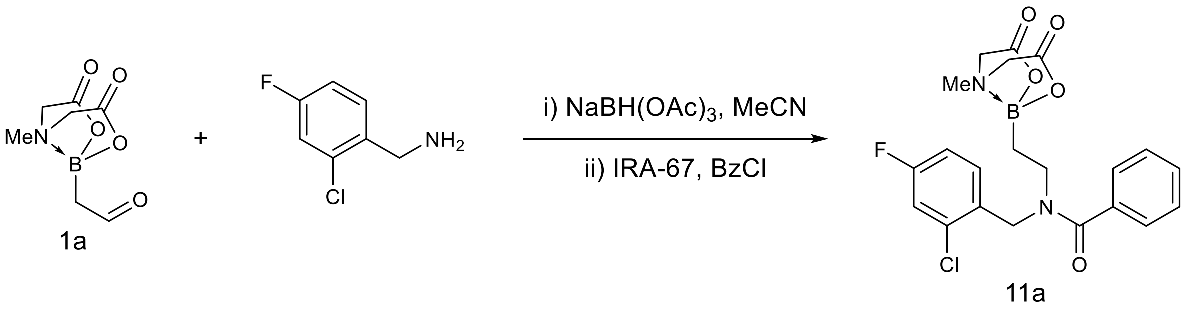

An oven-dried 50 mL round bottom flask equipped with a magnetic stir bar was charged with α-boryl acetaldehyde **1a** (80 mg, 1.0 equiv., 0.40 mmol) and (2-chloro-4-fluorophenyl)methanamine (60.6 μL, 1.2 equiv., 0.48 mmol) in acetonitrile (8 mL, 0.05 M). The reaction was allowed to stir under a nitrogen atmosphere for 1 hour at room temperature. Sodium tri(acetoxy)borohydride (128 mg, 1.5 equiv., 0.60 mmol) was added and the reaction was vigorously stirred for 6 hours. Amberlite IRA-67 resin (1g) was then added to the reaction mixture and stirred for 10 minutes before the dropwise addition of benzoyl chloride (93.4 μL, 2.0 equiv., 0.81 mmol) to the reaction mixture. After 4 hours, Amberlite IRA-743 was

added to the crude mixture for the removal of borate/boronic acid by-products from $NaBH(OAc)_3$.The solution was then stirred for approximately 5 minutes (or until the solution turned clear) and then filtered immediately through a plug of Celite while rinsing with acetonitrile. The filtrate was concentrated and adsorbed onto Celite *in vacuo* from an ethyl acetate solution. The resulting powder was subjected to reverse-phase flash-chromatography (water:acetonitrile 95:5 → 0:100, with 1% formic acid). The fractions containing product (determined by LC-MS) were pooled and lyophilized to afford **11a** (40 mg, 22.3 mmol) as a white powder. Compound **11a** exhibited rotamers at room temperature but this was resolved by heating the molecules to 60 ºC.

MIDA (2-(*N*-(2-chloro-4-fluorobenzyl)benzamido)ethyl)boronate (11a)

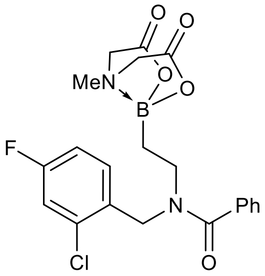

White solid; yield = 40 mg, 22 %; R_f = 0.18 (4:3, hex:ace). ^{1}H NMR (400 MHz, Acetonitrile-d_3) δ 7.45 (s, 6H), 7.25 (dd, J = 8.7, 2.6 Hz, 1H), 7.14 (td, J = 8.5, 2.7 Hz, 1H), 3.90 (d, J = 17.0 Hz, 2H), 3.75 (d, J = 16.9 Hz, 2H), 3.41 (s, 2H), 2.80 (s, 3H), 0.97 (d, J = 26.8 Hz, 2H). ^{13}C NMR (126 MHz, DMSO-d_6) δ 170.4, 168.1 (s, 2C, carbonyls of MIDA ligand), 160.8 (d, $^{1}J_{C\text{-}F}$ = 247.2 Hz), 136.4, 132.7 (d, $^{3}J_{C\text{-}F}$ = 10.3 Hz), 130.9, 129.9 (d, $^{3}J_{C\text{-}F}$ = 9.1 Hz), 128.9, 128.1, 126.1, 116.4 (d, $^{2}J_{C\text{-}F}$ = 25.1 Hz), 114.2 (d, $^{2}J_{C\text{-}F}$ = 21.1 Hz), 61.4 (s, 2C, methylene carbons of MIDA ligand), 45.3. ^{11}B NMR (128 MHz, Acetonitrile-d_6): δ 11.9. ^{19}F NMR (377 MHz, Acetonitrile-d_3) δ -114.9, -115.3 (major rotamer). HRMS (ESI, positive): *m/z* for $C_{21}H_{22}BClN_2O_5$: calcd 446.1325, observed 446.1320 [M+H].

Preparation of β, β-aminocyanobenzyamide (11b)

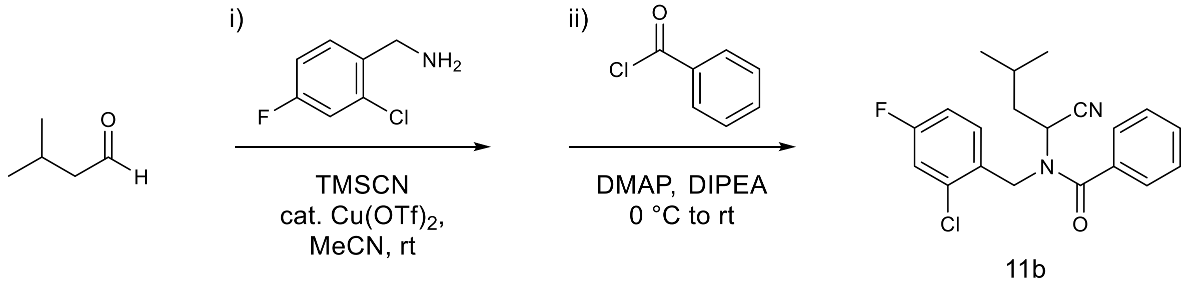

11b

To a flame-dried, round bottom flask equipped with a magnetic stir bar under nitrogen atmosphere was added isovaleraldehyde (1.0 equiv.), copper (II) trifluoromethanesulfonic acid (0.05 equiv.) and an amine (2 equiv.) in MeCN (0.1 M). After 30 min of stirring, trimethylsilyl cyanide (2.0 equiv.) was added and then the reaction was left to stir at room temperature until completion. Another equivalent of amine and trimethylsilyl cyanide was added if the reaction did not go to completion overnight. The reaction was monitored by TLC. Next, catalytic 4-dimethylaminopyridine and N,N-diisopropylethylamine (5.0 equiv.) was added and the reaction was cooled to 0 ºC. The corresponding acid chloride or anhydride (4.0 equiv.) was added to the solution dropwise or in 2 separate increments every 2 hours. The reaction was stirred at room temperature for 4 hours until completion. The solvent was removed by *in vacuo* and then extracted with ethyl acetate (x3) and brine. The organic layer was collected and then acidified to pH 1 using 0.1 M HCl. The aqueous layer was removed and then saturated $NaHCO_3$ was added to the organic layer until it reached pH 8-9. The organic layer was dried over Na_2SO_4, filtered, concentrated *in vacuo* and purified by flash chromatography or CombiFlash using hexanes:acetone to afford pure product. Compound **11b** exhibited rotamers at room temperature but this was resolved by heating the molecules to 50-55 ºC.

***N*-(2-chloro-4-fluorobenzyl)-*N*-(1-cyano-3-methylbutyl)benzamide (11b)**

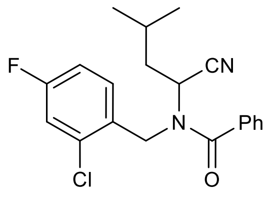

White solid; yield = 18 mg, 91 %; R_f = 0.71 (3:1, hex:EtOAc). ^{1}H NMR (700 MHz, Acetonitrile-d_3) δ 7.56 – 7.38 (m, 6H), 7.22 (dd, J = 8.7, 2.6 Hz, 1H), 7.13 (tdd, J = 8.7, 2.6, 0.4 Hz, 1H), 4.90 (dd, J = 8.7, 6.8 Hz, 1H), 4.80 (d, J = 16.6 Hz, 1H), 4.72 (dt, J = 16.6, 0.9 Hz, 1H), 1.94 – 1.90 (m, 1H), 1.76 (ddd, J = 13.5, 7.5, 6.8 Hz, 1H), 1.70 (dp, J = 13.4, 6.6 Hz, 1H), 0.91 (d, J = 6.6 Hz, 3H), 0.86 (d, J = 6.6 Hz, 3H). ^{13}C NMR (126 MHz, Acetonitrile-d_3) δ 173.1, 163.3 (d, $^{1}J_{C-F}$ = 248.2 Hz), 136.5, 135.1 (d, $^{3}J_{C-F}$ = 10.5 Hz), 132.2 (d, $^{3}J_{C-F}$ = 9.1 Hz), 131.6, 131.6, 130.0, 128.1, 118.9, 118.1 (d, $^{2}J_{C-F}$ = 25.3 Hz), 115.6 (d, $^{2}J_{C-F}$ = 21.4 Hz), 48.9, 48.5, 41.2, 26.2, 22.8, 22.2. ^{19}F NMR (377 MHz, Acetonitrile-d_3) δ -113.93 (s, 3F). HRMS (DART-TOF+): *m/z* for $C_{20}H_{21}ClFN_2O$: calcd 359.1326, observed 359.1321 [M+H].

Synthesis of β, β-aminocyanoboronic acid (11c)

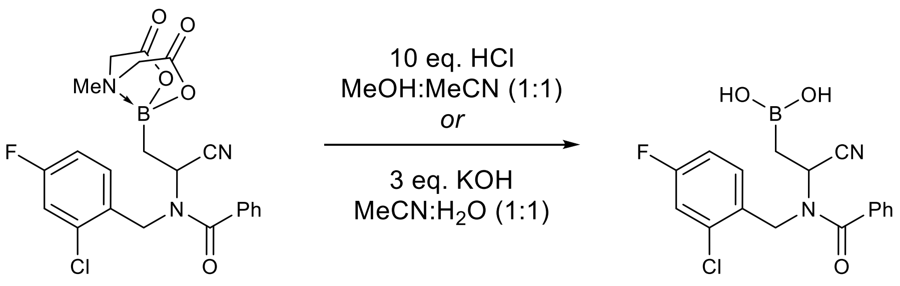

4j 11c

Acidic MIDA Deprotection

To an oven dried, scintillation vial equipped with a magnetic stir bar under nitrogen atmosphere was added MIDA boronate **4j**, hydrochloric acid (0.3 M, 10 equiv.) in MeOH:MeCN (1:1, v/v). The reaction was left to stir at room temperature overnight. The reaction was monitored by TLC and LC-MS. The solvent was removed under a stream of nitrogen. The crude mixture was purified using either of the following procedures: A) The crude mixture was triturated in MeCN and filtered through celite. The solvent was removed *in vacuo* to afford pure product. B) The crude mixture was re-dissolved in MeCN and loaded onto Celite. The reaction was purified via a short silica plug (flushed with 100% MeCN and eluted with 100% Ace) or reverse-phase HPLC (Method A) to afford pure product.

Basic MIDA Deprotection

To an oven dried, scintillation vial equipped with a magnetic stir bar under nitrogen atmosphere was added MIDA boronate **4j**, potassium hydroxide (3 equiv.) in MeCN:H_2O (1:1, v/v). The reaction was left to stir at room temperature until completion. The reaction was monitored by TLC and LC-MS. A) The crude mixture was triturated in MeCN and filtered through celite. The solvent was removed *in vacuo* to afford pure product. B) The crude mixture was re-dissolved in MeCN and loaded onto Celite. The reaction was purified via a short silica plug (flushed with 100% MeCN and eluted with 100% Ace) or reverse-phase HPLC (Method A) to afford pure product. Compound **11c** exhibited rotamers at room temperature but this was resolved by heating the molecules to 50-55 ºC. There are multiple peaks present in the ^{19}F spectrum due to the presence of rotamers and an equilibrium between multiple boron species.

(2-(*N*-(2-chloro-4-fluorobenzyl)benzamido)-2-cyanoethyl)boronic acid (11c)

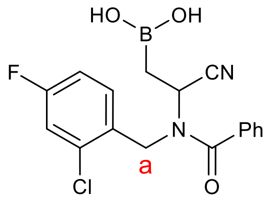

White solid; yield = 38 mg, quantitative; LC-MS retention time = 7.83 min (Method A. ^{1}H NMR (500 MHz, C<u>D</u>$_3$OD, ref: δ 3.31) δ 7.53 – 7.45 (m, 6H), 7.20 (ddd, J = 10.6, 8.5, 2.6 Hz, 1H), 7.15 – 7.09 (m, 1H), 4.98 (t, J = 7.7 Hz, 1H), 4.81 (s, 2H), 1.61 (d, J = 7.7 Hz, 2H). ^{13}C NMR (126 MHz, Methanol-d_4) δ 174.3, 163.6 (d, $^{1}J_{C\text{-}F}$ = 249.3 Hz), 136.4, 135.2 (d, $^{3}J_{C\text{-}F}$ = 9.7 Hz), 132.1 (d, $^{3}J_{C\text{-}F}$ = 8.9 Hz), 131.7, 131.4 (d, $^{4}J_{C\text{-}F}$ = 3.7 Hz), 129.8, 127.9, 119.7, 118.0 (d, $^{2}J_{C\text{-}F}$ = 25.4 Hz), 115.4 (d, $^{2}J_{C\text{-}F}$ = 21.6 Hz), 47.3. ^{11}B NMR (128 MHz, Acetonitrile-d_3) δ 29.5. ^{19}F NMR (377 MHz, Methanol-d_4) δ -113.8, -114.1, -115.5, -115.8, -117.0. LRMS (ESI, positive): *m/z* for $C_{18}H_{23}BN_3O_5$: observed 361.0 [M+H]**.**

*****The ^{13}C of the benzyl carbon **a** can be seen in the HSQC at 55 ºC overlapping with the solvent peak.

Activity-Based Protein Profiling Experiments

Preparation of Tissue and Cell Lysates

Cell lysates were prepared by lysing cell pellets in cold PBS using a probe sonicator. Mouse brains were homogenized by bead beating with a Bullet Blender (Nextadvance) according to the manufacturer specifications. Samples were fractionated by ultracentrifugation (100,000 g's, 45 min, 4 °C), flash frozen, and stored at -80 °C prior to use. The harvesting of mouse tissues was performed with the approval of the Institutional Animal Care and Use Committee at The Scripps Research Institute in accordance with the Guide for the Care and Use of Laboratory Animals.

Transient Overexpression of Proteins

HEK 293T cells were seeded at 3 x 10^5 per well on 6 well plates and grown for 24 hours. They were then transfected with mABHD3 (pCMV-Sport6), hABHD3 (pCMV6-XL4) or control vector, by incubating 0.5 µg of plasmid DNA with 3 µL of PEI MAX (1 mg/mL, Polysciences, Inc.) in serum-free DMEM for 30 min, and then adding this mixture to cells. After another 48 hours cells were either treated with compound *in situ*, as described below, or harvested for *in vitro* studies.

***In Vitro* Inhibitor and Probe Treatment of Tissue and Cell Lysates**

Proteome lysates (50 µL, 1 mg/mL), prepared as described above, were treated with either 1 µL of DMSO or inhibitor (50x final concentration, in DMSO). Following a 30 minute incubation at 37 °C, lysates were chased with FP-Rh (1 µM, 30 min) at room temperature. Samples were then quenched by adding 4x SDS-PAGE loading buffer (17 µl), resolved by SDS-PAGE, and visualized using a ChemiDoc MP (Bio-Rad). Signal intensity was quantified with ImageJ 1.45s, and IC50 values were determined with GraphPad Prism 7.

***In Situ* Inhibitor Treatment of Human Cell Lines**

Human SW620 and HEK 293T cells were grown in SILAC-RPMI or DMEM media, respectively, supplemented with 10% FBS. SILAC SW620s were split 72 hours prior to inhibitor treatment. The media was replaced with fresh media containing either DMSO or inhibitor, and the cells were incubated for 2 hours at 37 °C. Next, cells were washed with cold PBS, harvested with a cell scraper, centrifuged, and aspirated to remove PBS. Cell pellets were flash frozen and stored at -80 prior to lysis and probe treatment. HEK 293Ts were treated similarly but also transfected after 24 hours, as described above.

ABPP-SILAC Sample Preparation and Data Analysis

ABPP-SILAC samples were prepared and analyzed as described previously (Cognetta et al., 2015). Briefly, isotopically heavy and light SW620 cell lysates, prepared from *in situ* treated cells as described above, were incubated with FP-biotin (6 µM, 1 h) at room temperature. Light and heavy cell lysates were combined into a solution of MeOH/CHCl3 (4:1, 2.5 ml), additional cold PBS was added (1 mL), and the solution was centrifuged for 20 minutes (5,000 g, 4 °C) resulting in the precipitation of the protein fraction. The organic and aqueous layers were removed by aspiration, and the resulting pellets were then washed with 1:1 MeOH/$CHCl_3$ (1 mL, 2x), resuspended in 0.5 mL of 6 M urea in PBS, reduced using tris(2-carboxyethyl)phosphine (10 mM) for 30 min at 37 °C, and alkylated with iodoacetamide (40 mM) for 30 min at 25 °C in the dark. Biotinylated proteins were enriched with PBS-washed streptavidin beads (100 µL, Sigma-Aldrich) by rotating at room temperature for 1.5 hr in PBS (5.5 ml) with 0.2% SDS. The beads were washed sequentially with 10 mL of PBS with 0.2% SDS (3x), 10 mL of PBS (3x), and 10 mL of DI H_2O (3x). On-bead digestion was performed using sequencing-grade trypsin (2 µg; Promega) in 2 M urea in PBS with 2 mM $CaCl_2$ for 12–14 h at 37 °C (200 µl). Peptides obtained from this procedure were acidified using formic acid (5% final) and stored at -20 °C before analysis. Digested peptides were loaded on to a biphasic (strong cation exchange/reverse phase) capillary column and

analyzed by 2D LC-MS/MS on an LTQ-Orbitrap (Thermo Scientific). Samples were then searched using the ProLuCID algorithm against a human reverse-concatenated non-redundant (gene-centric) FASTA database that was assembled from the Uniprot database. SILAC ratios were quantified using in-house CIMAGE software[6].

Hydrolytic Stability Study of Compound 4j

Compound **4j** (4.0 mg, 8.5 μmol) was added to a ½ dram vial equipped with a magnetic stir and dissolved in acetonitrile (100 μL). A 0.1 M sodium phosphate solution in H_2O buffered to pH of 7.4 (300 μL) was then slowly added. The resulting solution was briefly sonicated and the reaction vial was then placed in an oil bath heated to 37 ºC. A 30 μL aliquot was taken out and added to a separate vial with 800 μL of water:acetonitrile for LC-MS analysis (time = 0 h). This process was repeated over 24 hours. See Supplementary Figure 4.

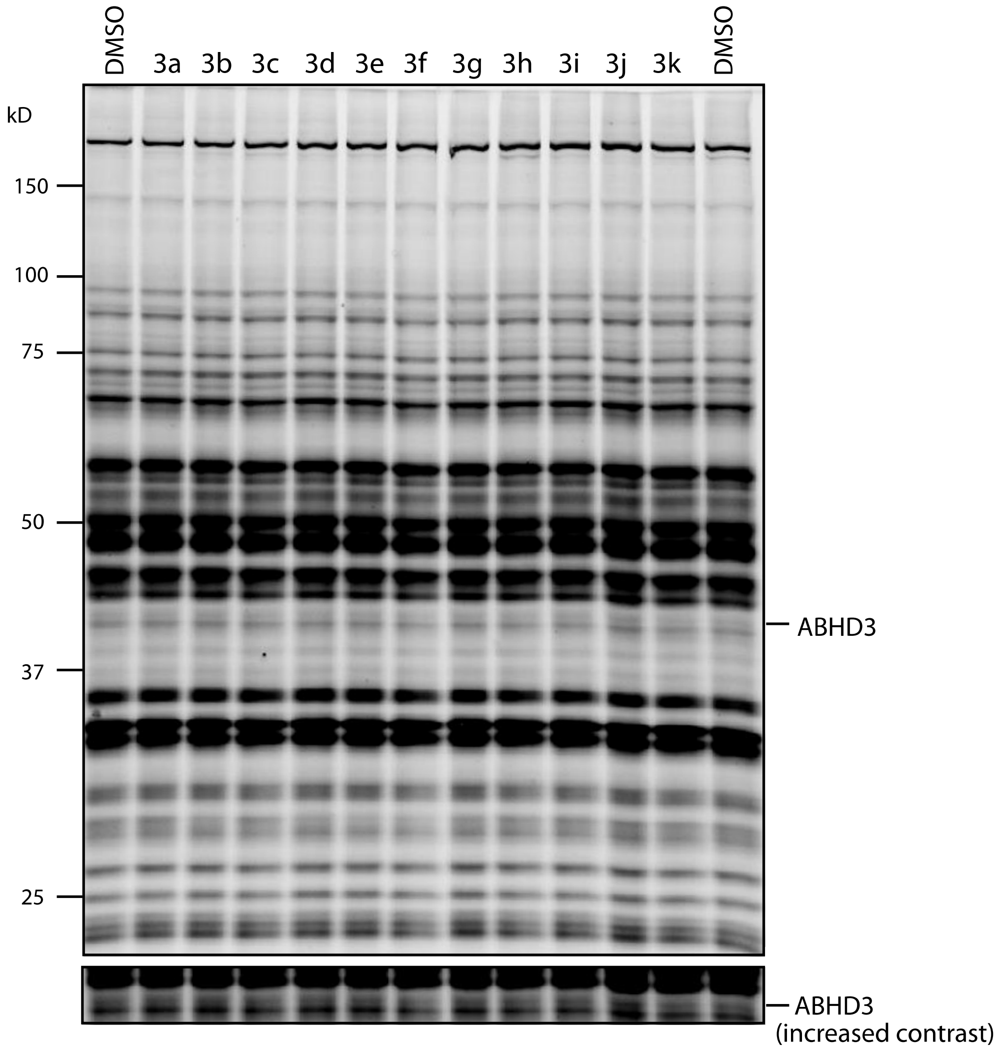

Supplementary Figure 1. ABPP gel of the membrane fraction of mouse brain treated with compounds **3a-3k** (20 µM, 30 min, 37 °C) followed by the broad-spectrum, serine hydrolase-directed activity-based probe FP-rhodamine (1 µM, 30 min, RT).

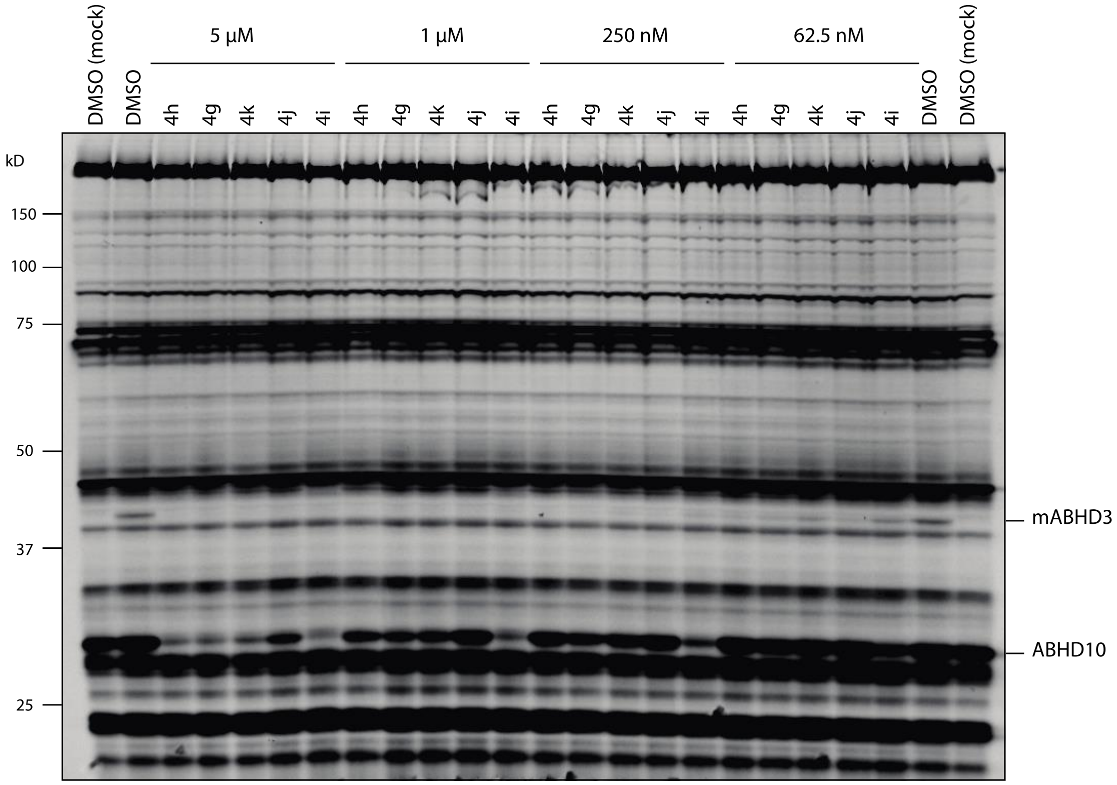

Supplementary Figure 2. ABPP gel showing concentration-dependent activity of representative compounds from MIDA Boronate library against cell lysates from mABHD3-overexpressing HEK 293Ts. Note that compounds **4j, 4h,** and **4g** show equivalent inhibitory activity against ABHD3, but **4j** is more selective against ABHD10.

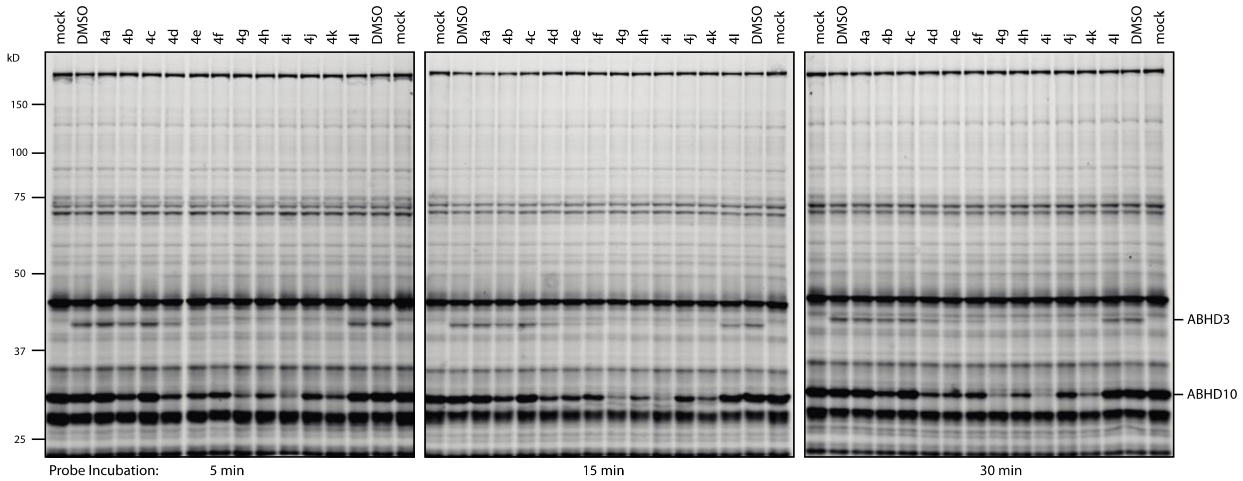

Supplementary Figure 3. ABPP gels testing the time-dependence of probe incubation on inhibition profiles of MIDA-boronates. Cell lysates from ABHD3-transfected HEK 293Ts (membrane fraction) were incubated with compounds 4a-4l (20 µM, 30 min, 37 °C) and chased with FP-Rh for 5, 15, or 30 minutes, after which samples were analyzed by gel-based ABPP. Note: 5 min and 15 min gel images are shown with increased contrast to match the intensity of the 30 min gel image.

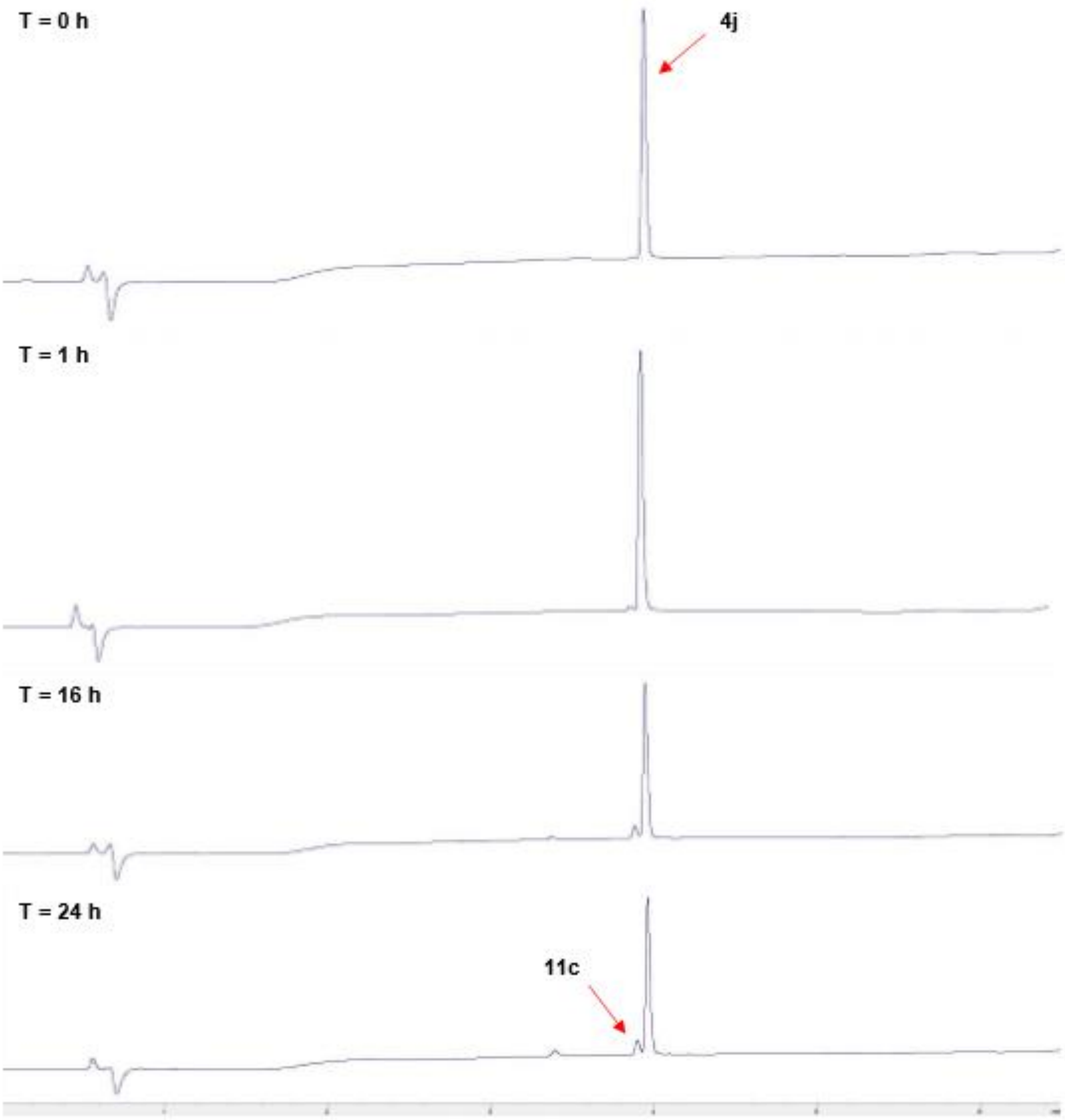

Supplementary Figure 4. Incubation of **4j** (20 mM) in acetonitrile:phosphate buffer (3:1) at pH 7.4 in a reaction volume of 400 μL at 37 ºC for 24 hours. Using liquid chromatography-mass spectrometry (LC-MS) analysis, less than 10% of boronic acid **11c** was released after 24 h. UV-Vis chromatogram at λ = 214 nm is shown.

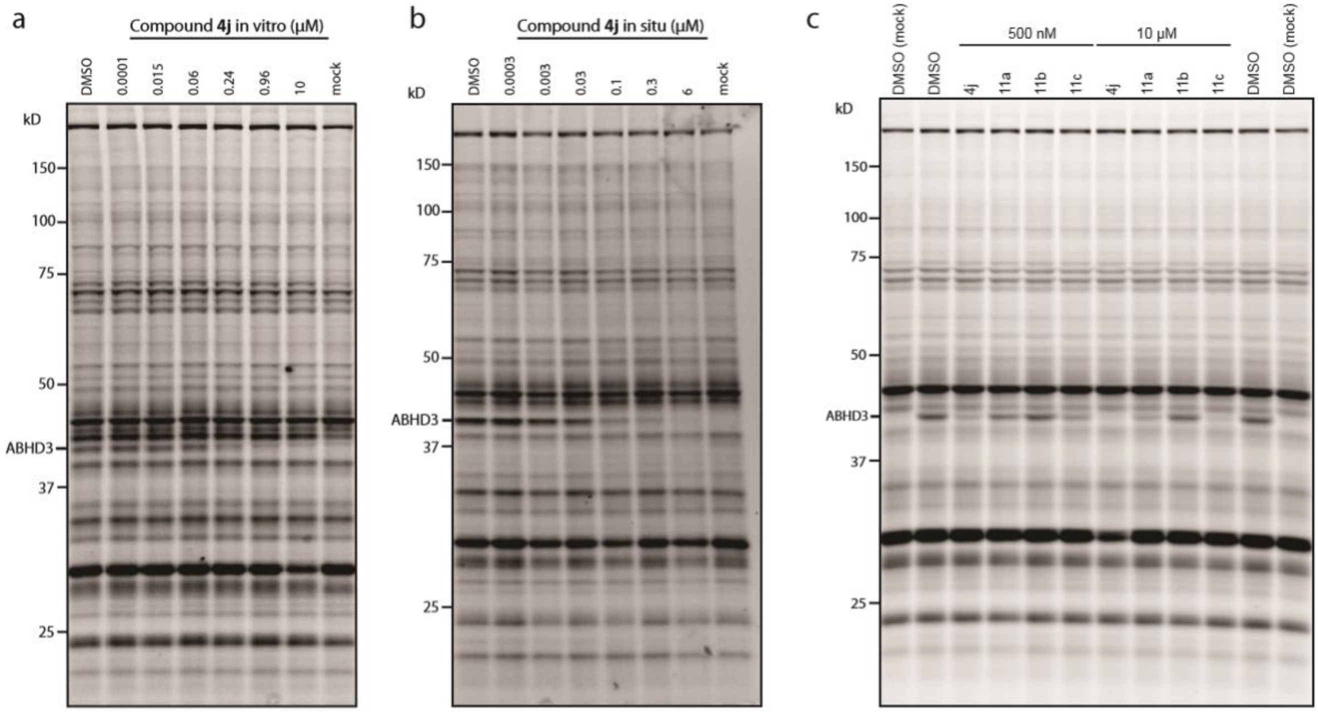

Supplementary Figure 5. a) Full gel for Fig. 5a. b) Full gel for Fig. 5b. c) Full gel for Fig. 6b.

Compound **3j**

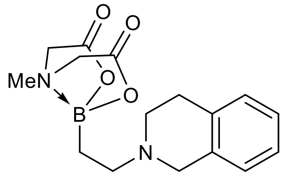

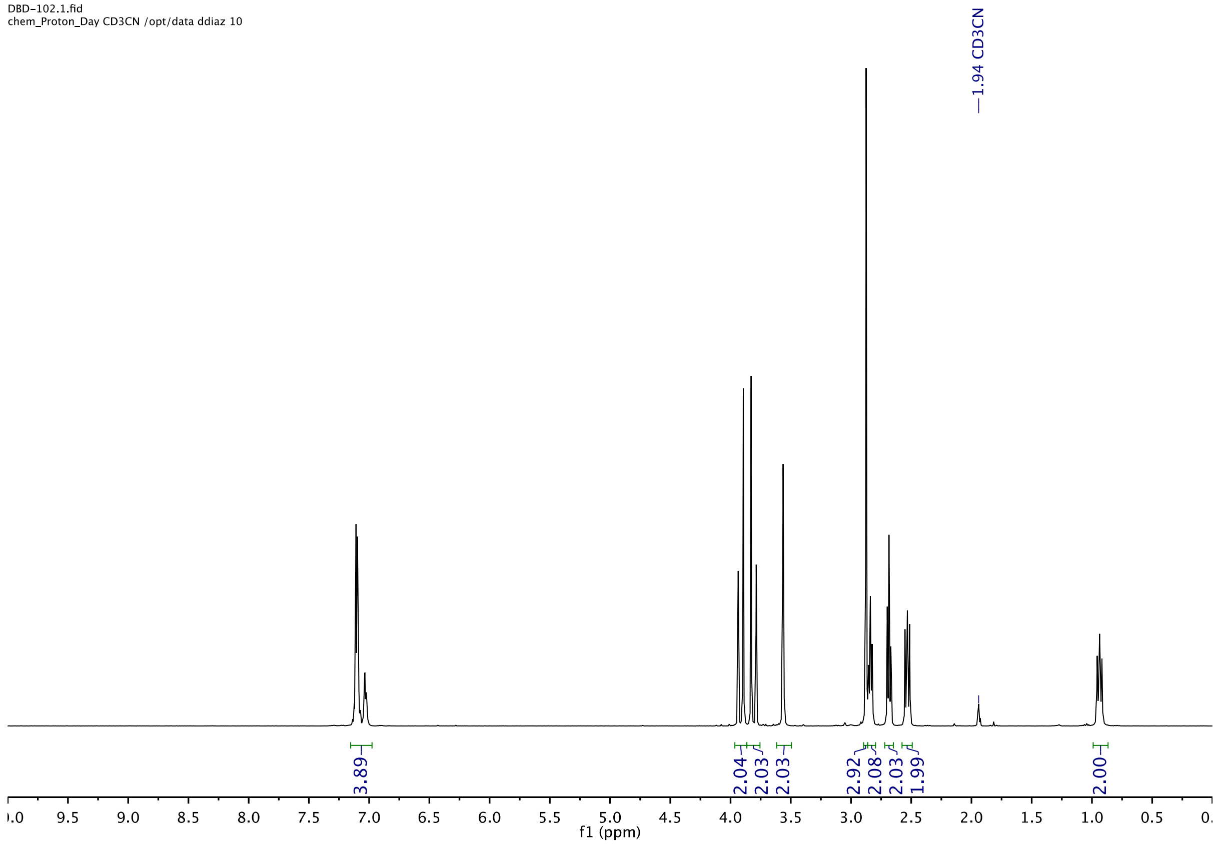

Supplementary Figure 5: ^{1}H NMR of **3j** in acetonitrile-d_3 at 25 °C.

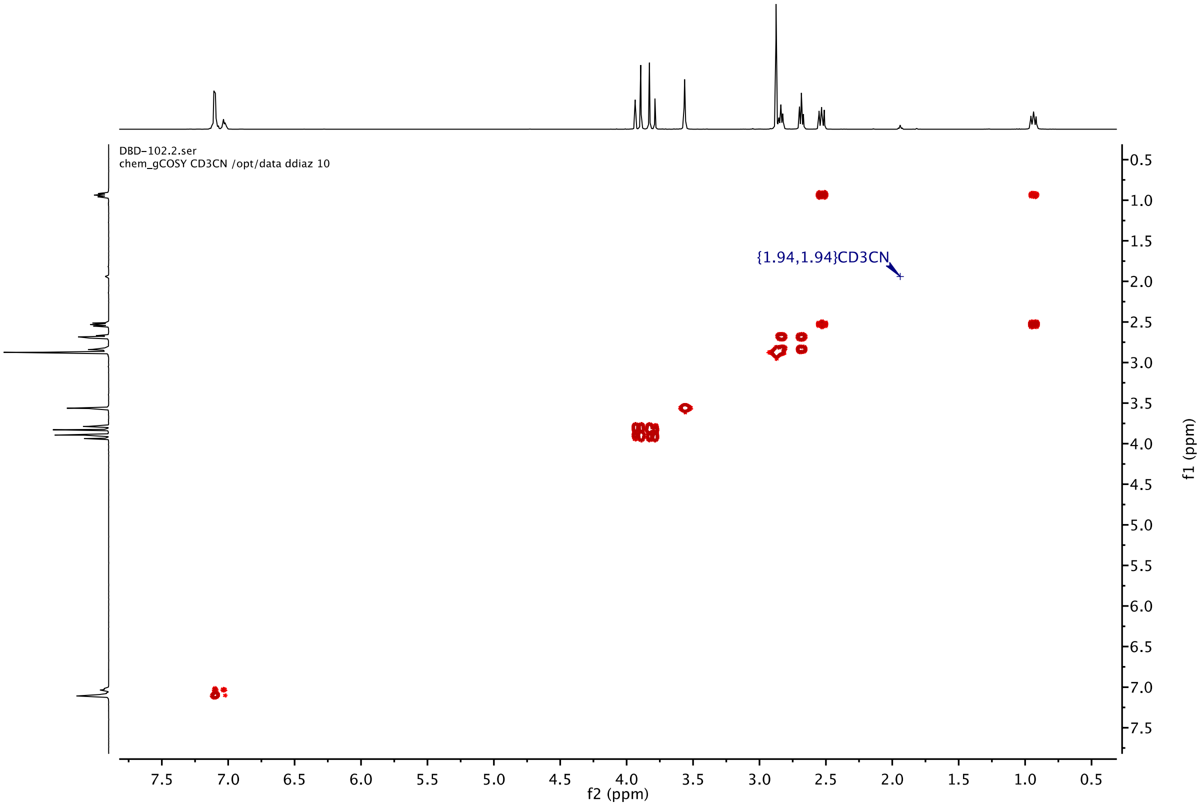

Supplementary Figure 6: ^{1}H-^{1}H COSY of **3j** in acetonitrile-d_3 at 25 °C.

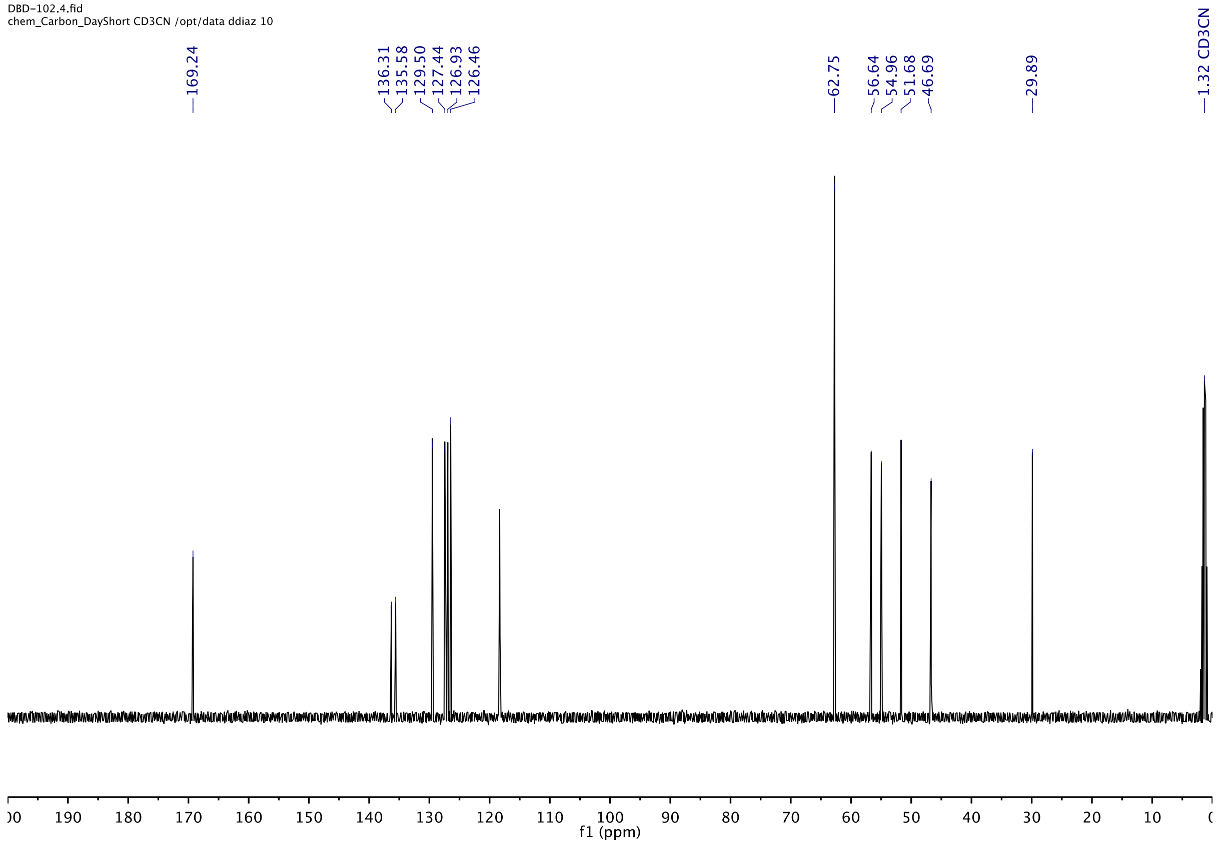

Supplementary Figure 7: ^{13}C NMR of **3j** in acetonitrile-*d*$_6$ at 25 °C.

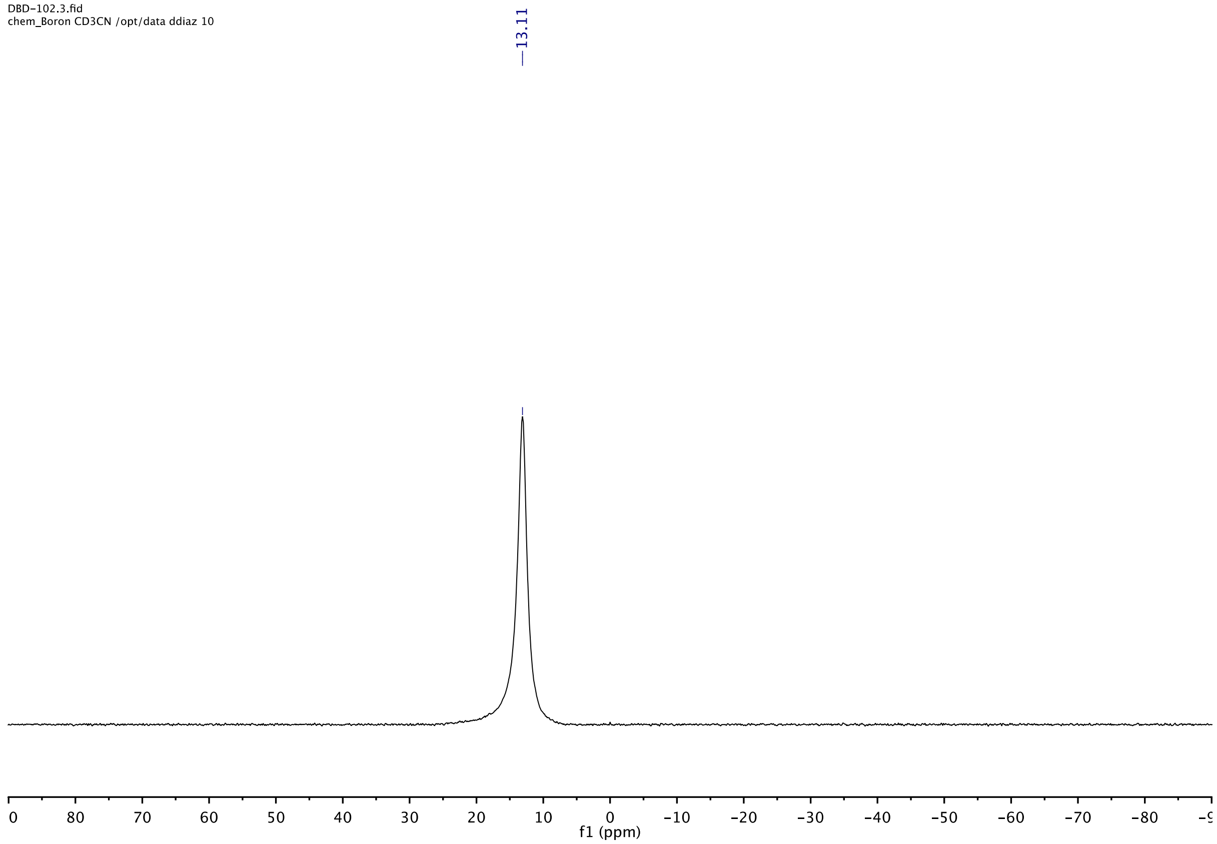

Supplementary Figure 8: ^{11}B NMR of **3j** in acetonitrile-d_3 at 25 °C.

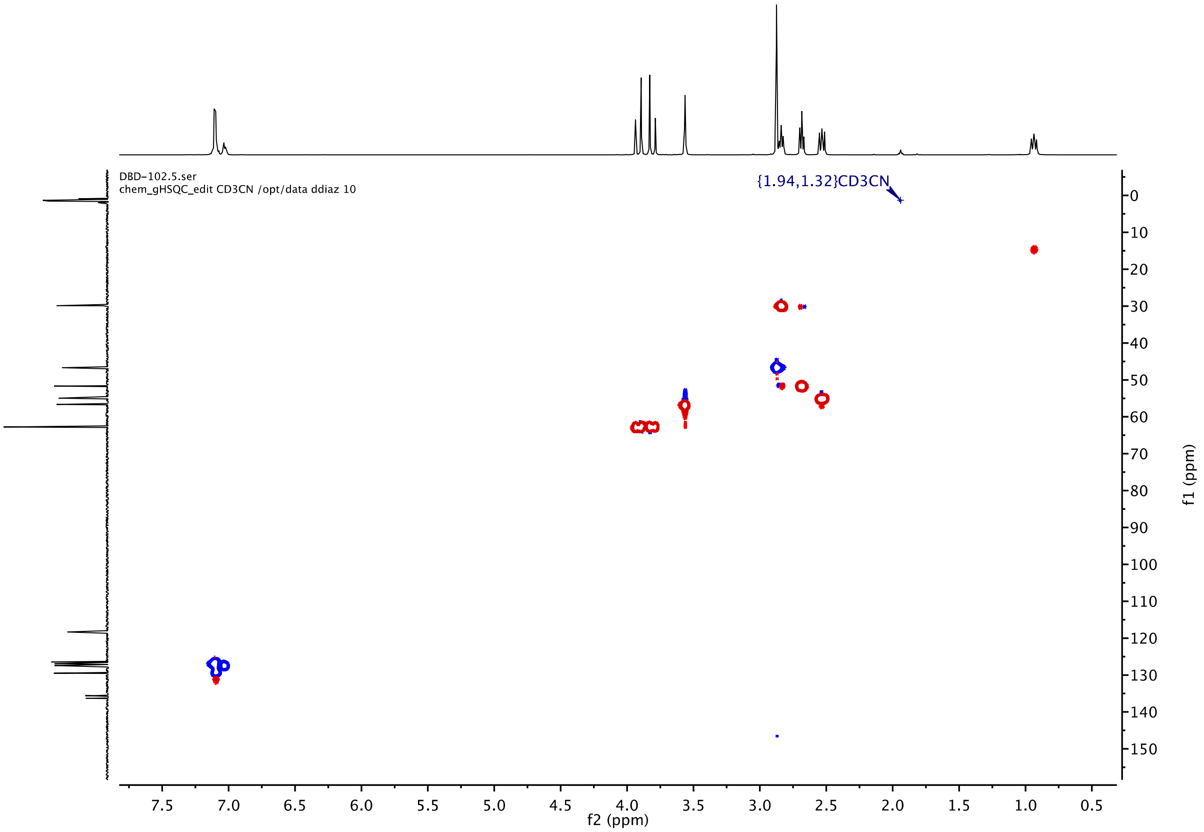

Supplementary Figure 9: ^{1}H-^{13}C HSQC NMR of **3j** in acetonitrile-d_3 at 25 °C.

Compound **3k**

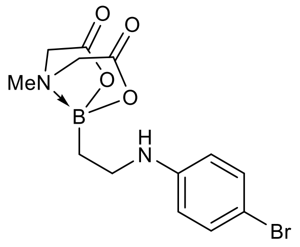

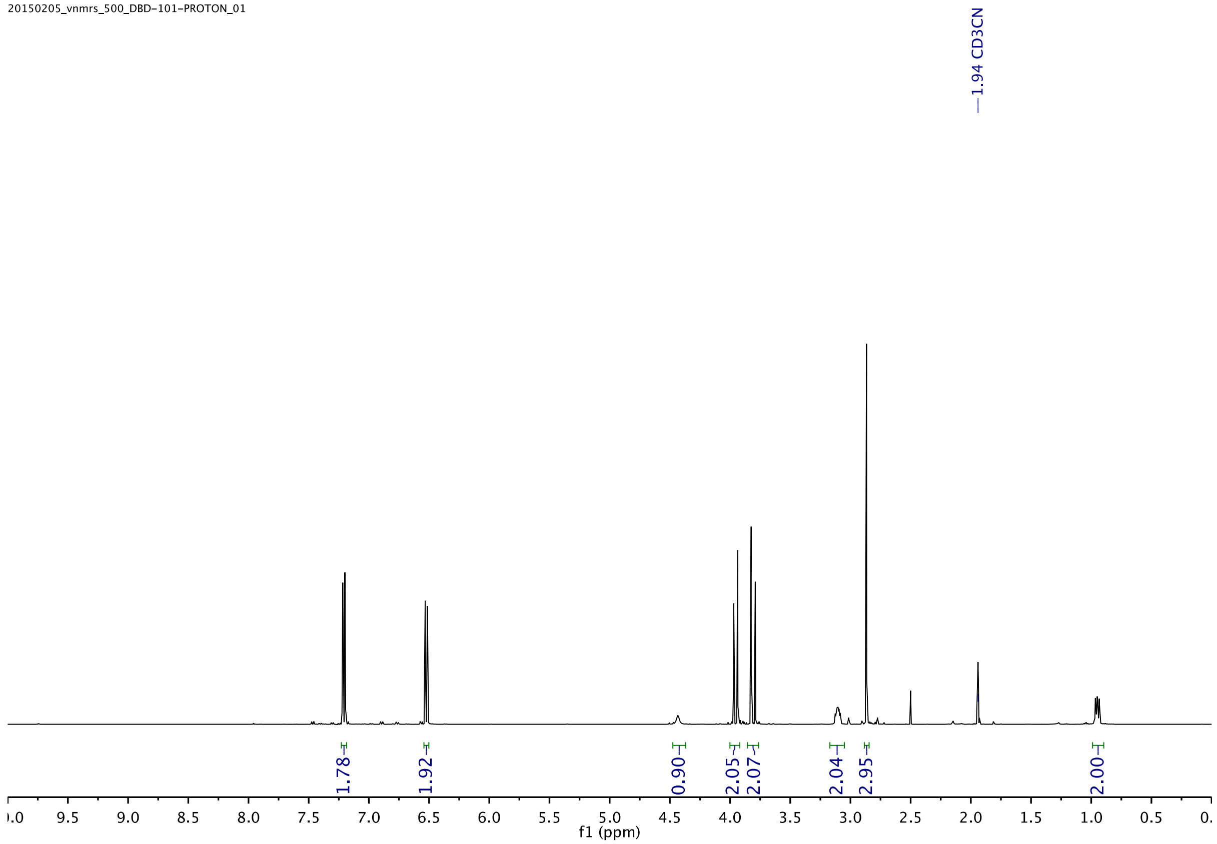

Supplementary Figure 10: ^{1}H NMR of **3k** in acetonitrile-d_3 at 25 °C.

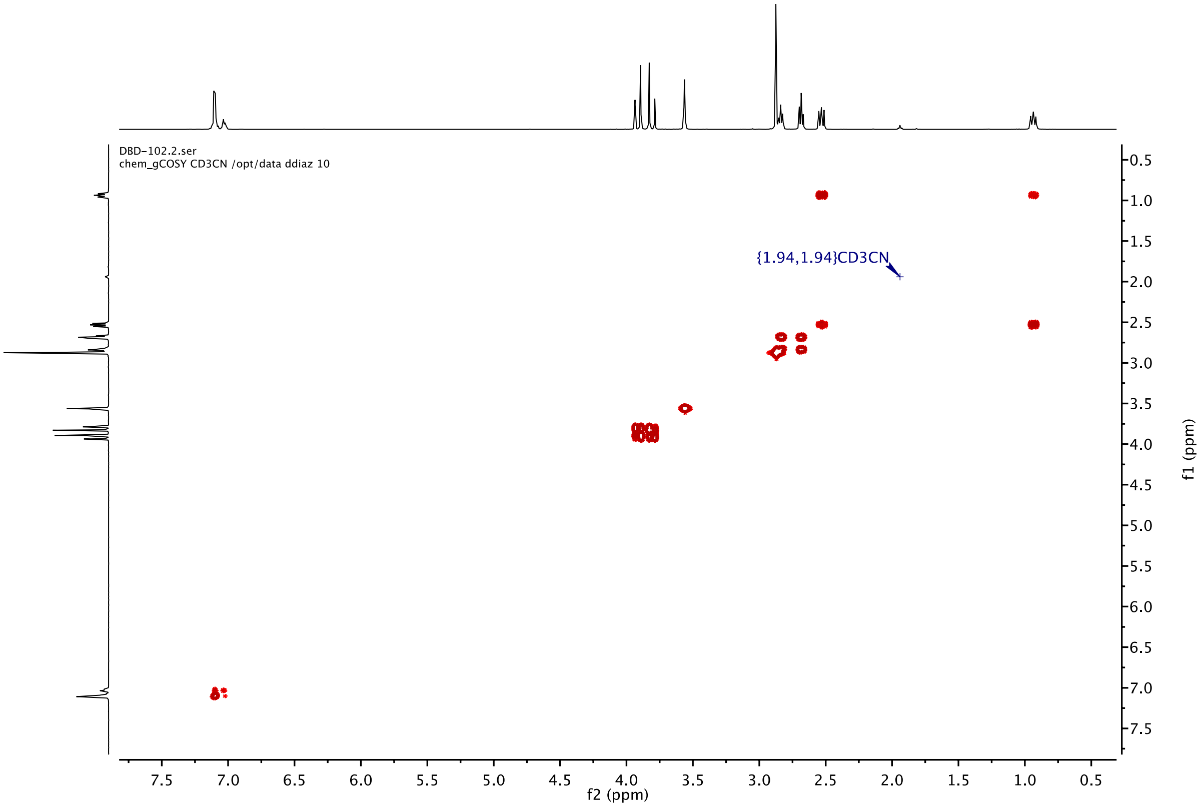

Supplementary Figure 11: ^{1}H-^{1}H COSY of **3k** in acetonitrile-d_3 at 25 °C.

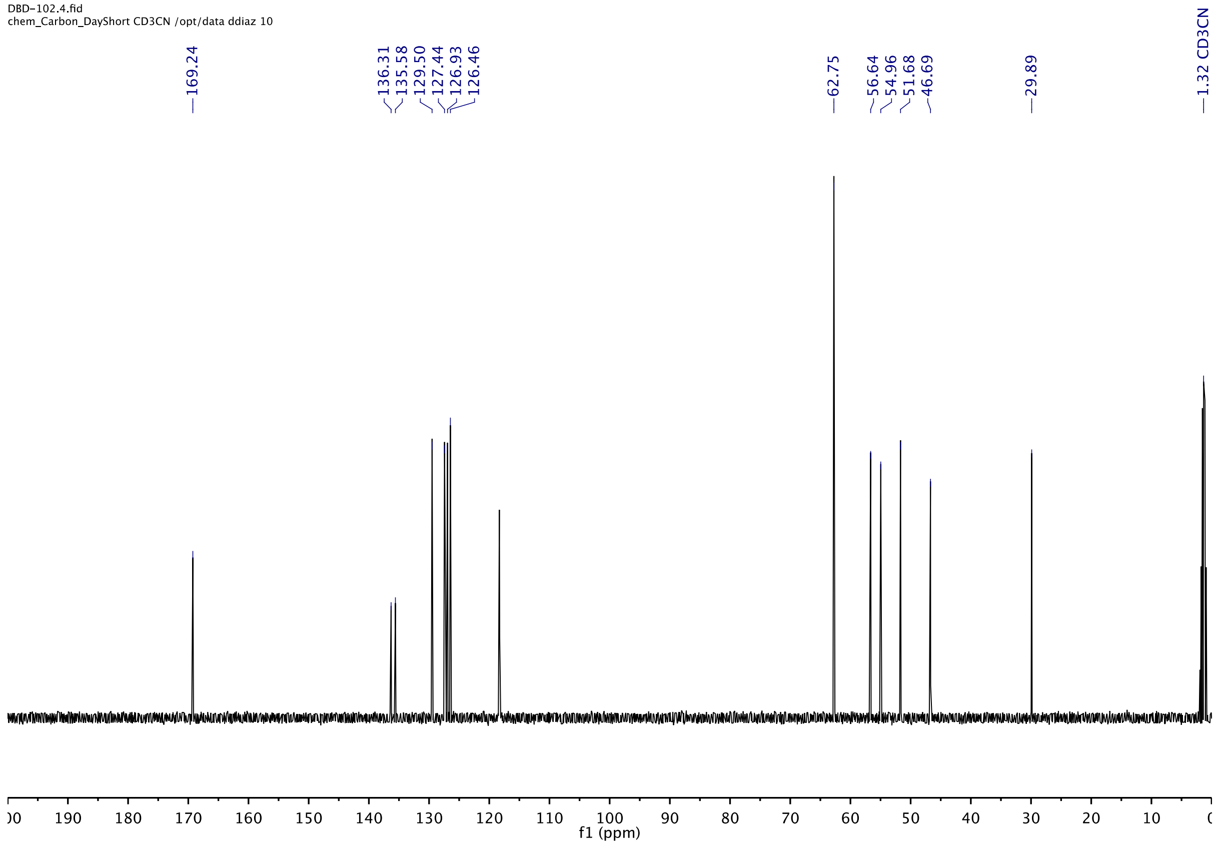

466

467 **Supplementary Figure 12:** ^{13}C NMR of **3k** in acetonitrile-d_6 at 25 °C.

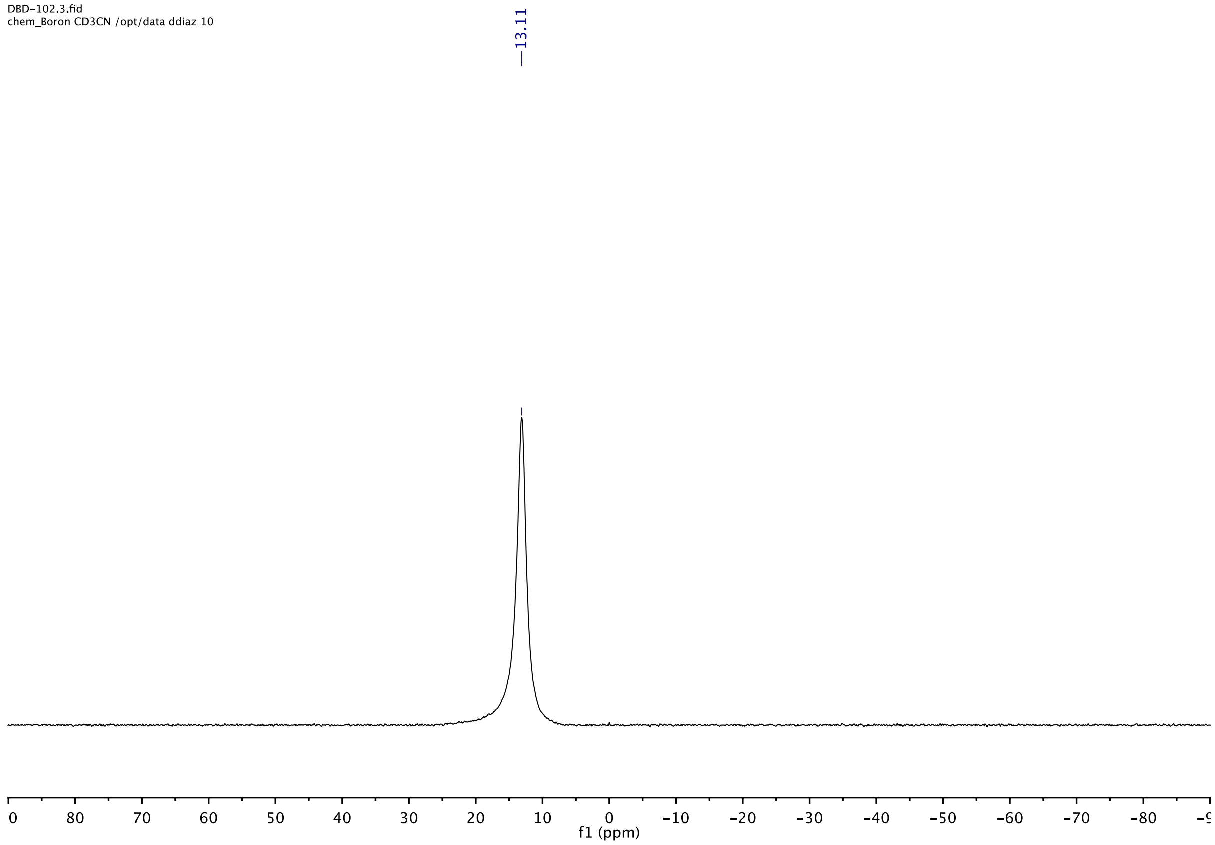

Supplementary Figure 13: ^{11}B NMR of **3k** in acetonitrile-d_3 at 25 °C.

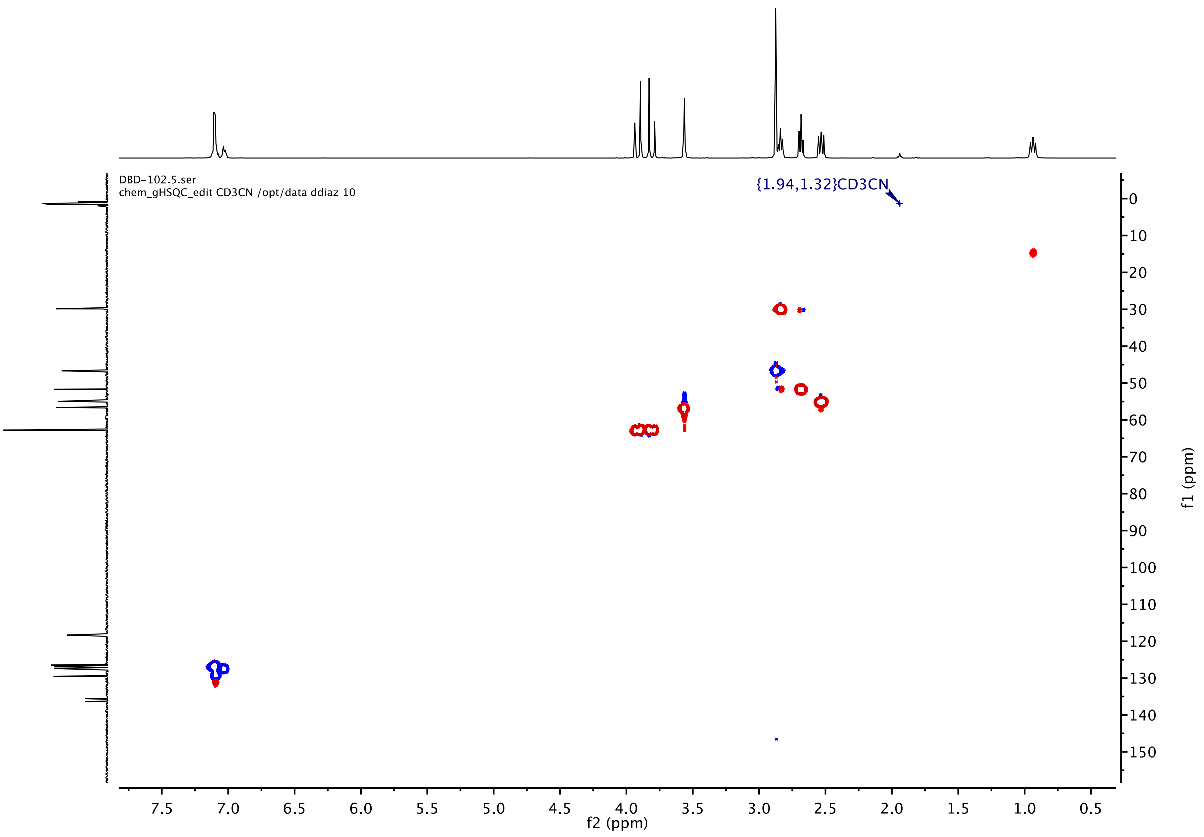

Supplementary Figure 14: ^{1}H-^{13}C HSQC NMR of **3k** in acetonitrile-d_3 at 25 °C.

Compound **4a**

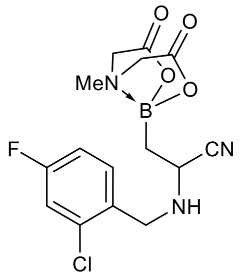

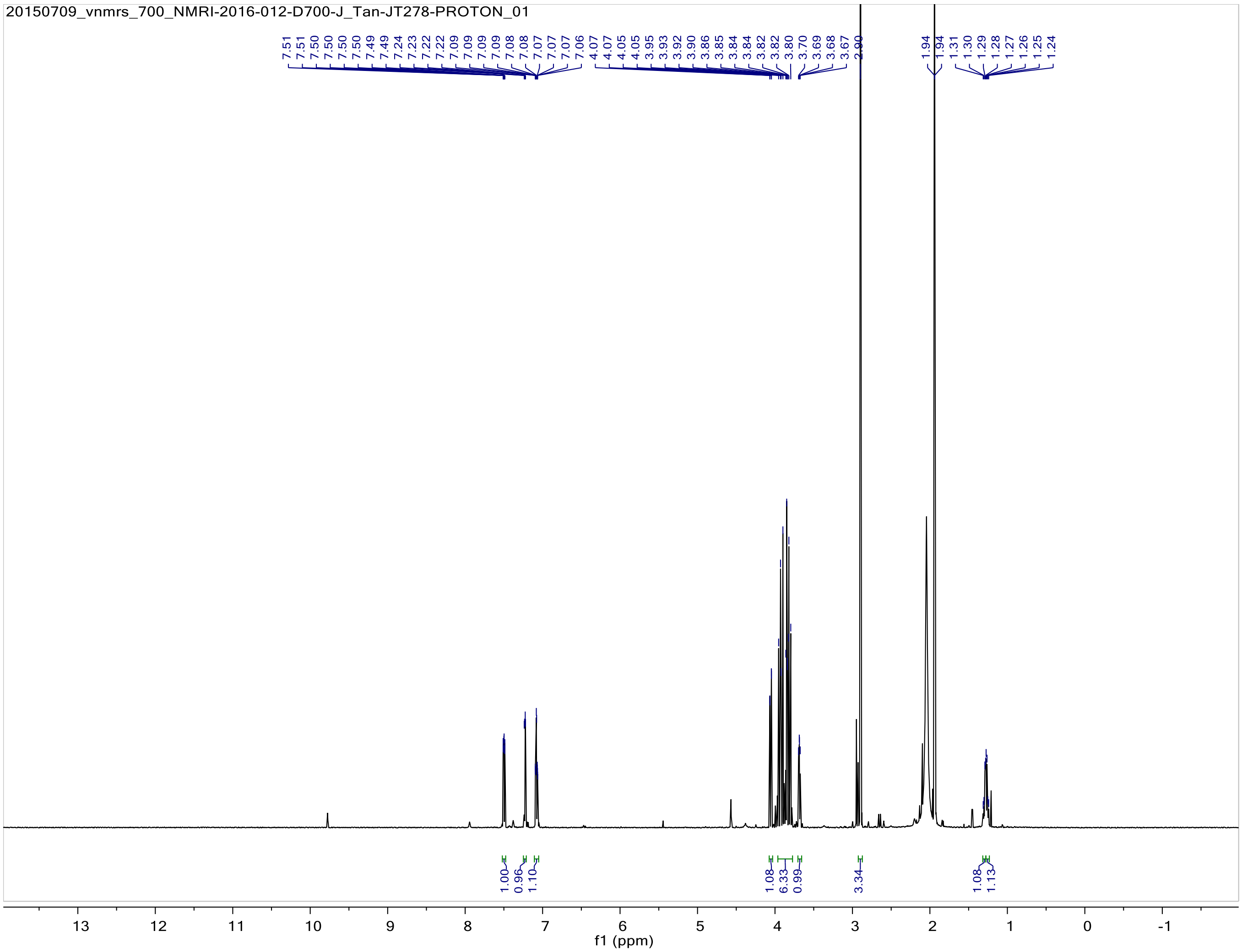

Supplementary Figure 15: ^{1}H NMR of **4a** in acetonitrile-d_3 at 60 °C.

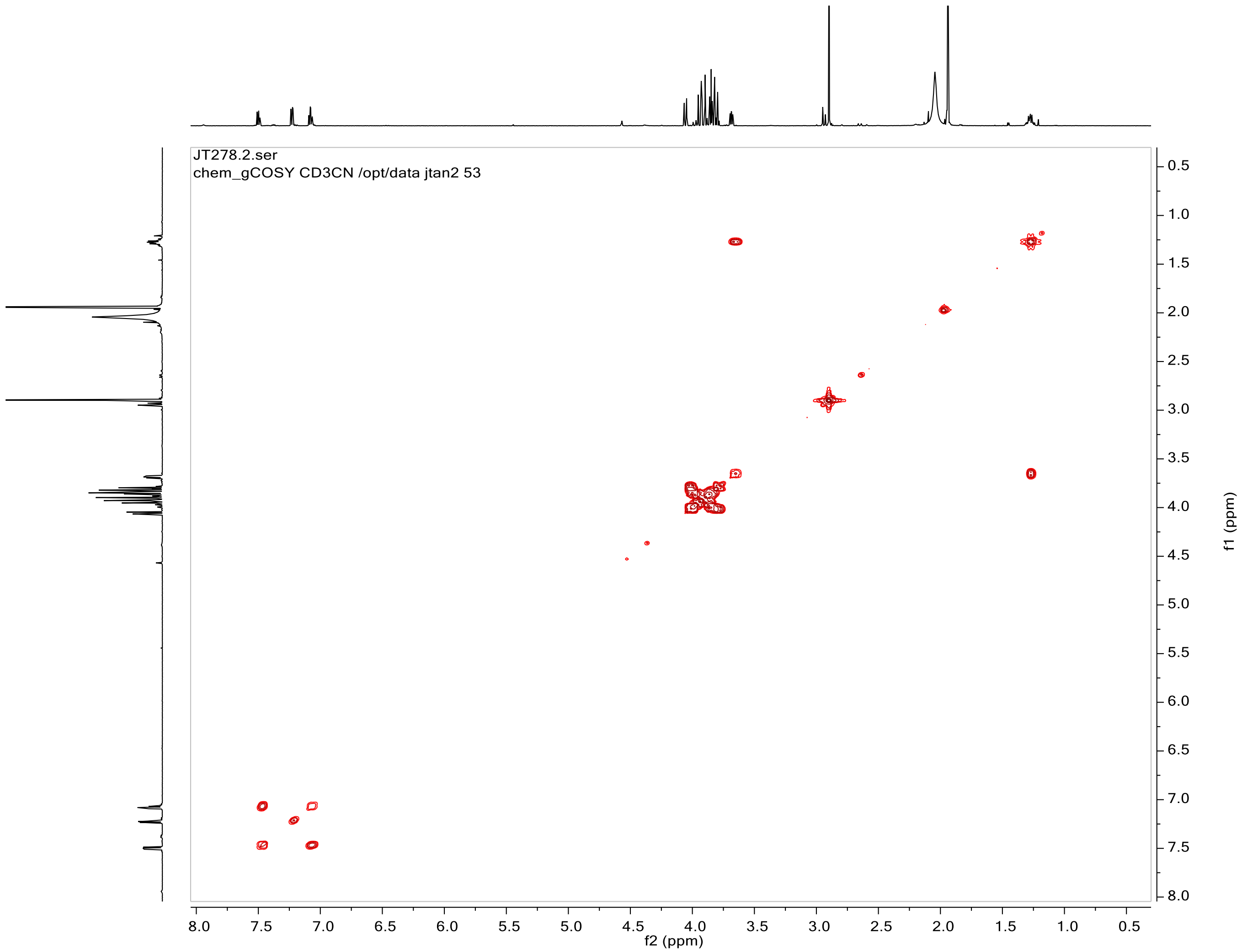

Supplementary Figure 16: ^{1}H-^{1}H COSY of **4a** in acetonitrile-d_3 at 25 °C.

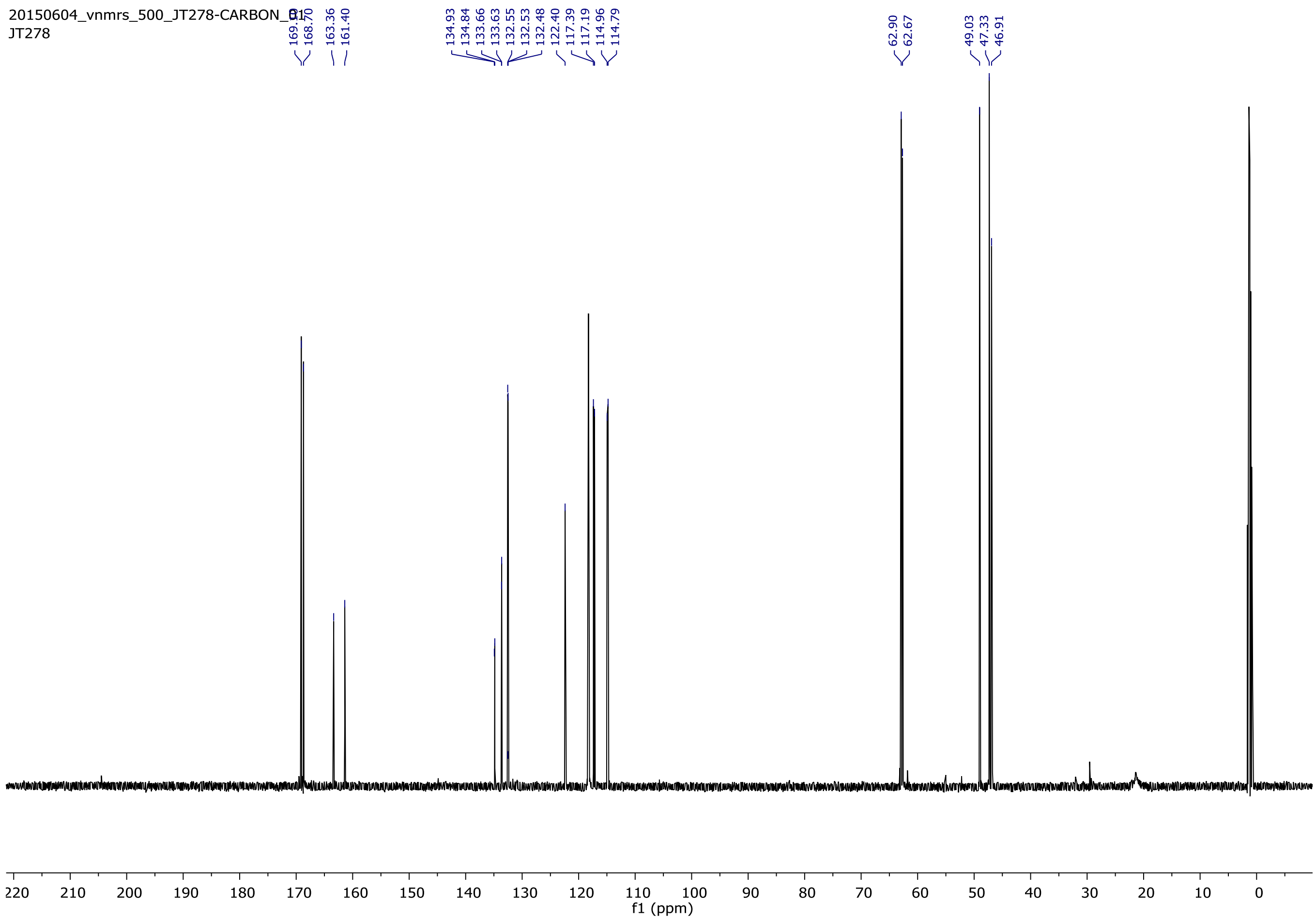

Supplementary Figure 17: ^{13}C NMR of **4a** in acetonitrile-d_3 at 25 °C.

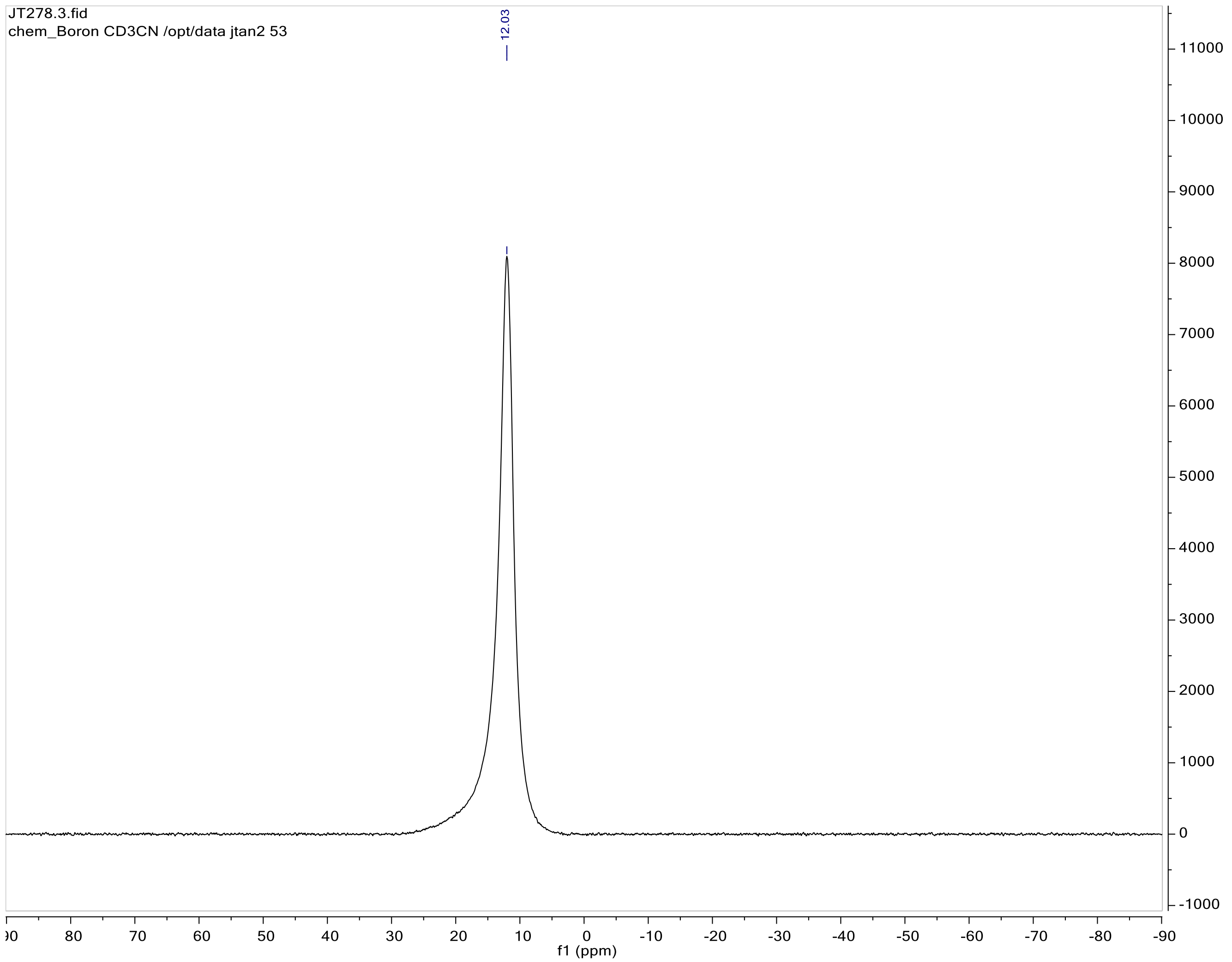

Supplementary Figure 18: ^{11}B NMR of **4a** in acetonitrile-d_3 at 25 °C.

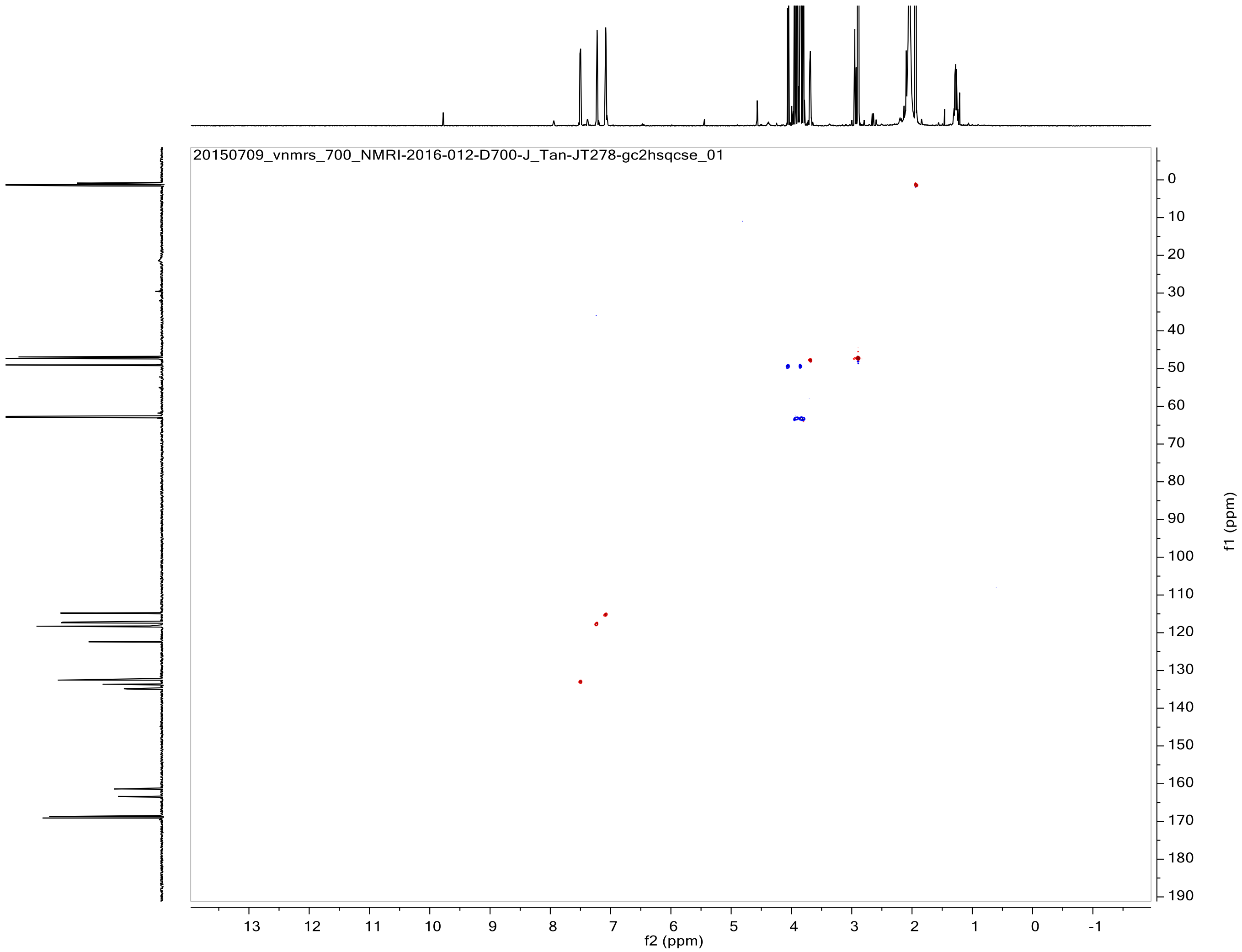

Supplementary Figure 19: ^{1}H-^{13}C HSQC NMR of **4a** in acetonitrile-d_3 at 60 °C.

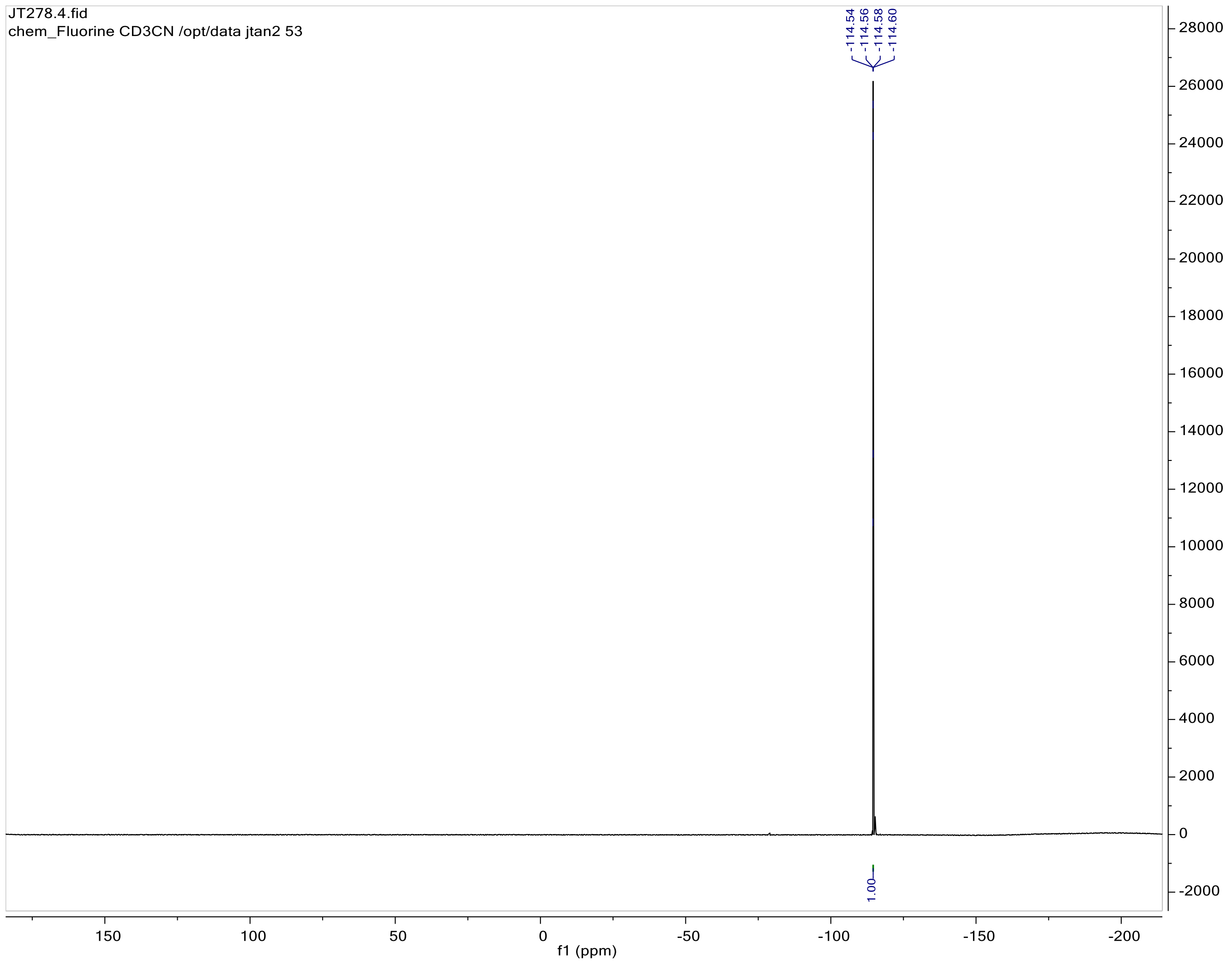

Supplementary Figure 20: ^{19}F NMR of **4a** in acetonitrile-d_3 at 25 °C.

Compound **4b**

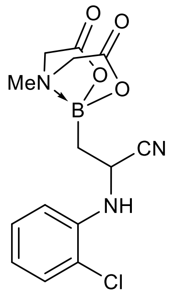

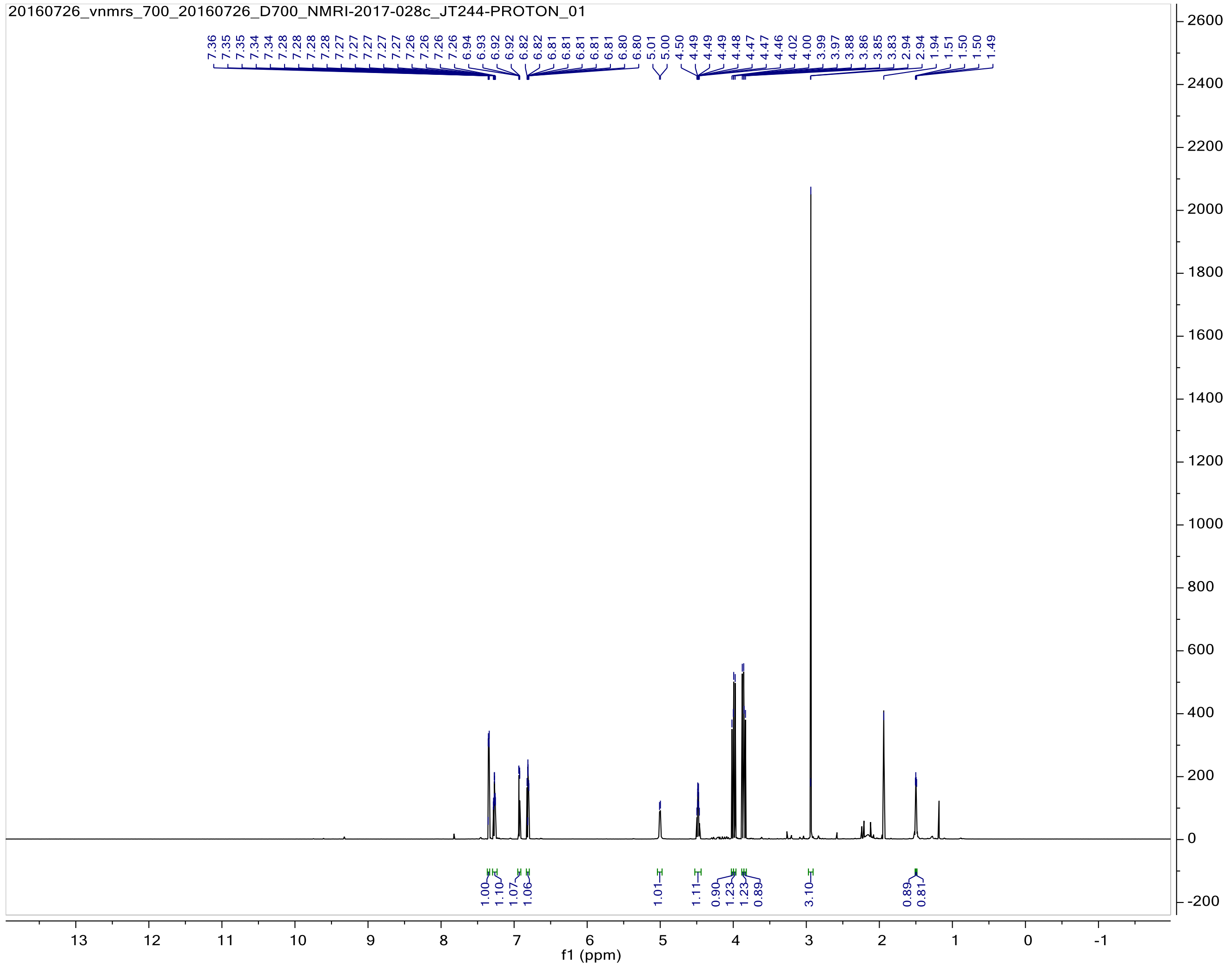

Supplementary Figure 21: ^{1}H NMR of **4b** in acetonitrile-d_3 at 25 °C.

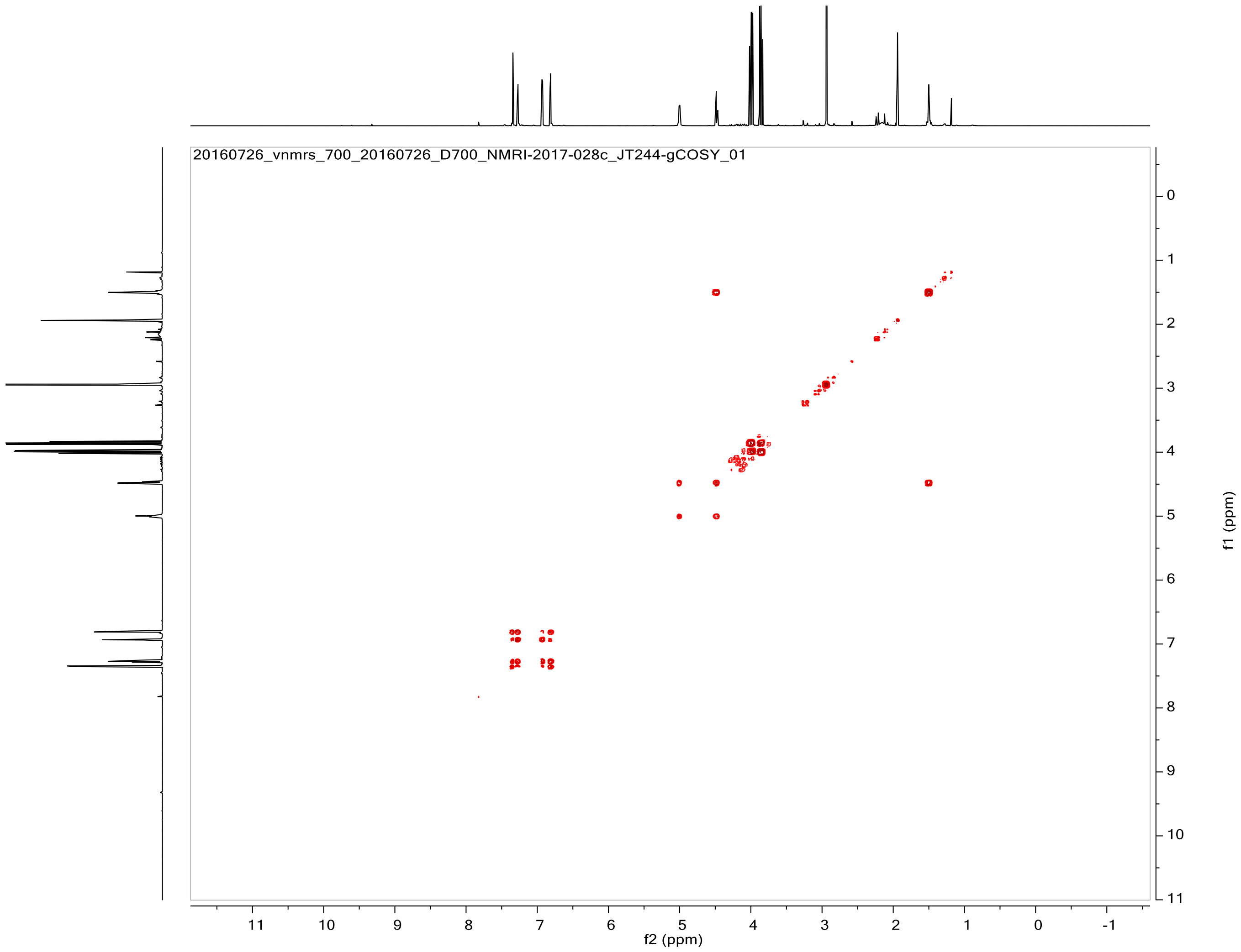

Supplementary Figure 22: ^{1}H-^{1}H COSY of **4b** in acetonitrile-d_3 at 25 °C.

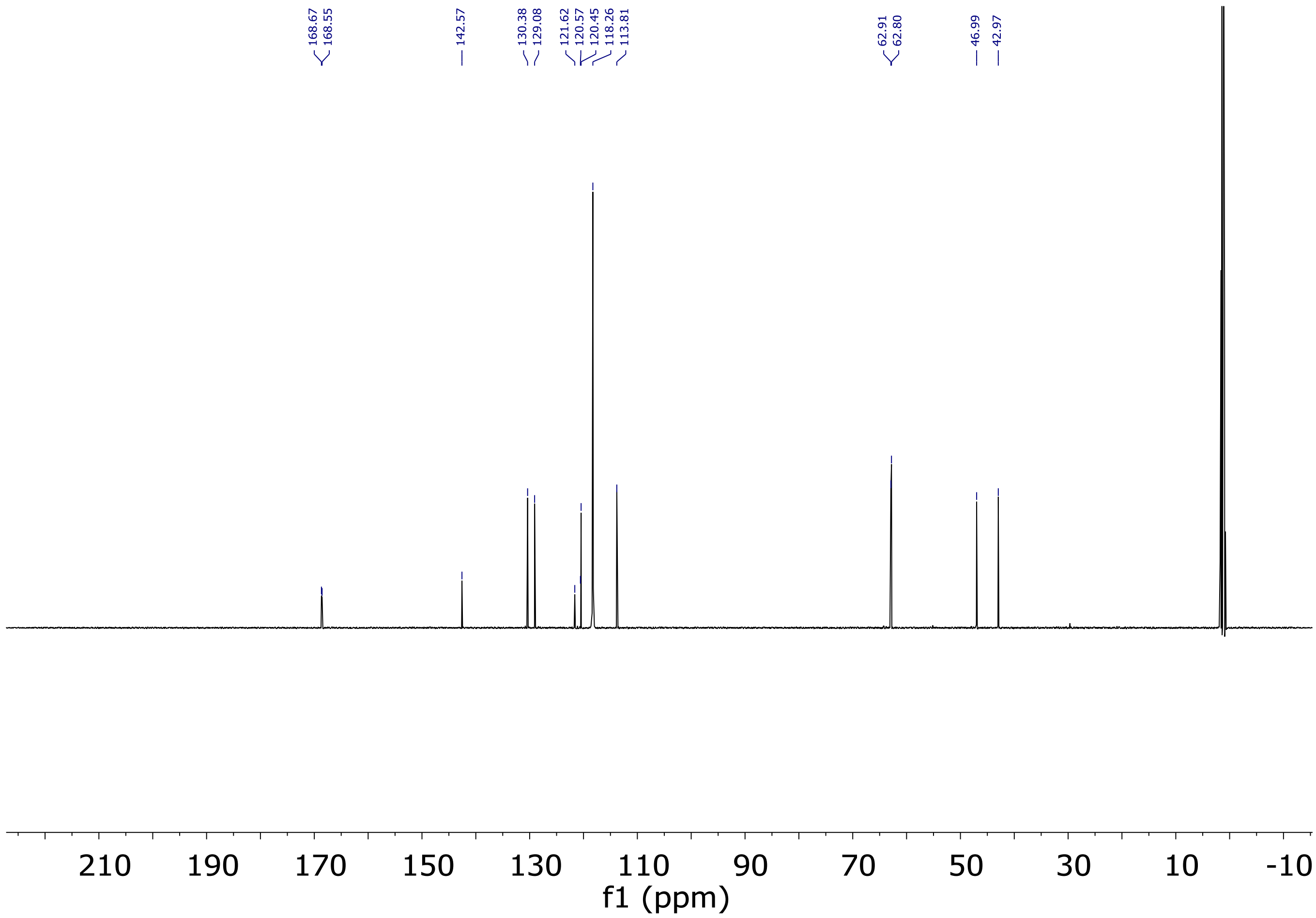

Supplementary Figure 23: ^{13}C NMR of **4b** in acetonitrile-d_3 at 25 °C.

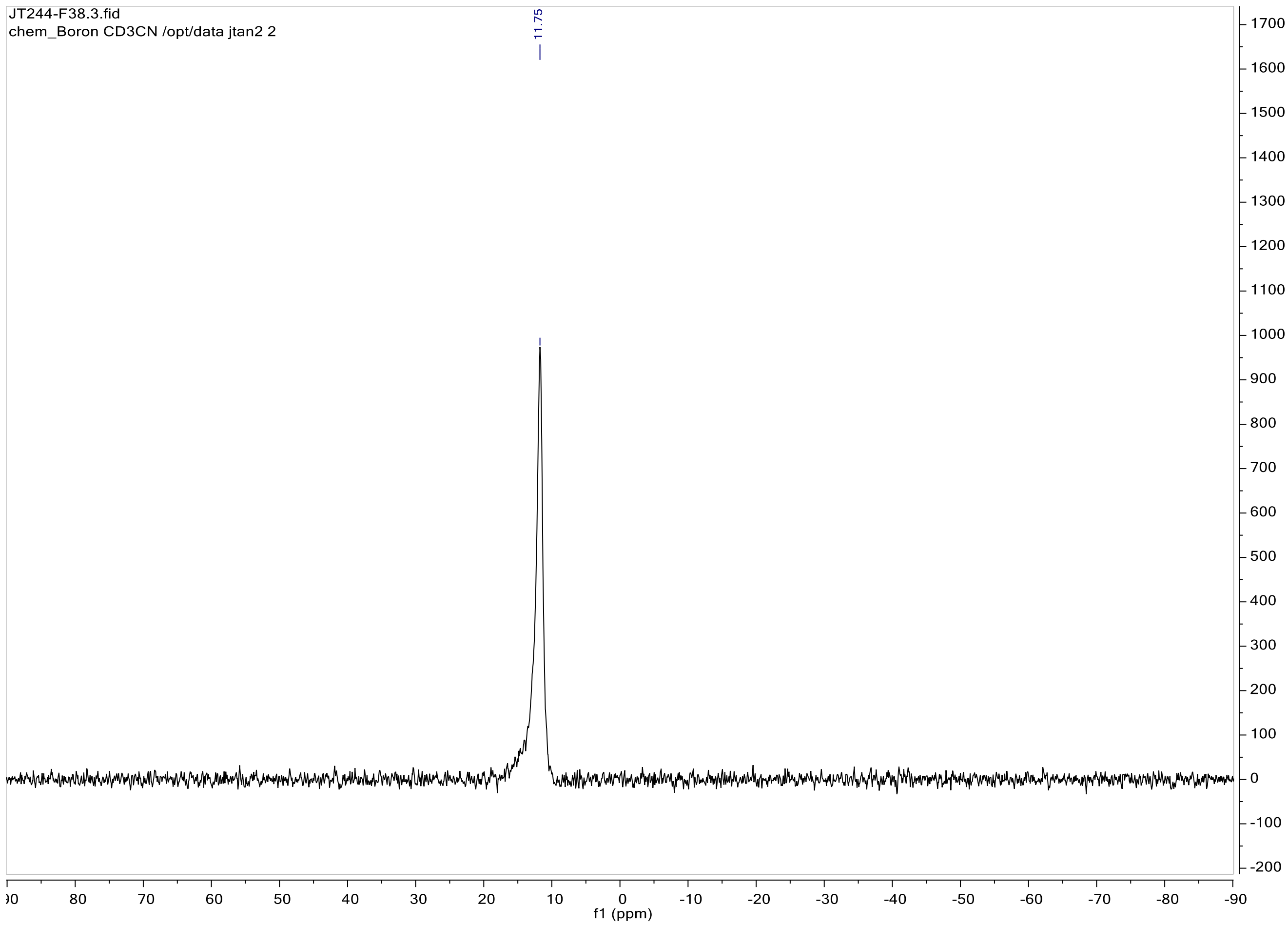

Supplementary Figure 24: ^{11}B NMR of **4b** in acetonitrile-d_3 at 25 °C.

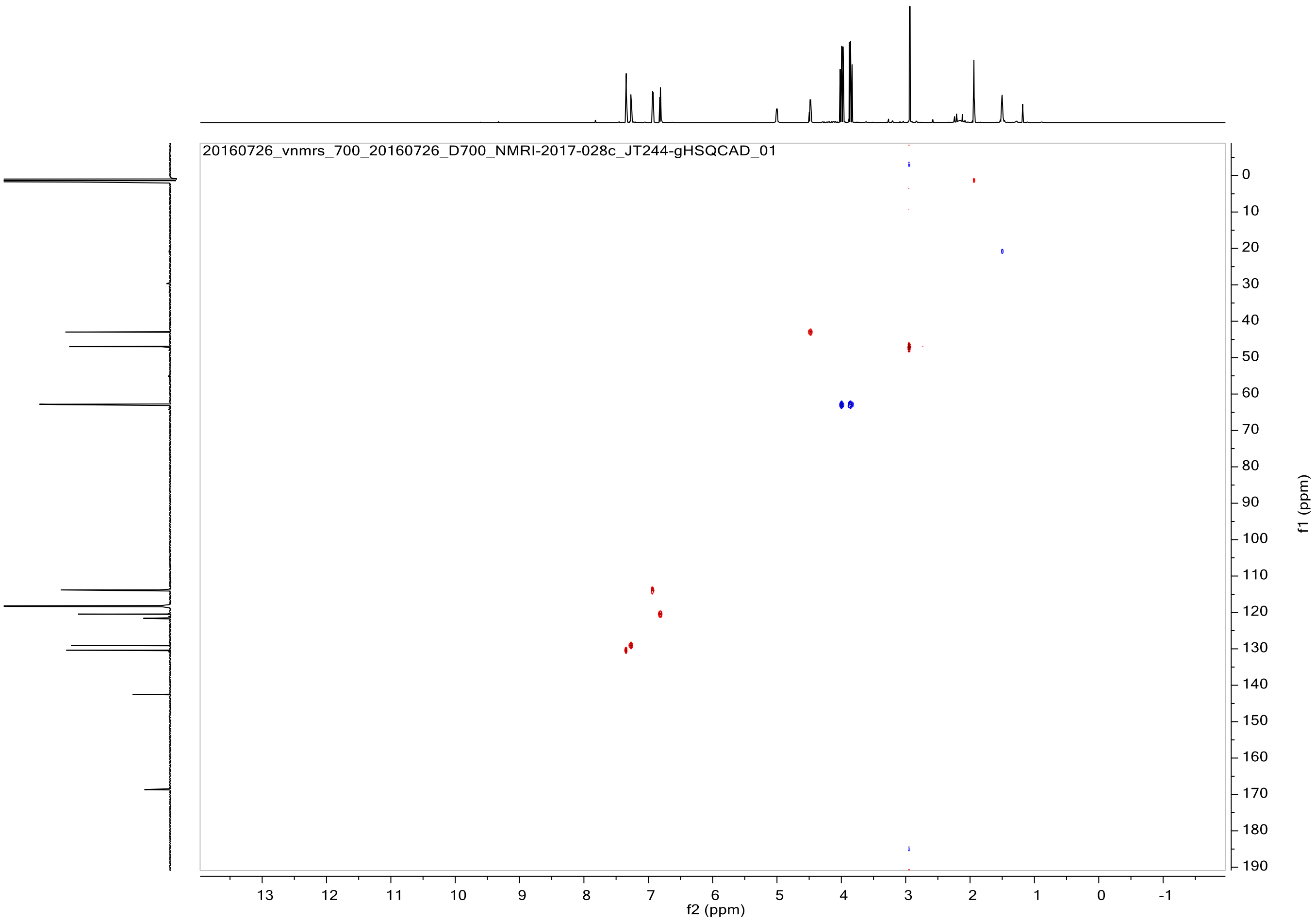

Supplementary Figure 25: ^{1}H-^{13}C HSQC NMR of **4b** in acetonitrile-d_3 at 60 °C.

Compound **4c**

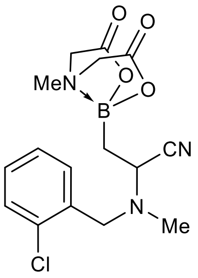

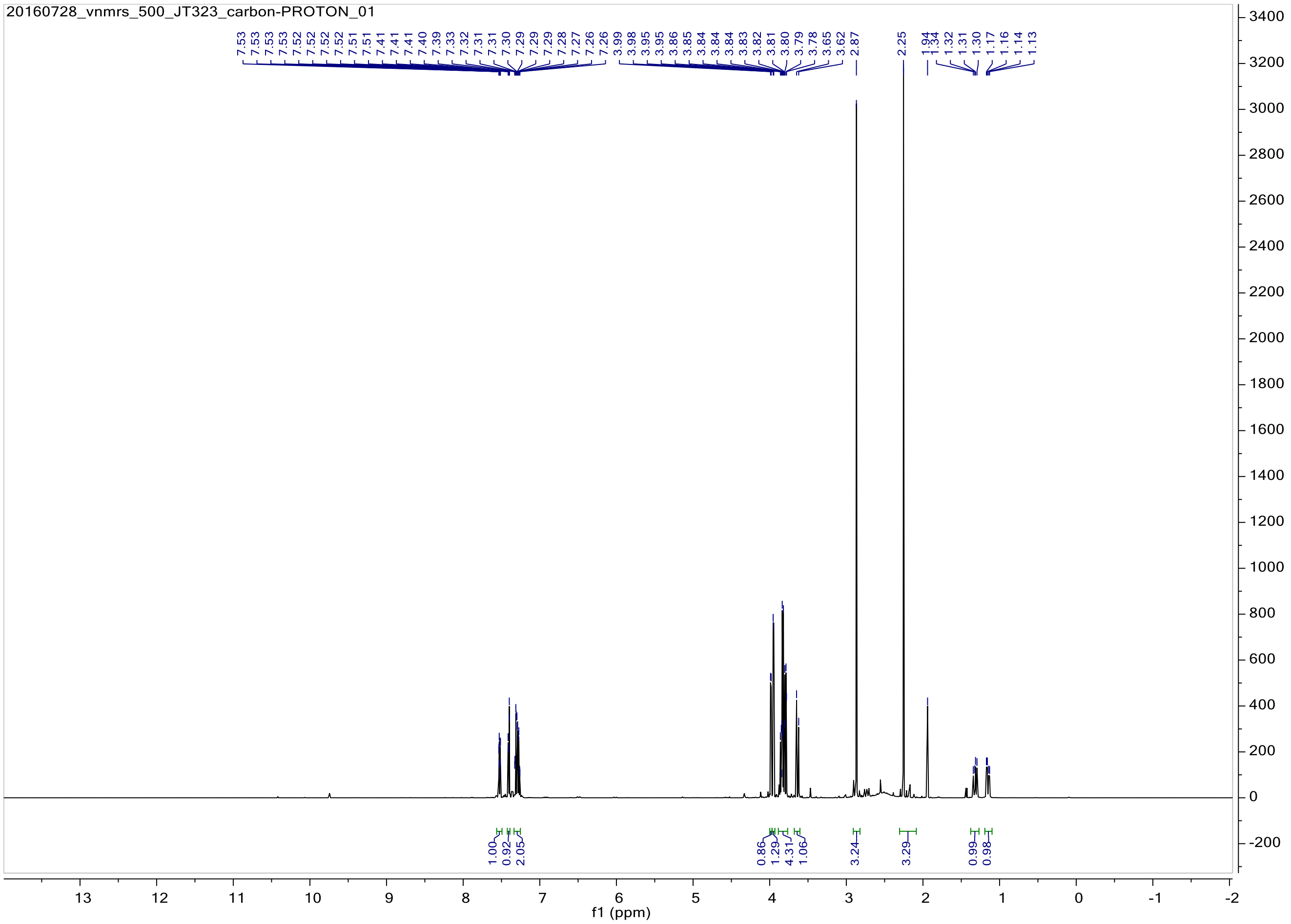

Supplementary Figure 26: ^{1}H NMR of **4c** in acetonitrile-d_3 at 25 °C.

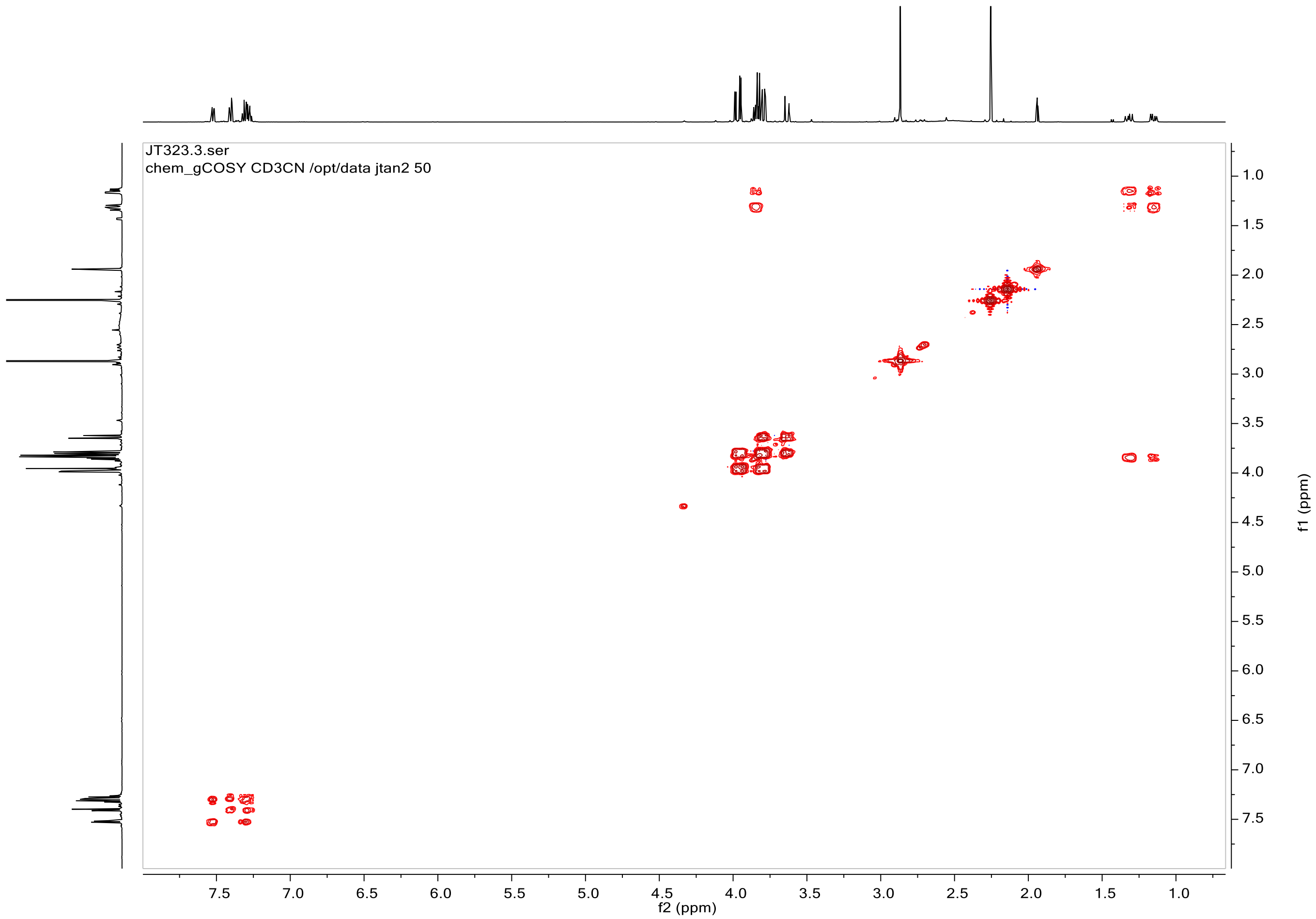

Supplementary Figure 27: ^{1}H-^{1}H COSY of **4c** in acetonitrile-d_3 at 25 °C.

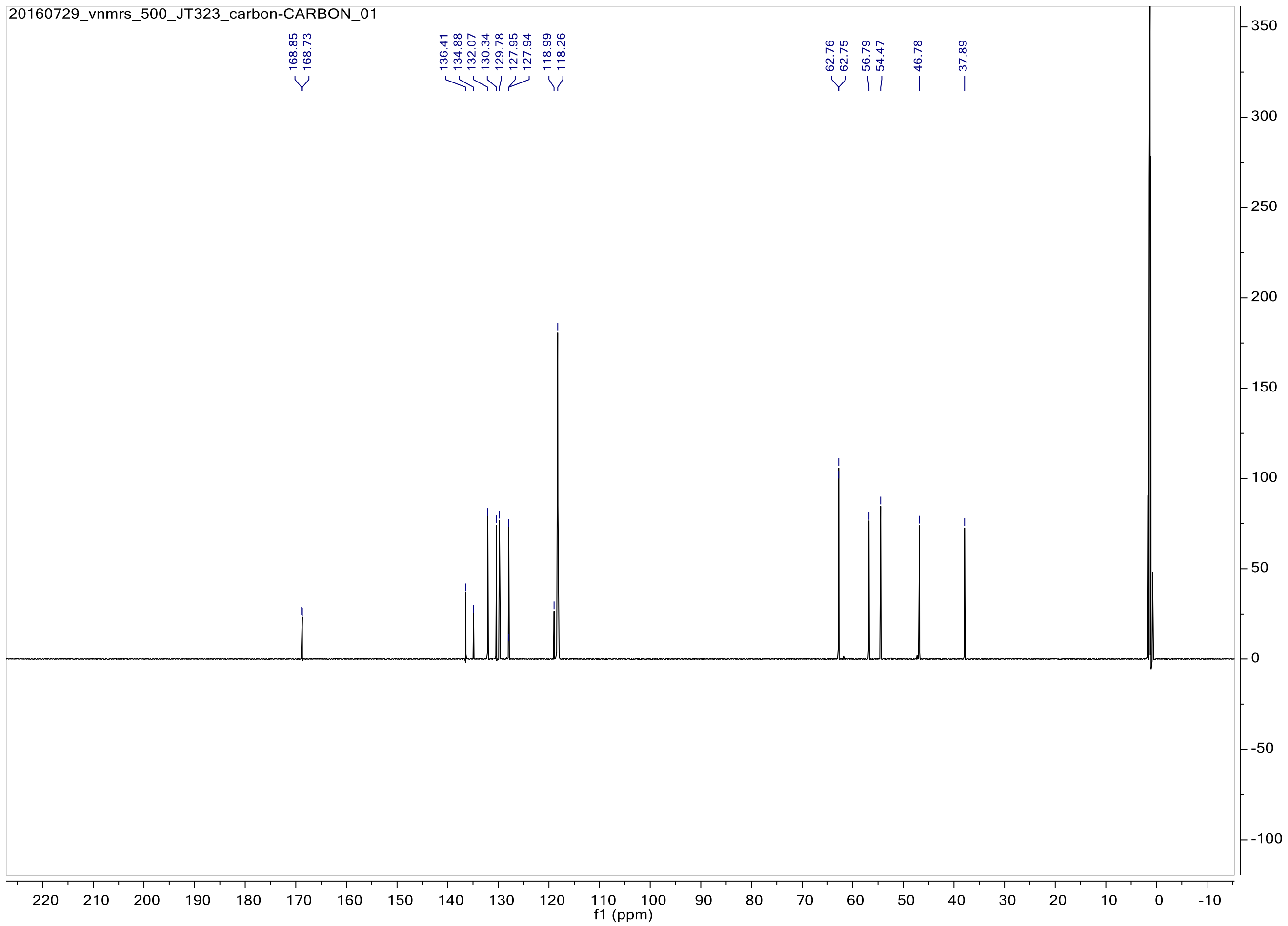

Supplementary Figure 28: ^{13}C NMR of **4c** in acetonitrile-d_3 at 25 °C.

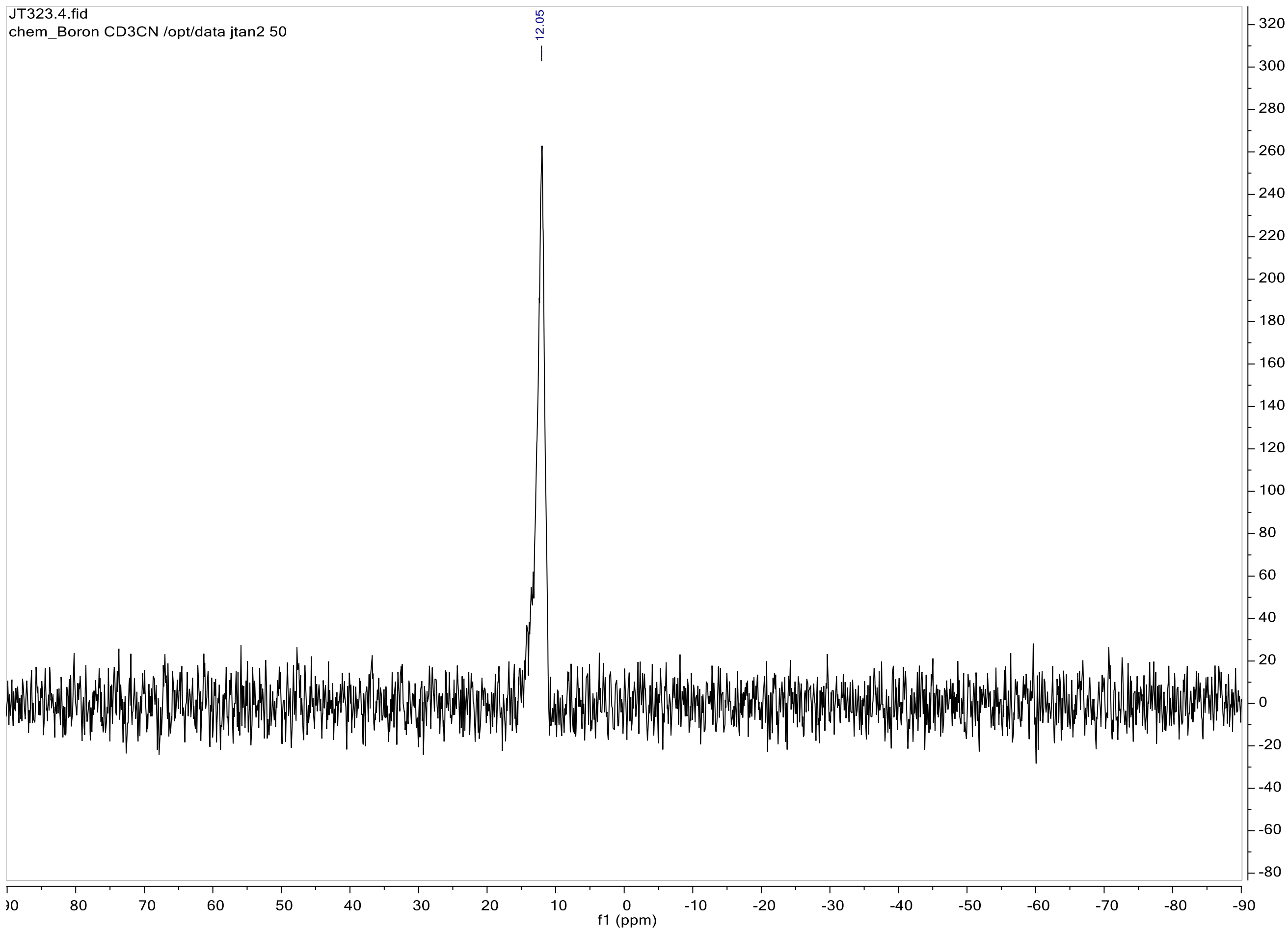

Supplementary Figure 29: ^{11}B NMR of **4c** in acetonitrile-d_3 at 25 °C.

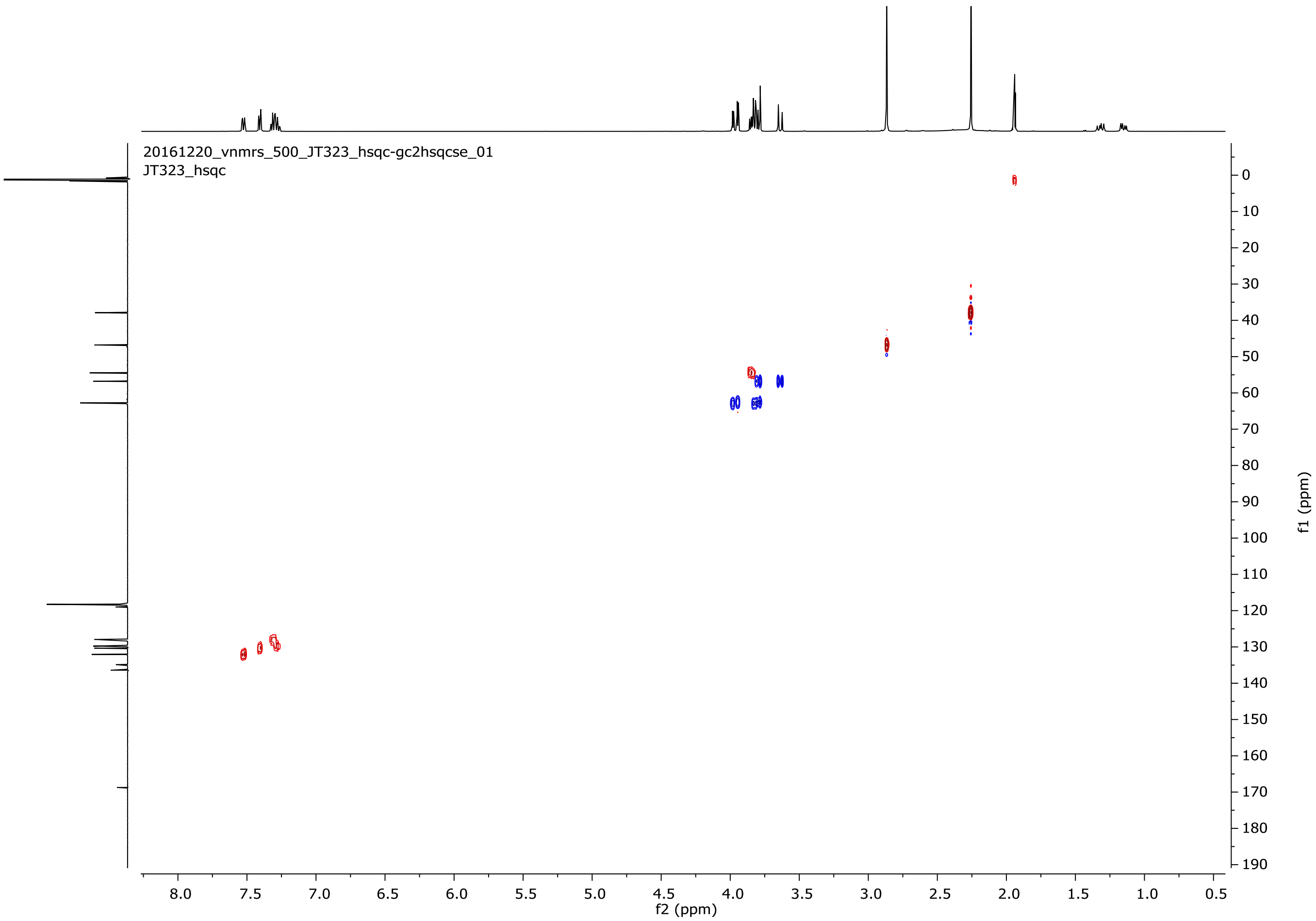

Supplementary Figure 30: ^{1}H-^{13}C HSQC NMR of **4c** in acetonitrile-d_3 at 25 °C.

Compound **4d**

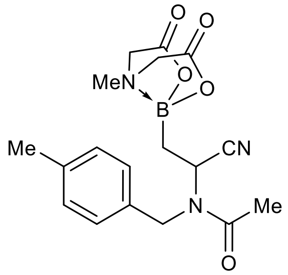

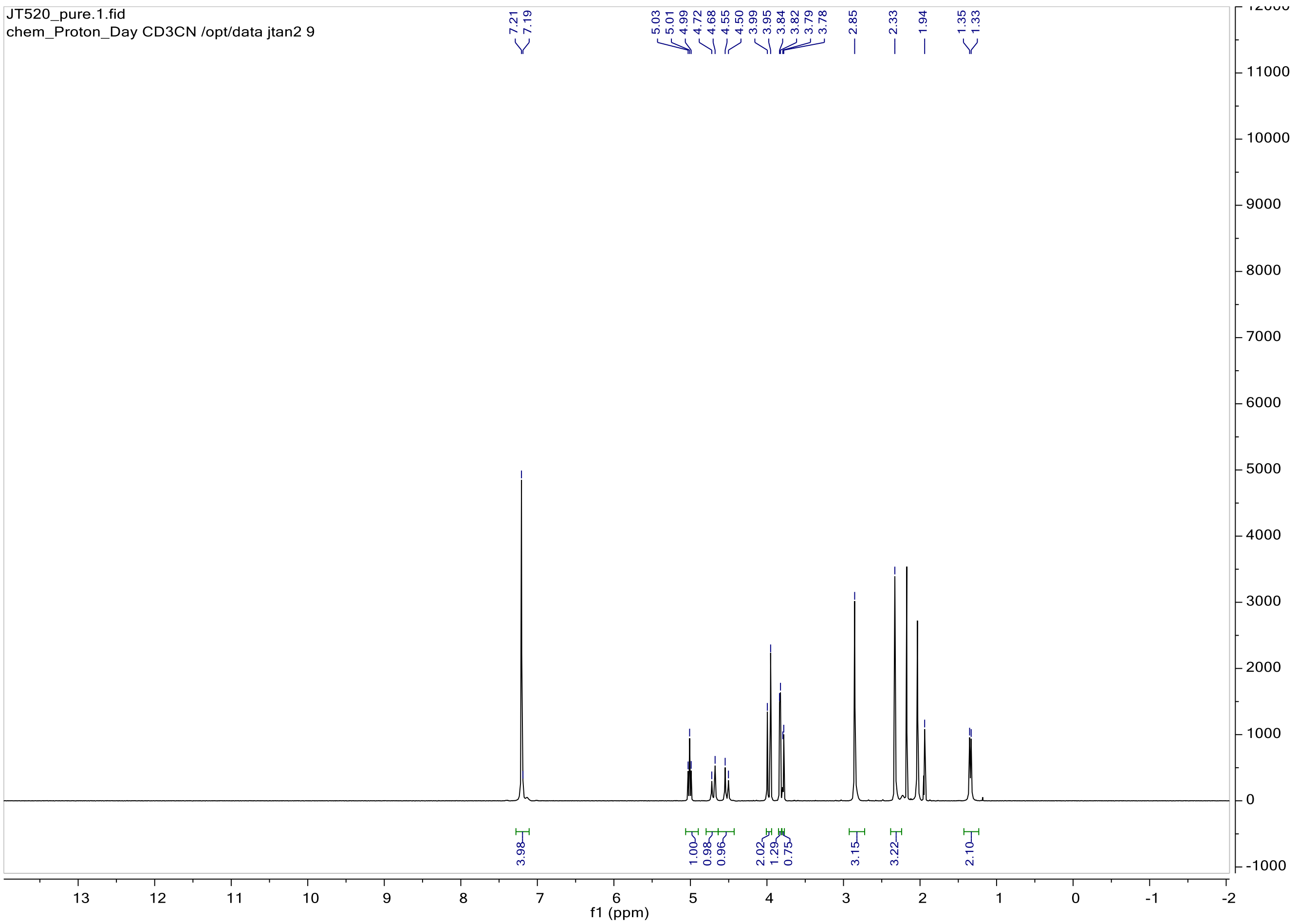

Supplementary Figure 31: ^{1}H NMR of **4d** in acetonitrile-d_3 at 25 °C.

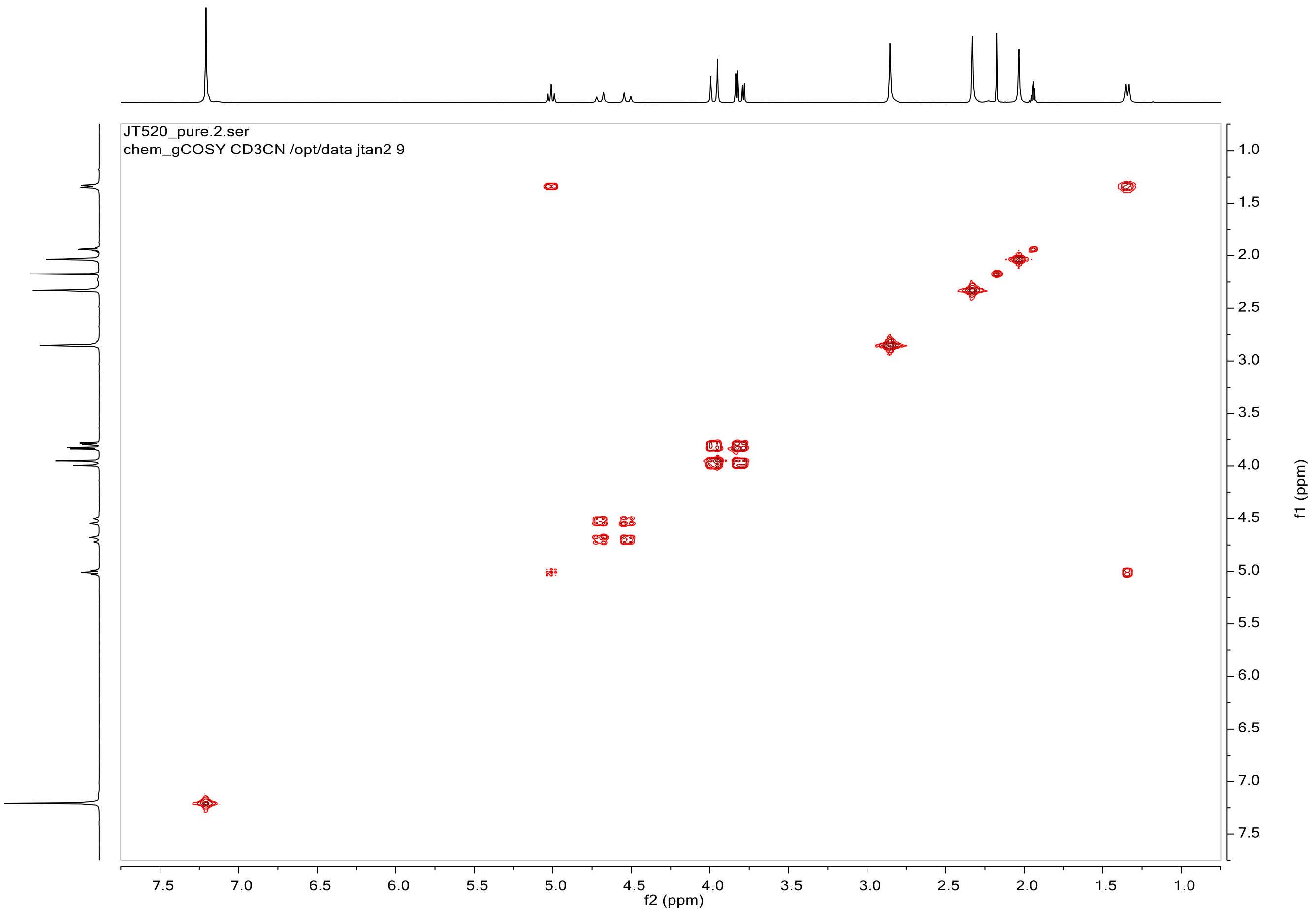

Supplementary Figure 32: ^{1}H-^{1}H COSY of **4d** in acetonitrile-d_3 at 25 °C.

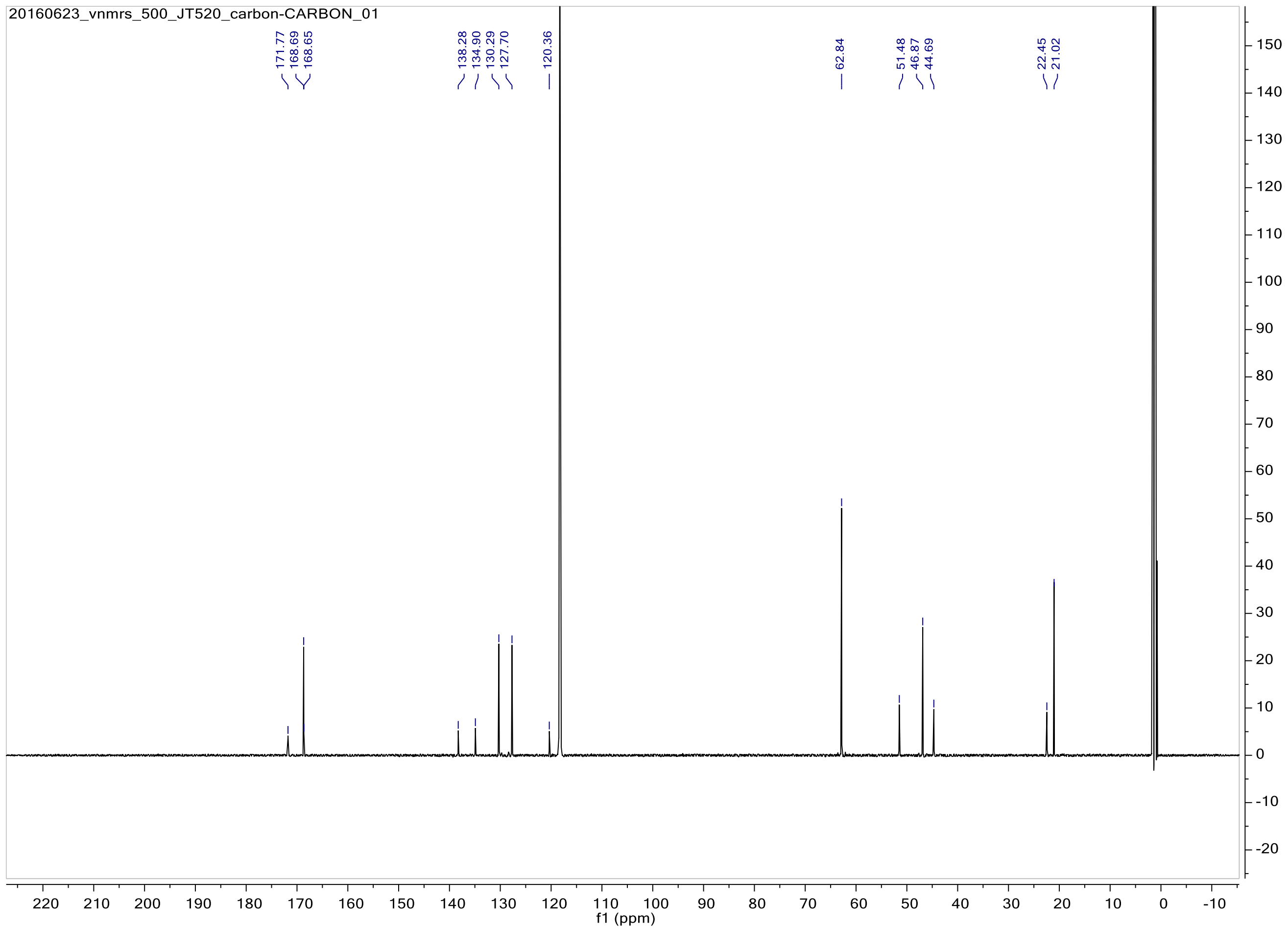

Supplementary Figure 33: ^{13}C NMR of **4d** in acetonitrile-d_3 at 25 °C.

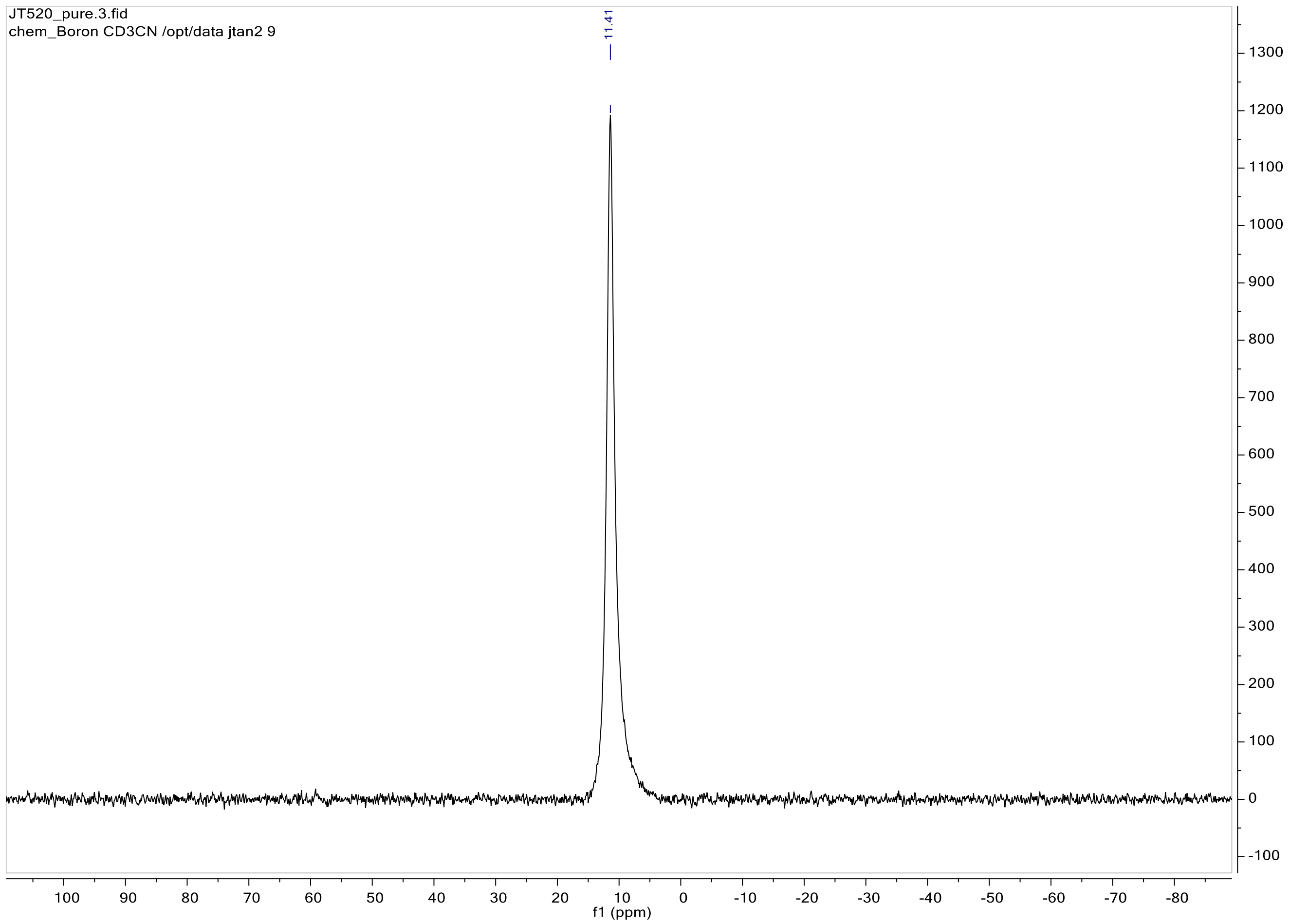

Supplementary Figure 34: ^{11}B NMR of **4d** in acetonitrile-d_3 at 25 °C.

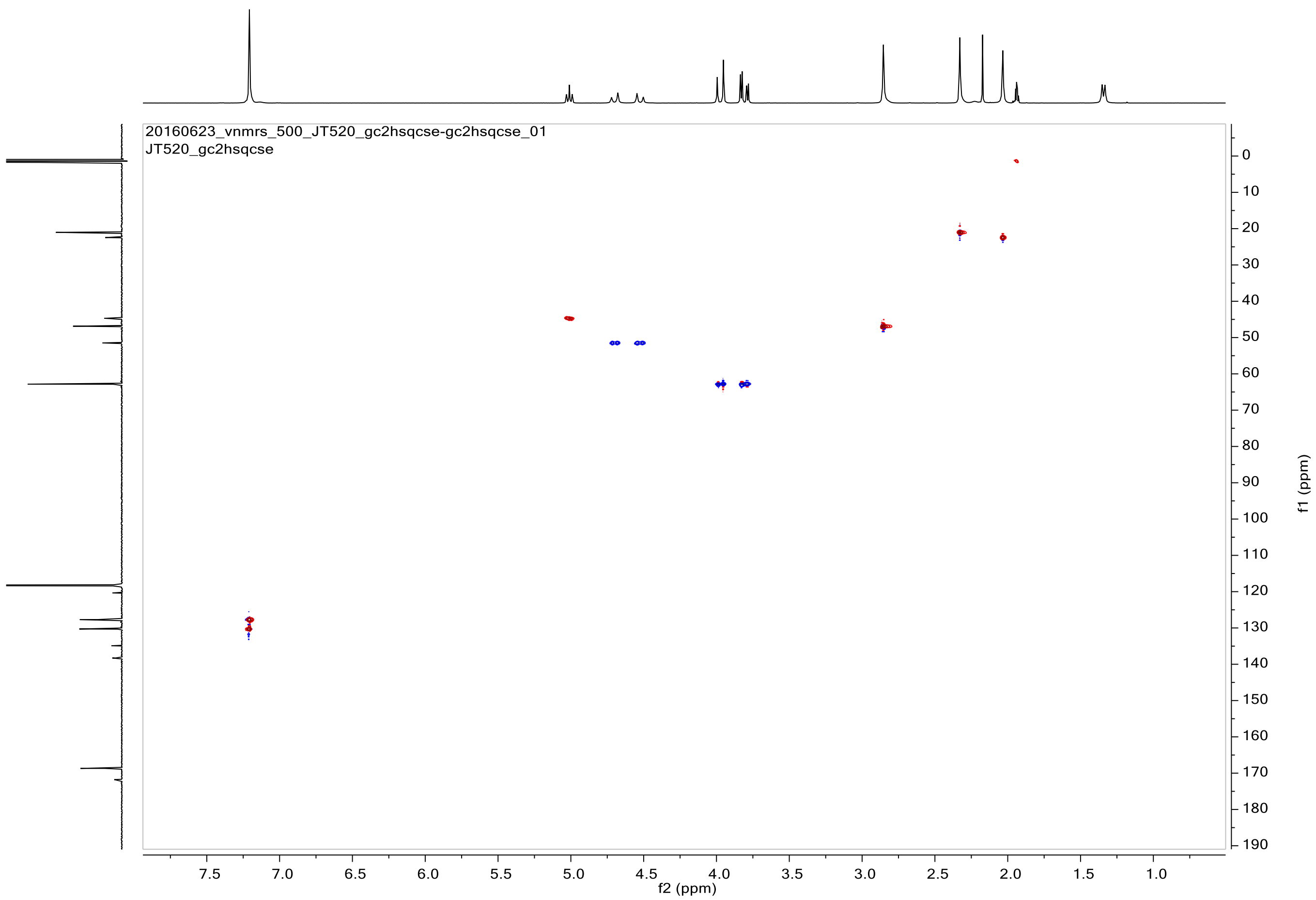

Supplementary Figure 35: ^{1}H-^{13}C HSQC NMR of **4d** in acetonitrile-d_3 at 25 °C.

Compound **4e**

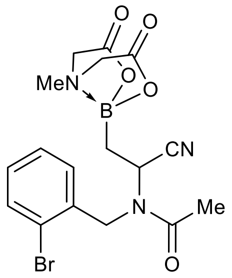

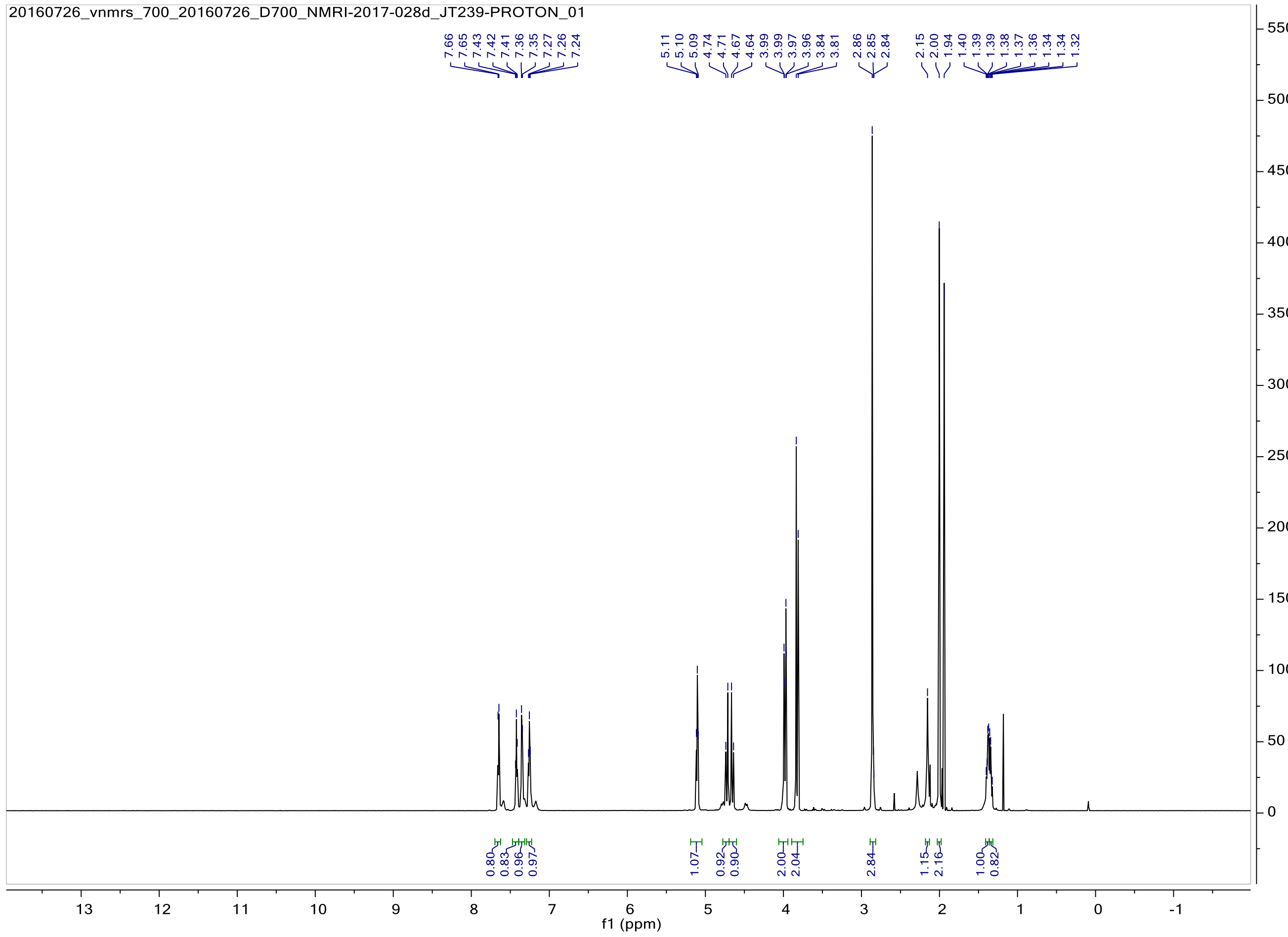

Supplementary Figure 36: ^{1}H NMR of **4e** in acetonitrile-d_3 at 25 °C.

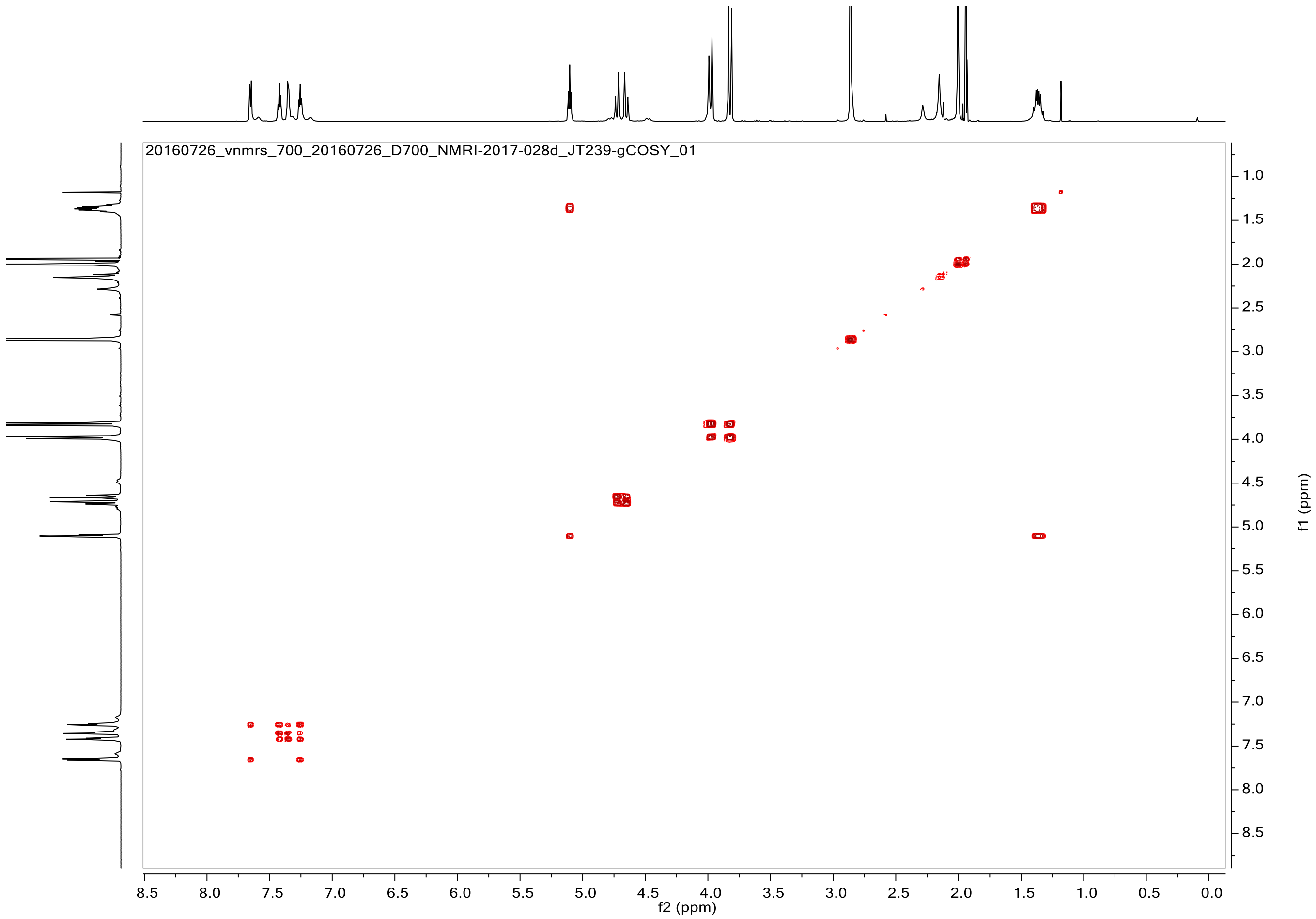

Supplementary Figure 37: ^{1}H-^{1}H COSY of **4e** in acetonitrile-d_3 at 25 °C.

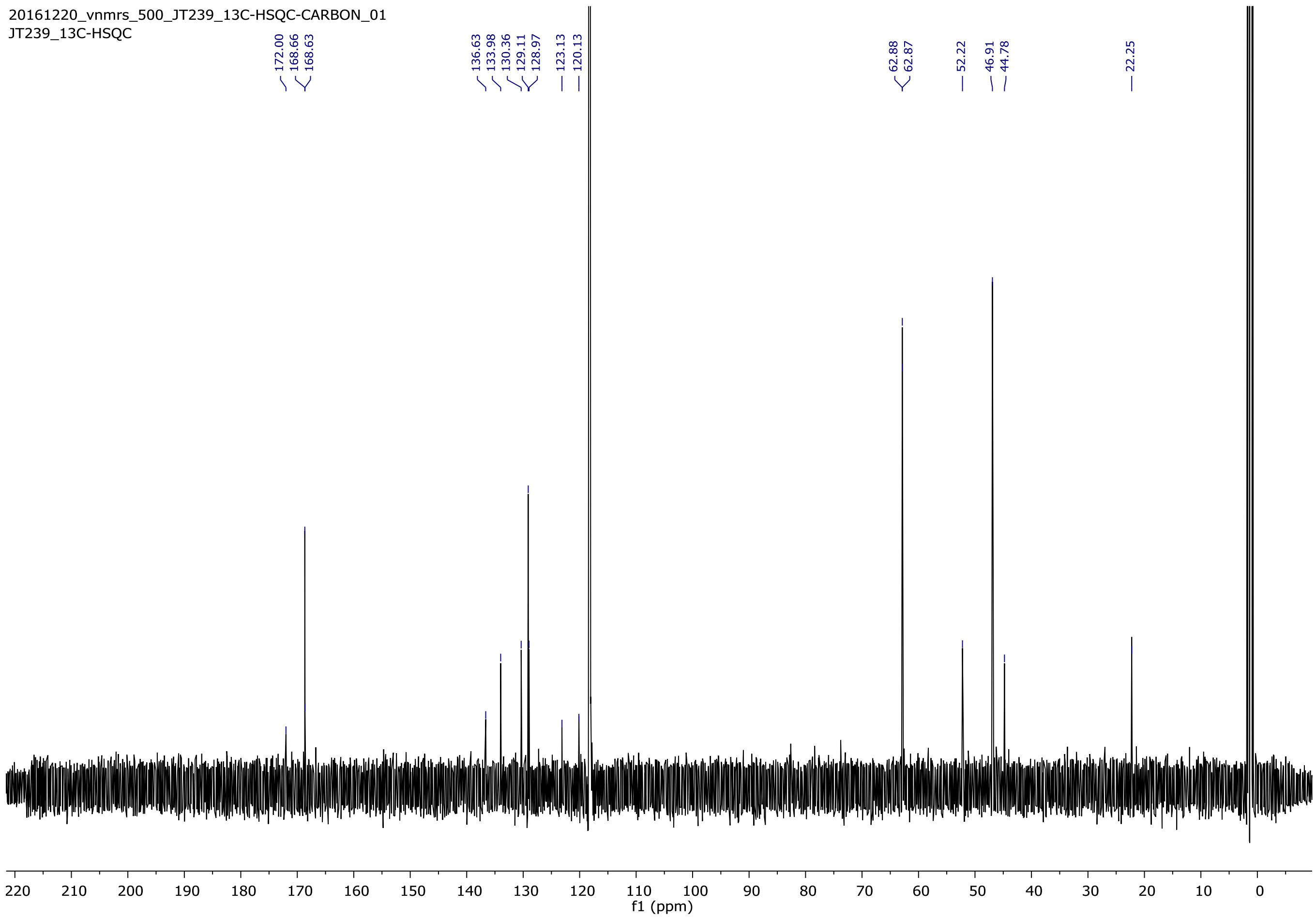

Supplementary Figure 38: ^{13}C NMR of **4e** in acetonitrile-d_3 at 25 °C.

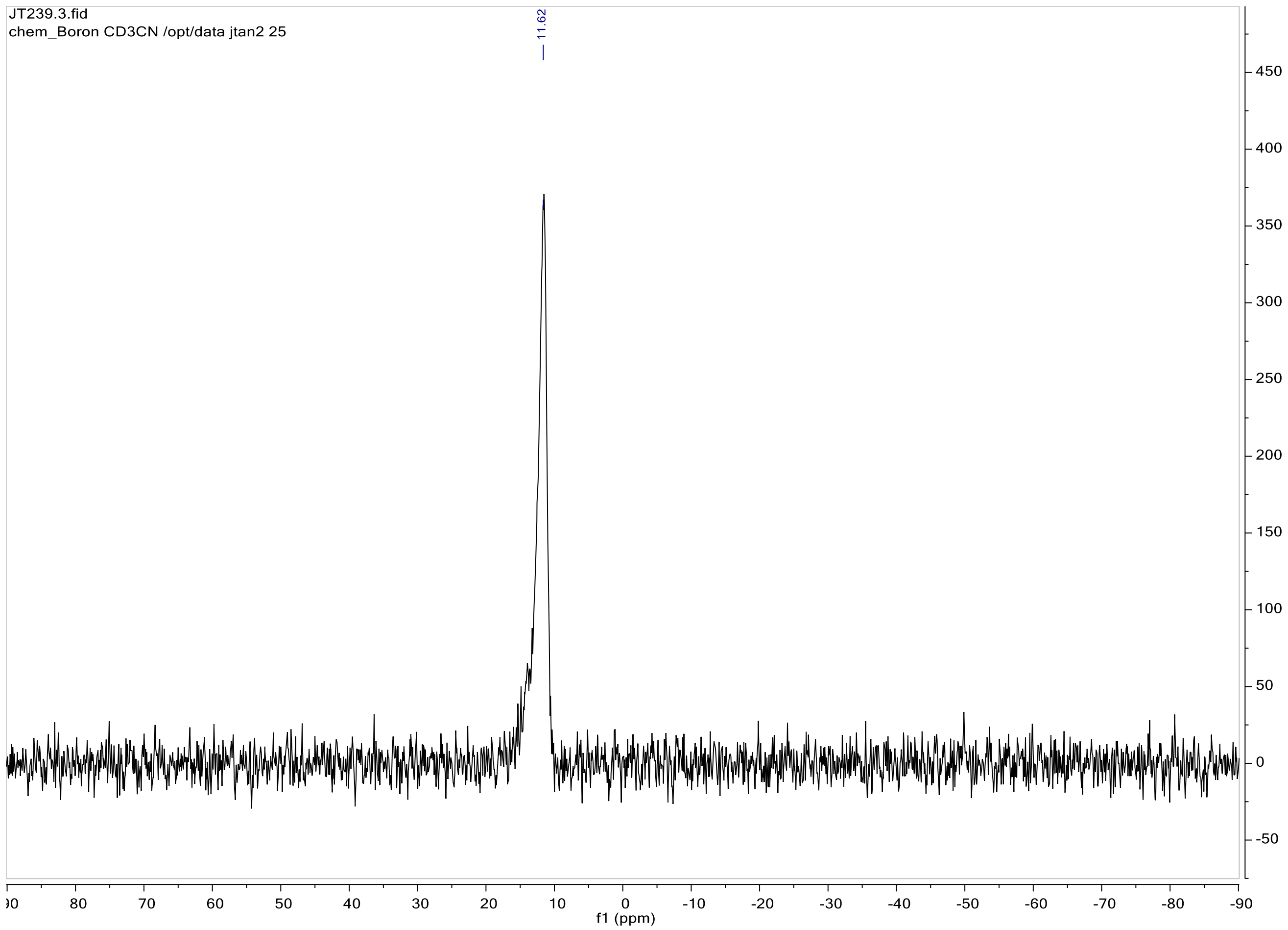

Supplementary Figure 39: ^{11}B NMR of **4e** in acetonitrile-d_3 at 25 °C.

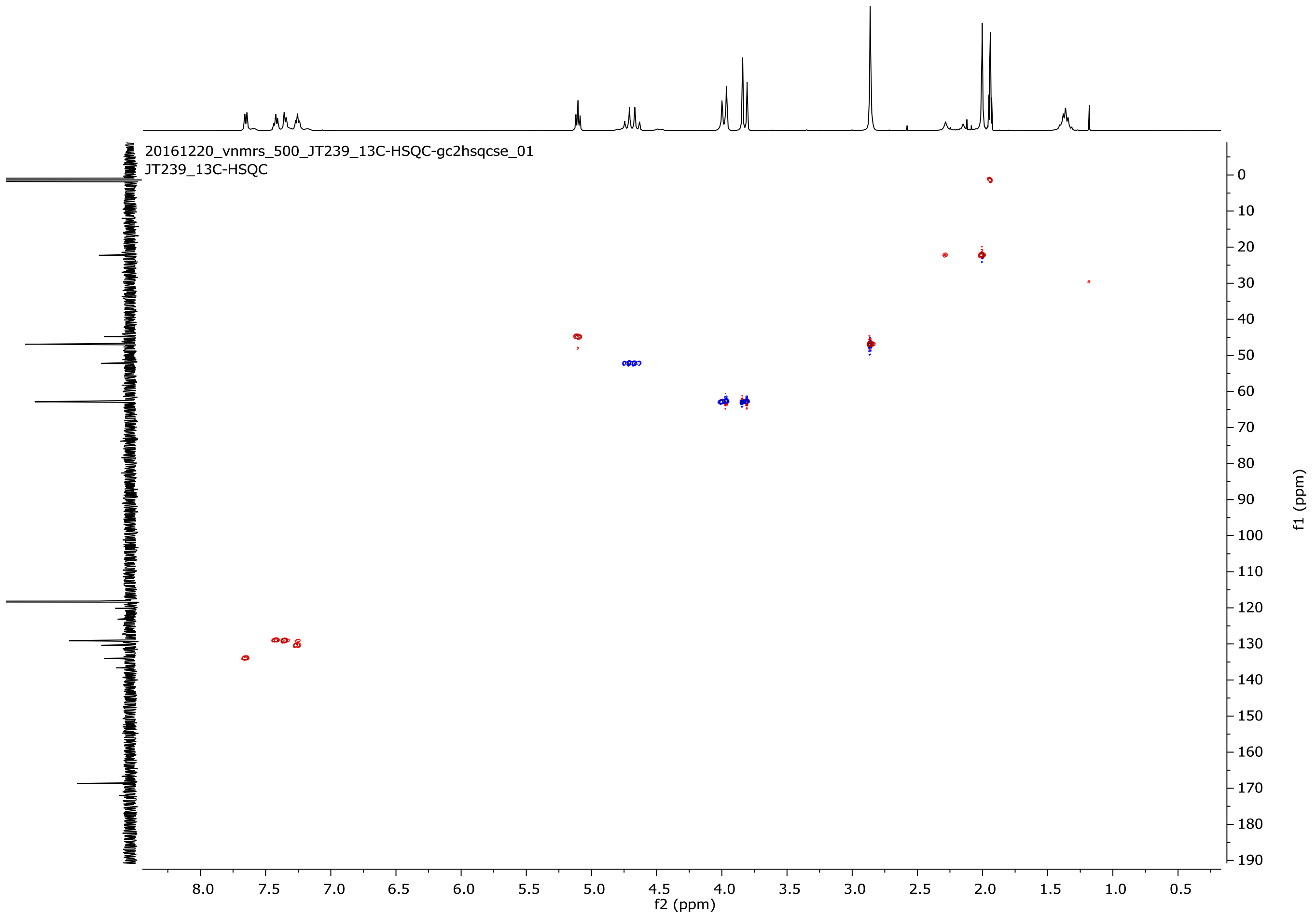

557

558 **Supplementary Figure 40:** ^{1}H-^{13}C HSQC NMR of **4e** in acetonitrile-d_3 at 25 °C.

559

Compound **4f**

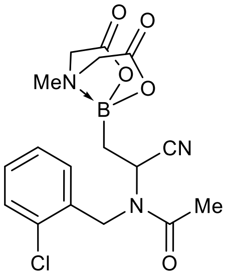

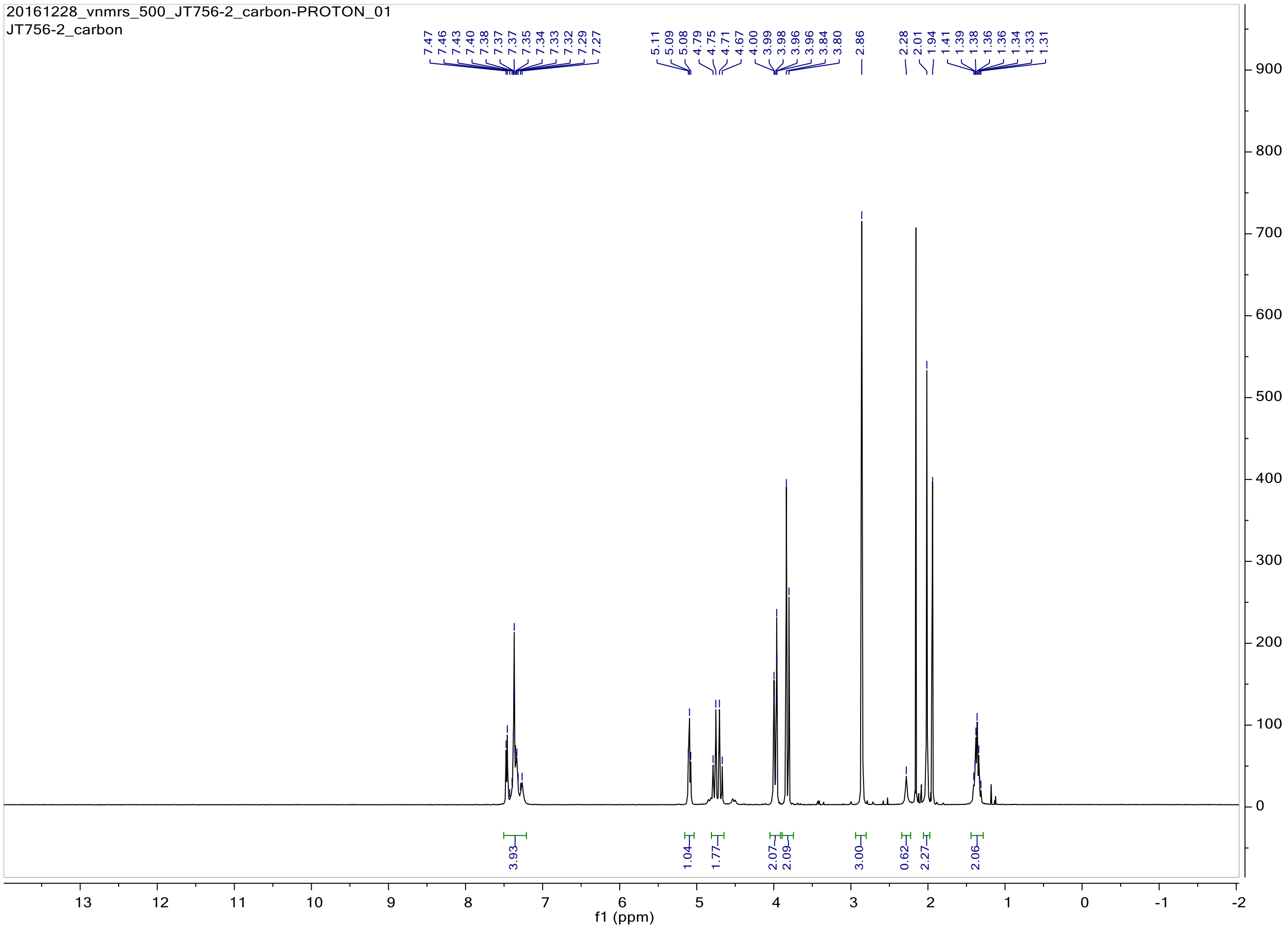

Supplementary Figure 41: ^{1}H NMR of **4f** in acetonitrile-d_3 at 25 °C.

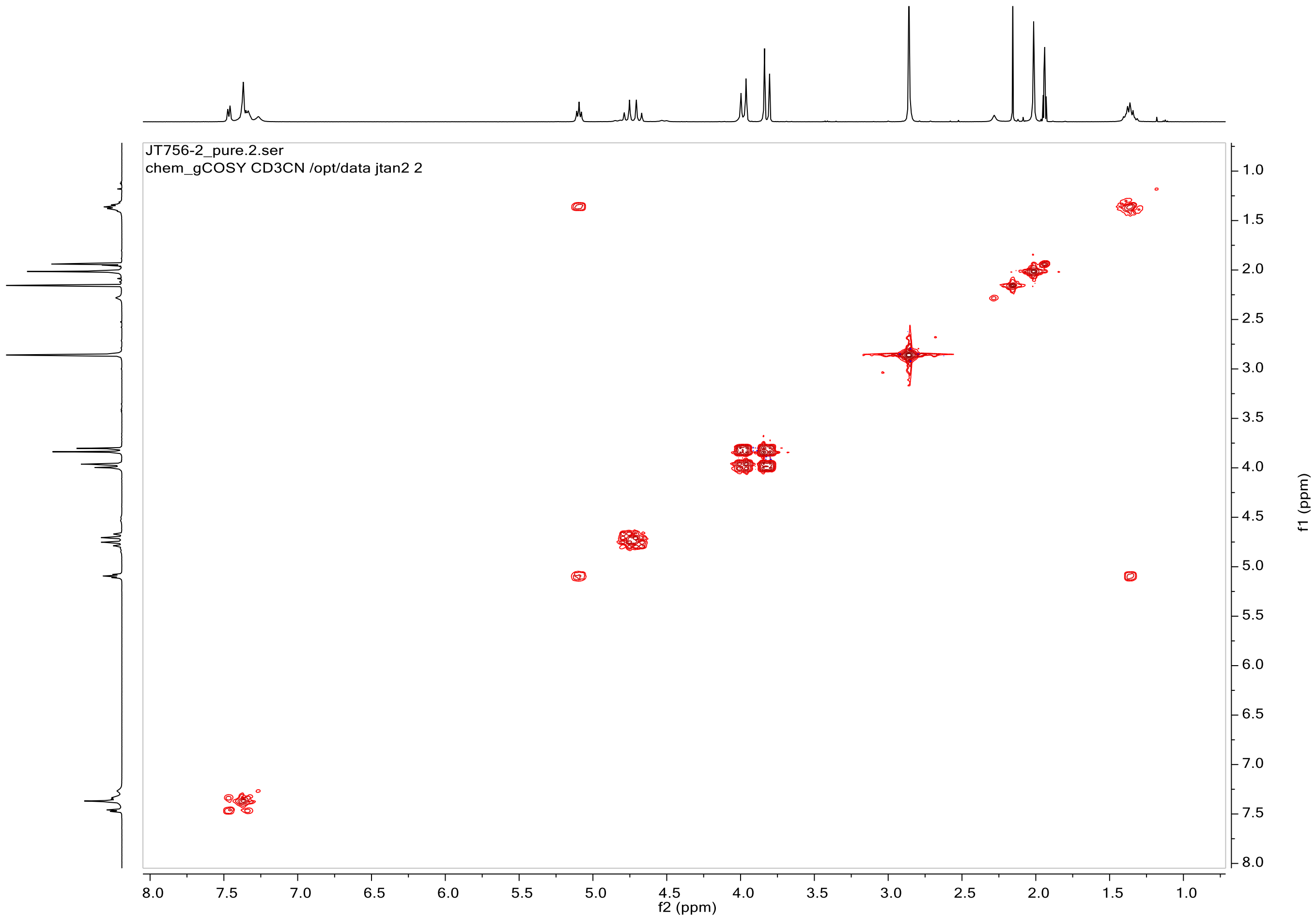

Supplementary Figure 42: ^{1}H-^{1}H COSY of **4f** in acetonitrile-d_3 at 25 °C.

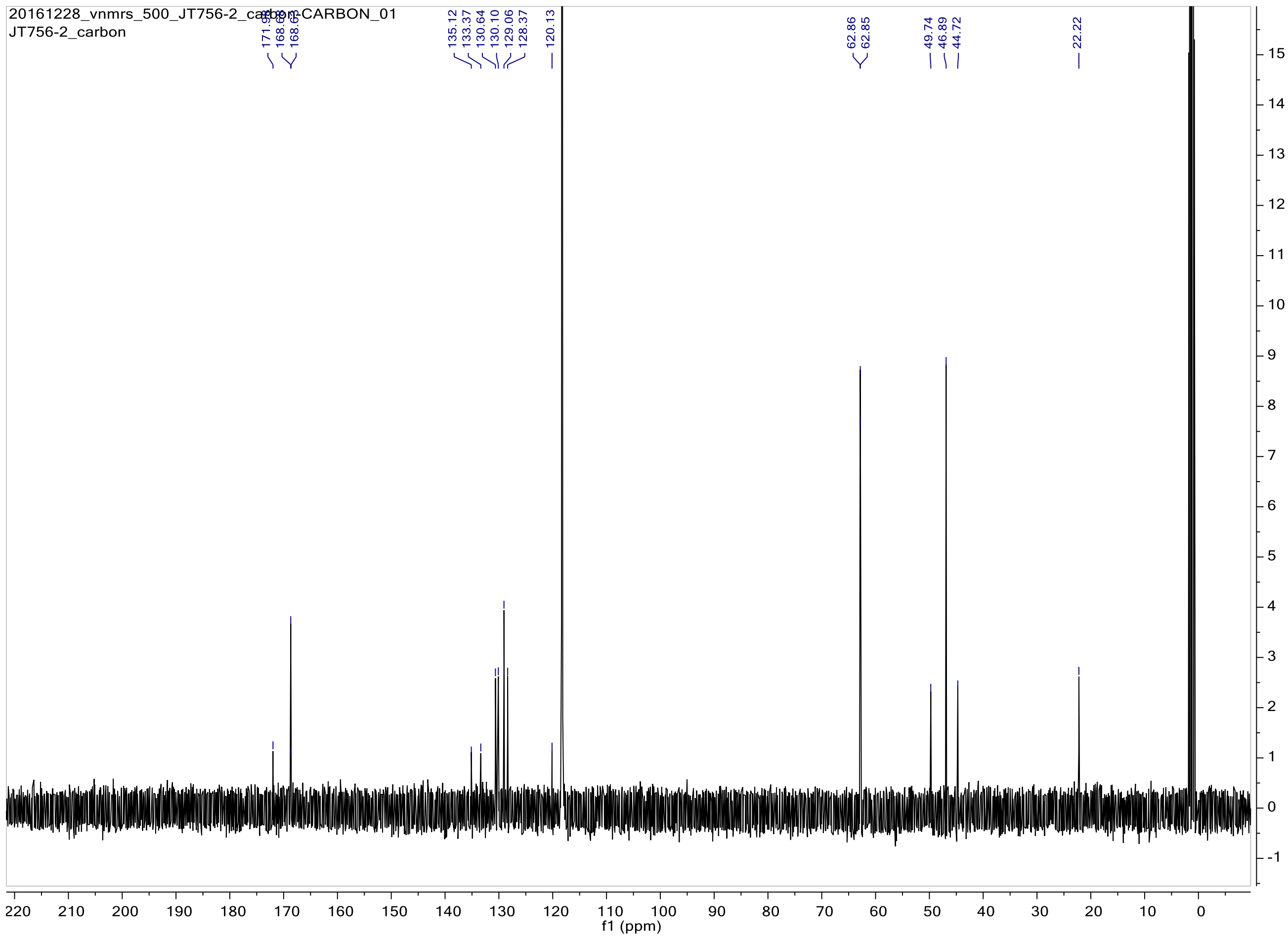

Supplementary Figure 43: ^{13}C NMR of **4f** in acetonitrile-d_3 at 25 °C.

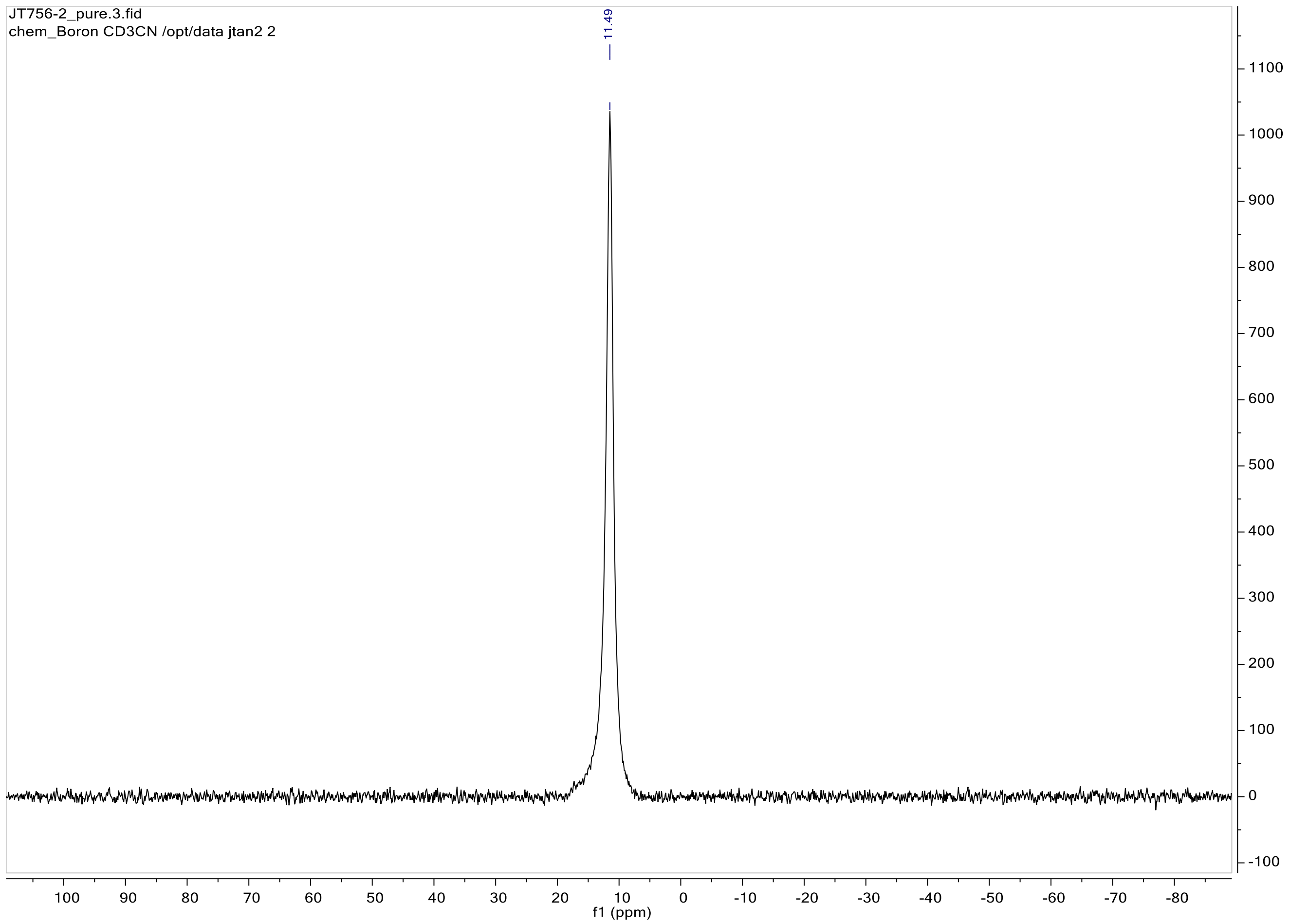

Supplementary Figure 44: ^{11}B NMR of **4f** in acetonitrile-d_3 at 25 °C.

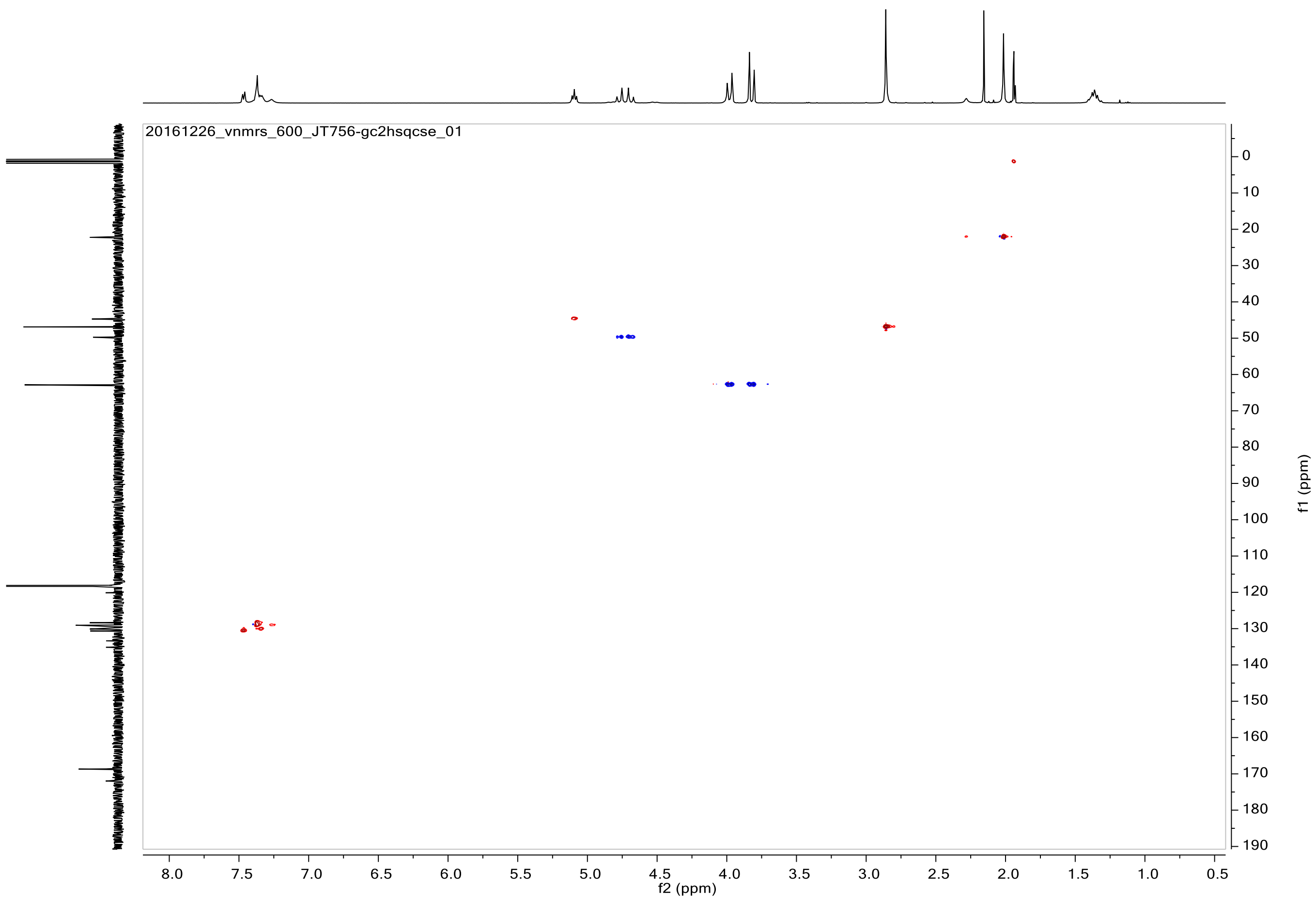

Supplementary Figure 45: ^{1}H-^{13}C HSQC NMR of **4f** in acetonitrile-d_3 at 25 °C.

Compound **4g**

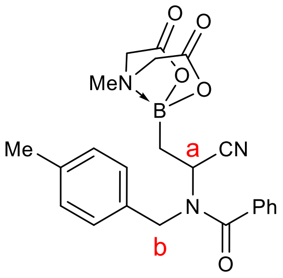

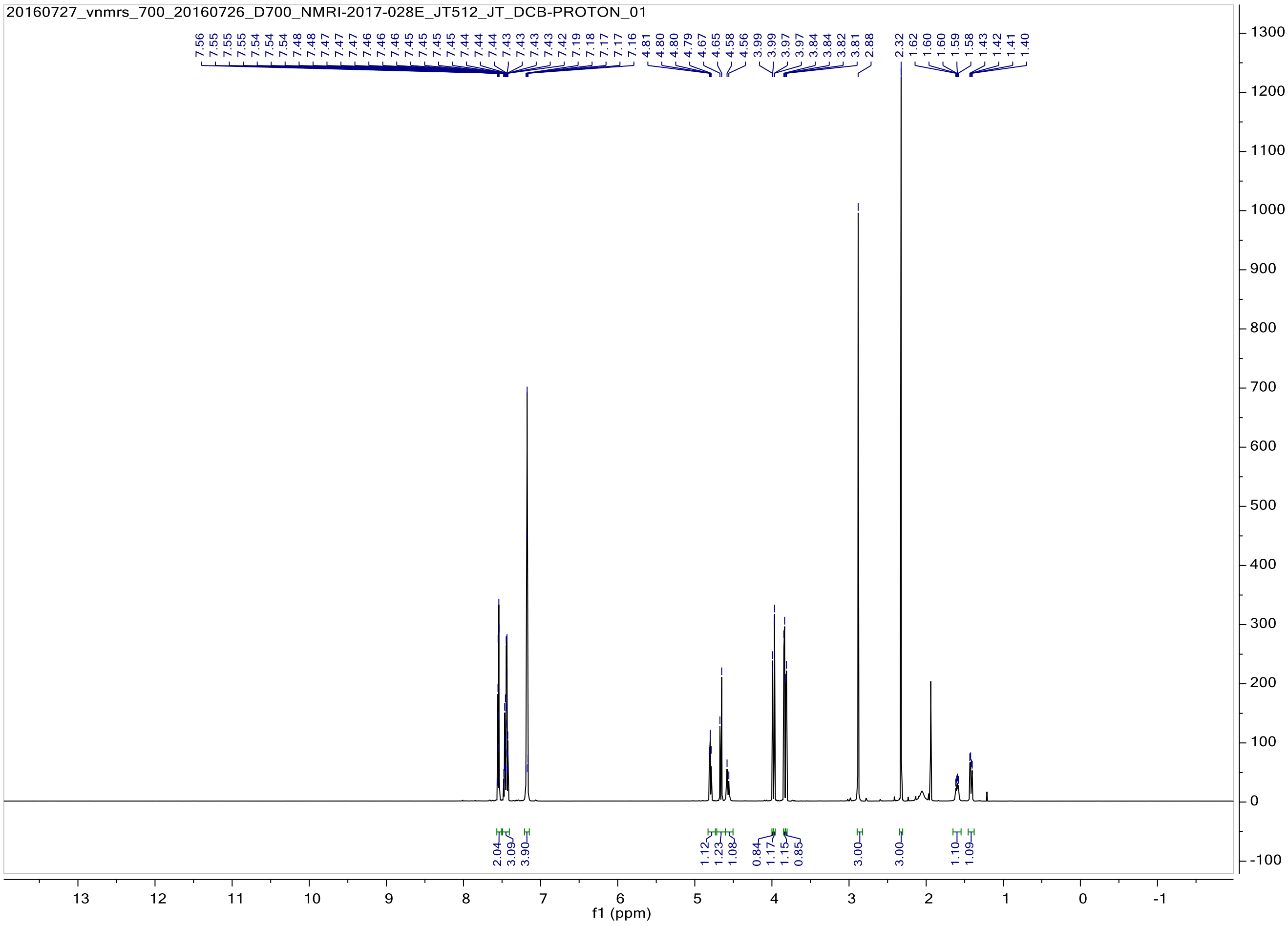

Supplementary Figure 46: ^{1}H NMR of **4g** in acetonitrile-d_3 at 60 °C.

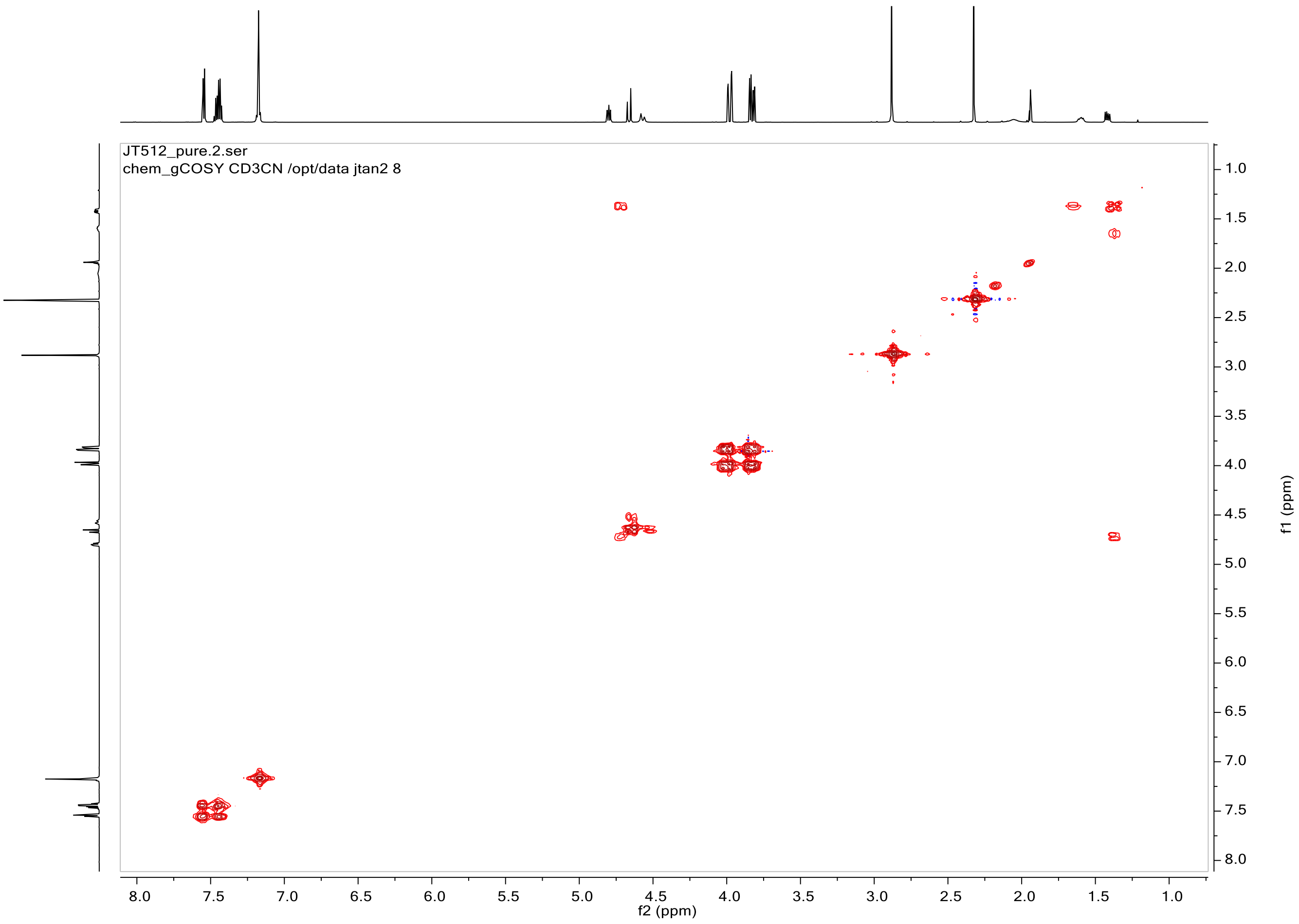

Supplementary Figure 47: ^{1}H-^{1}H COSY of **4g** in acetonitrile-d_3 at 25 °C.

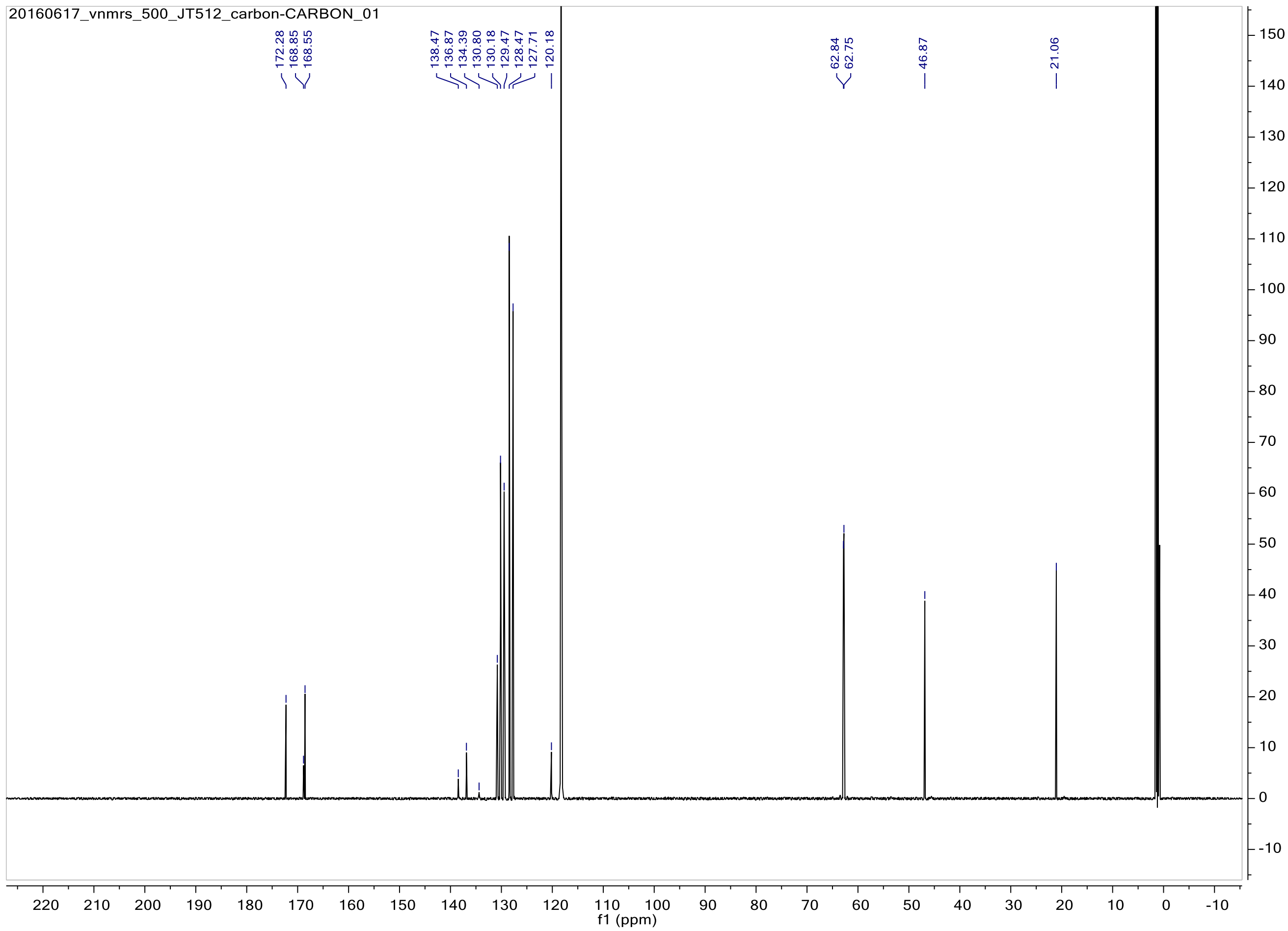

Supplementary Figure 48: ^{13}C NMR of **4g** in acetonitrile-d_3 at 25 °C.

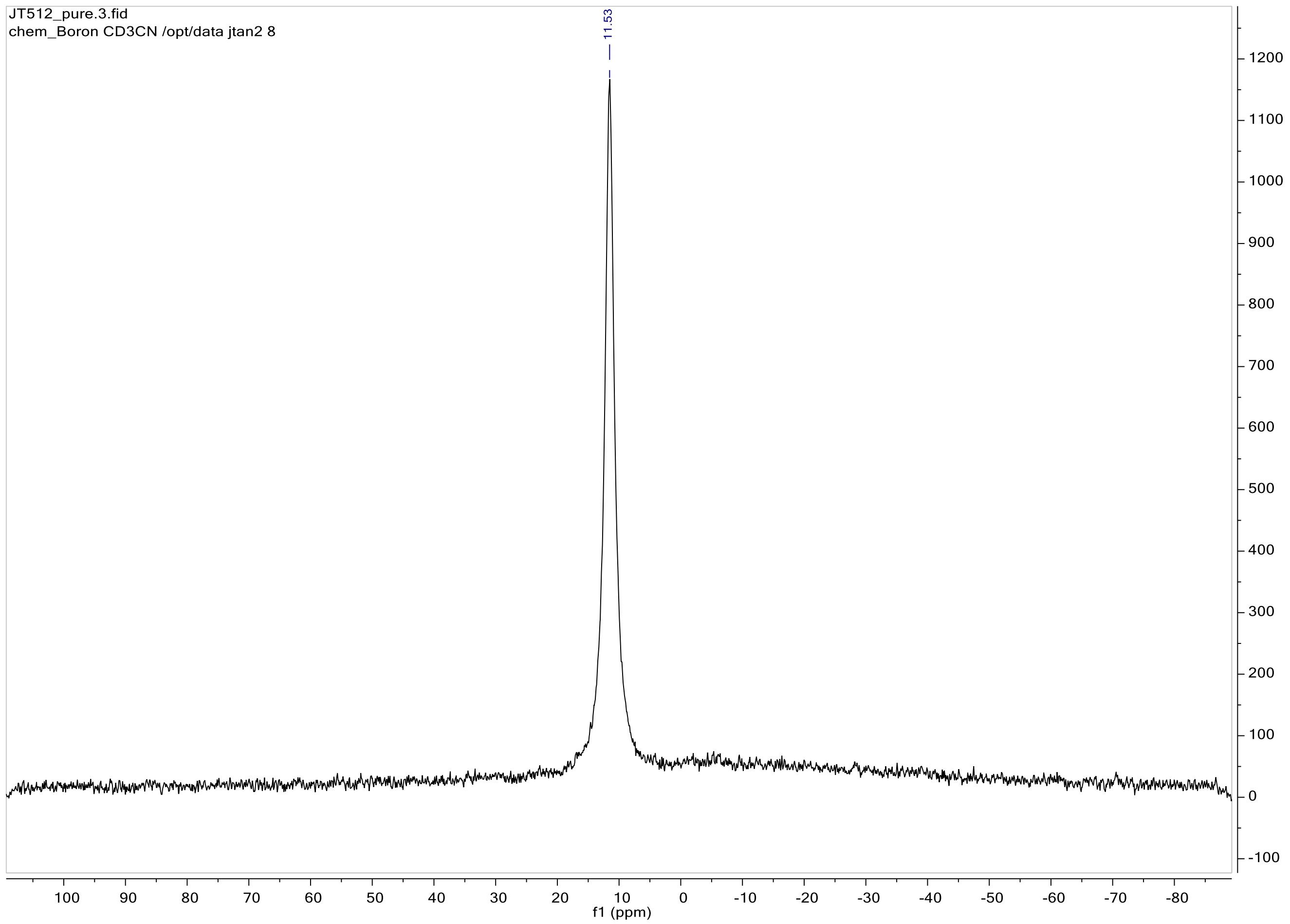

Supplementary Figure 49: ^{11}B NMR of **4g** in acetonitrile-*d*$_3$ at 25 °C.

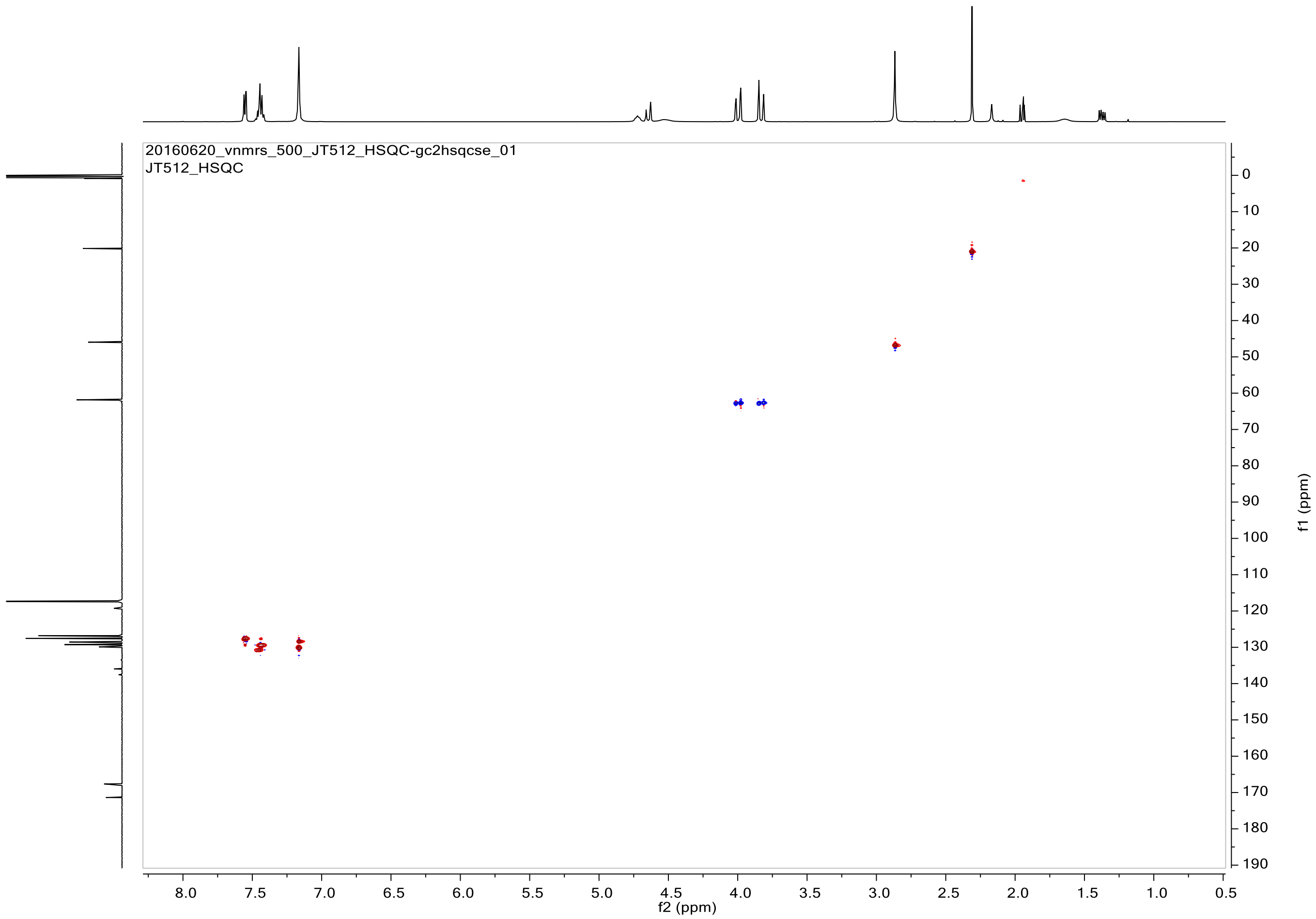

Supplementary Figure 50: ^{1}H-^{13}C HSQC NMR of **4g** in acetonitrile-d_3 at 25 °C.

Compound **4h**

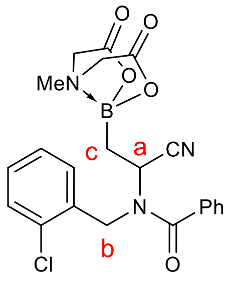

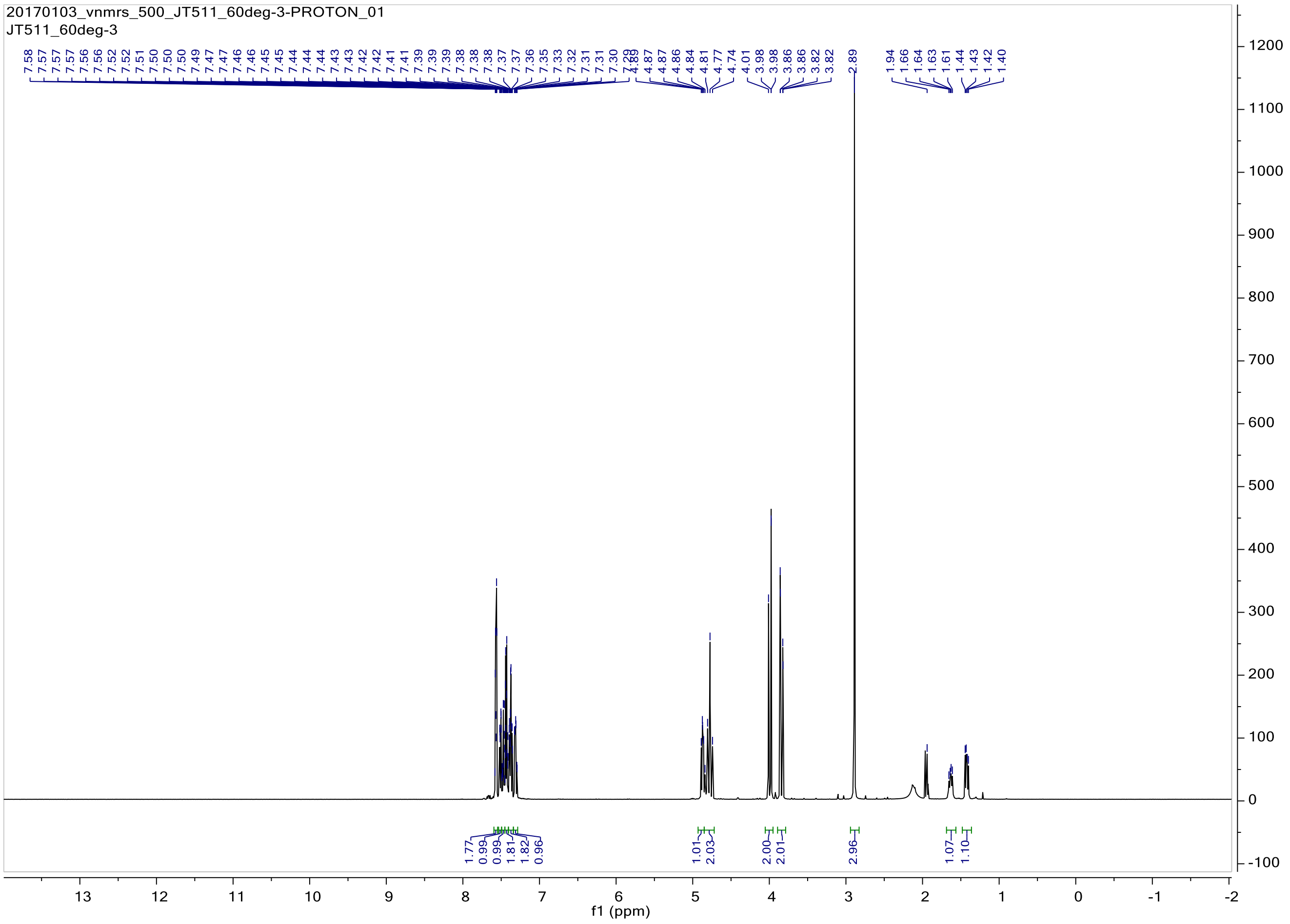

Supplementary Figure 51: ^{1}H NMR of 4h in acetonitrile-d_3 at 60 °C.

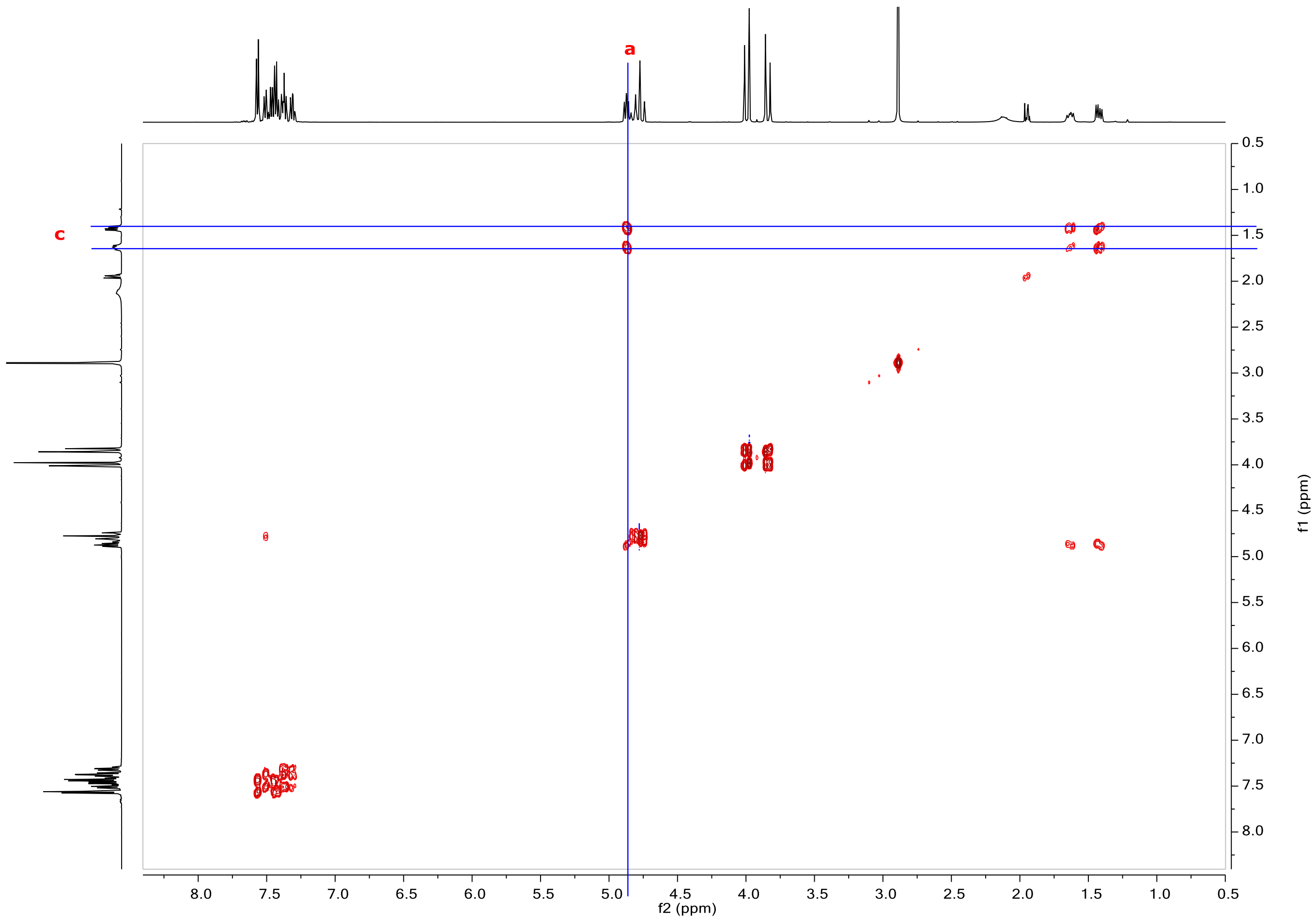

Supplementary Figure 52: ^{1}H-^{1}H COSY of **4h** in acetonitrile-d_3 at 60 °C.

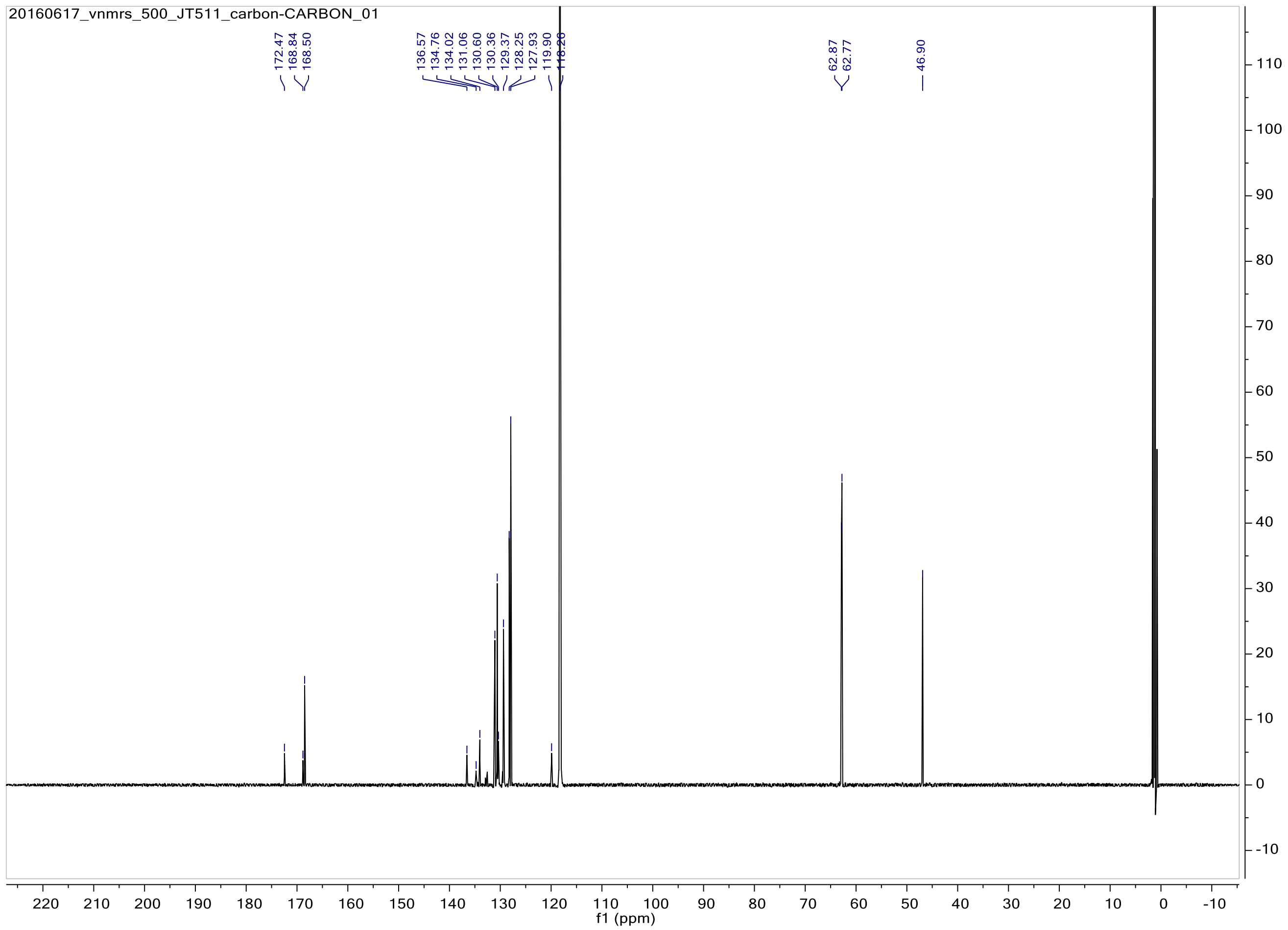

Supplementary Figure 53: ^{13}C NMR of **4h** in acetonitrile-d_3 at 25 °C.

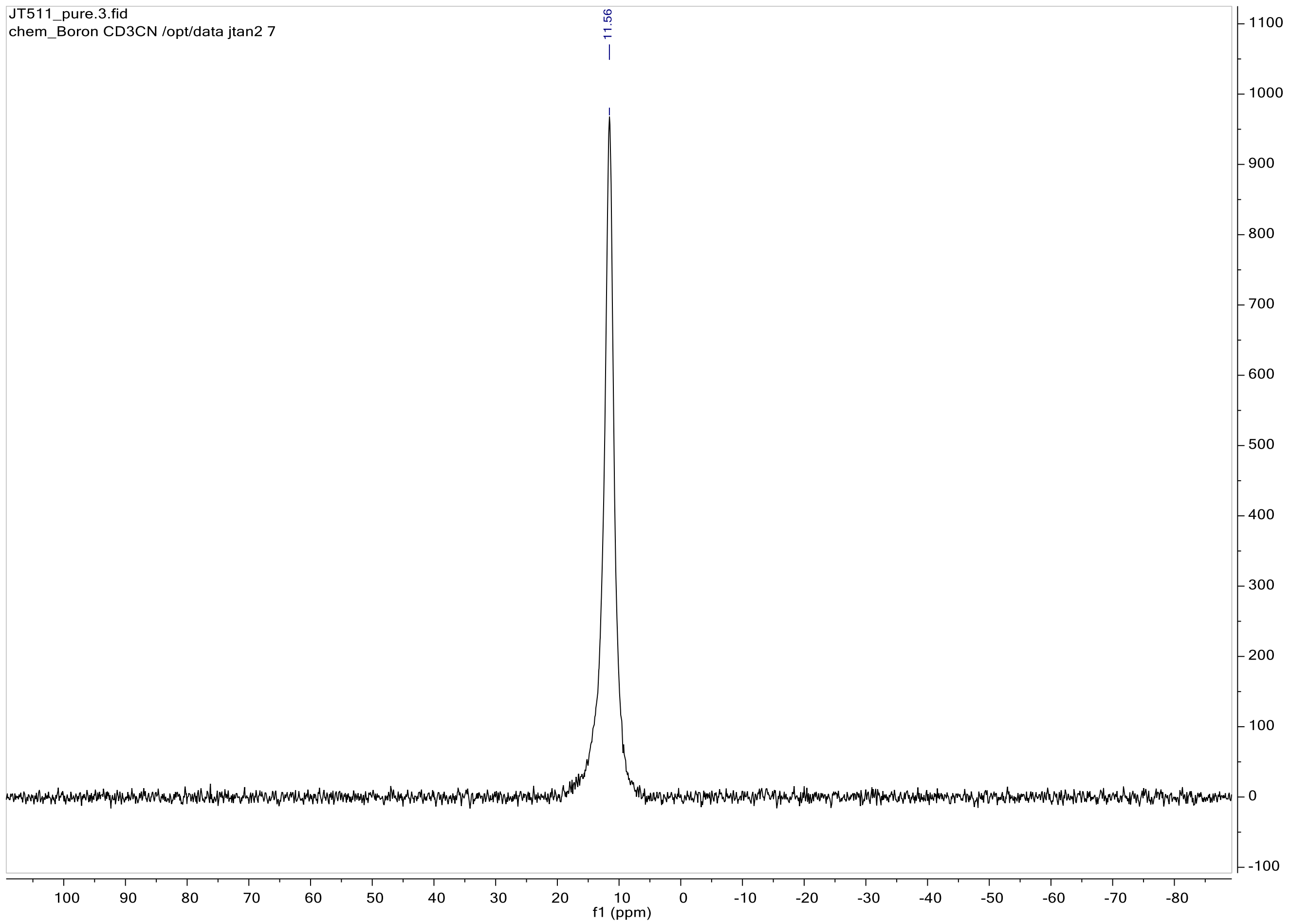

Supplementary Figure 54: ^{11}B NMR of **4h** in acetonitrile-d_3 at 25 °C.

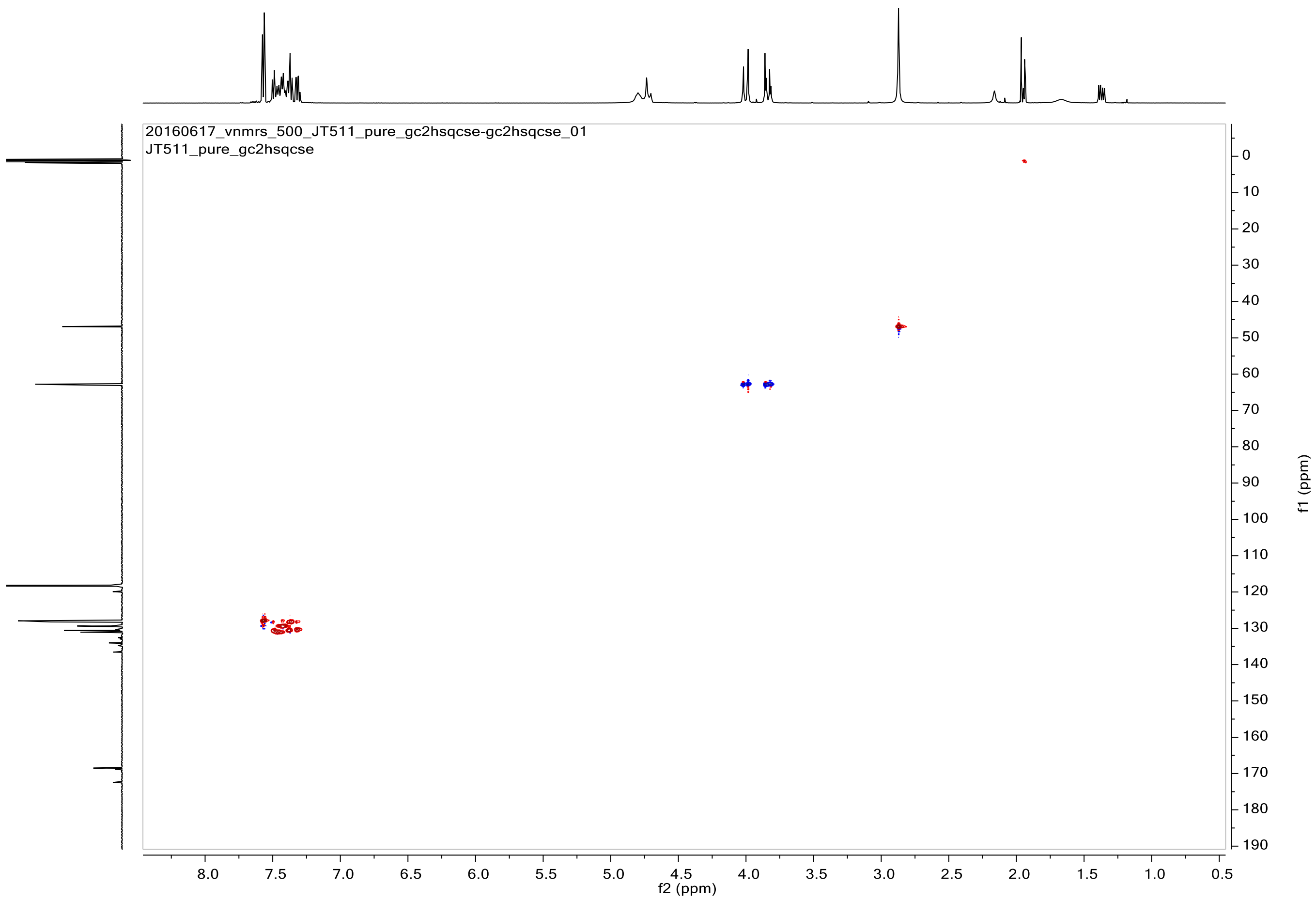

Supplementary Figure 55: ^{1}H-^{13}C HSQC NMR of **4h** in acetonitrile-d_3 at 25 °C.

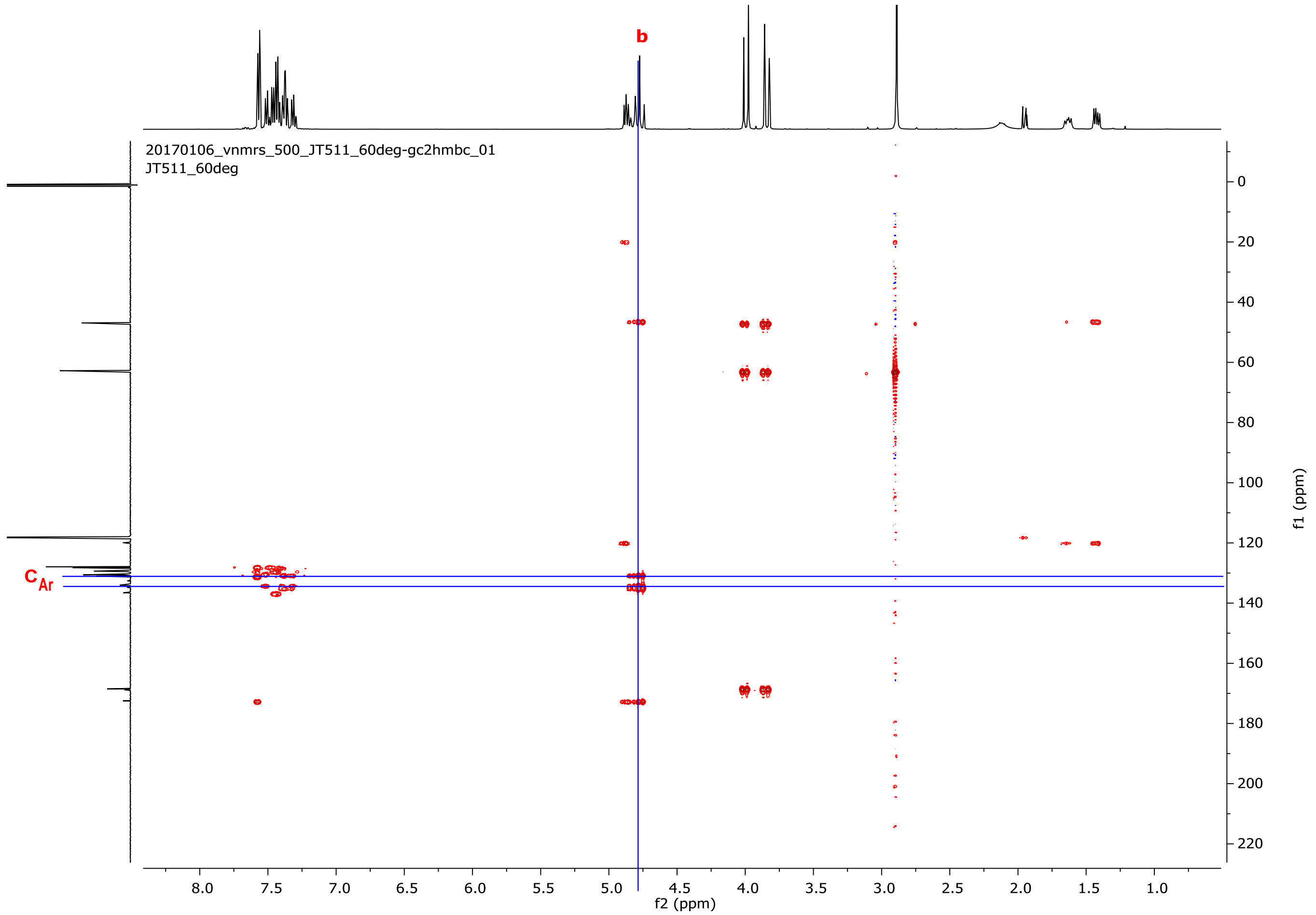

609 **Supplementary Figure 56:** ^{1}H-^{13}C HMBC NMR of **4h** in acetonitrile-d_3 at 60 °C.

Compound **4i**

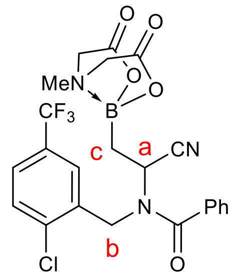

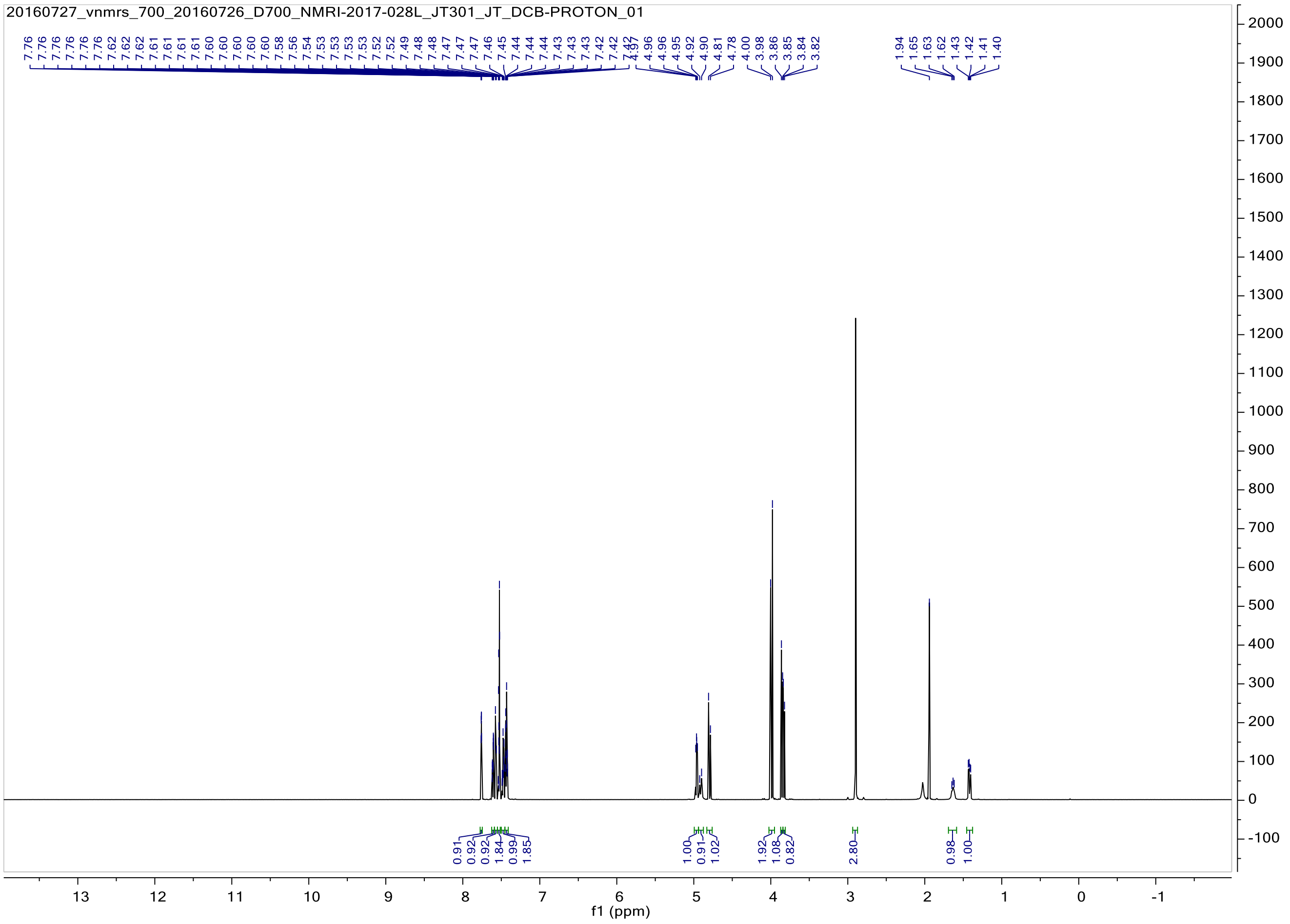

Supplementary Figure 57: ^{1}H NMR of **4i** in acetonitrile-d_3 at 60 °C.

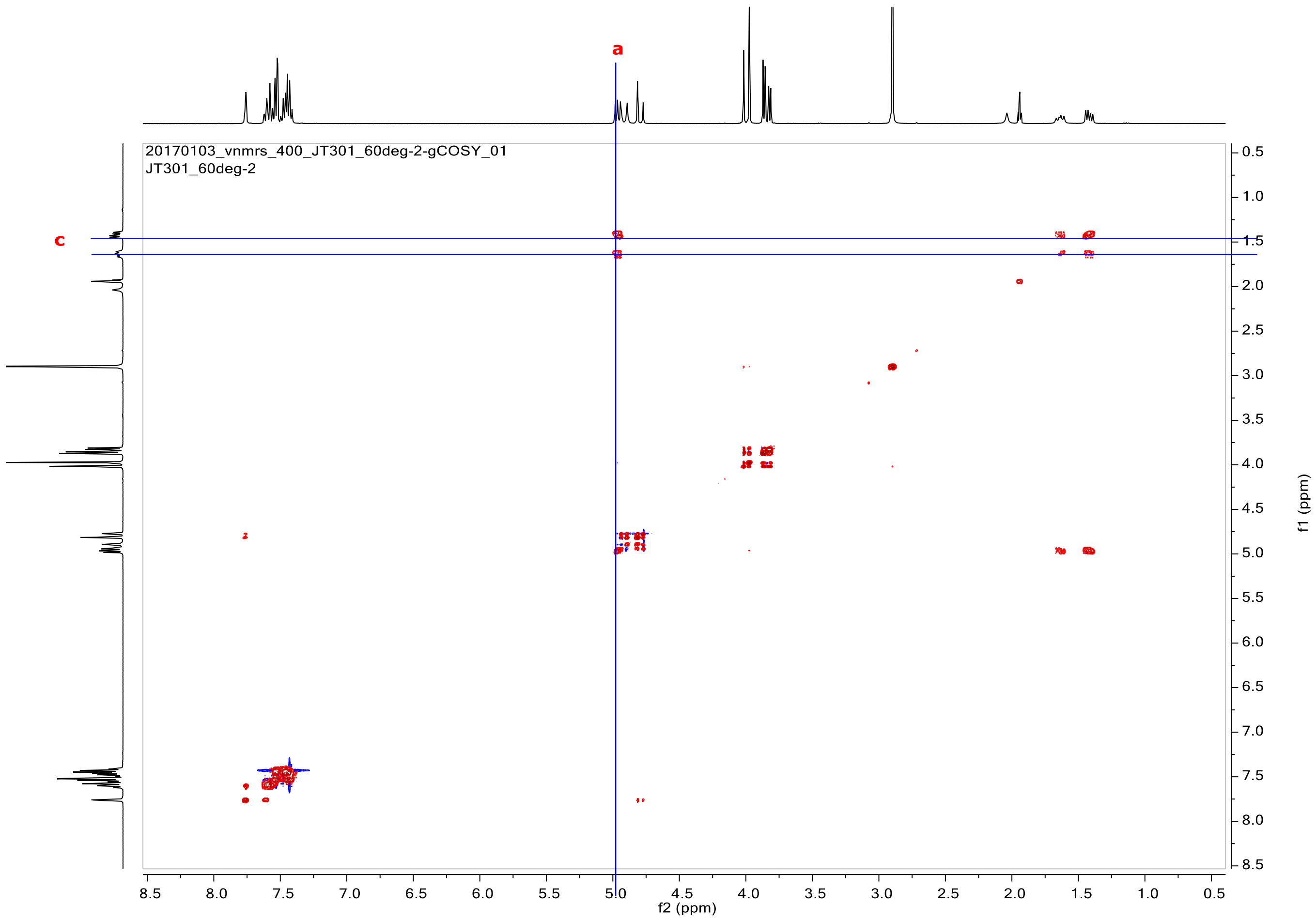

Supplementary Figure 58: ^{1}H-^{1}H COSY of **4i** in acetonitrile-d_3 at 60 °C.

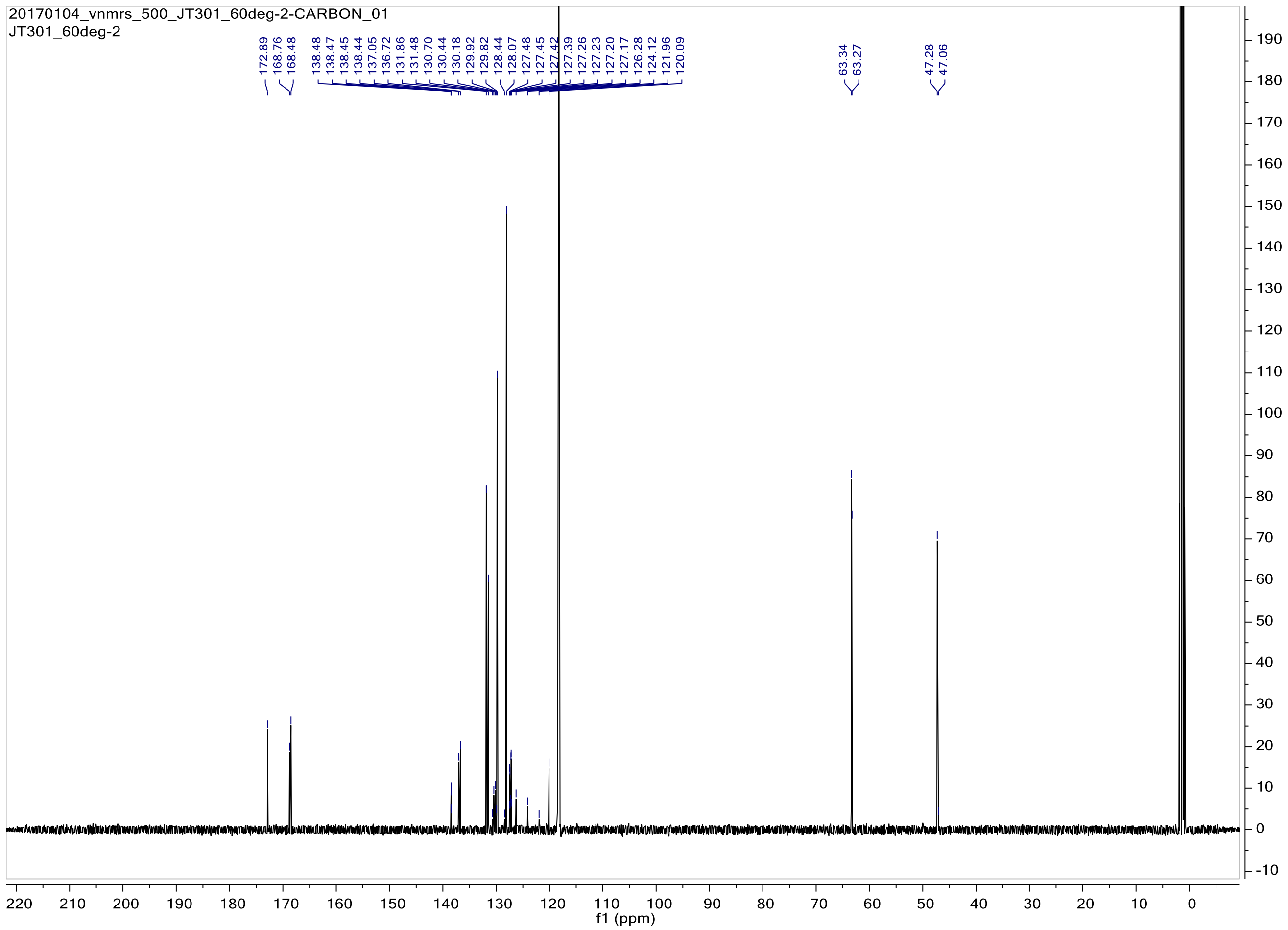

Supplementary Figure 59: ^{13}C NMR of **4i** in acetonitrile-*d_3* at 60 °C.

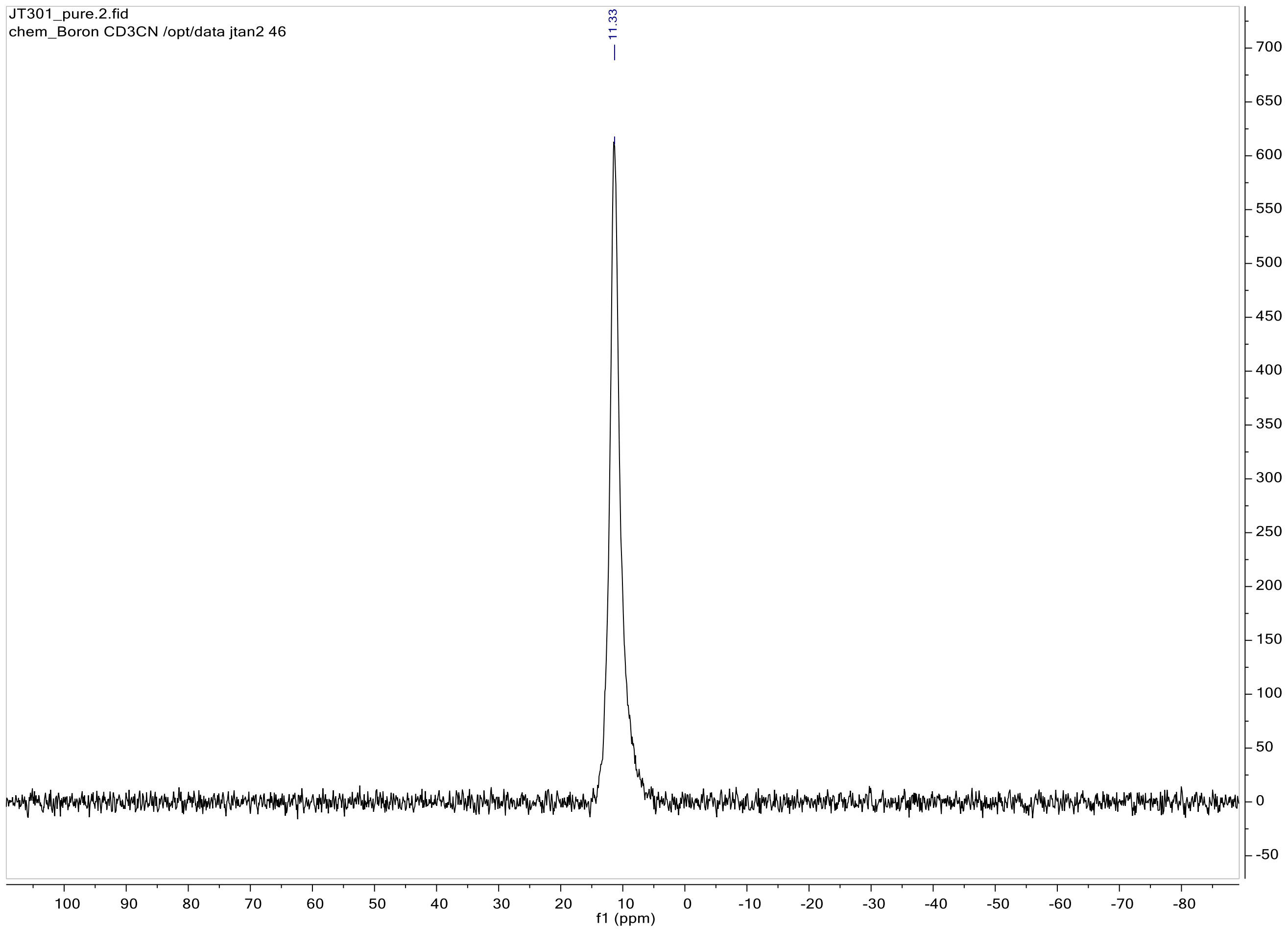

Supplementary Figure 60: ^{11}B NMR of **4i** in acetonitrile-d_3 at 25 °C.

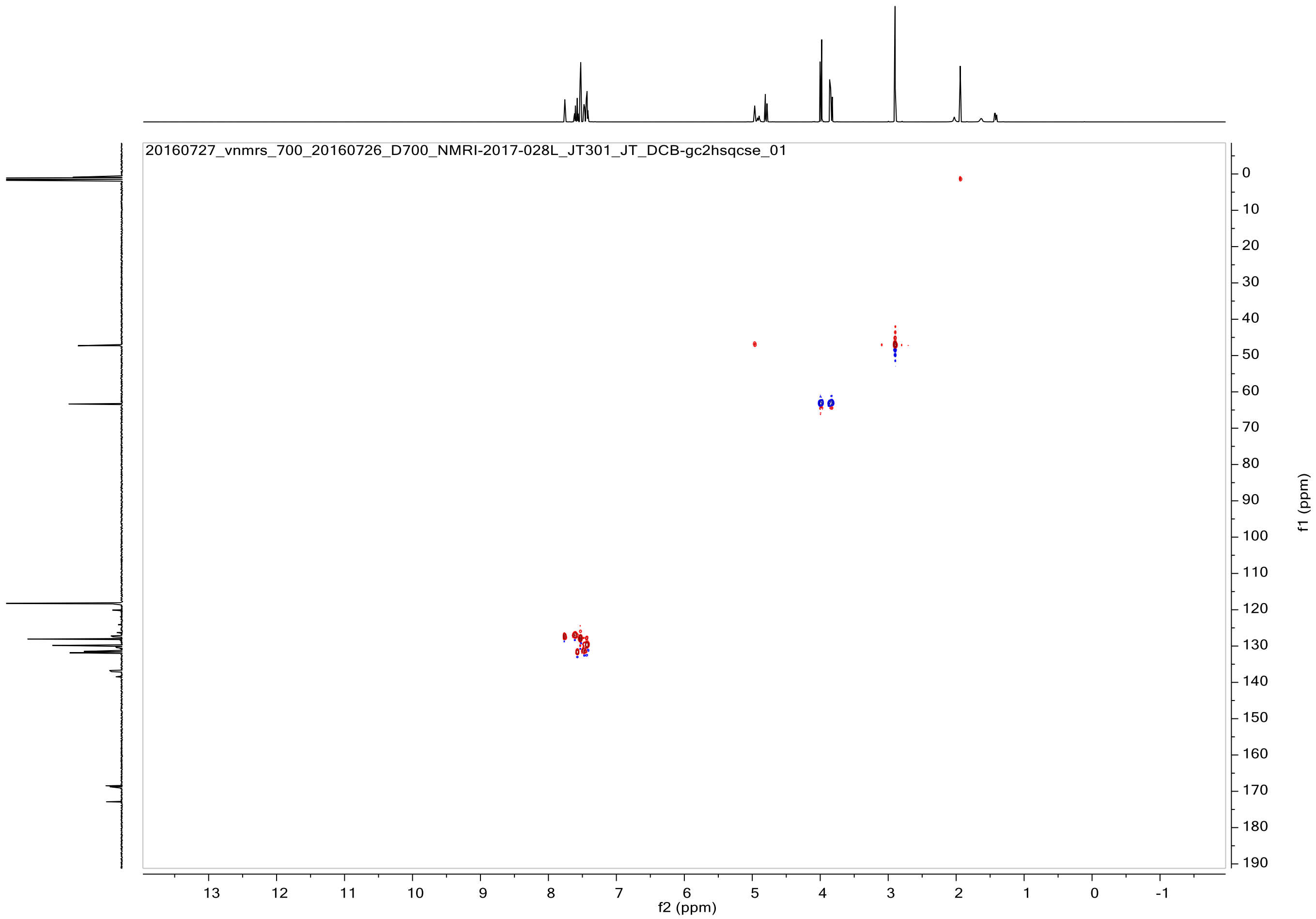

Supplementary Figure 61: ^{1}H-^{13}C HSQC NMR of **4i** in acetonitrile-d_3 at 60 °C.

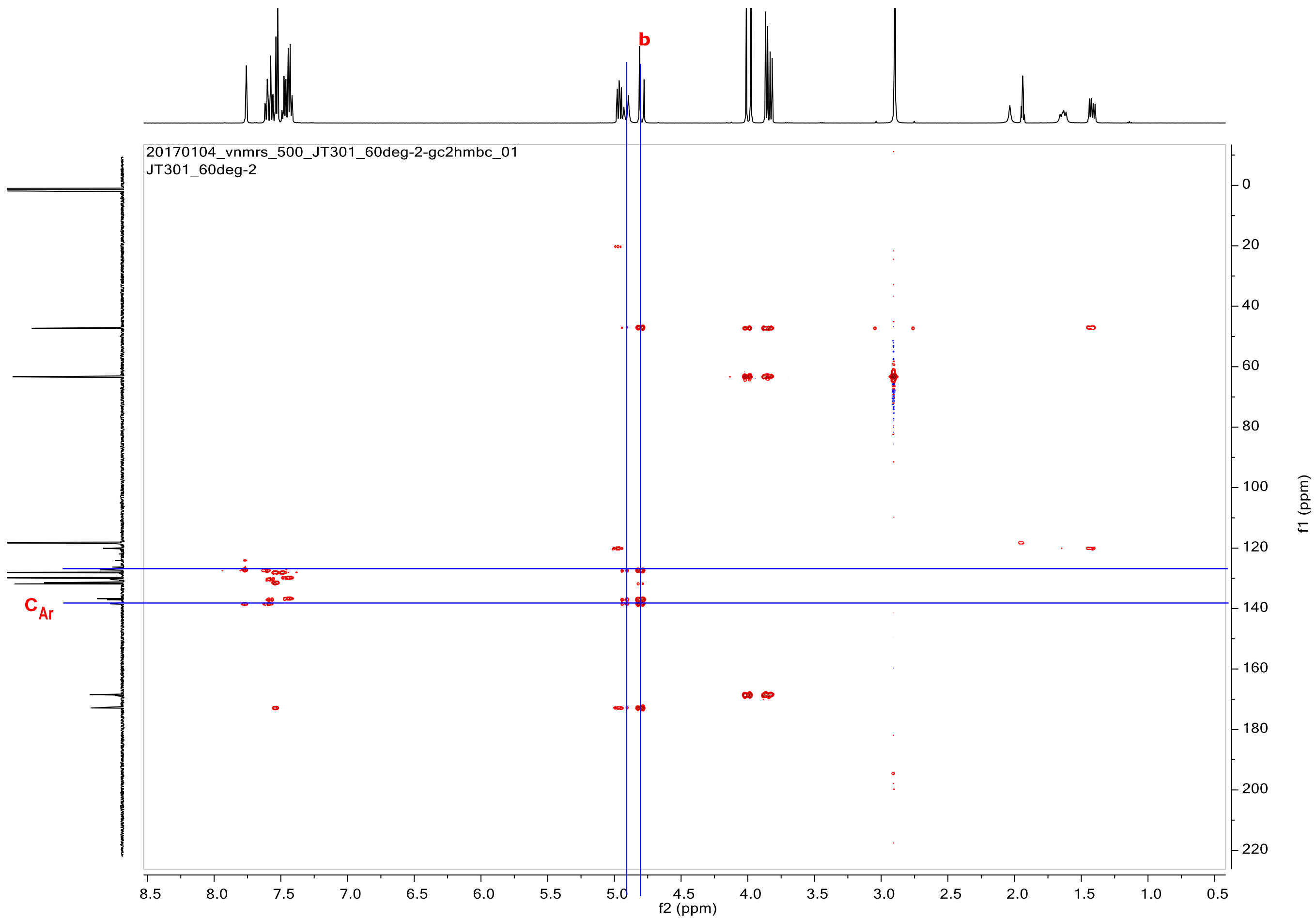

Supplementary Figure 62: ^{1}H-^{13}C HMBC NMR of **4i** in acetonitrile-d_3 at 60 °C.

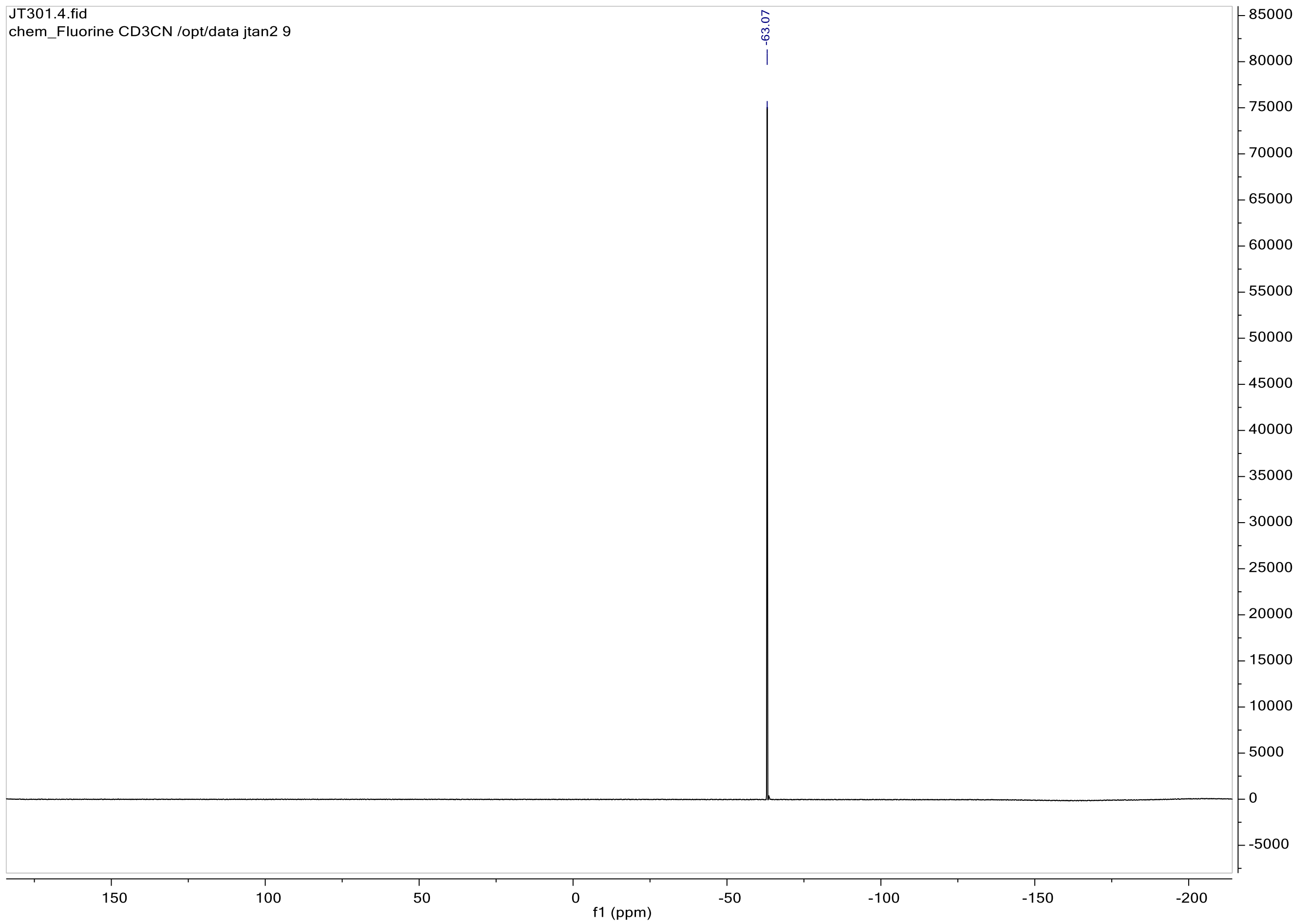

630

631 **Supplementary Figure 63:** ^{19}F NMR of **4i** in acetonitrile-*d*$_3$ at 25 °C.

Compound **4j**

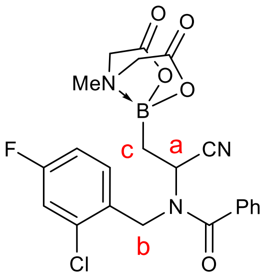

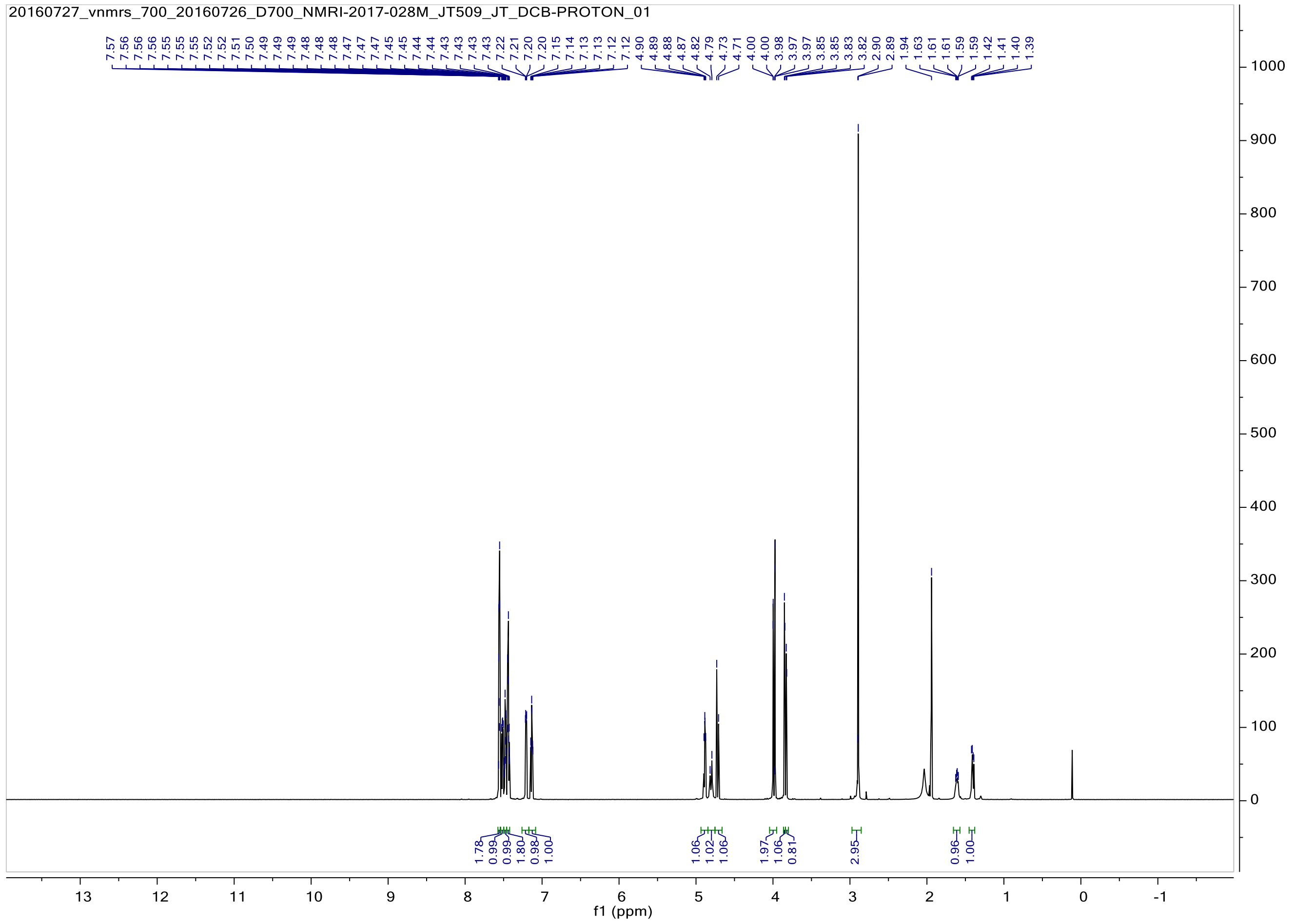

Supplementary Figure 64: ^{1}H NMR of **4j** in acetonitrile-d_3 at 60 °C.

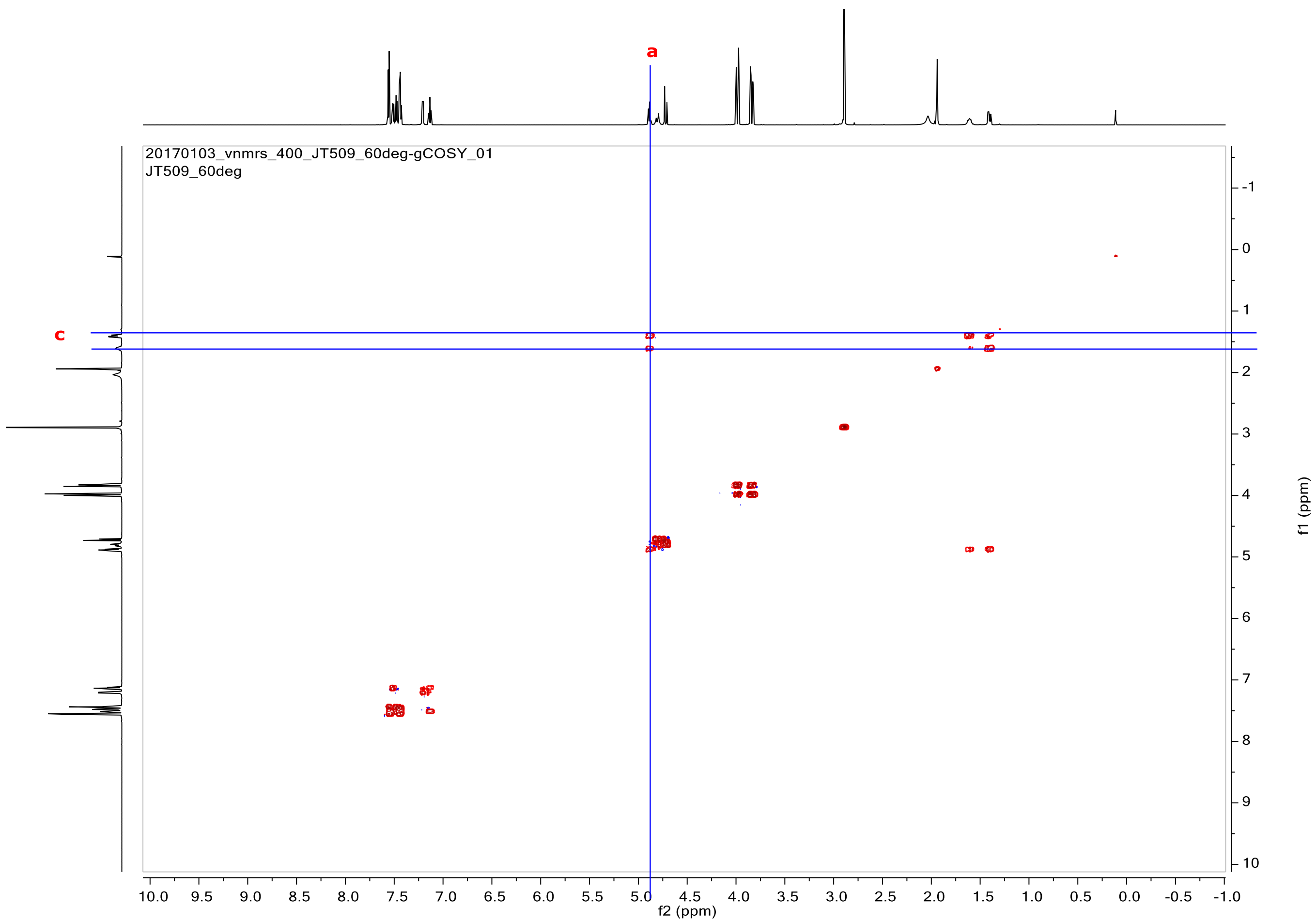

Supplementary Figure 65: ^{1}H-^{1}H COSY of **4j** in acetonitrile-d_3 at 60 °C.

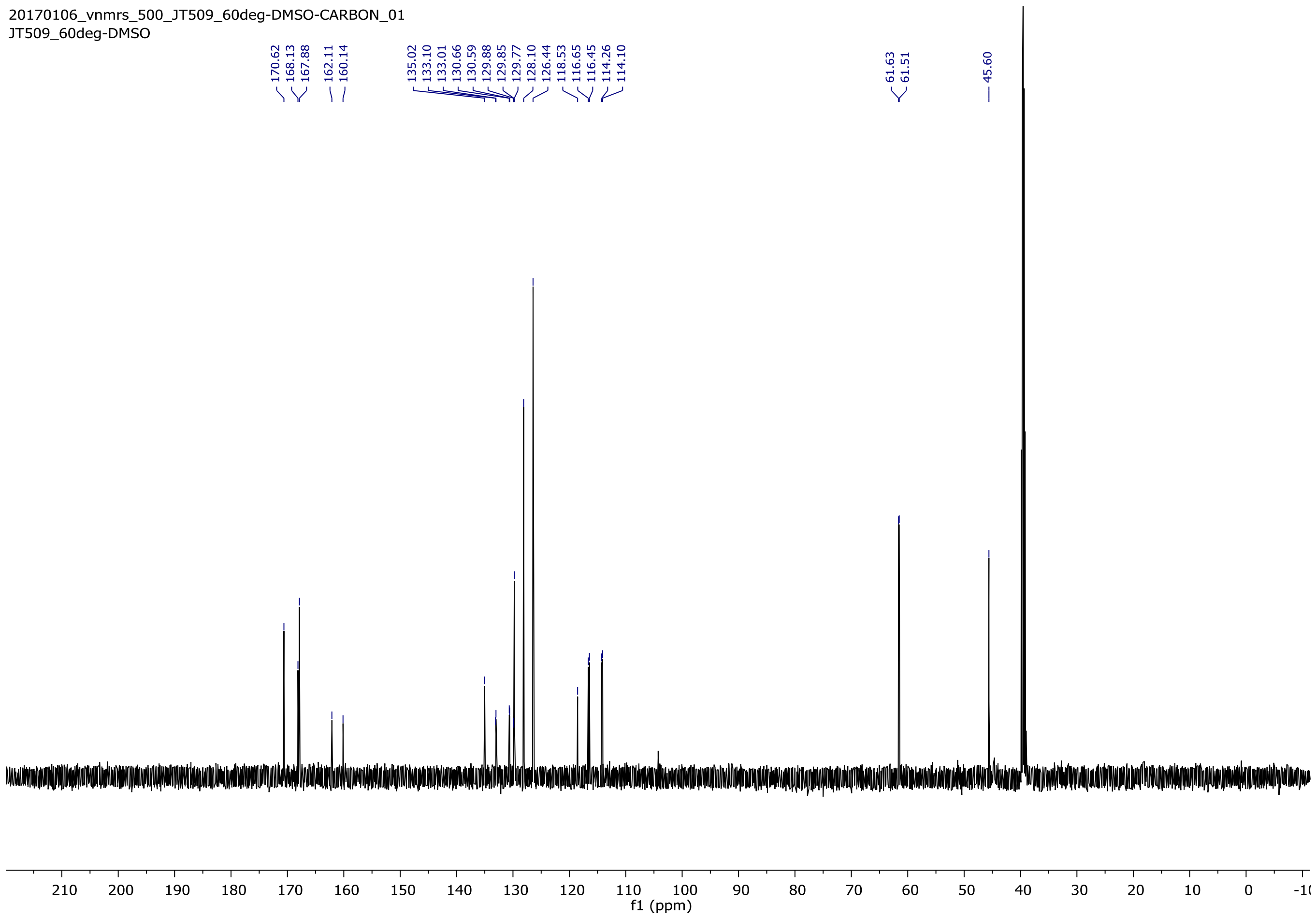

Supplementary Figure 66: ^{13}C NMR of **4j** in dimethylsulfoxide-d_6 at 60 °C.

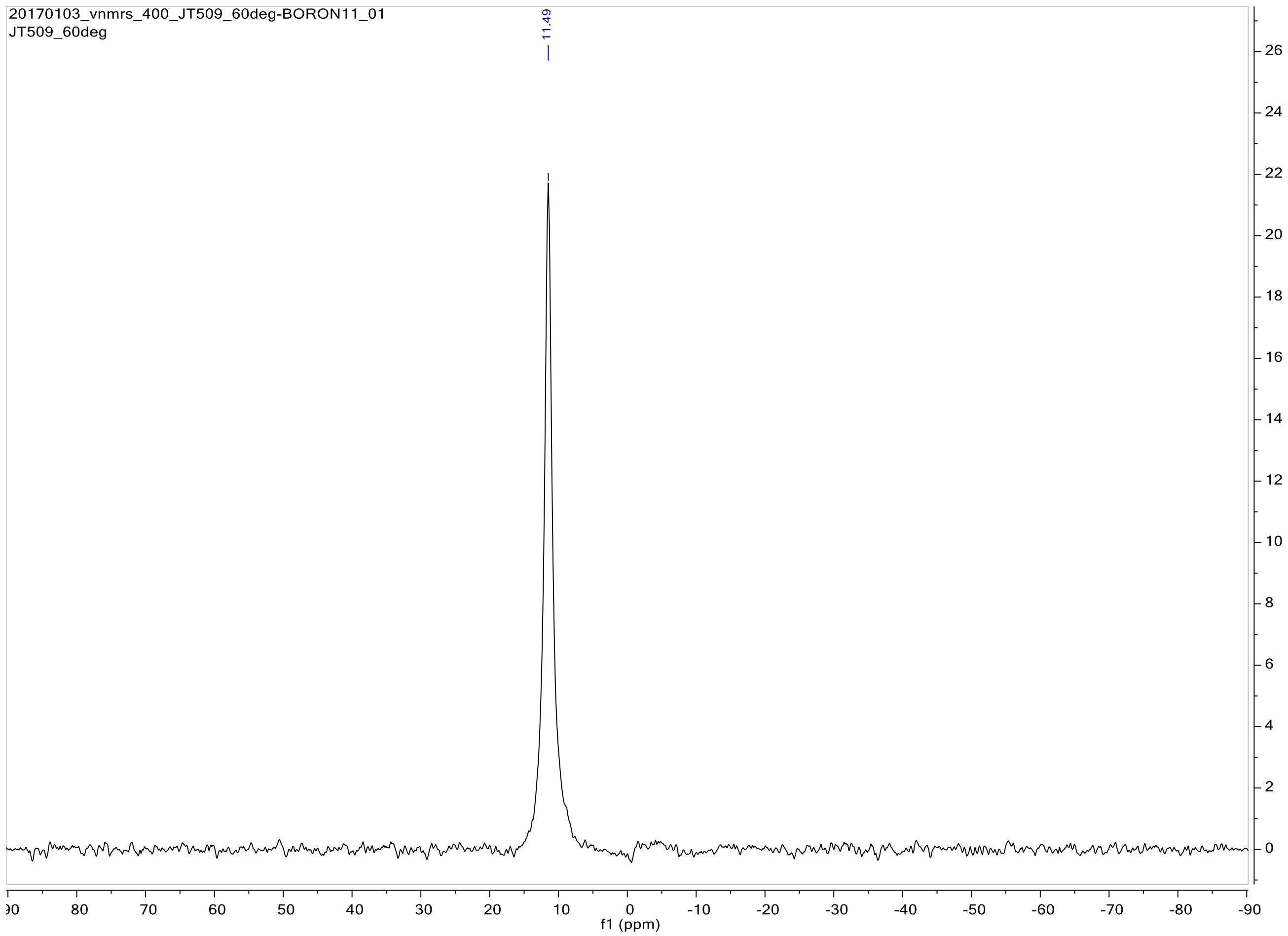

Supplementary Figure 67: ^{11}B NMR of **4j** in acetonitrile-d_3 at 60 °C.

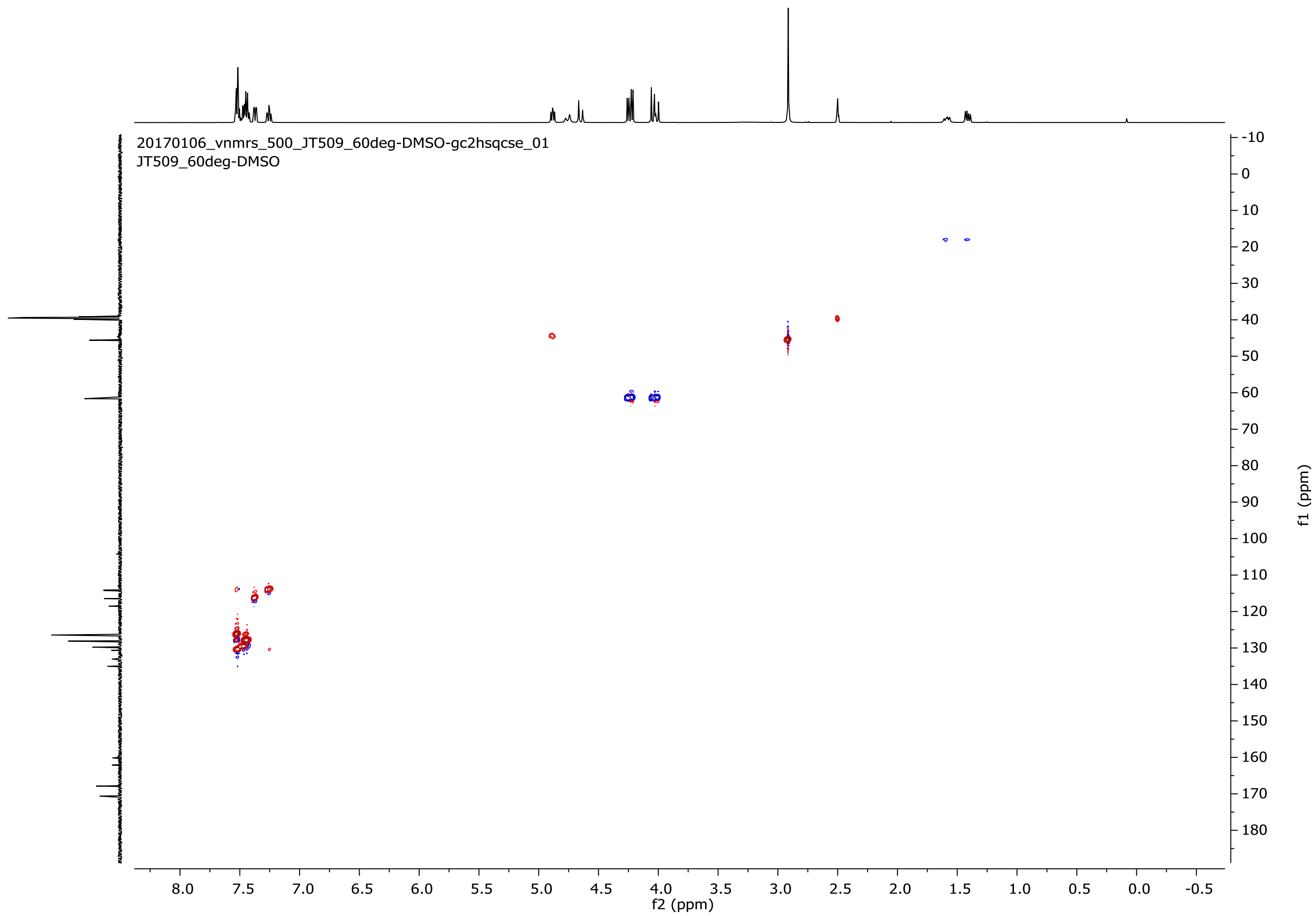

Supplementary Figure 68: ^{1}H-^{13}C HSQC NMR of **4j** in dimethylsulfoxide-d_6 at 60 °C.

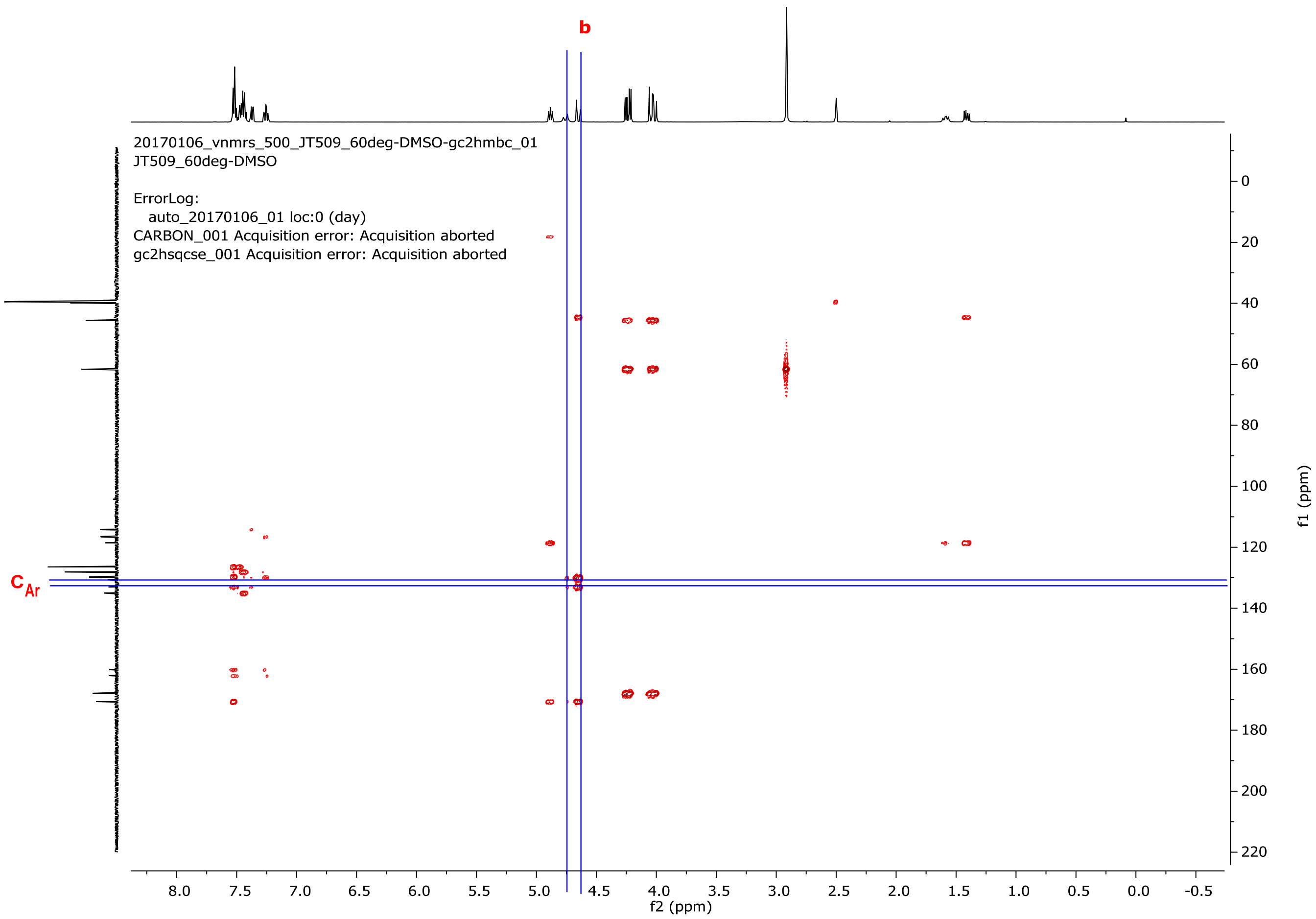

Supplementary Figure 69: ^{1}H-^{13}C HMBC NMR of **4j** in dimethylsulfoxide-d_6 at 60 °C.

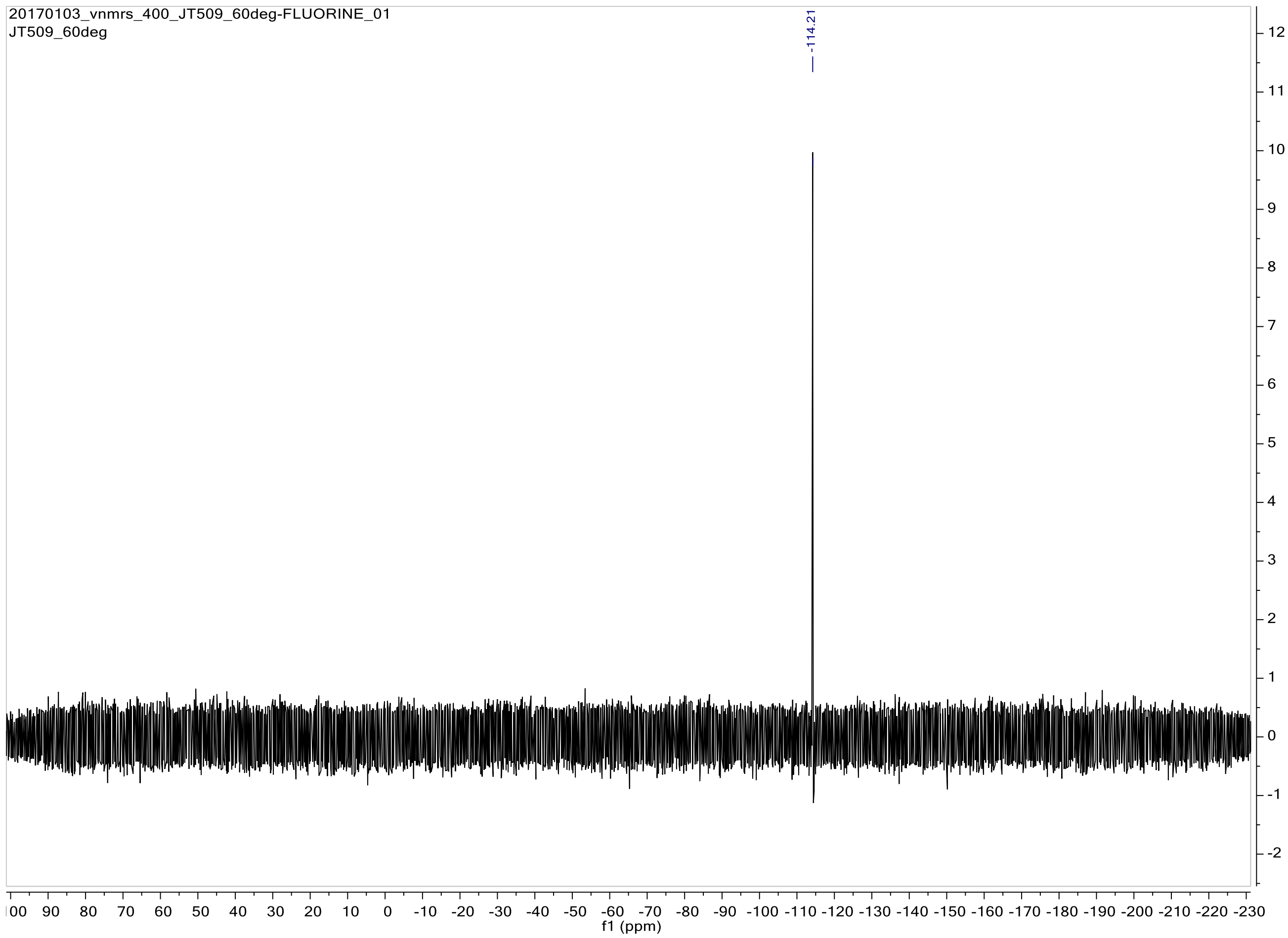

Supplementary Figure 70: ^{19}F NMR of **4j** in acetonitrile-d_3 at 60 °C.

Compound **4k**

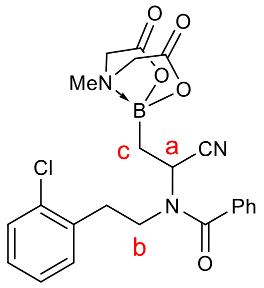

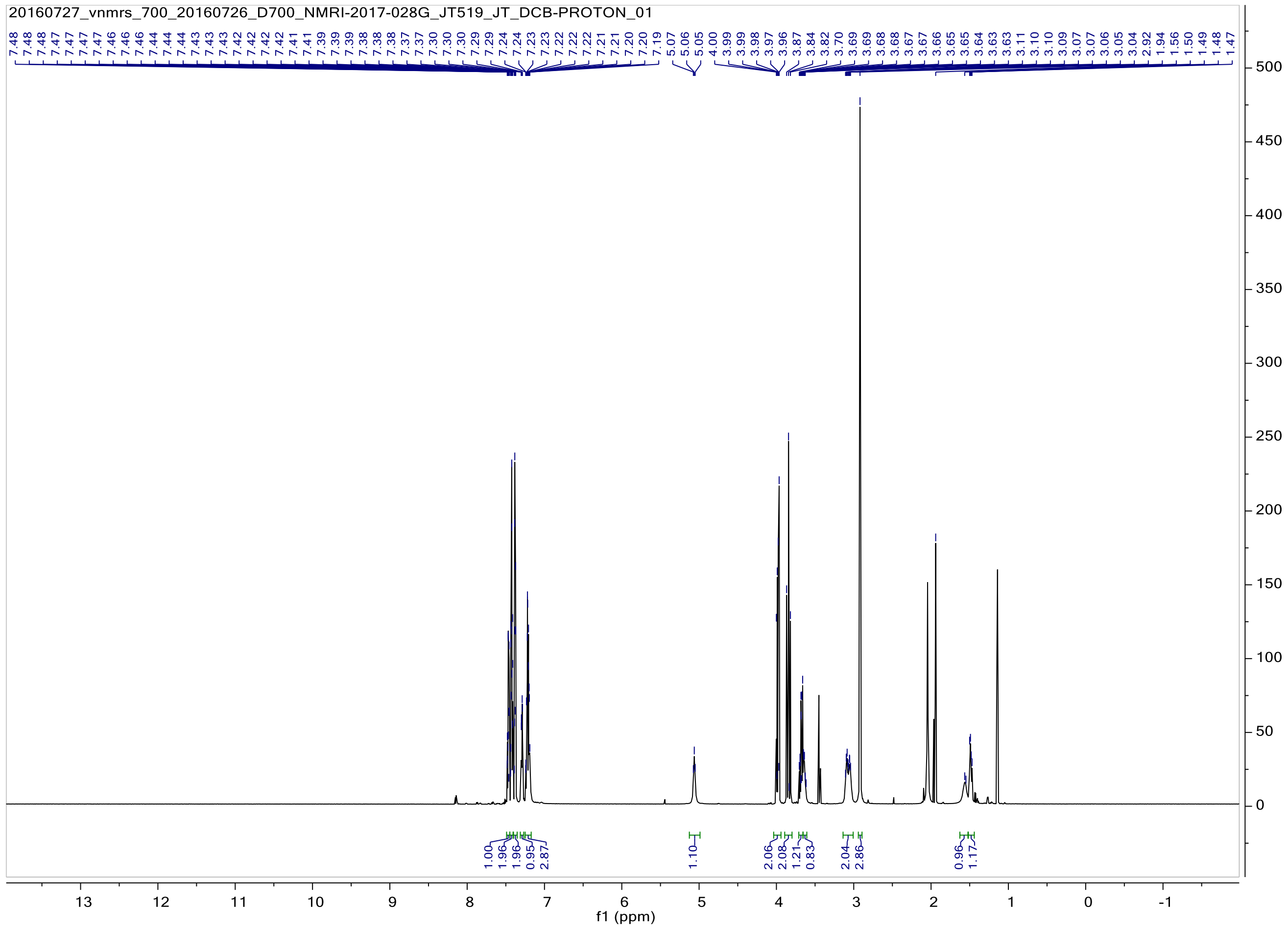

Supplementary Figure 71: ^{1}H NMR of **4k** in acetonitrile-d_3 at 60 °C.

659 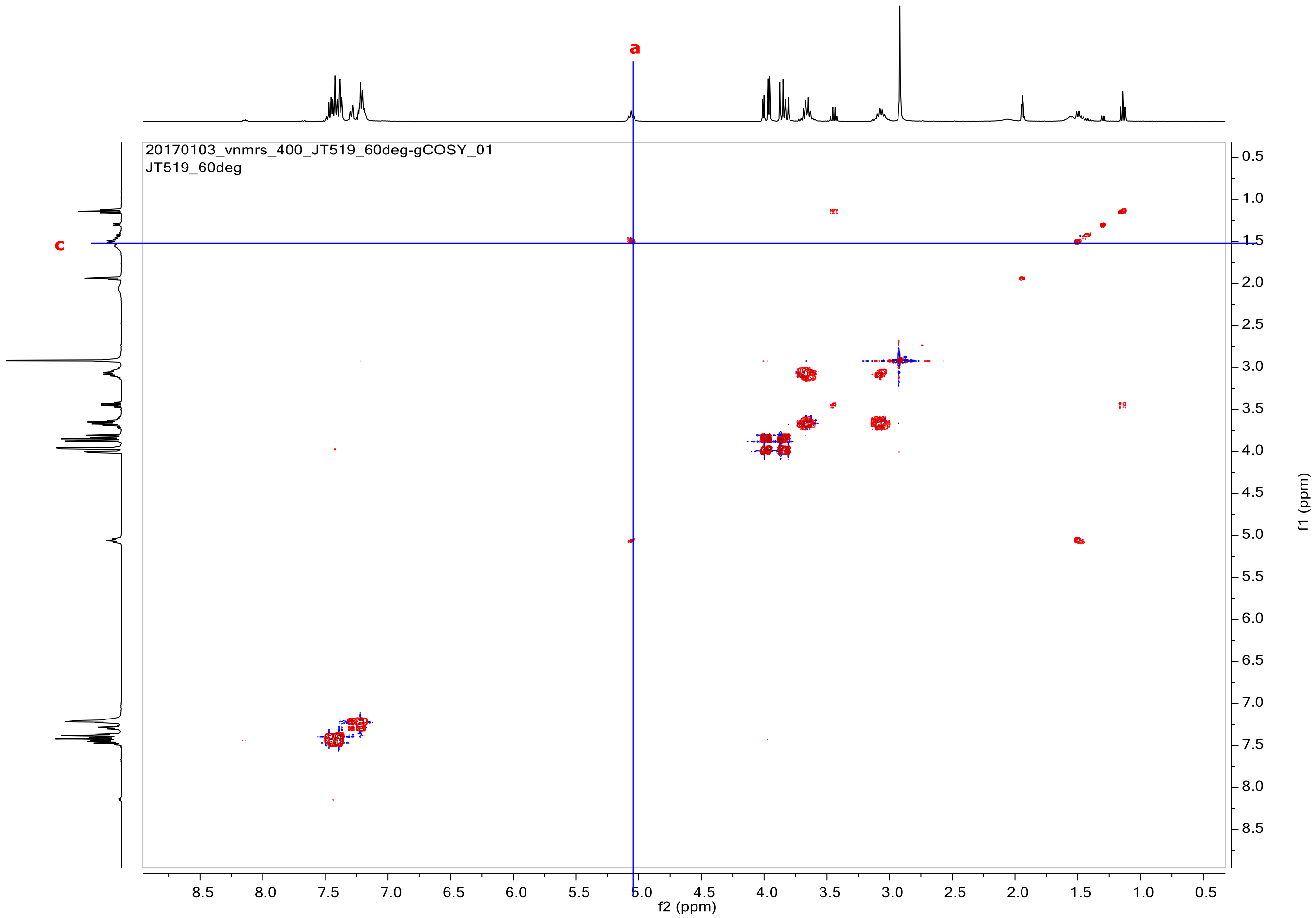

660 **Supplementary Figure 72:** ^{1}H-^{1}H COSY of **4k** in acetonitrile-d_3 at 60 °C.

661

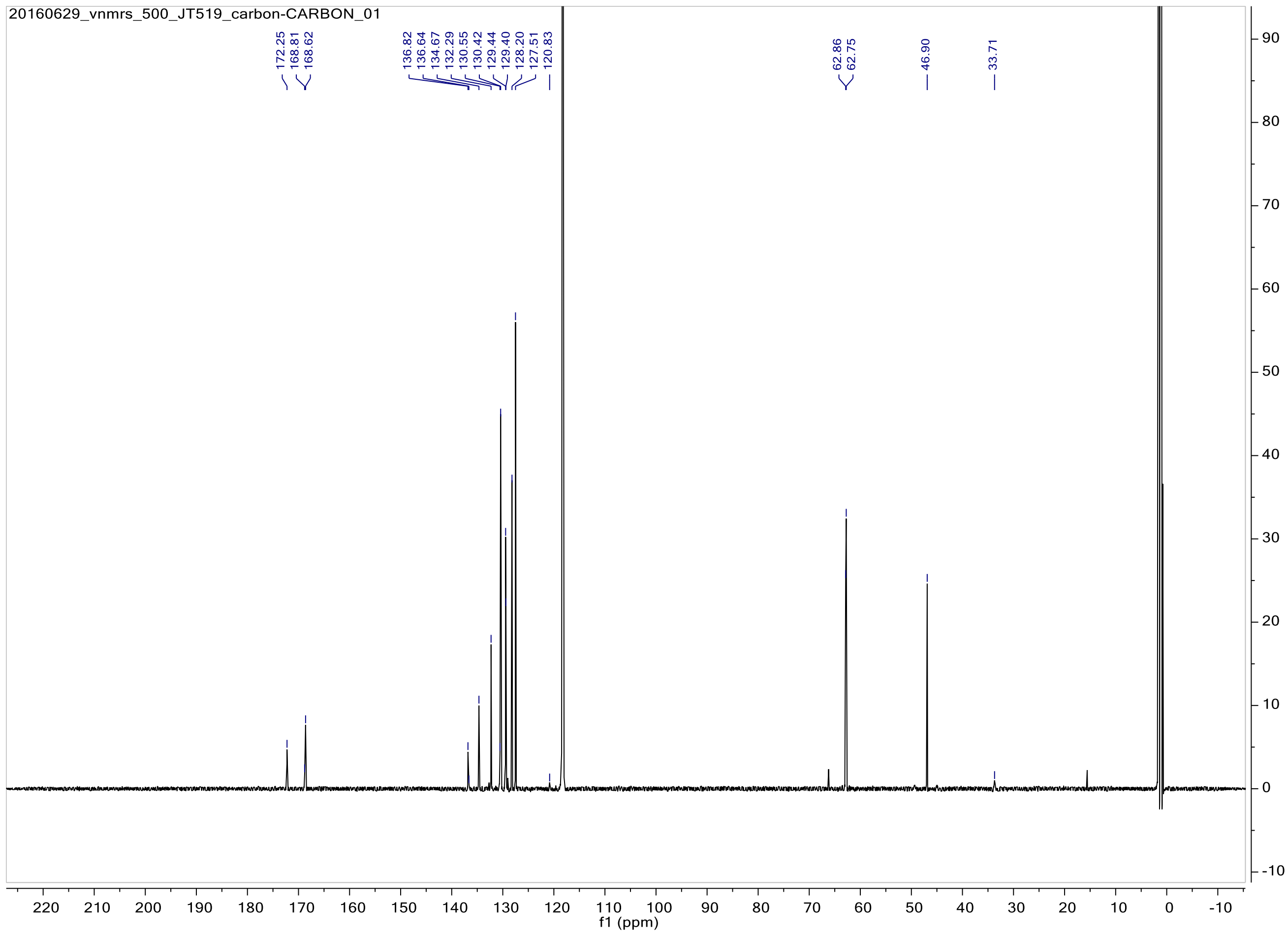

Supplementary Figure 73: ^{13}C NMR of **4k** in acetonitrile-d_3 at 25 °C.

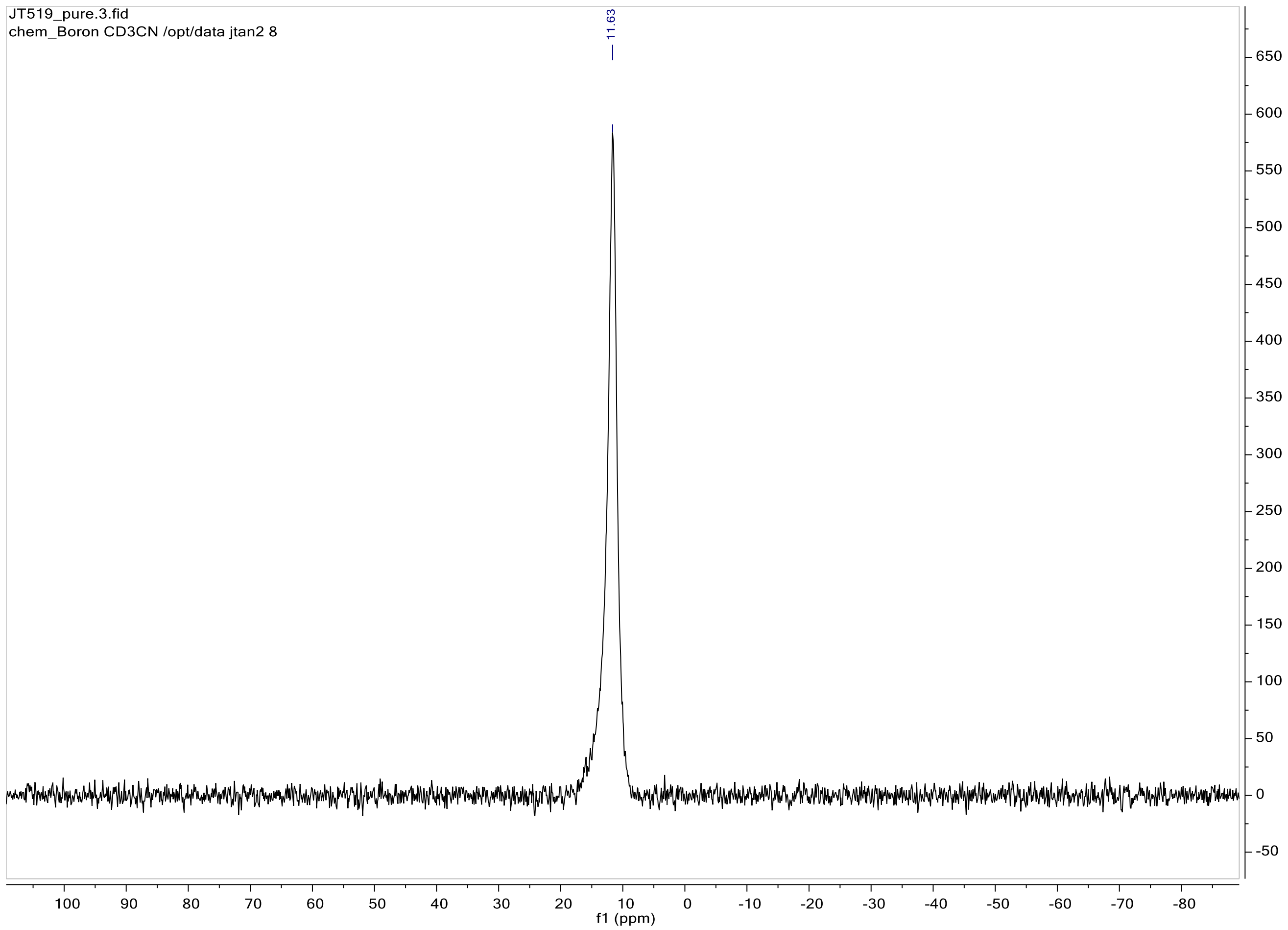

Supplementary Figure 74: ^{11}B NMR of **4k** in acetonitrile-d_3 at 25 °C.

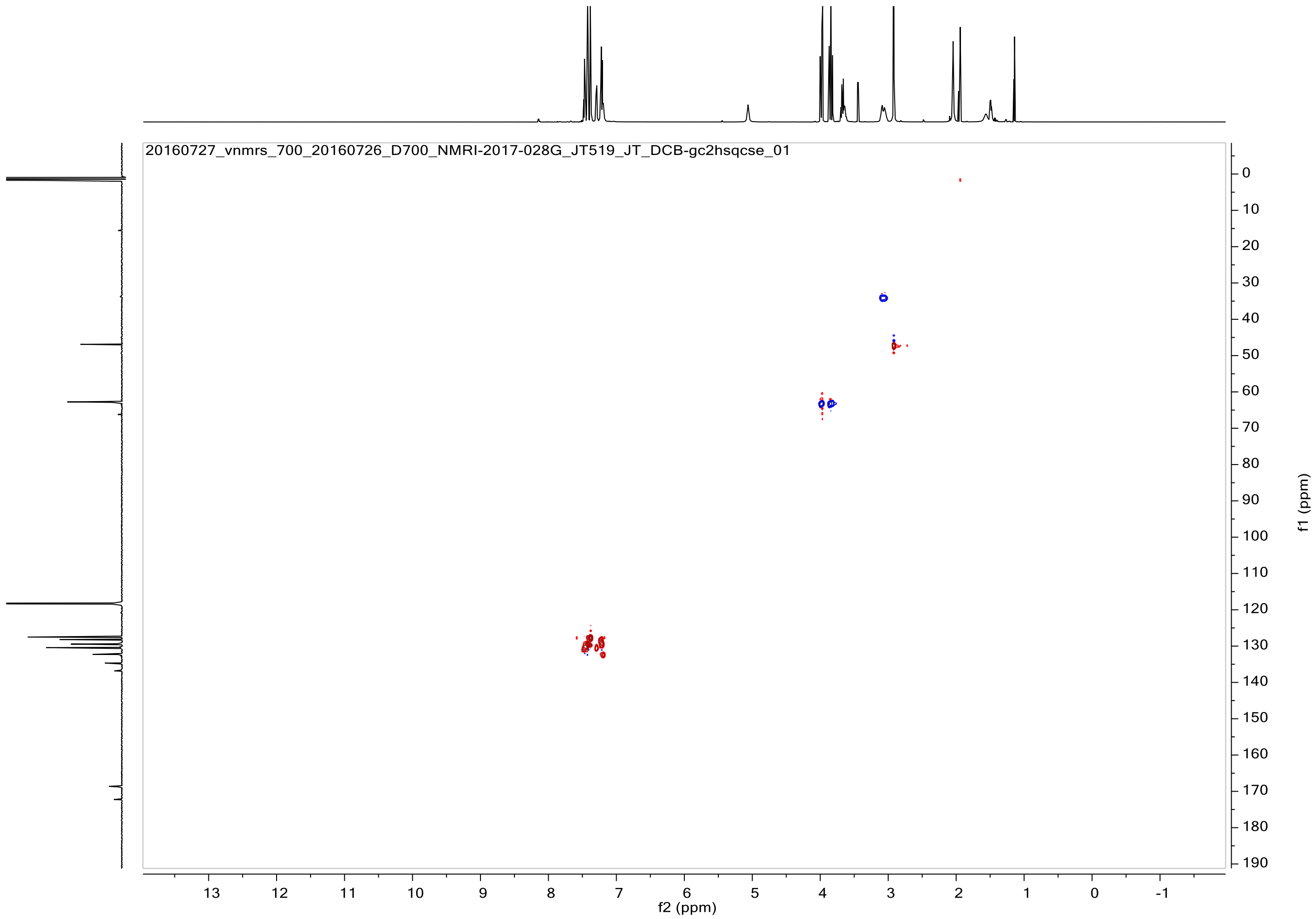

669

670 **Supplementary Figure 75:** ^{1}H-^{13}C HSQC NMR of **4k** in acetonitrile-d_3 at 60 °C.

Compound **4l**

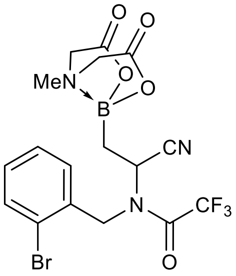

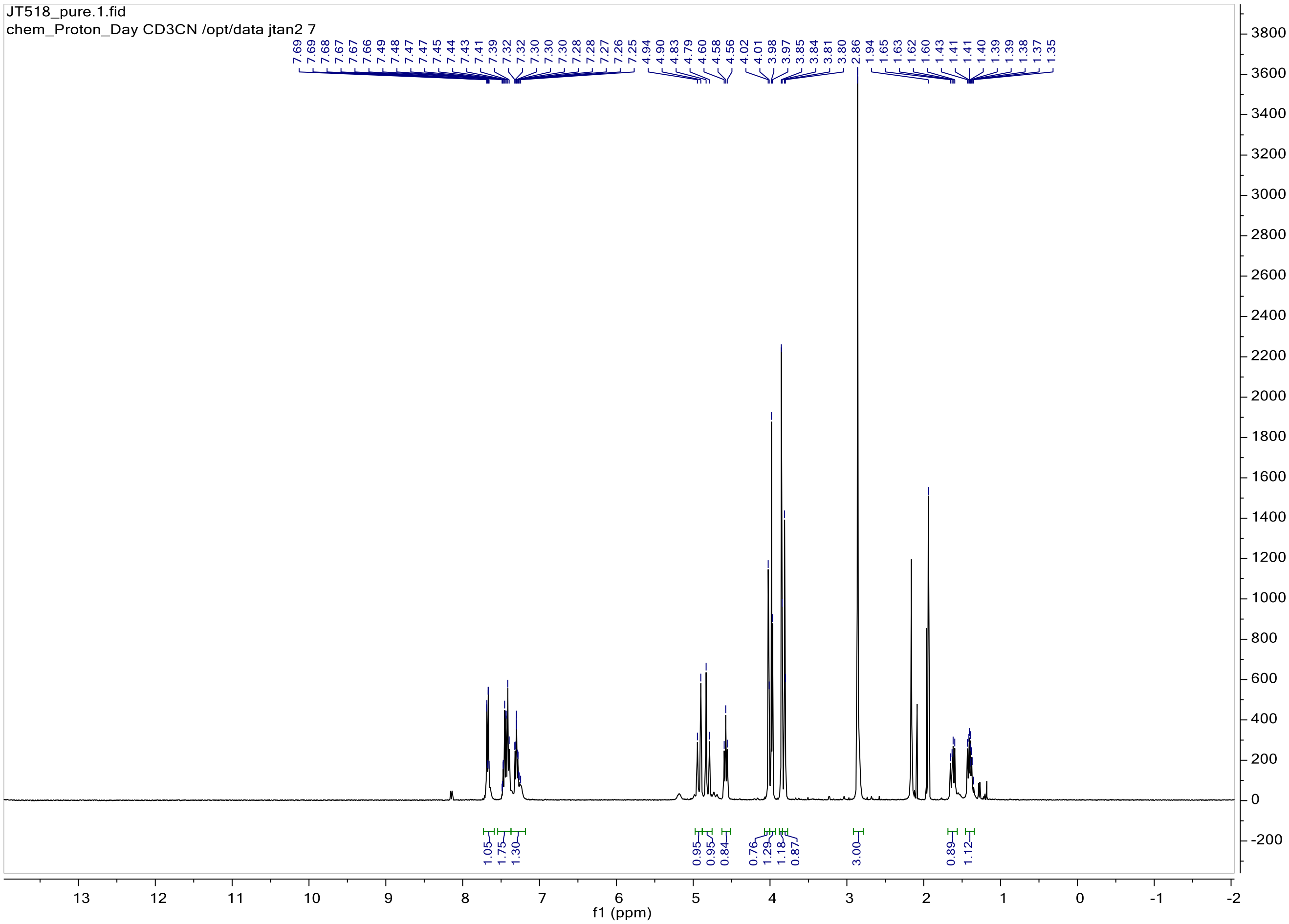

Supplementary Figure 76: ^{1}H NMR of **4l** in acetonitrile-d_3 at 25 °C.

676

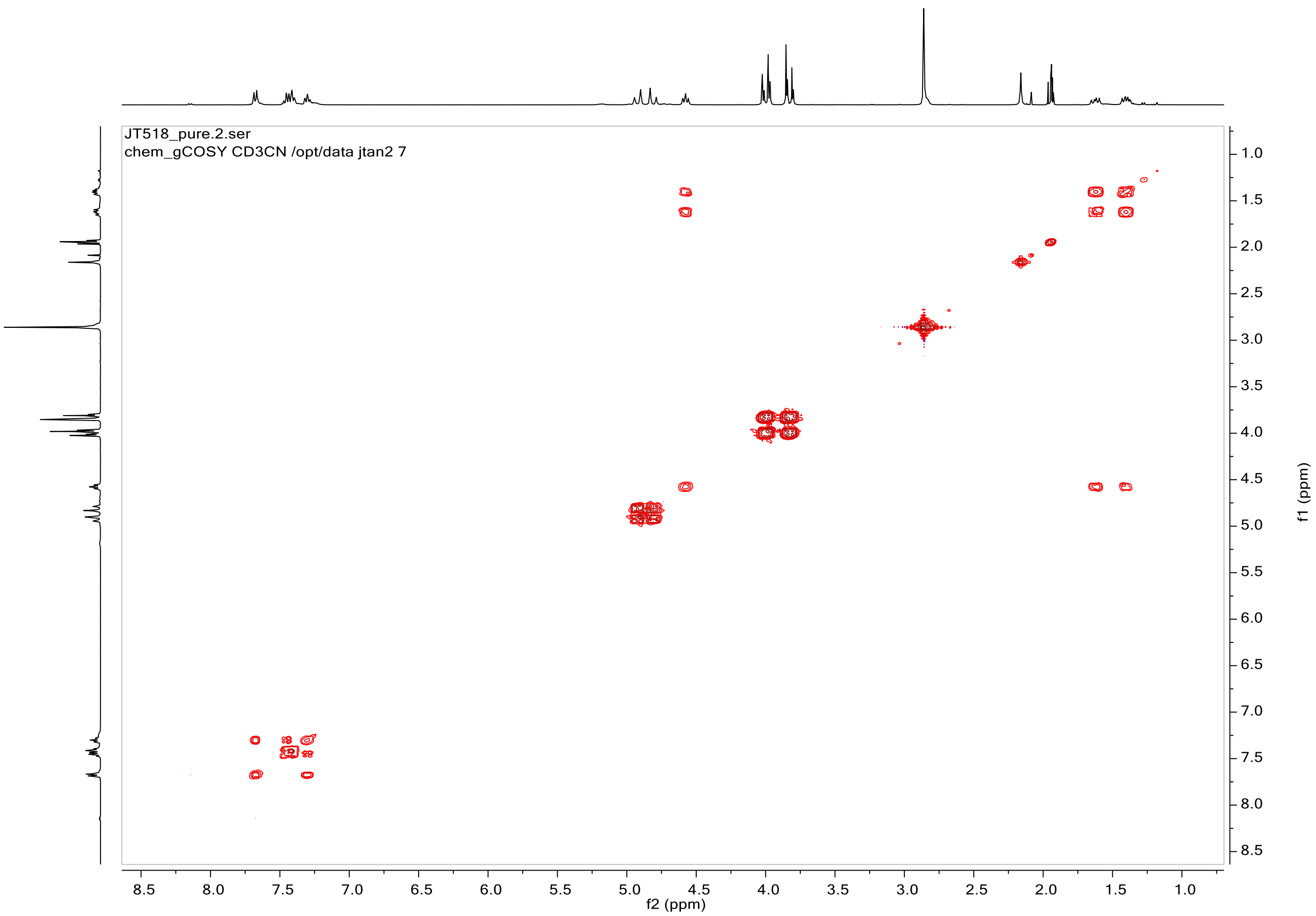

677 **Supplementary Figure 77:** ^{1}H-^{1}H COSY of **4l** in acetonitrile-d_3 at 25 °C.

678

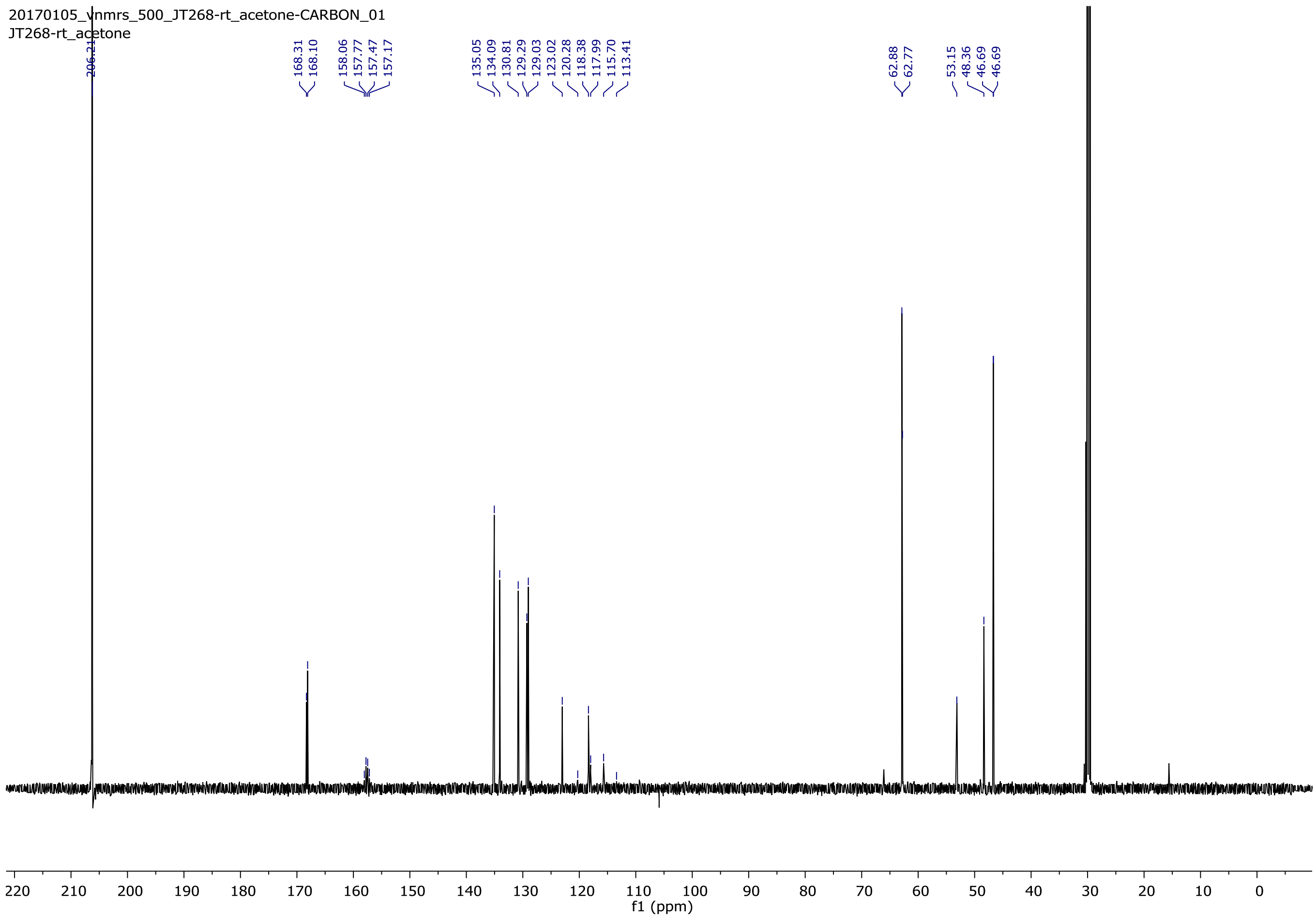

Supplementary Figure 78: ^{13}C NMR of **4l** in acetone-d_6 at 25 °C.

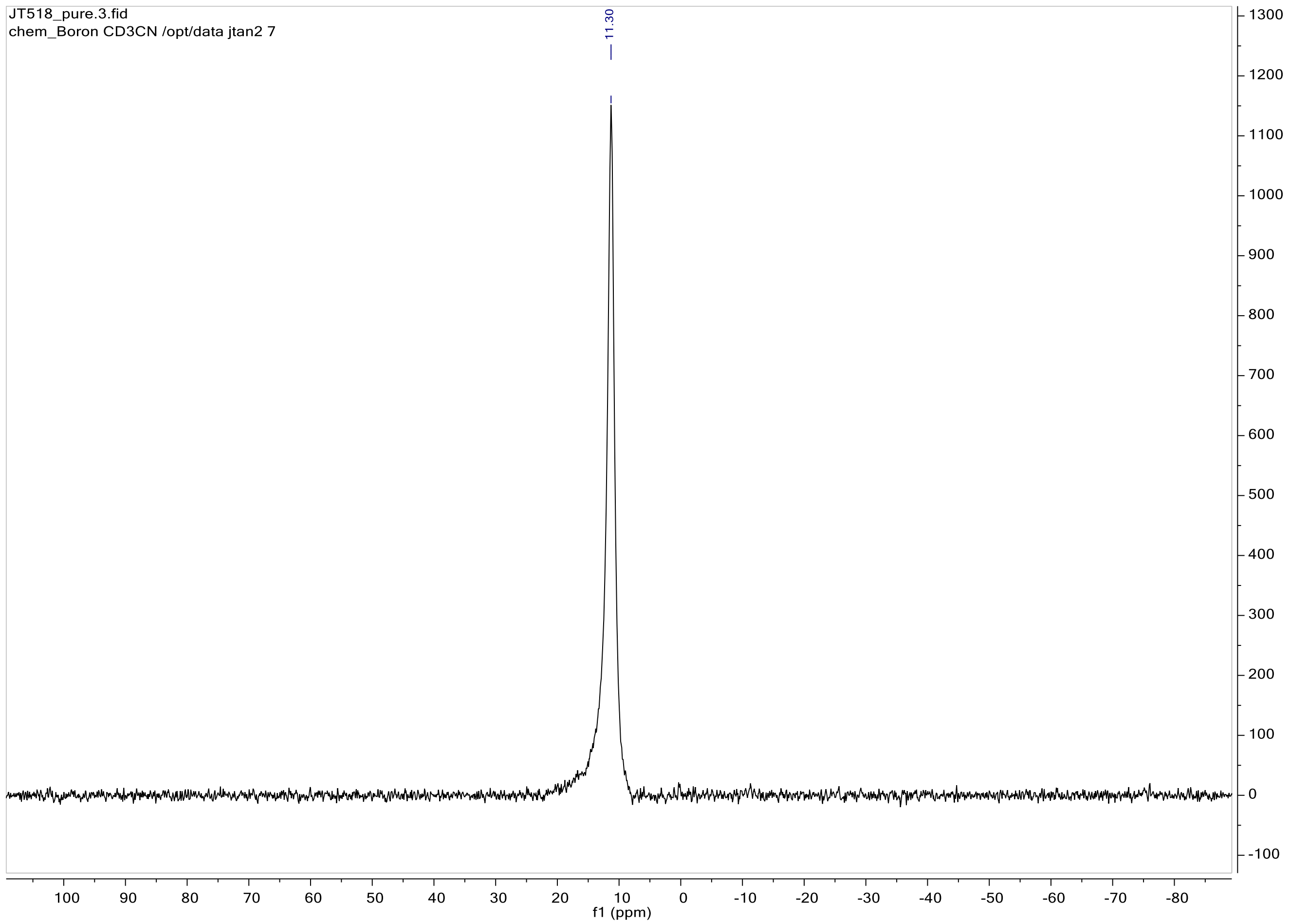

Supplementary Figure 79: ^{11}B NMR of **4l** in acetonitrile-d_3 at 25 °C.

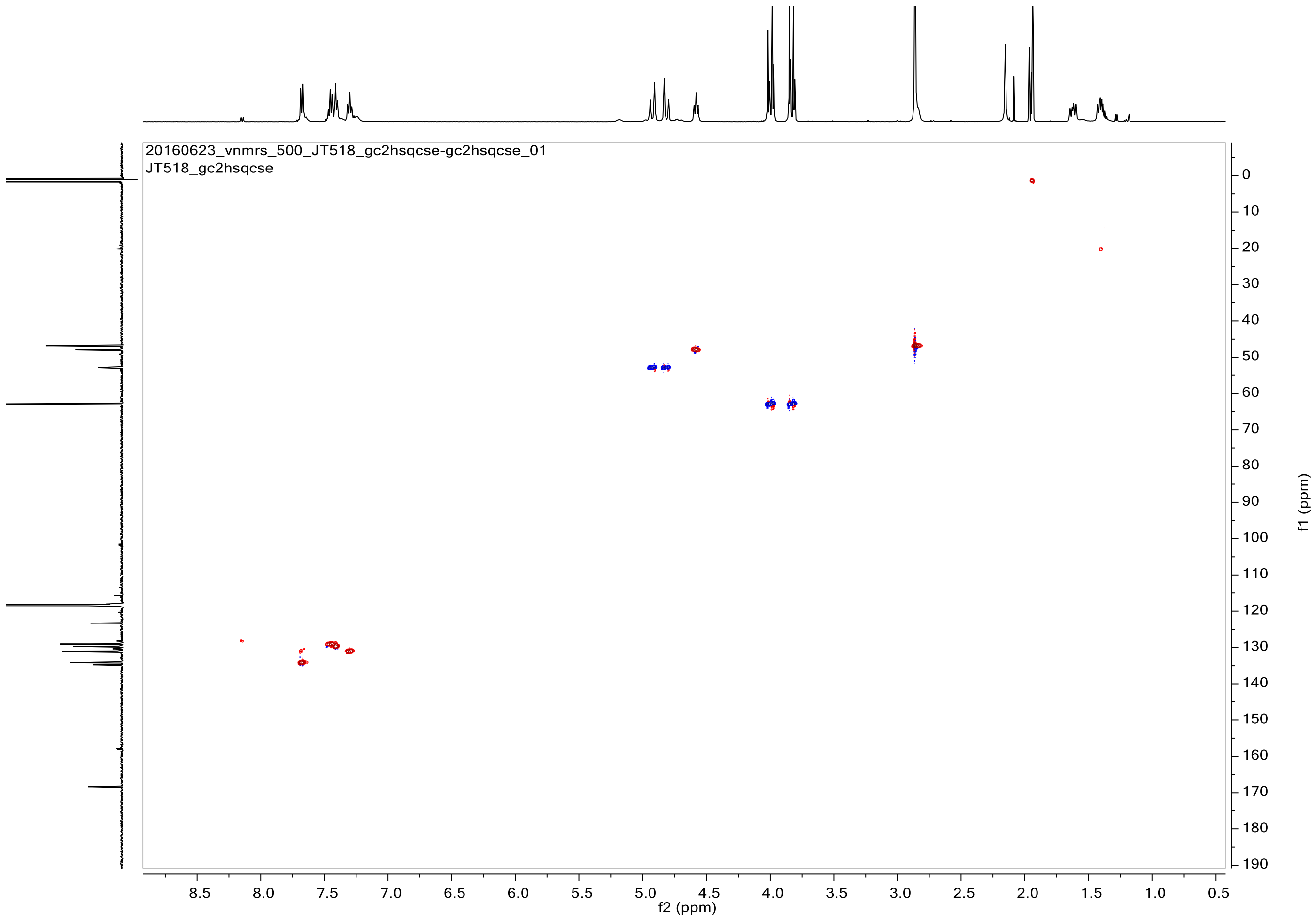

Supplementary Figure 80: ^{1}H-^{13}C HSQC NMR of **4l** in acetonitrile-d_3 at 25 °C.

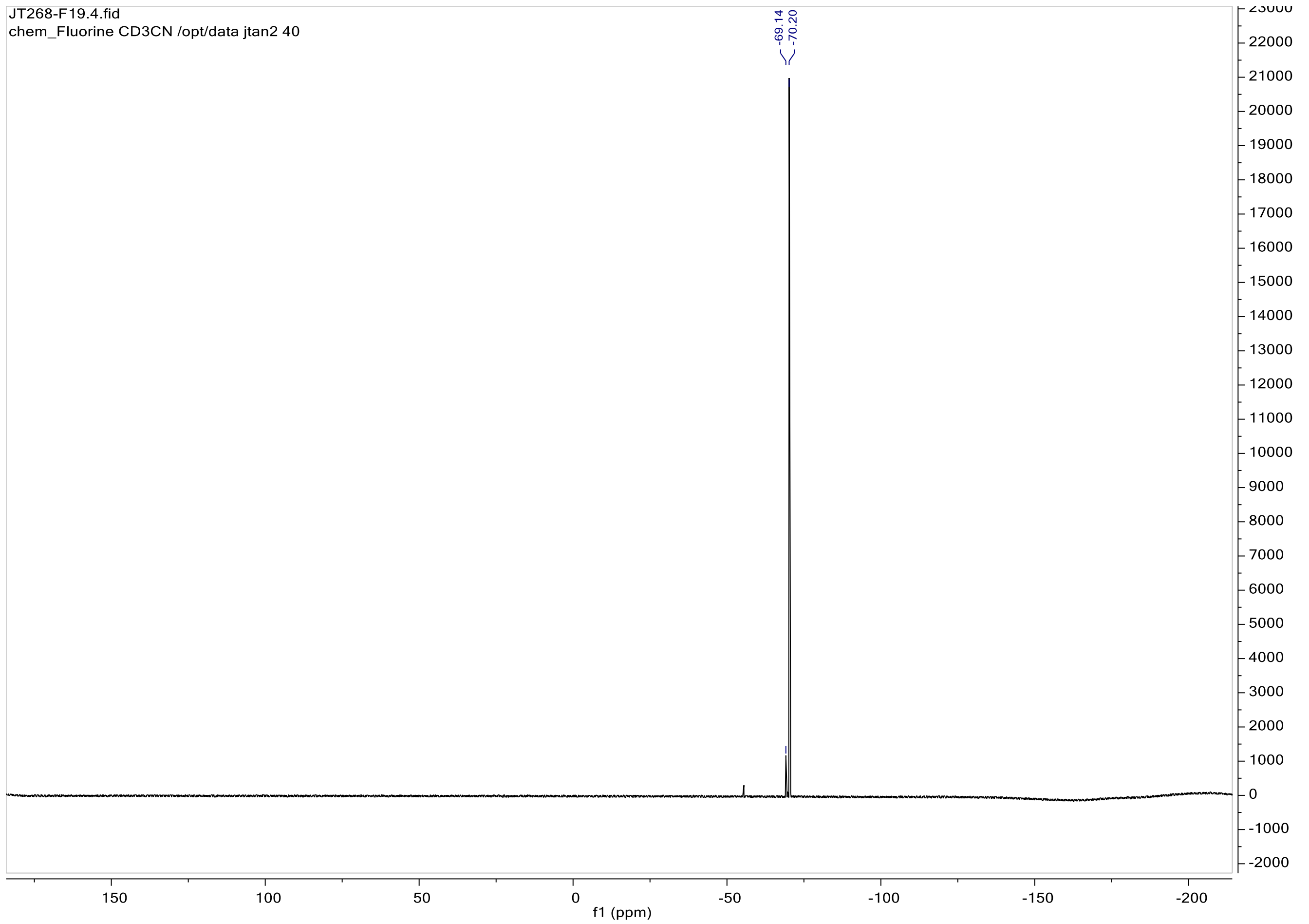

689 **Supplementary Figure 81:** ^{19}F NMR of **4l** in acetonitrile-d_3 at 25 °C.

Compound **7a**

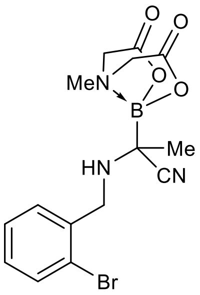

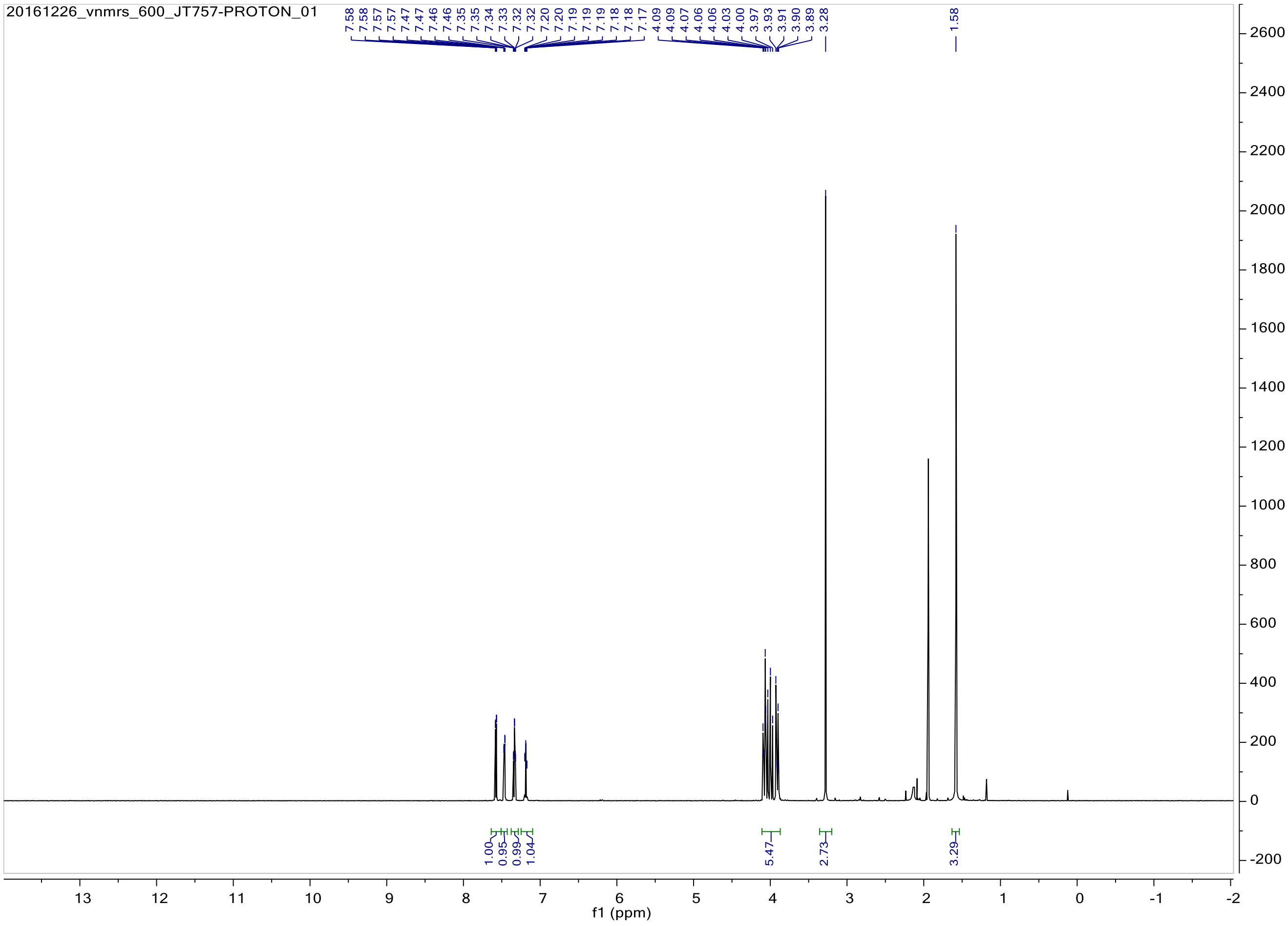

Supplementary Figure 82: ^{1}H NMR of **7a** in acetonitrile-d_3 at 25 °C.

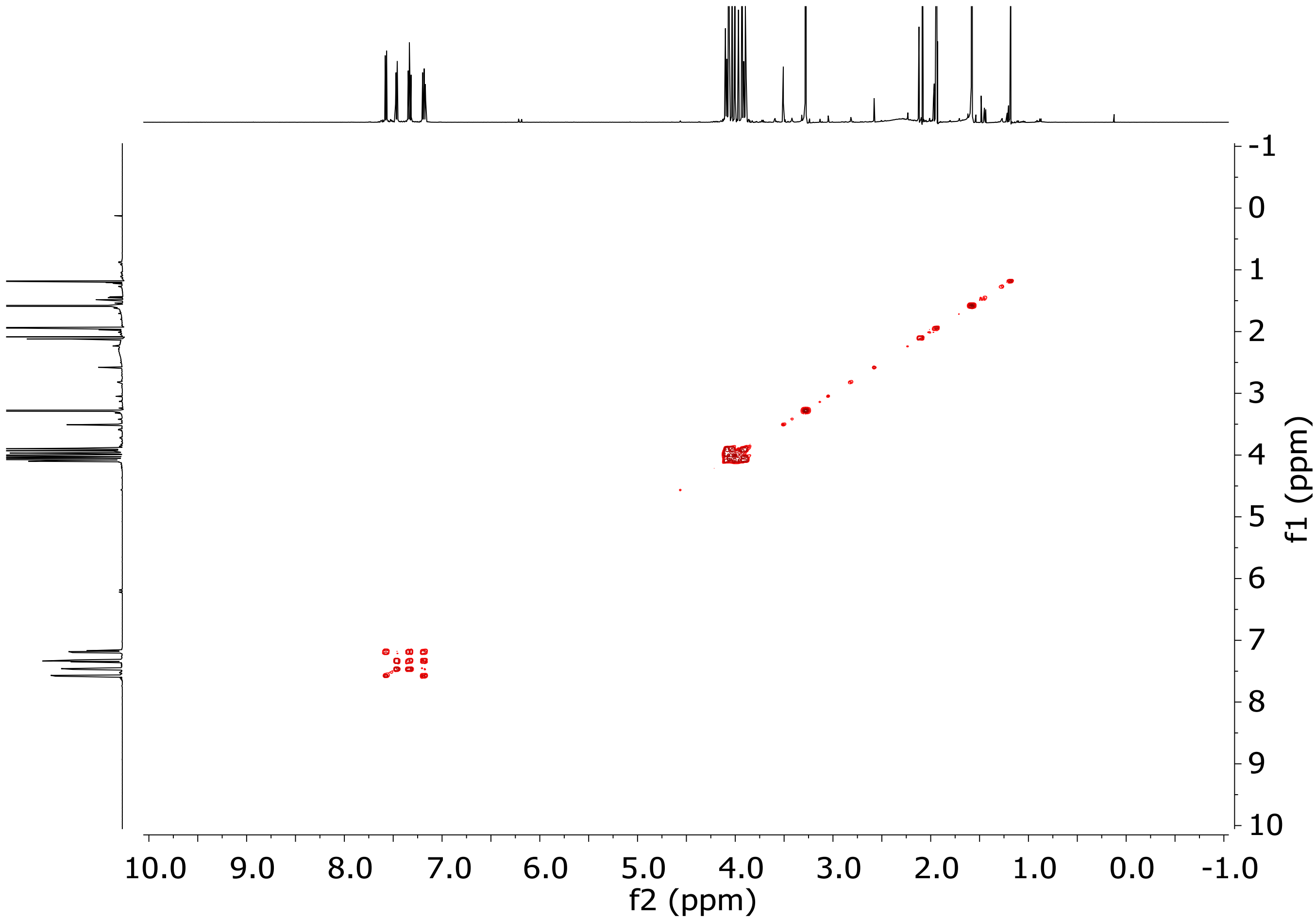

Supplementary Figure 83: ^{1}H-^{1}H COSY of **7a** in acetonitrile-d_3 at 25 °C.

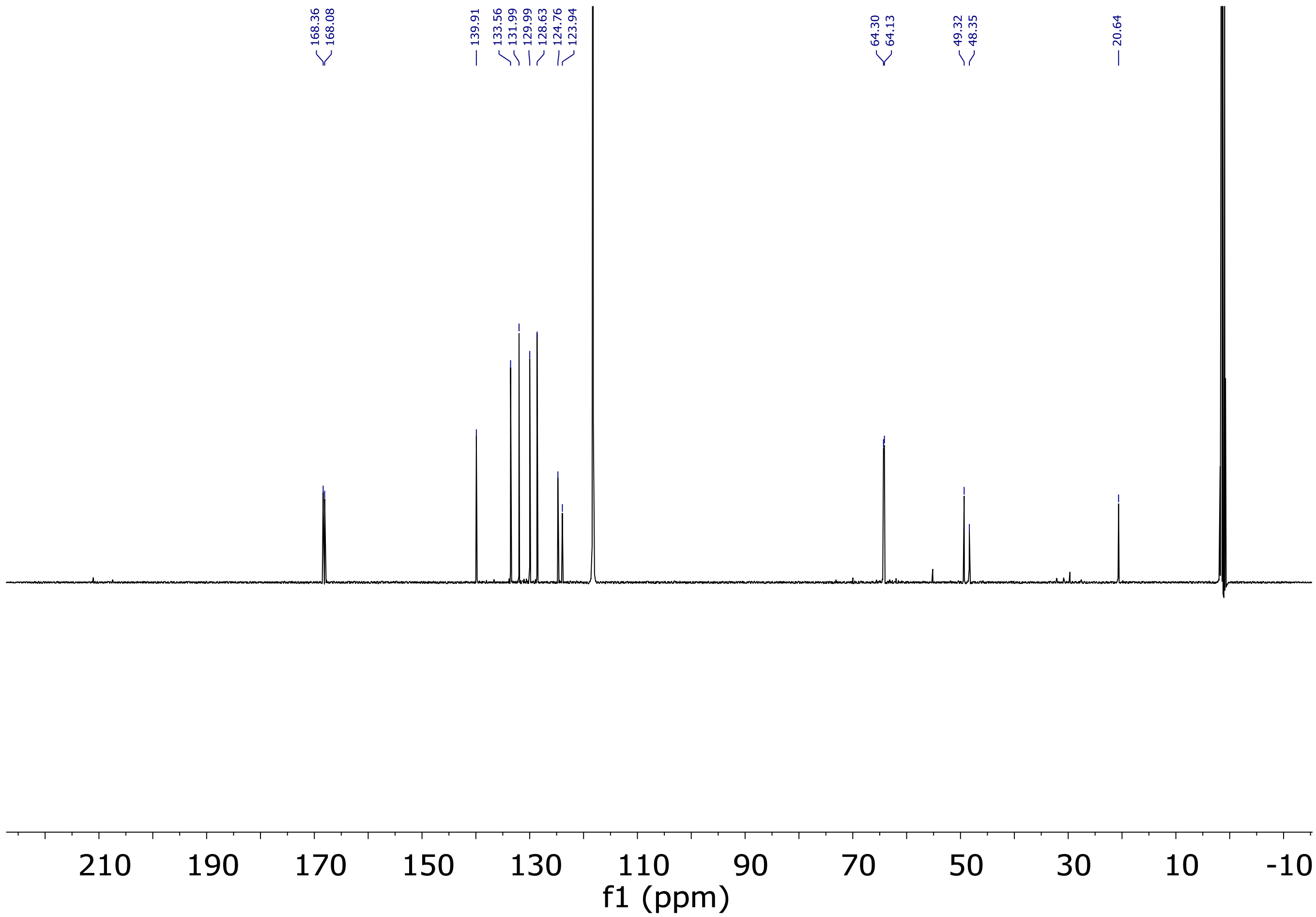

Supplementary Figure 84: ^{13}C NMR of **7a** in acetonitrile-d_3 at 25 °C.

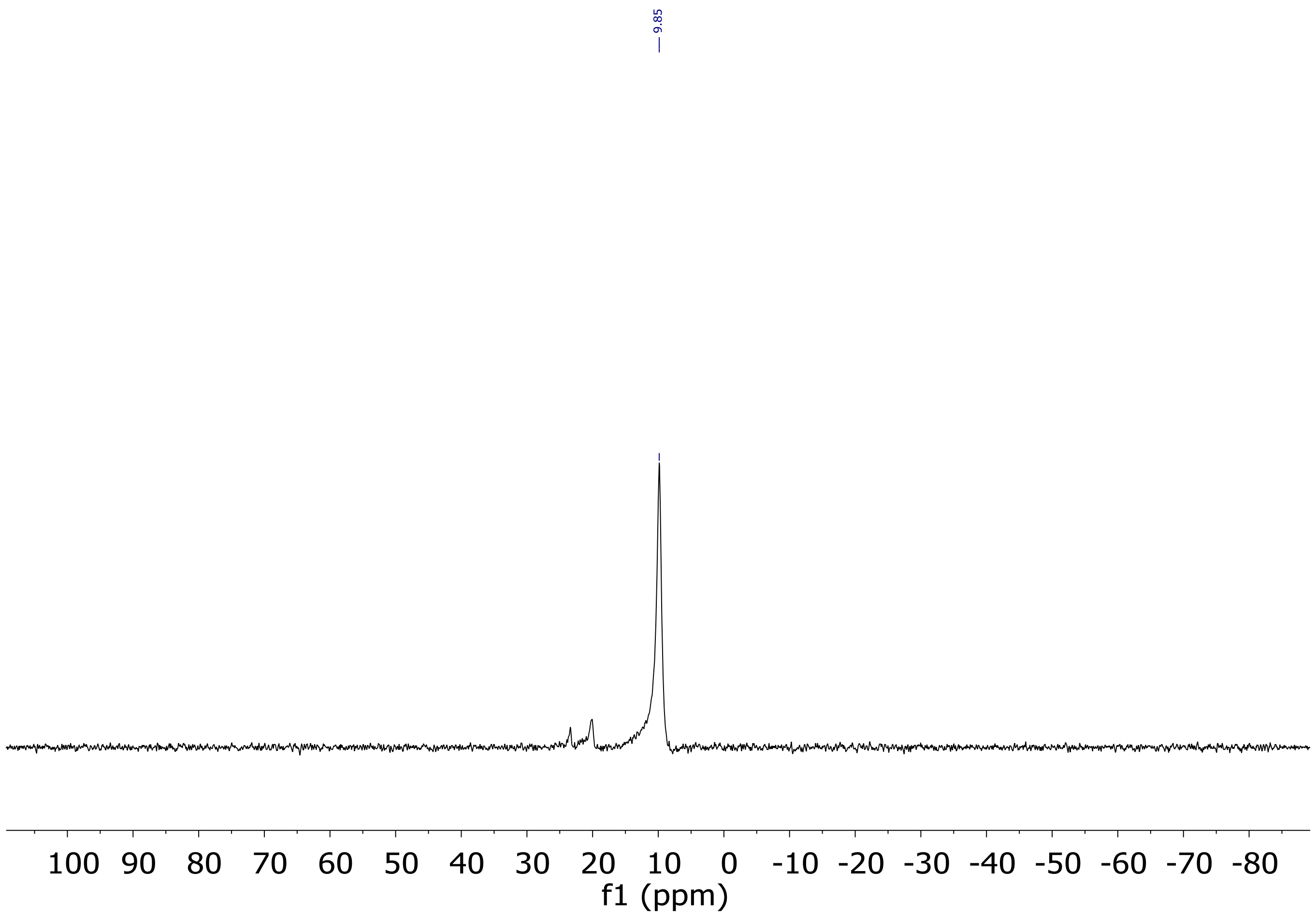

Supplementary Figure 85: ^{11}B NMR of **7a** in acetonitrile-d_3 at 25 °C.

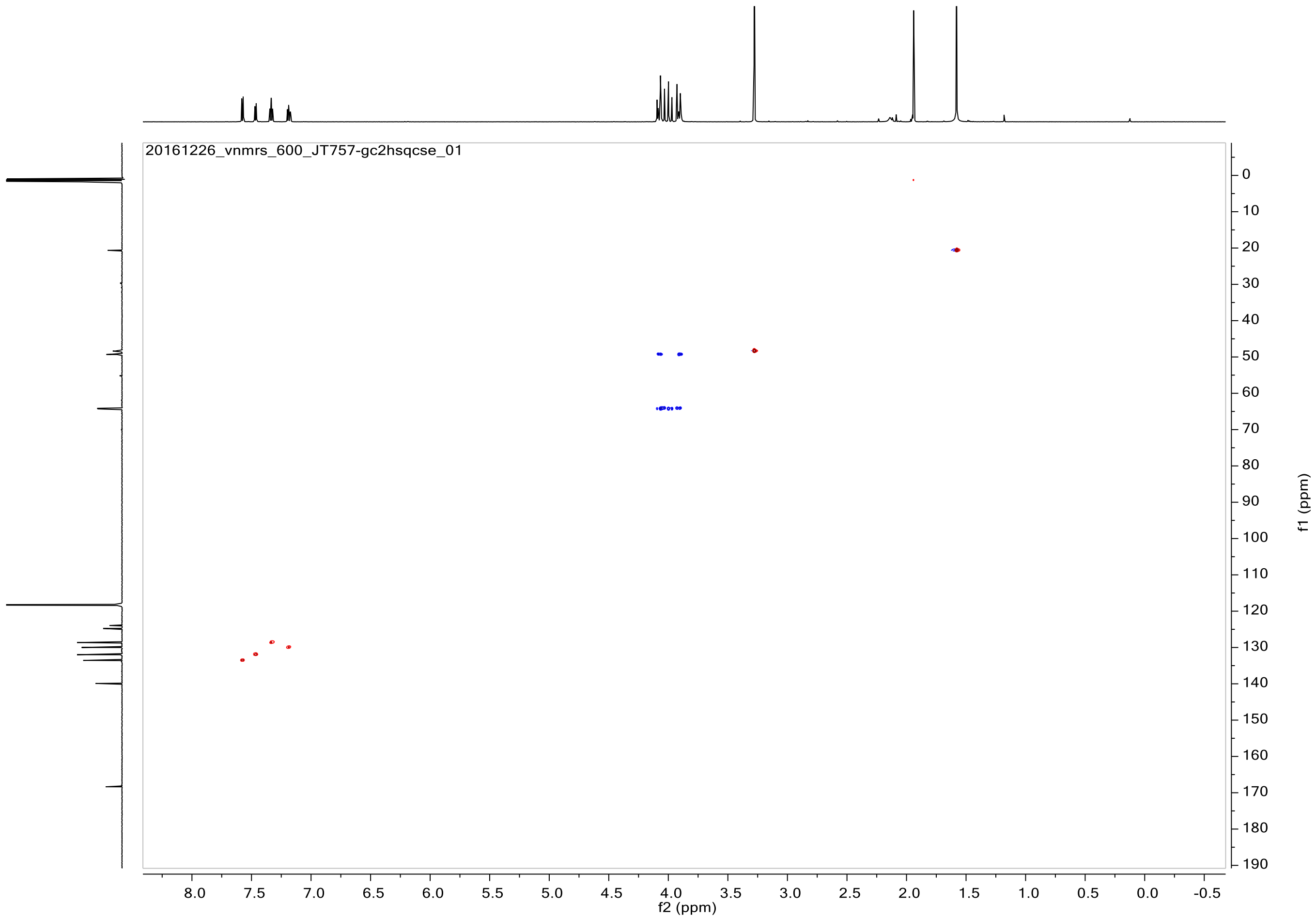

Supplementary Figure 86: ^{1}H-^{13}C HSQC NMR of **7a** in acetonitrile-d_3 at 25 °C.

Compound **11a**

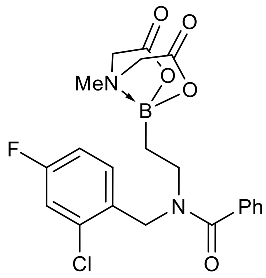

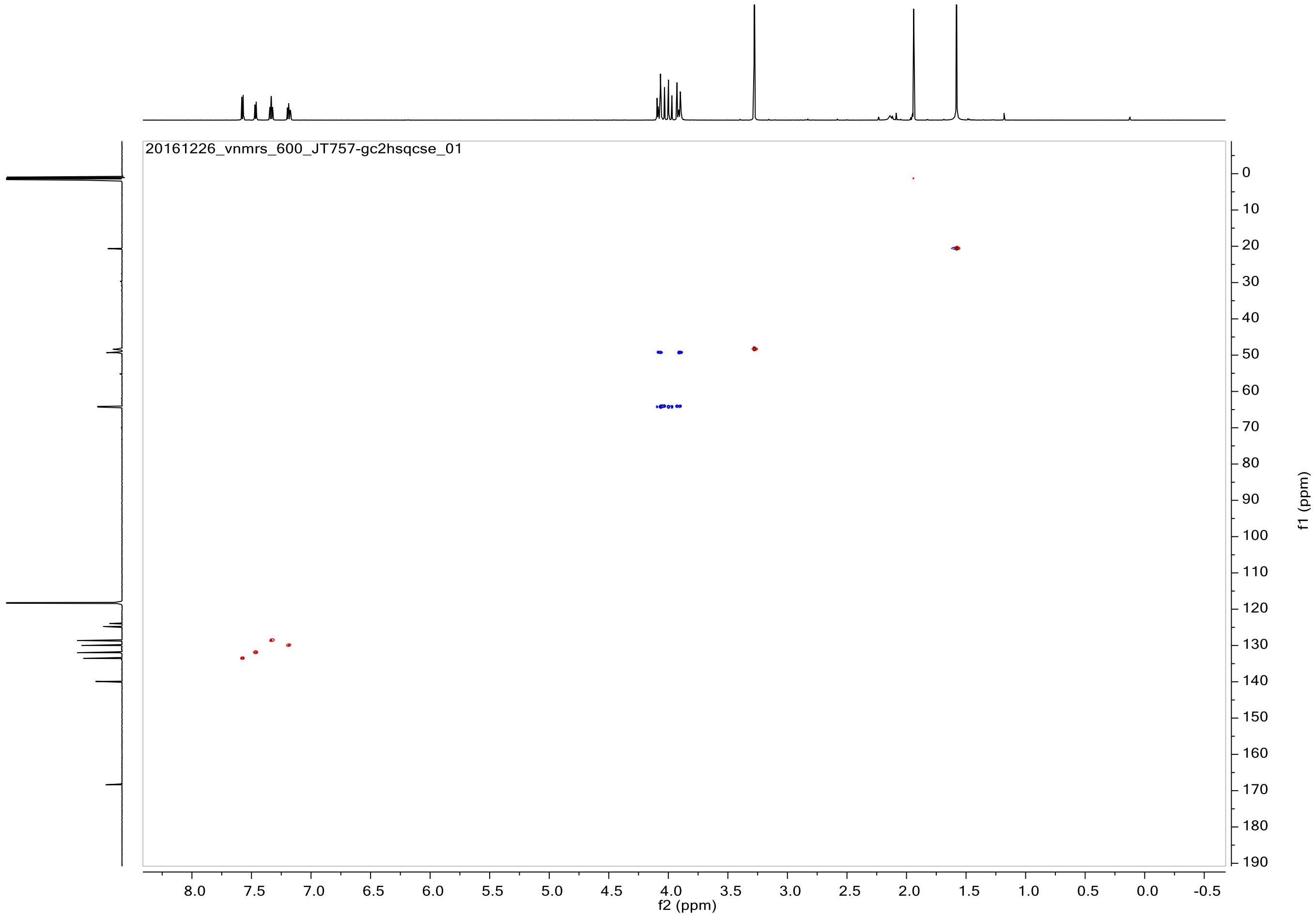

Supplementary Figure 87: ^{1}H NMR of **11a** in acetonitrile-d_3 at 60 °C.

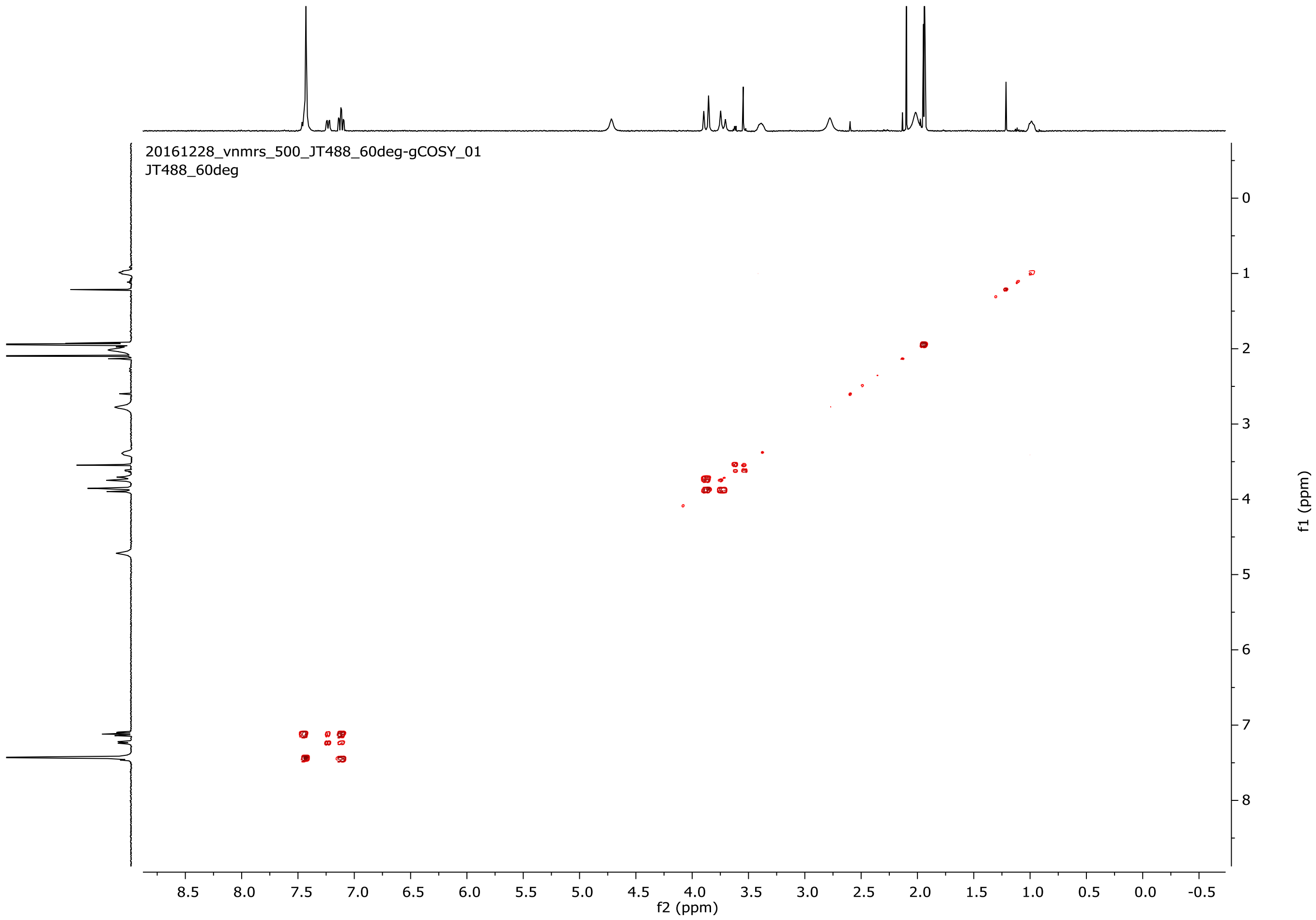

Supplementary Figure 88: ^1H-^1H COSY of **11a** in acetonitrile-d_3 at 60 °C.

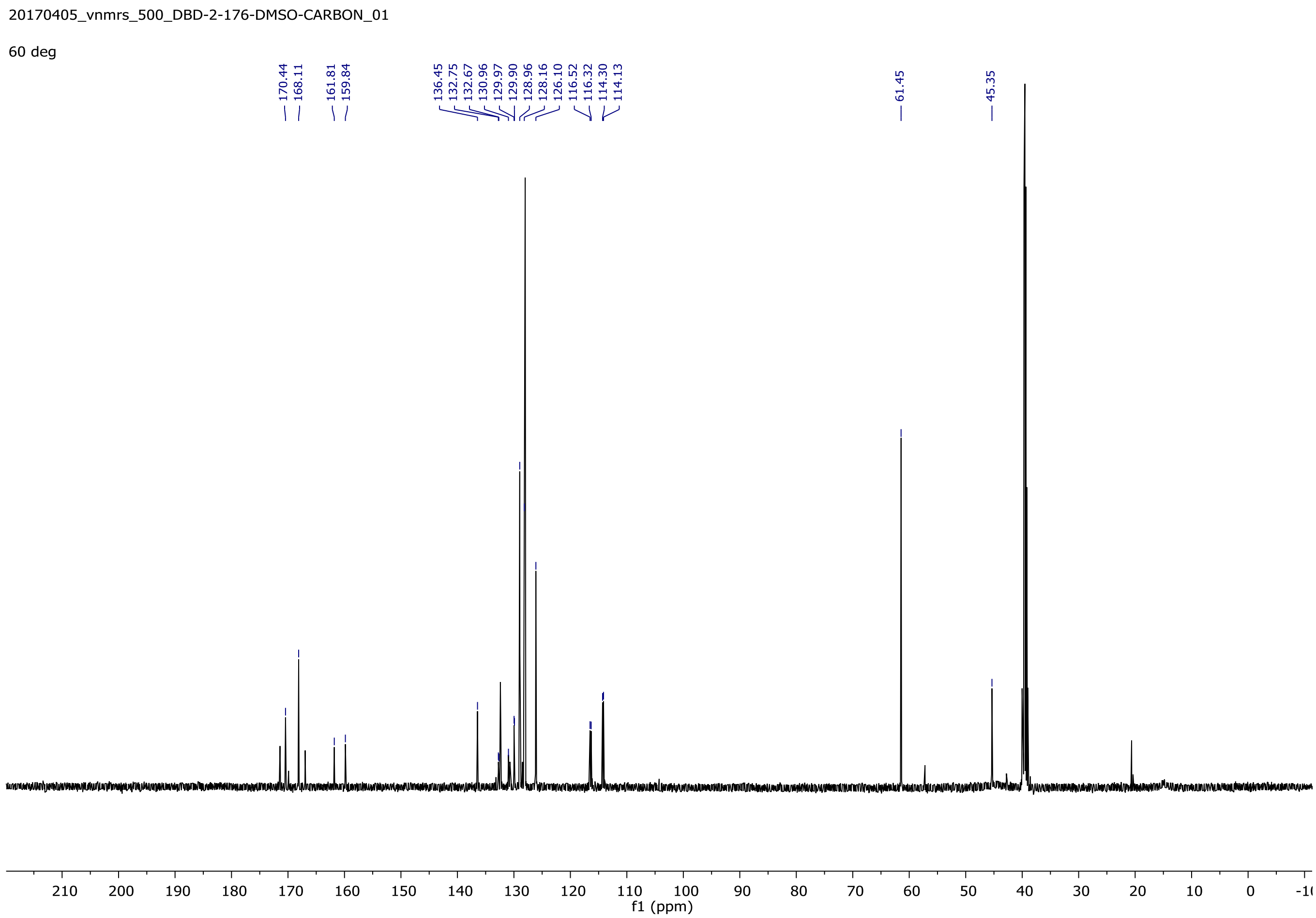

Supplementary Figure 89: ^{13}C NMR of **11a** in dimethylsulfoxide-*d_3* at 60 °C.

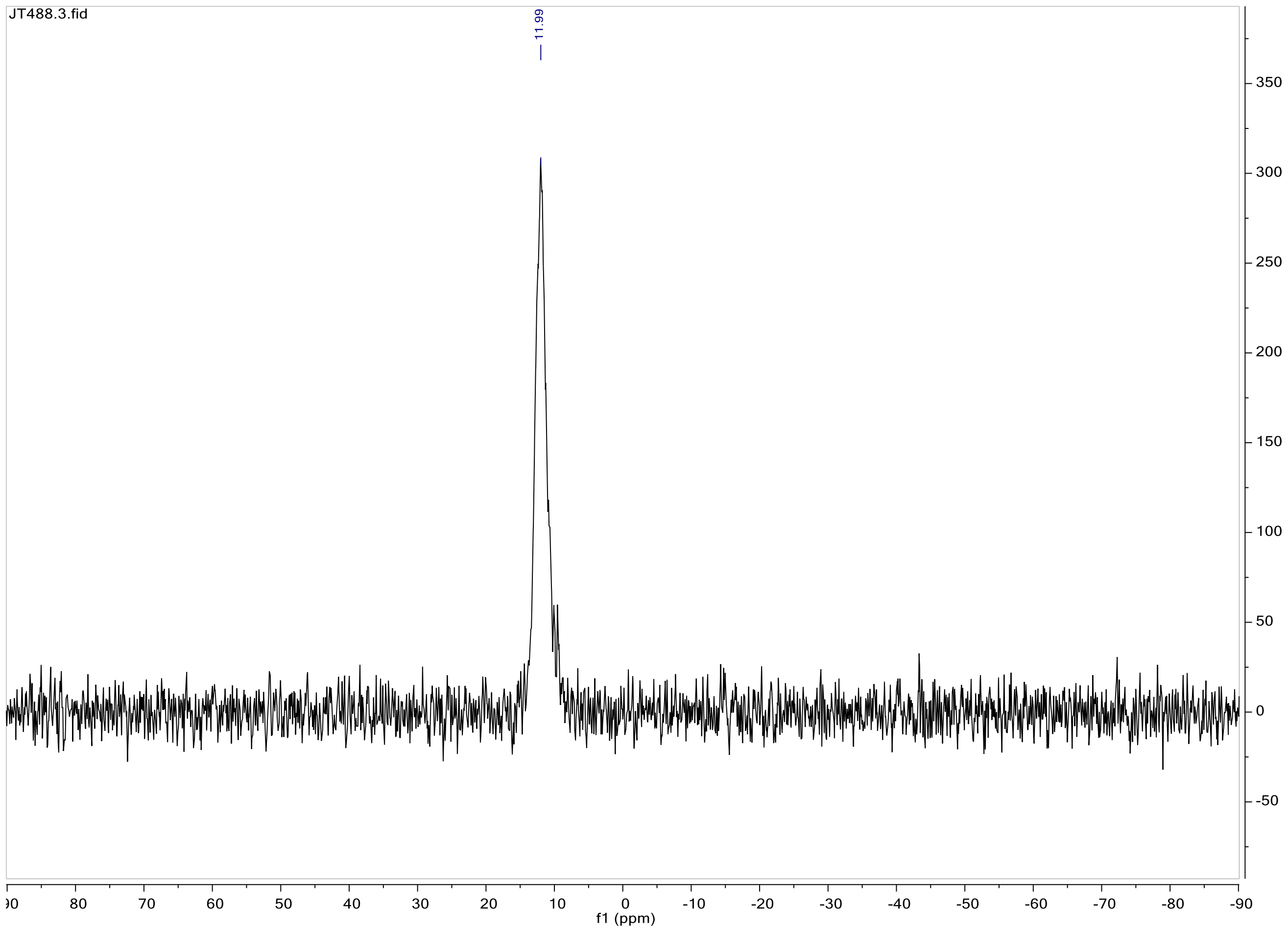

Supplementary Figure 90: ^{11}B NMR of **11a** in acetonitrile-d_3 at 25 °C.

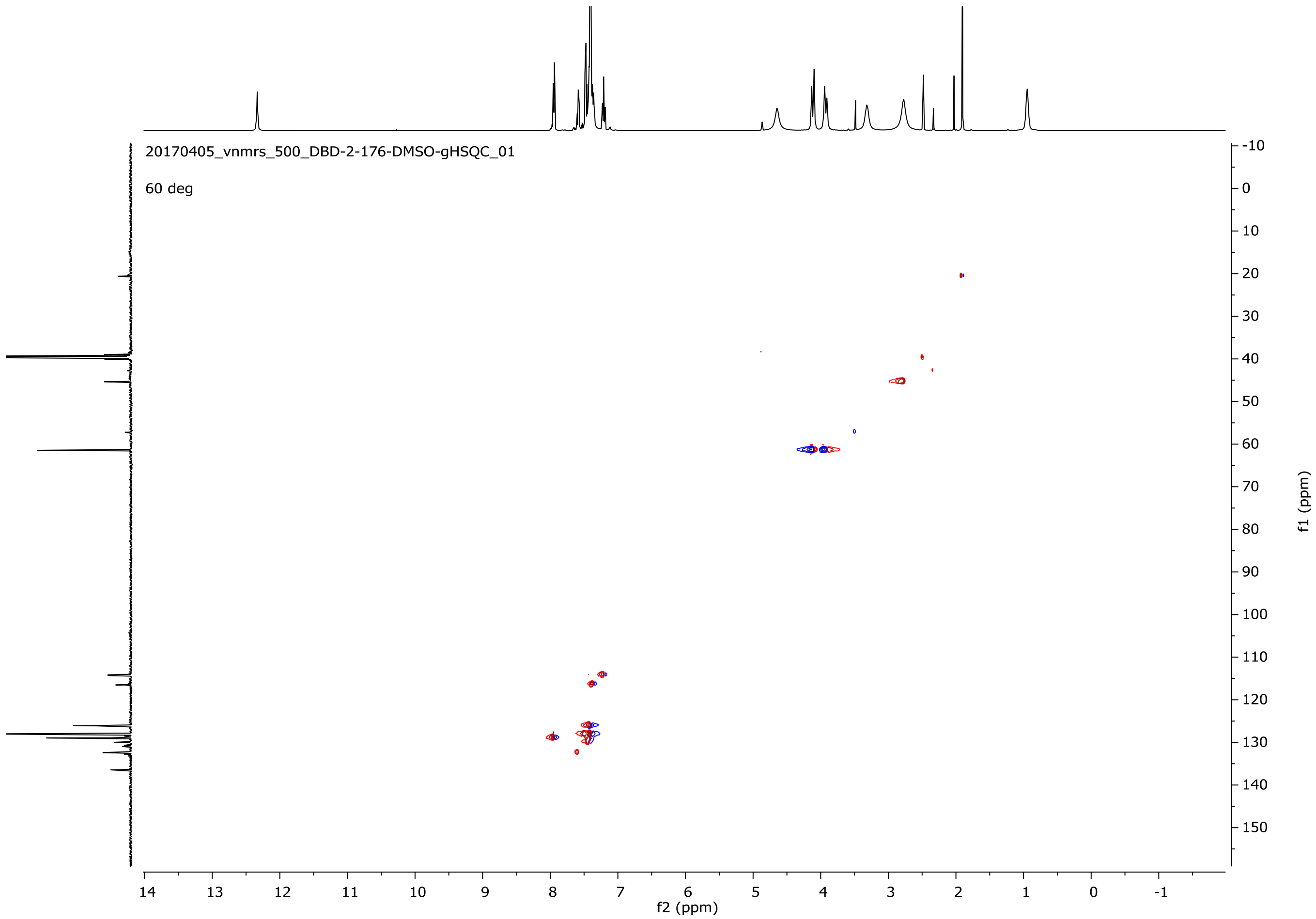

Supplementary Figure 91: ^{1}H-^{13}C HSQC NMR of **11a** in dimethylsulfoxide-d_3 at 60 °C.

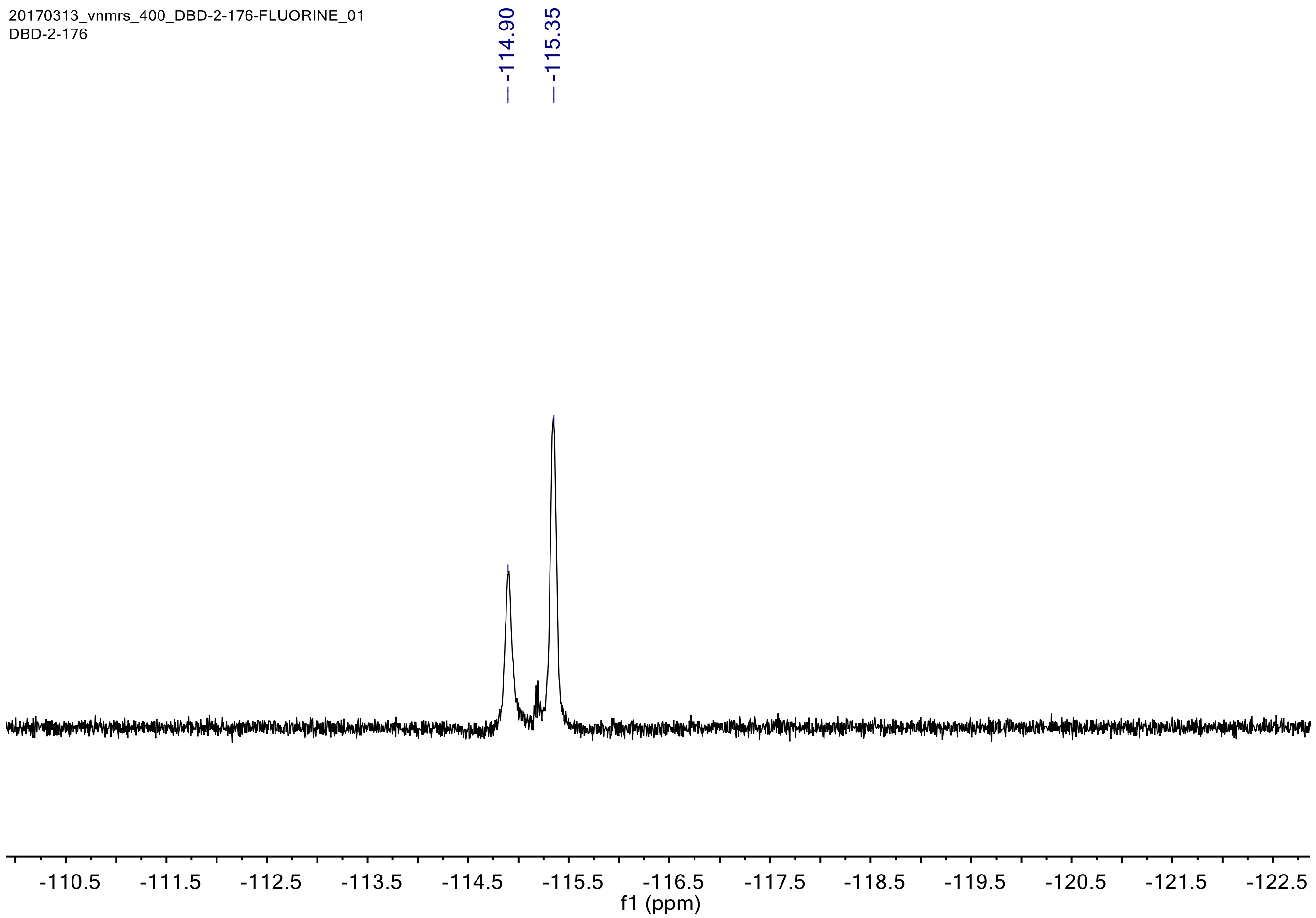

Supplementary Figure 92: ^{19}F NMR of **11a** in acetonitrile-d_3 at 25 °C.

Compound **11b**

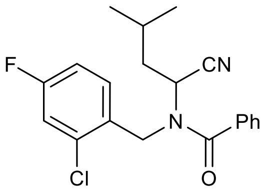

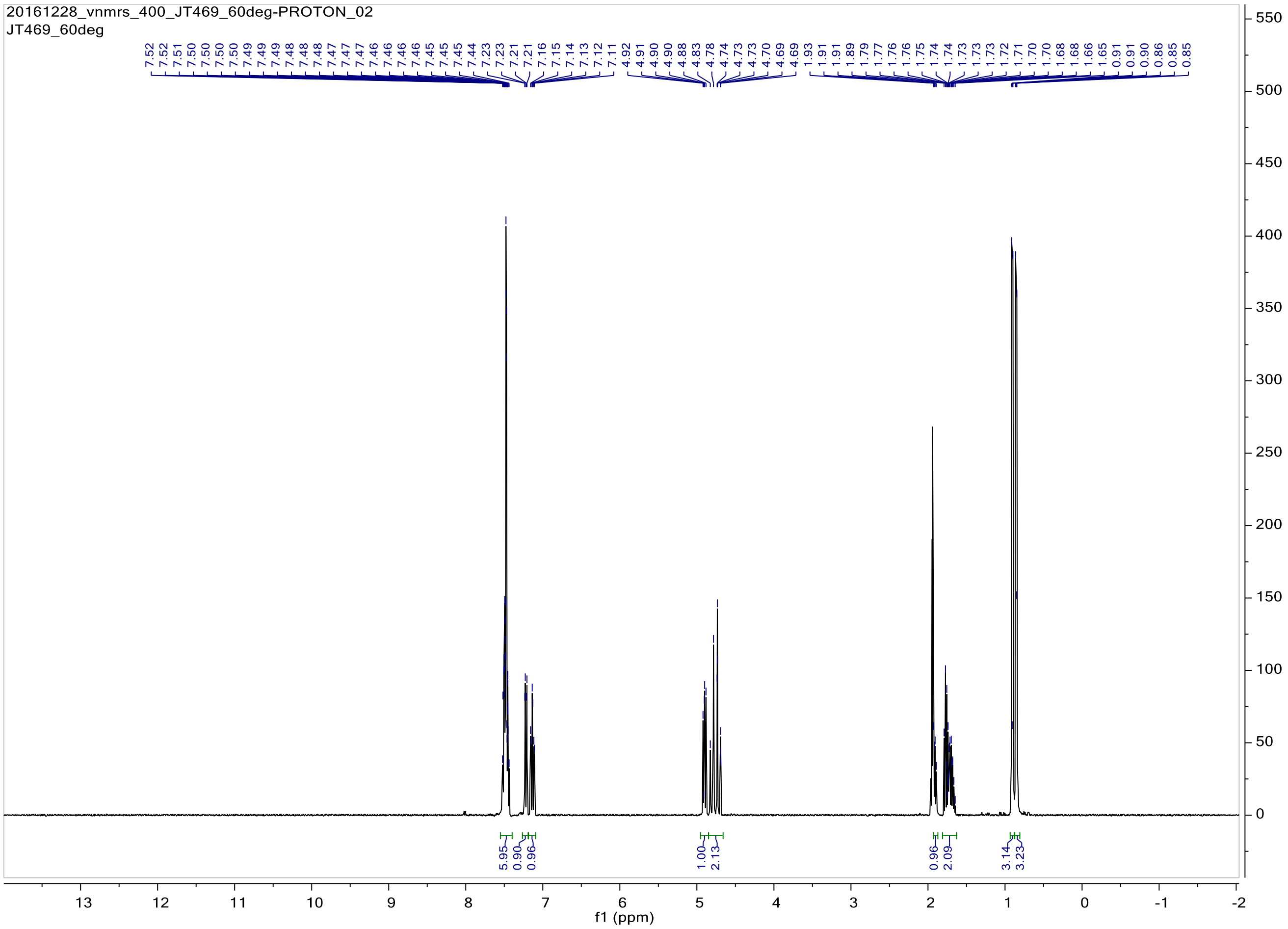

Supplementary Figure 93: ^{1}H NMR of **11b** in acetonitrile-d_3 at 60 °C.

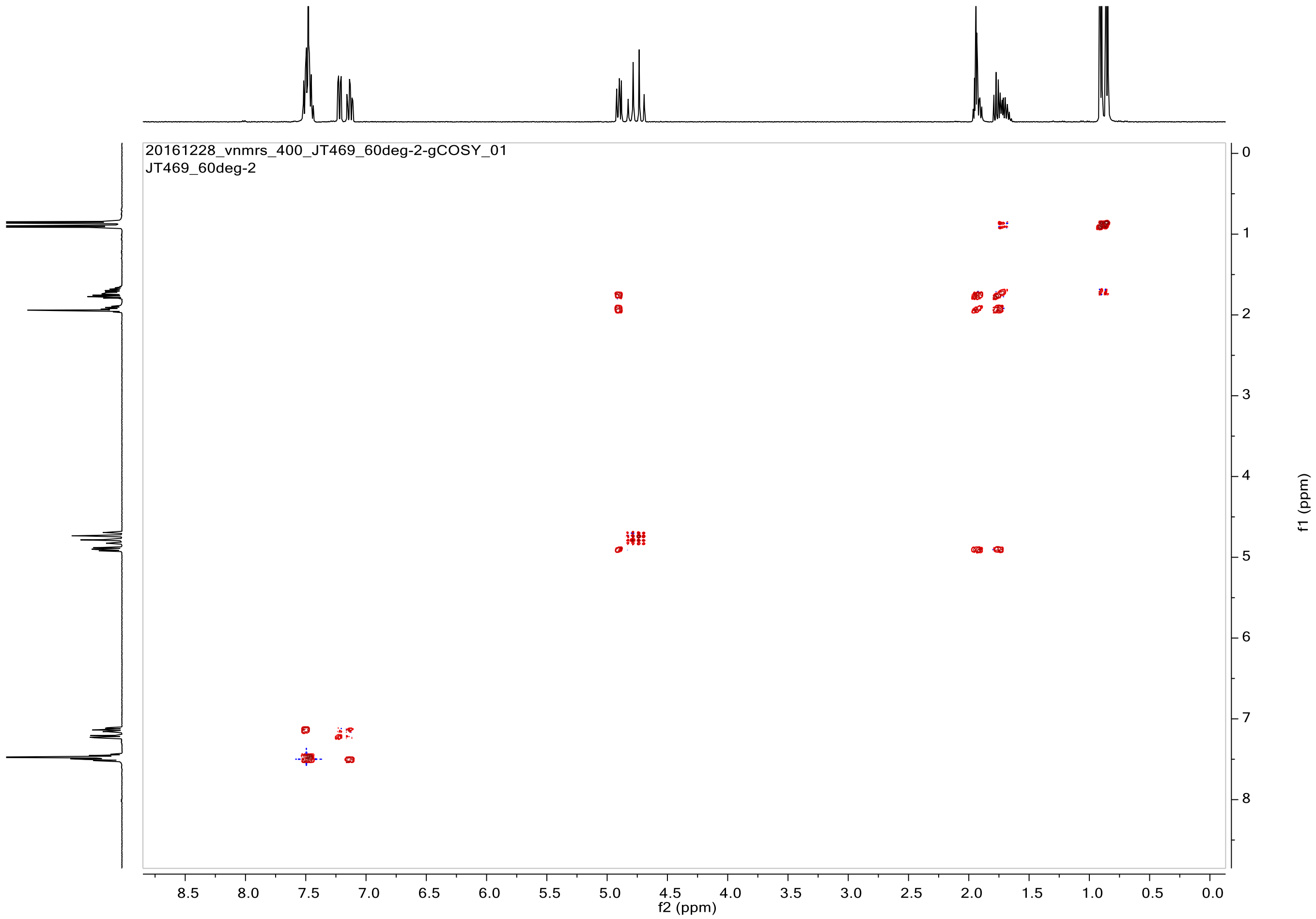

Supplementary Figure 94: ^{1}H-^{1}H COSY of **11b** in acetonitrile-d_3 at 60 °C.

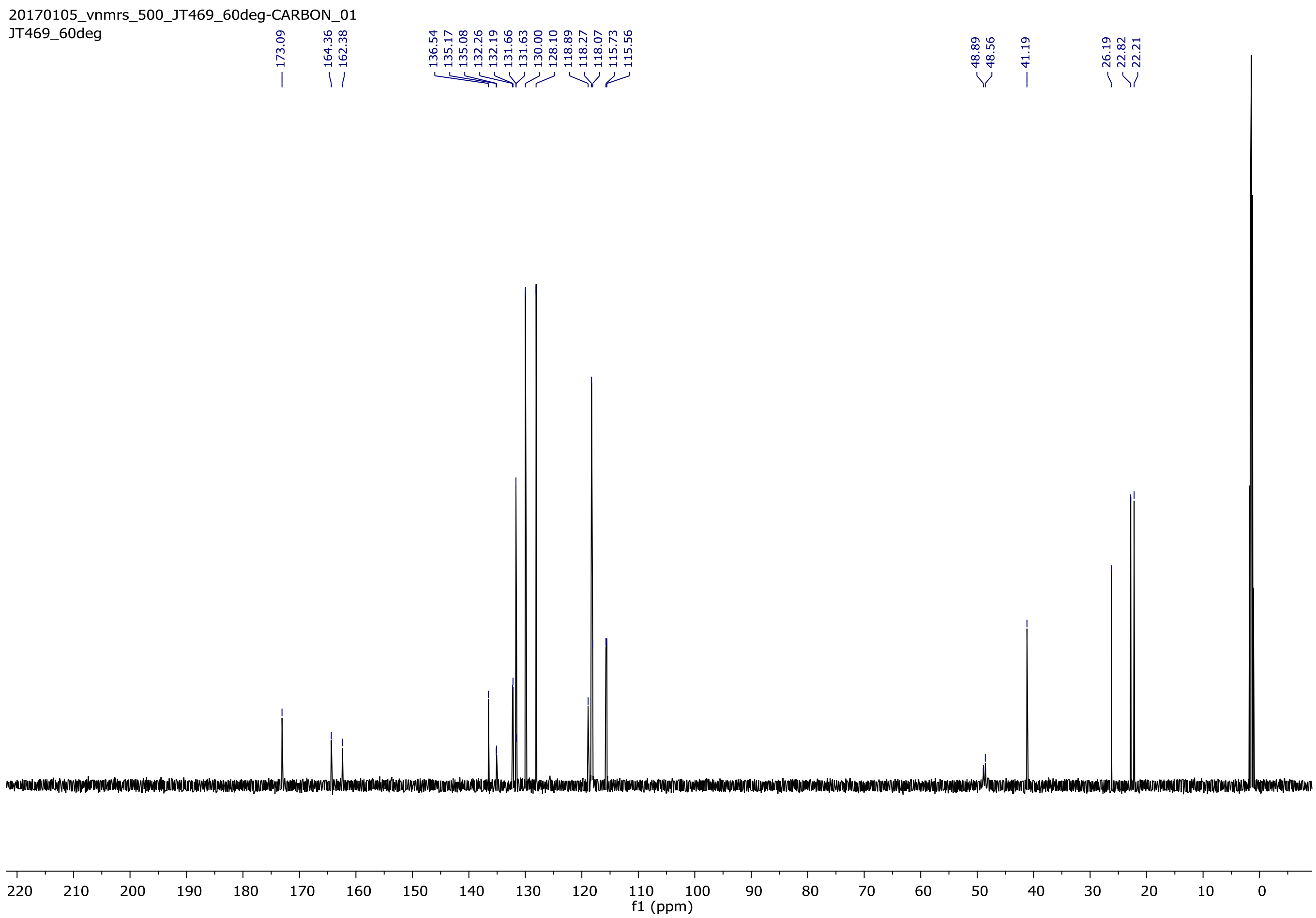

Supplementary Figure 95: ^{13}C NMR of **11b** in acetonitrile-d_6 at 60 °C.

730
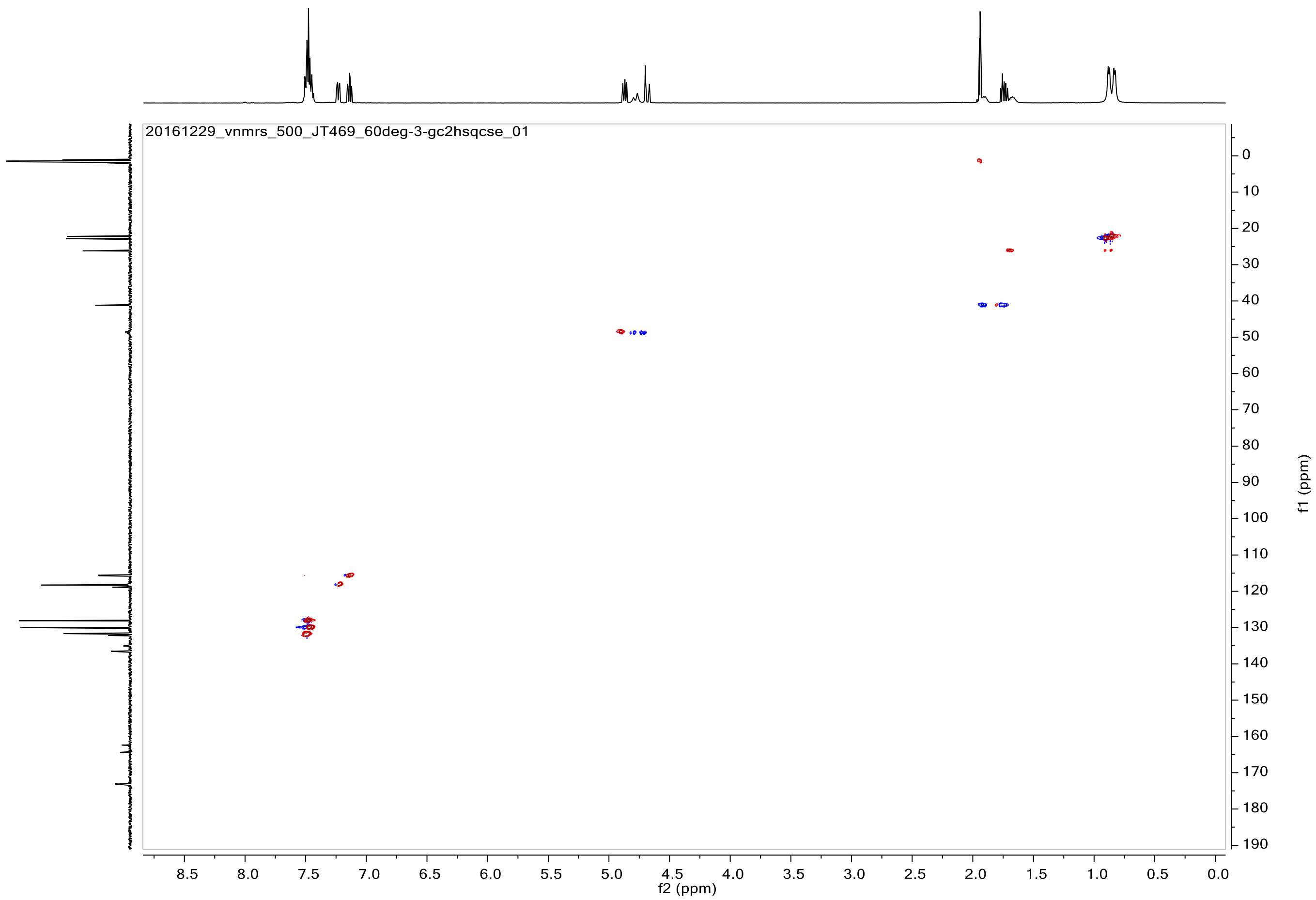

731 **Supplementary Figure 96:** ^{1}H-^{13}C HSQC NMR of **11b** in acetonitrile-d_3 at 60 °C.

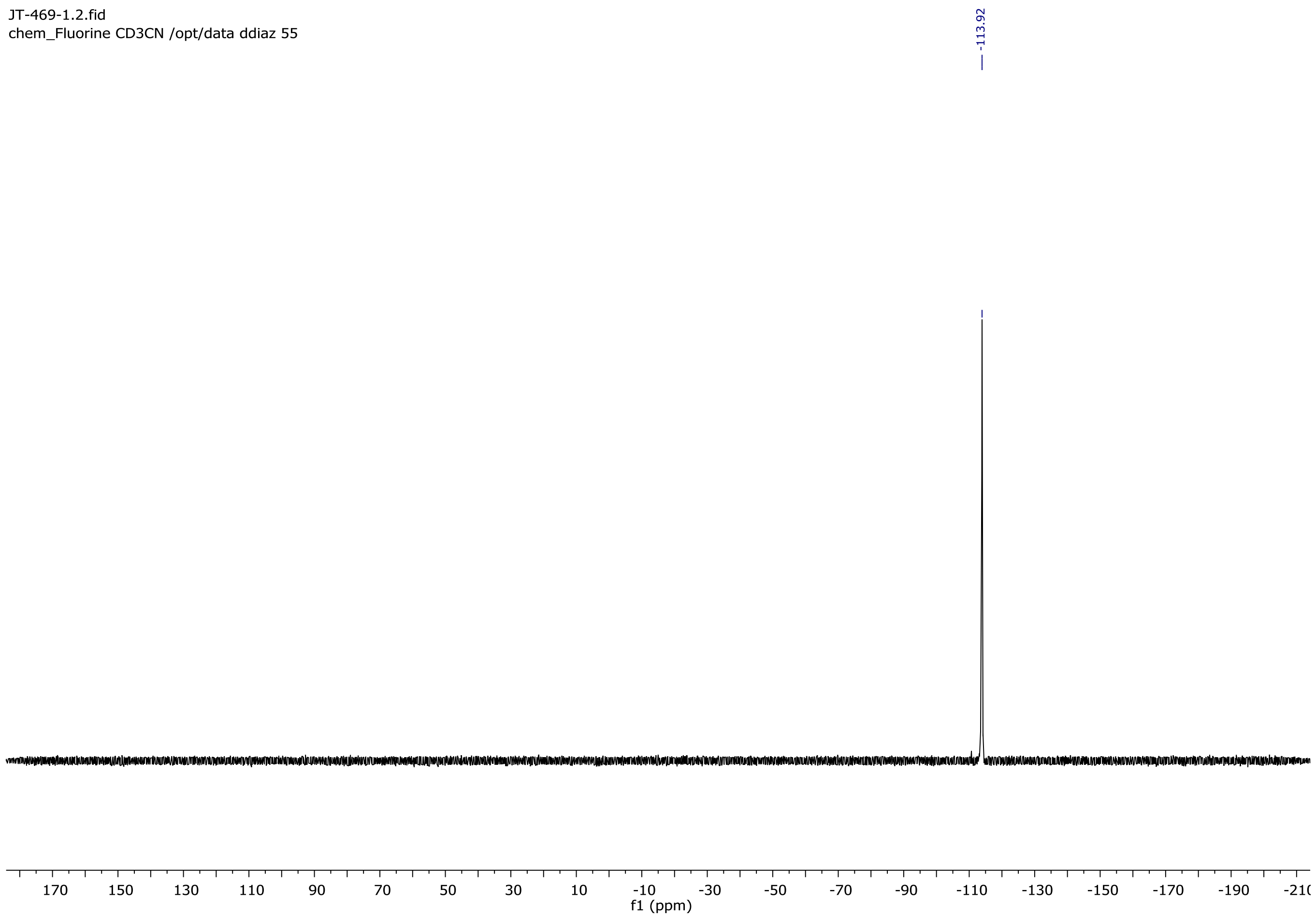

Supplementary Figure 97: ^{19}F NMR of **11b** in acetonitrile-d_3 at 25 °C.

Compound **11c**

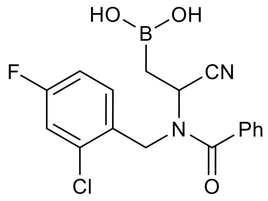

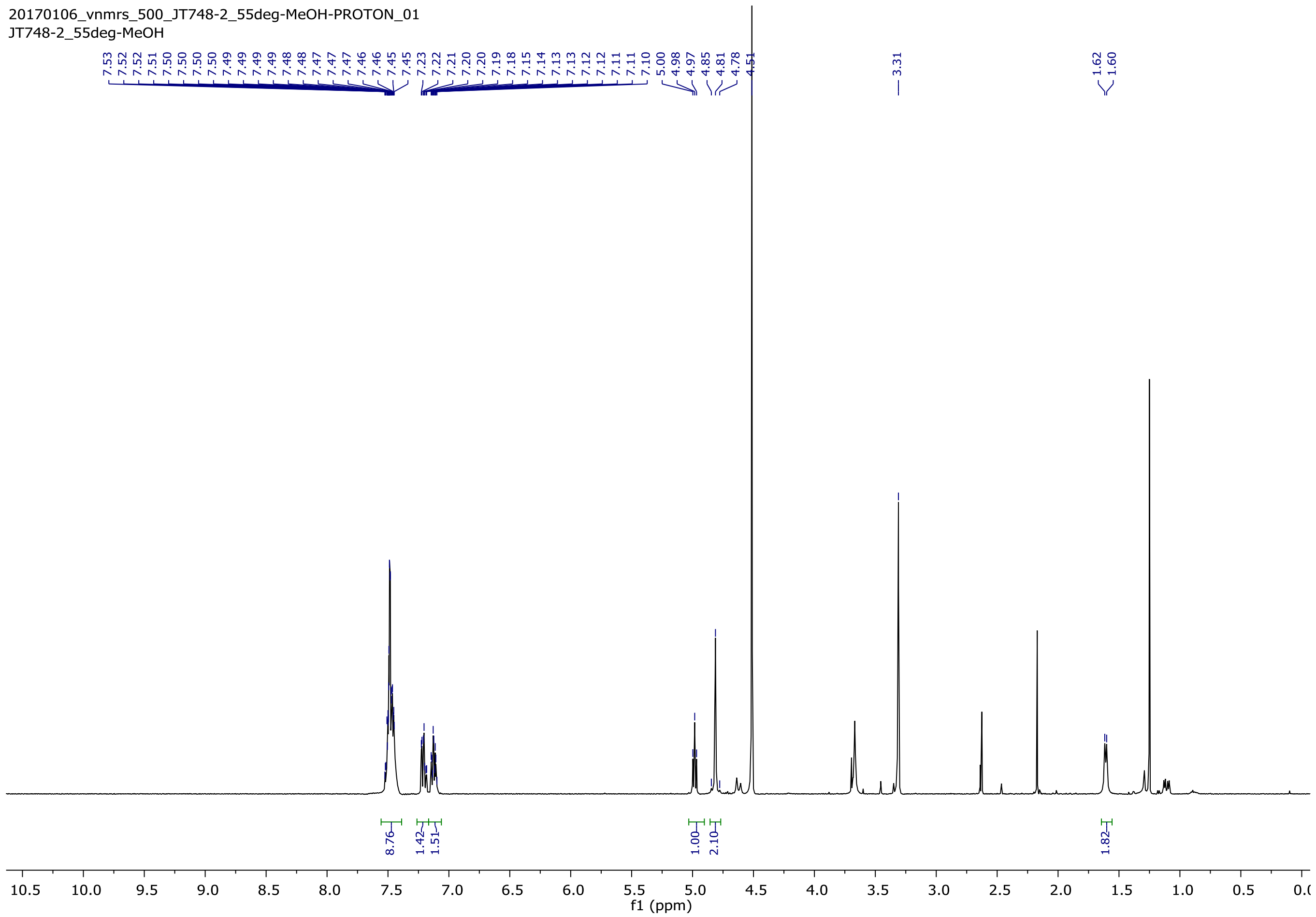

Supplementary Figure 98: ^{1}H NMR of **11c** in methanol-d_4 at 55 °C.

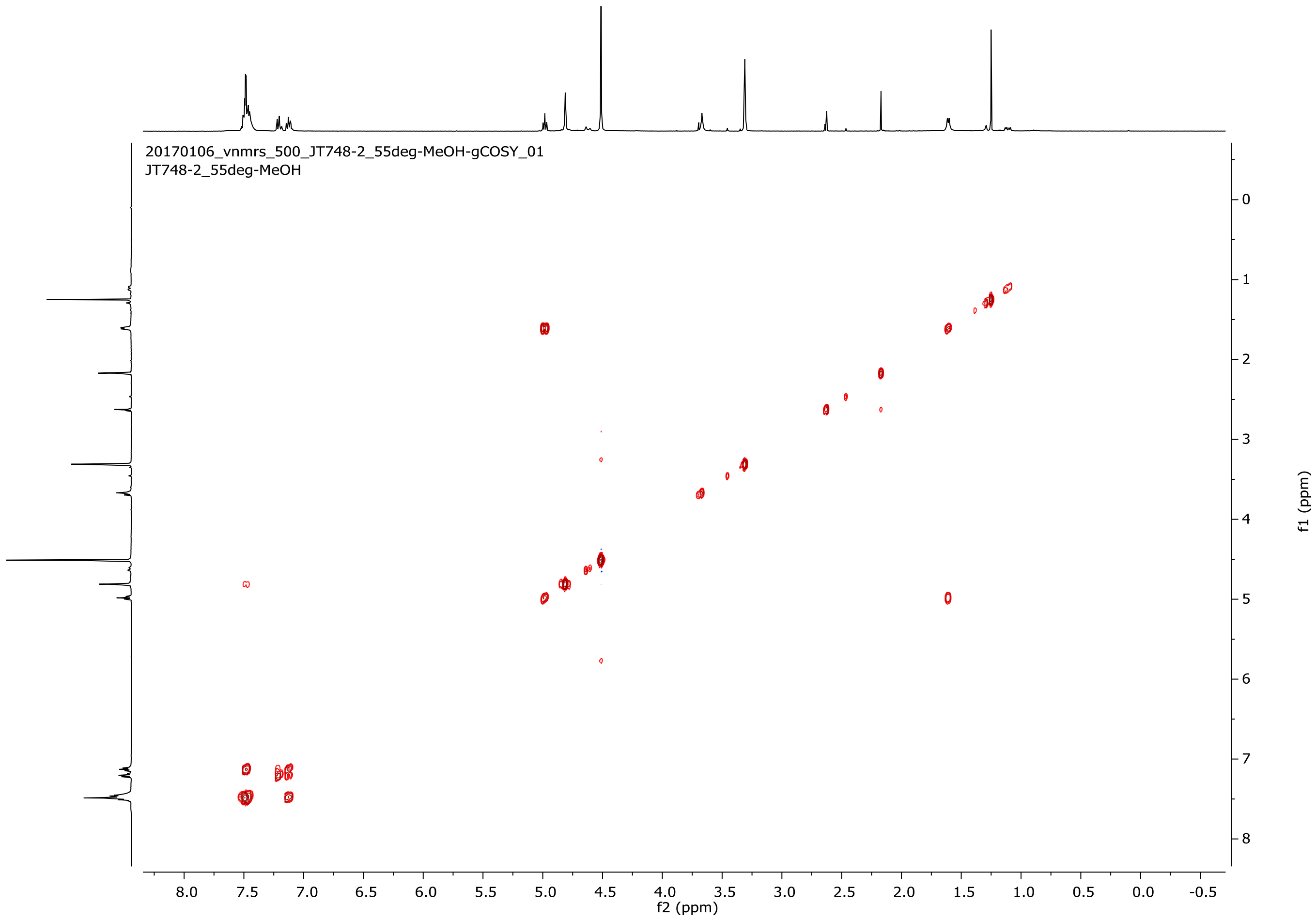

Supplementary Figure 99: ^{1}H-^{1}H COSY of **11c** in methanol-d_4 at 55 °C.

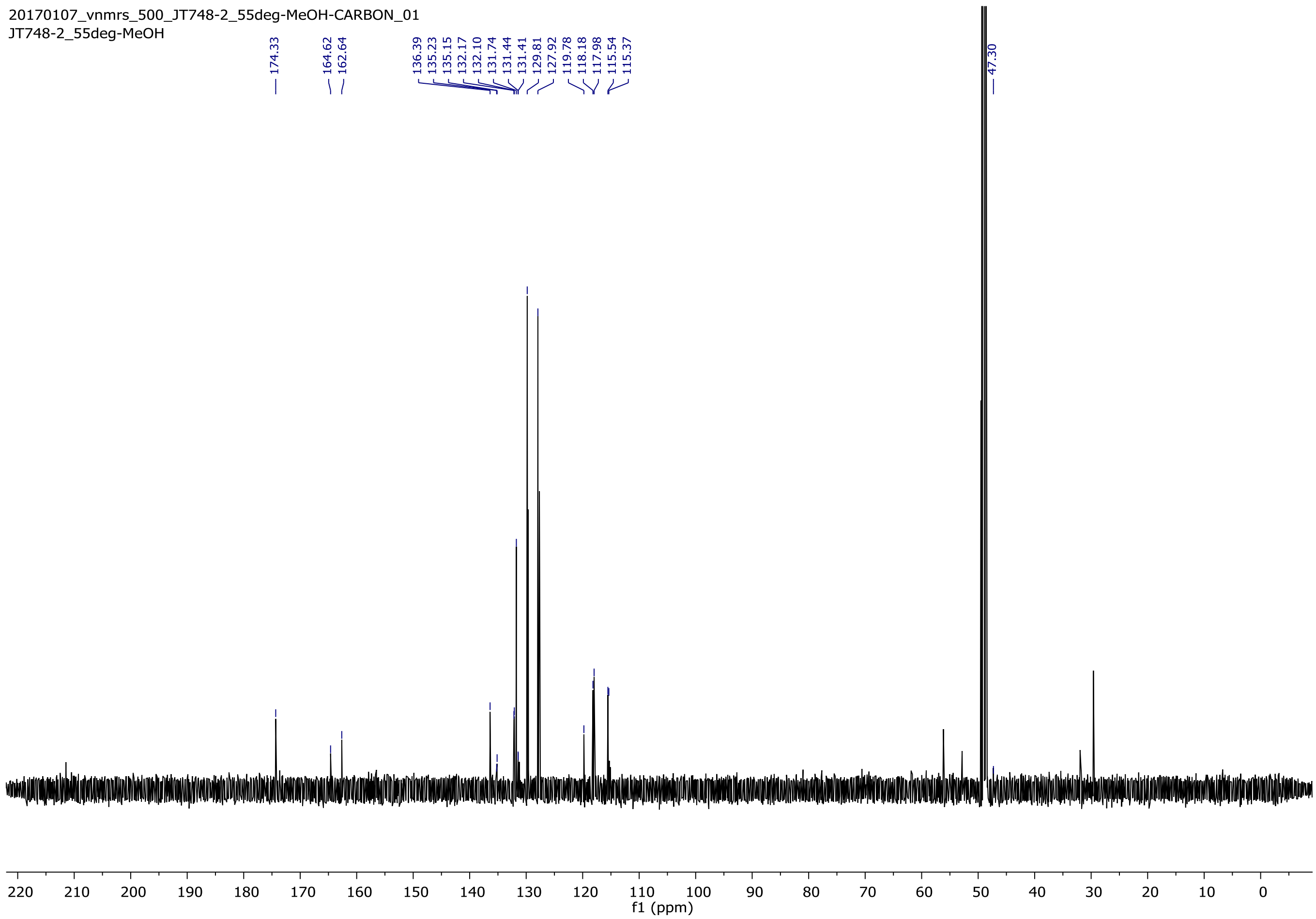

Supplementary Figure 100: ^{13}C NMR of **11c** in methanol-d_4 at 55 °C.

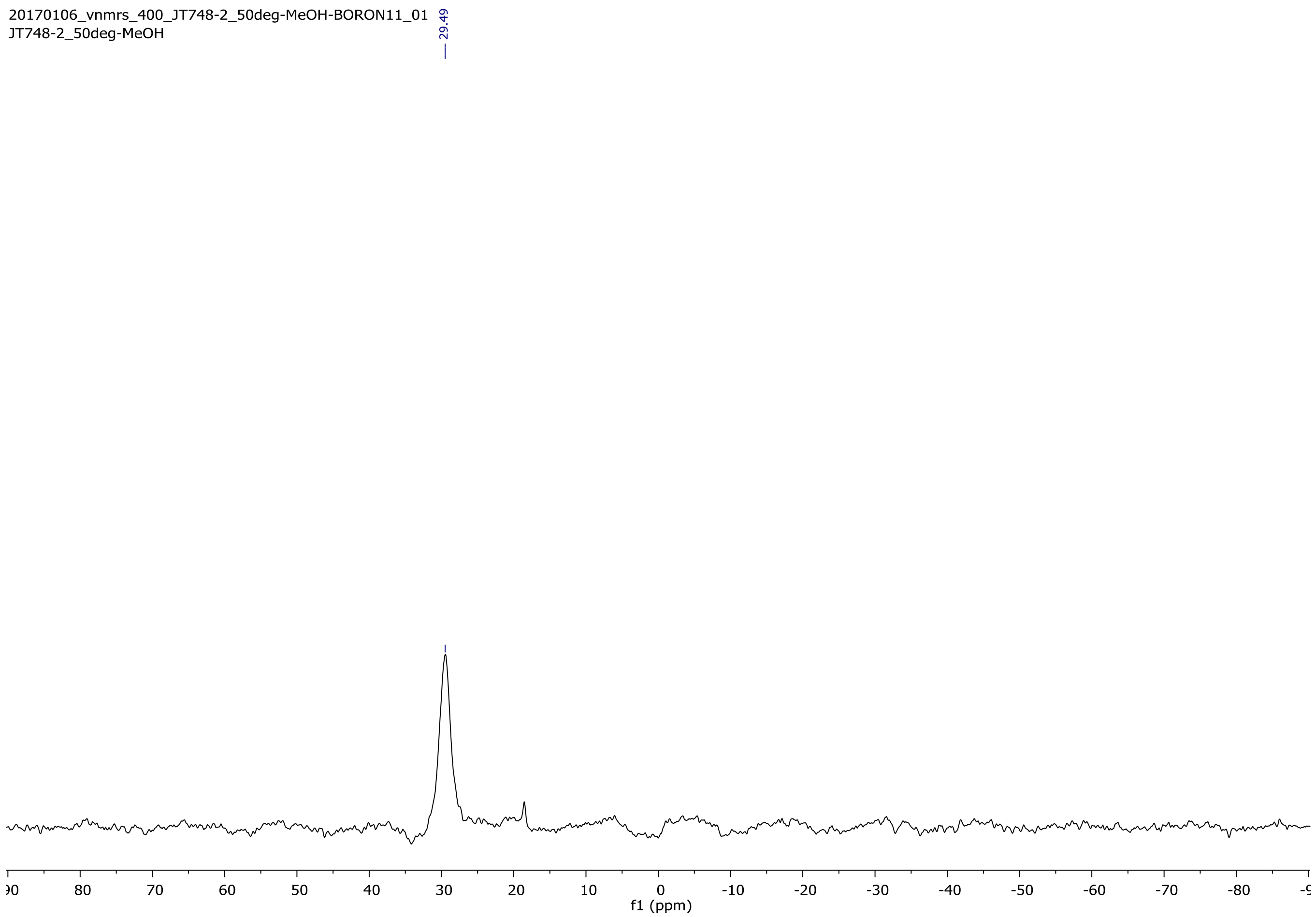

Supplementary Figure 101: ^{11}B NMR of **11c** in methanol-d_4 at 50 °C.

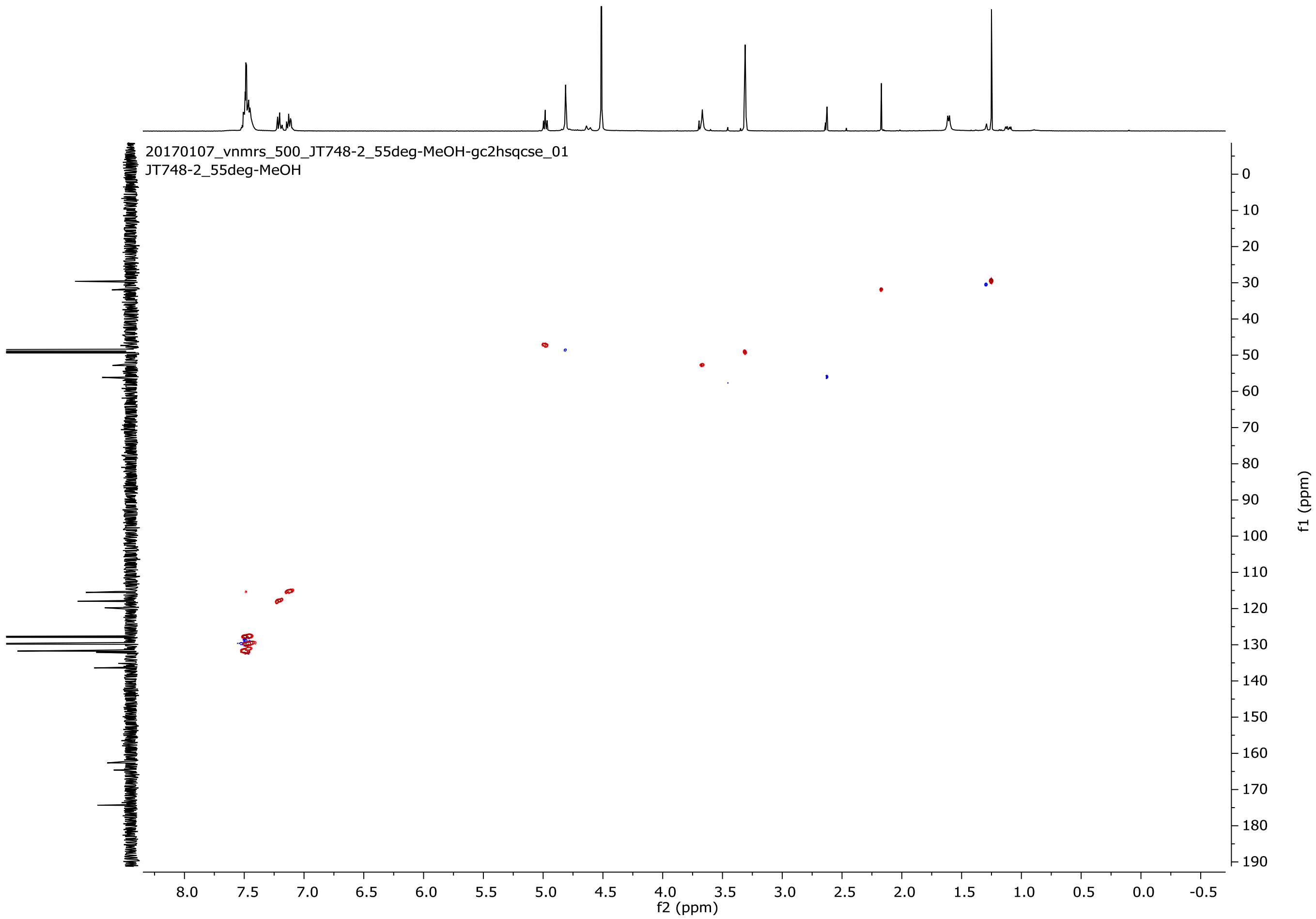

Supplementary Figure 102: ^{1}H-^{13}C HSQC NMR of **11c** in methanol-*d₄* at 55 °C.

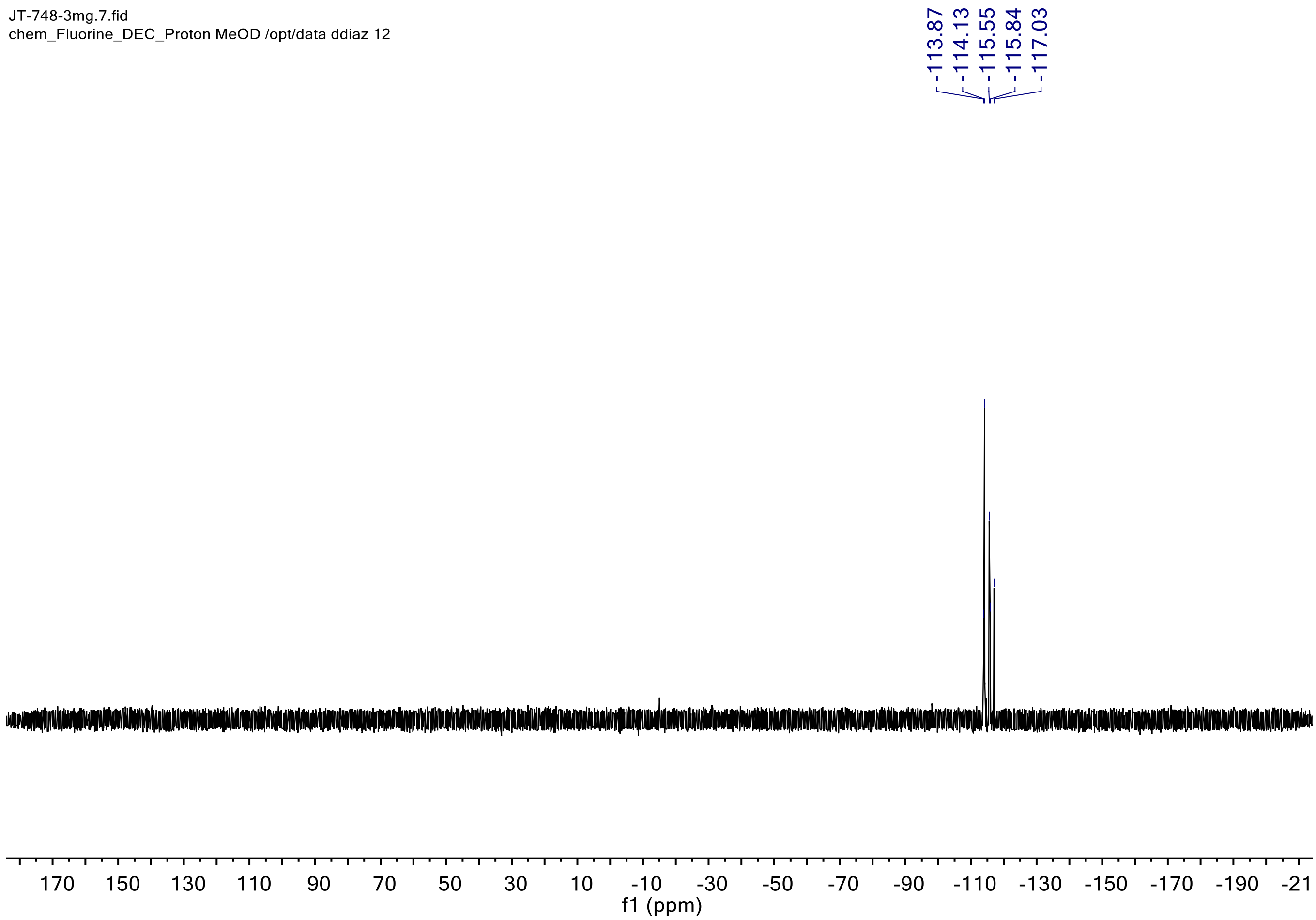

Supplementary Figure 103: ^{19}F NMR of **11c** in methanol-d_4 at 25 °C.

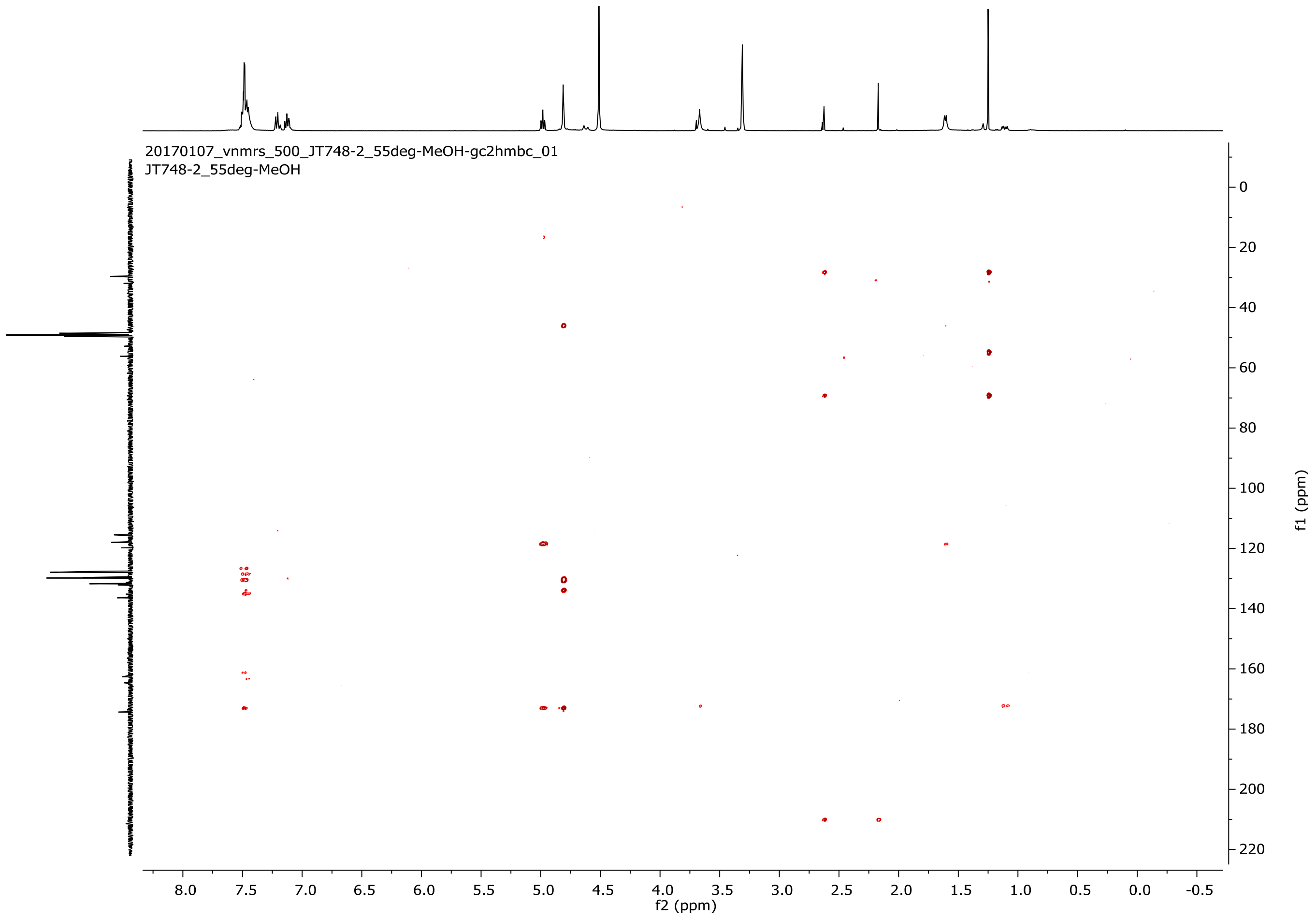

Supplementary Figure 104: ^{1}H-^{13}C HMBC NMR of **11c** in methanol-d_4 at 55 °C.

Supplementary References

1. St. Denis, J. D. *et al.* Boron-containing enamine and enamide linchpins in the synthesis of nitrogen heterocycles. *J. Am. Chem. Soc.* **136,** 17669–17673 (2014).
2. He, Z. & Yudin, A. K. Amphoteric α-boryl aldehydes. *J. Am. Chem. Soc.* **133,** 13770–13773 (2011).
3. He, Z., Trinchera, P., Adachi, S., St. Denis, J. D. & Yudin, A. K. Oxidative geminal functionalization of organoboron compounds. *Angew. Chem. Int. Ed.* **51,** 11092–11096 (2012).
4. Diaz, D. B. *et al.* Synthesis of aminoboronic acid derivatives from amines and amphoteric boryl carbonyl compounds. *Angew. Chemie Int. Ed.* **55,** 12659–12663 (2016).
5. Adachi, S. *et al.* Facile synthesis of borofragments and their evaluation in activity-based protein profiling. *Chem. Commun.* **51,** 3608–3611 (2015).
6. Weerapana, E. *et al.* Quantitative reactivity profiling predicts functional cysteines in proteomes. *Nature* **468,** 790–795 (2010).
