# Supplementary material for: Multicomponent mapping of boron chemotypes furnishes selective enzyme inhibitors
Source: Nat Commun. 2017 Nov 24;8:1760. doi: 10.1038/s41467-017-01319-4 (PMC5701053; doi:10.1038/s41467-017-01319-4)
Supplement: Supplementary file 3 — Description of Additional Supplementary Files [file 41467_2017_1319_MOESM3_ESM.pdf]

Description of Additional Supplementary Files

File Name: Supplementary Data 1
Description: **Proteomics datasets and filtered SH data.** Data was filtered for known Serine Hydrolases which had at least two unique quantified peptides per dataset in both biological replicates. Median ratios across replicates are displayed as inhibitor/DMSO treatment. Full datasets are shown in tabs 2-5. Ratios are displayed as DMSO/inhibitor treatment.
